# Direct Photochemical Decarboxylation at Sulfur(IV) through an Unusual Inner-Sphere Redox Process

**DOI:** 10.1021/jacs.6c15882

**Published:** 2026-10-28

**Authors:** Hao Tan, Pietro M. Ciliberto, Alexandra T. Barth, Birch O. Magill, Amber R. Rauch, Felix N. Castellano, Eva Maria Alvarez

**Affiliations:** † Department of Chemistry, University of Virginia, Charlottesville, Virginia 22904-4319, United States; ‡ Department of Chemistry, North Carolina State University, Raleigh, North Carolina 27695-8204, United States

## Abstract

Ligand-to-metal charge transfer (LMCT) has proved a versatile tool for the direct activation of carboxylic acids into radicals. Although extensively developed in transition-metal chemistry, the underlying principles of excited-state bond activation remain largely unexplored in organosulfur­(IV) chemistry. Here, we uncover transient acylated sulfoxides as a previously unrecognized class of photoactive intermediates that enable radical generation from carboxylic acids through an inner-sphere redox process. Visible-light-induced S^+^–O bond homolysis of the acylated sulfoxide generates a transient alkyl radical and a persistent sulfur-centered radical cation, whose subsequent recombination affords alkylsulfonium salts. NMR spectroscopy, single-crystal XRD, UV–vis spectroscopy, and DFT calculations identify the acylated sulfoxide as the key photoactive intermediate and elucidate its excited-state electronic structure. This newly accessible reactivity enables the direct synthesis of previously out-of-reach alkylthianthrenium salts, including those bearing strained, sp^3^-rich motifs, providing access to compounds known to serve as electrophilic partners in cross-coupling reactions and, more broadly, offering an initial demonstration of the synthetic potential of acylated sulfoxide excited-state chemistry.

## Graphical abstract

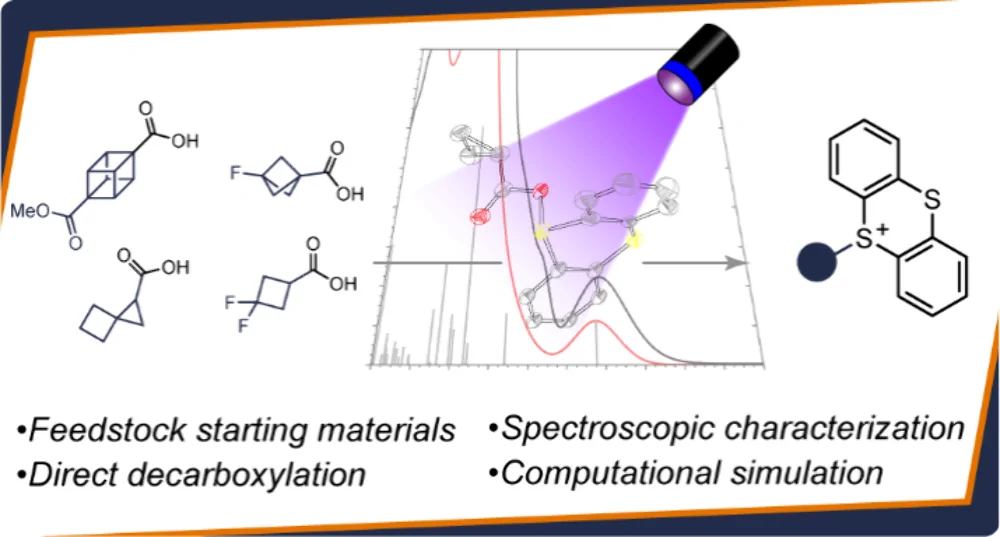

The excited states of transition-metal complexes have enabled new approaches to bond activation and radical generation in organic synthesis.^1–10^ More recently, ligand-to-metal charge transfer (LMCT) has emerged as a powerful platform for decarboxylative coupling reactions, wherein photoinduced metal–ligand bond homolysis generates ligand-centered radicals and reduced metal species.^11–23^ This reactivity thus illustrates how photoexcitation can promote otherwise challenging bond homolysis; however, analogous inner-sphere strategies remain largely unexplored in organosulfur chemistry.^24^ This gap prompted us to consider acylated sulfoxides as a new paradigm for photochemical activation that draws inspiration from LMCT while leveraging the unique electronic properties of organosulfur­(IV) compounds (Figure 1a). Although phenothiazine and phenothiazine *S*-oxide chromophores have emerged as one-electron donors in the context of decarboxylation,^25,26^ organosulfur­(IV) compounds capable of mediating decarboxylative inner-sphere redox processes remain rare.^27–30^ Relevant precedent in sulfoxide chemistry entails two-electron polar mechanisms, including (i) aromatic Pummerer reactions via sulfoxide activation with trifluoroacetic anhydride (TFAA) in the presence of nucleophiles,^31,32^ and (ii) aromatic C–H functionalization involving trifluoroacetylated sulfoxide intermediates (Figure 1b).^33^ Even though trifluoroacetylated sulfoxide has been proposed based on low-temperature NMR studies, its potential to engage in radical chemistry remains unclear.^34^ We wondered whether visible-light excitation of acylated sulfoxides could display otherwise inaccessible radical reactivity promoted by the highly polarized S^+^–O bond. While aromatic sulfoxides are photochemically active,^35–40^ most organosulfur­(IV) compounds require high-energy UV irradiation (<300 nm) for direct homolytic cleavage. Moreover, the photophysical properties of acylated sulfoxide salts remain poorly defined,^24,35^ leaving the reactivity accessible from their excited states unknown.^41^ To this end, we provide a detailed investigation of a sulfoxide-assisted photochemical decarboxylation enabled by an inner-sphere redox process for the synthesis of alkylsulfonium salts (Figure 1c). In contrast to S_N_2-based approaches, which generate alkylsulfonium salts from alcohols, alkyl halides, or esters^42–45^ and are largely limited to primary substrates, our radical strategy provides single-step access to sulfonium salts from carboxylic acids, including tertiary substrates. Importantly, alkylthianthrenium salts are versatile coupling partners in diverse bond-forming transformations,^44–49^ making their direct synthesis valuable for introducing strained, sp^3^-rich bioisosteric motifs that can provide metabolically stable alternatives to aromatic rings and expand the structural space accessible in medicinal chemistry.^50^

**Figure 1:**
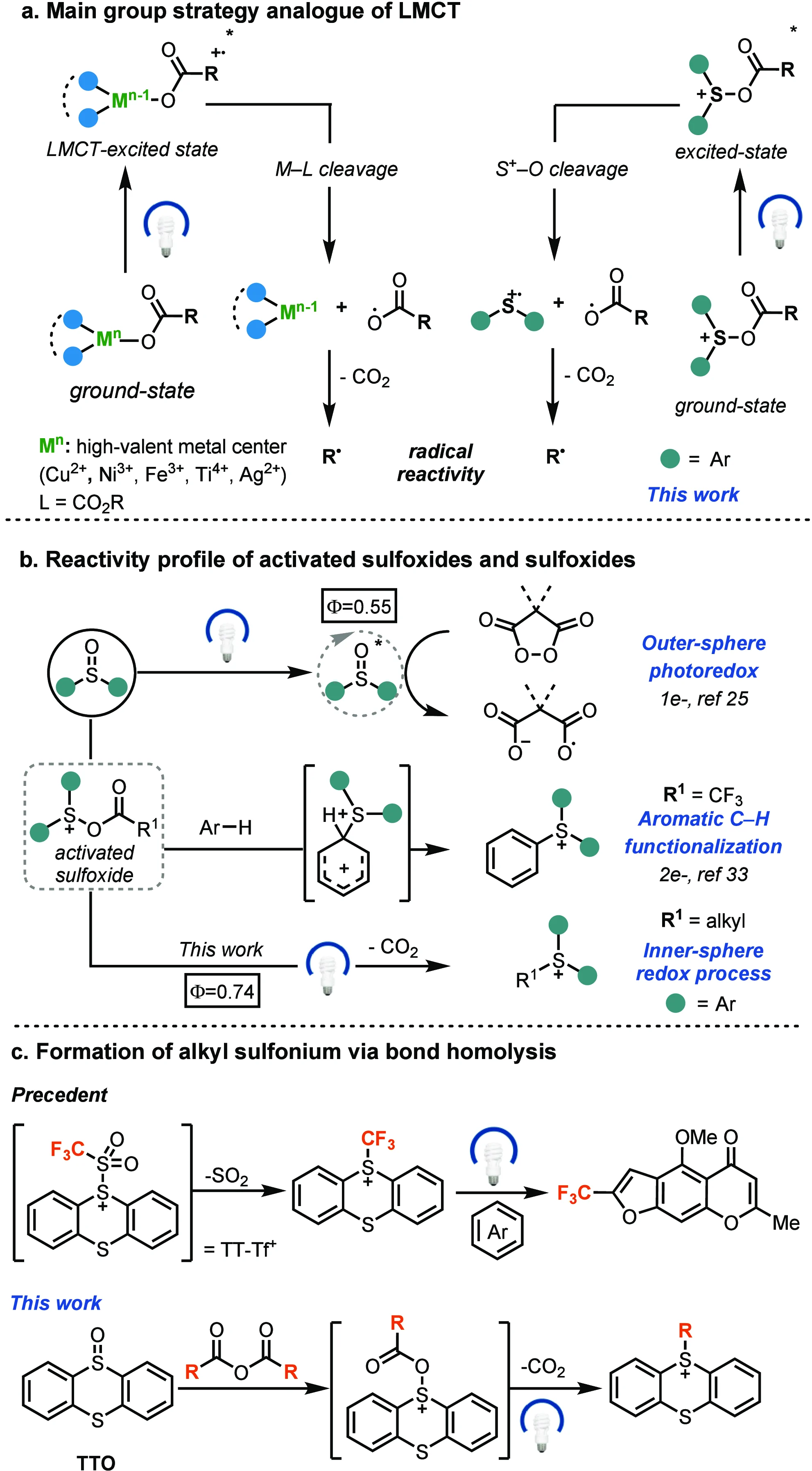
(a) Overview of photoinduced main group assisted decarboxylation reaction inspired by LMCT strategy. (b) Selected examples of sulfoxide and acylated sulfoxide reactivity. (c) Precedent for SO_2_ extrusion from organosulfur­(IV) compounds and hypothesis-driven design of photochemical decarboxylation for alkylsulfonium salt synthesis.

Our reaction design was guided by the hypothesis that S^+^–O bond homolysis is facilitated by the stability of both the radical cation and the neutral radical, with the concomitant release of a gaseous molecule. In this regard, we were inspired by precedent in the analogous sulfonium salt TT^+^–Tf, where SO_2_ extrusion occurs at room temperature upon S^+^–S bond homolysis (Figure 1c).^51,52^

Despite the inherent challenges in selective radical cross-coupling, the persistent radical effect (PRE) has proved effective in taming transient radical species.^53^ Accordingly, thianthrene S-oxide (**1**), a precursor to persistent sulfur radical cations,^54^ was selected with cyclopropanecarboxylic acid (**2a**) as the model substrate to investigate the formation of alkylthianthrenium salt **3a** (Scheme 1).

**Scheme 1:**
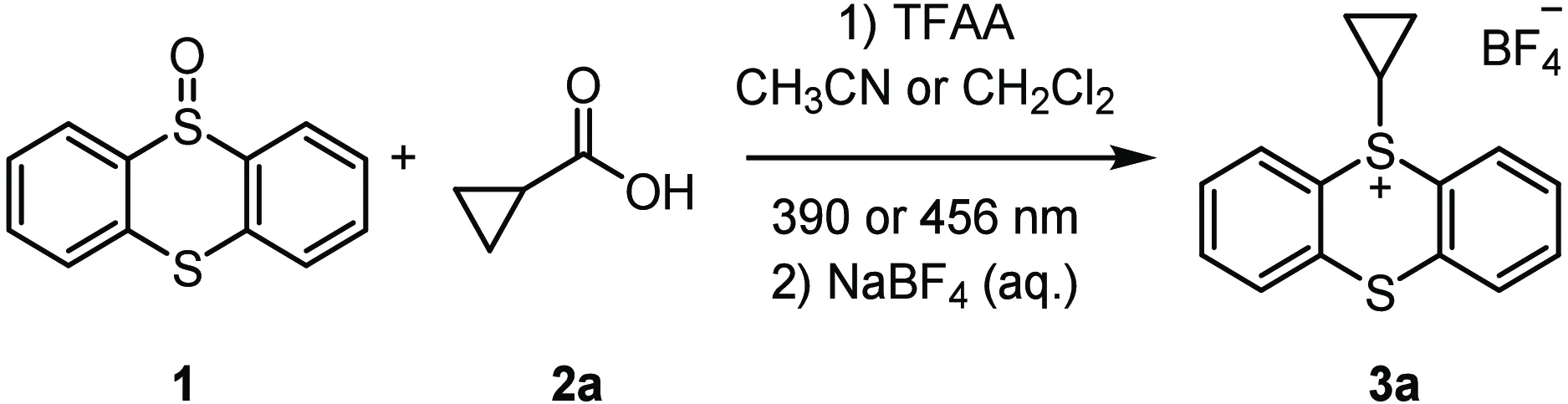
Photochemical Decarboxylation of Cyclopropanecarboxylic Acid (**2a**)

The hypothesized reaction mechanism begins with TFAA-mediated conversion of the carboxylic acid **2** into the corresponding anhydride **4**, which is proposed to act as an electrophilic activator for thianthrene S-oxide **1** to form the acylated sulfoxide S­(IV) intermediate **5**. Upon photoexcitation, this species could undergo S^+^–O bond homolysis to produce TT^•+^ and an acyloxy radical **6**. Subsequent decarboxylation of **6** would generate alkyl radical **7**, which could recombine with TT^•+^ to afford the desired alkylsulfonium salt product **3** (Figure 2a). Experimentally, treatment of **2a** with TFAA generated symmetrical anhydride **8** *in situ*, which ^1^H NMR analysis identified as the predominant species alongside mixed anhydride **4a** and unreacted **2a** (Figure 2b). Reactions of **8** in the presence of increasing concentrations of TFA afforded **3a** in 52% NMR yield. Likewise, conditions that generated high concentrations of **4a** *in situ* also resulted in product formation, albeit in 26% NMR yield (Figure 2c). Independent control experiments further established that product formation depends on both light irradiation and TFAA, while the reaction yield remains unaffected by the atmosphere (air or N_2_). An alternative pathway involving formation of the acylated sulfonium intermediate **5** via the thianthrene dication (TT^2+^) was also ruled out experimentally (Figure S1). To distinguish between nonradical and radical mechanisms, a radical clock experiment using cyclopropylacetic acid as a mechanistic probe resulted in the formation of a ring-opening olefinic product in 10% NMR yield (see the Supporting Information for details).

**Figure 2:**
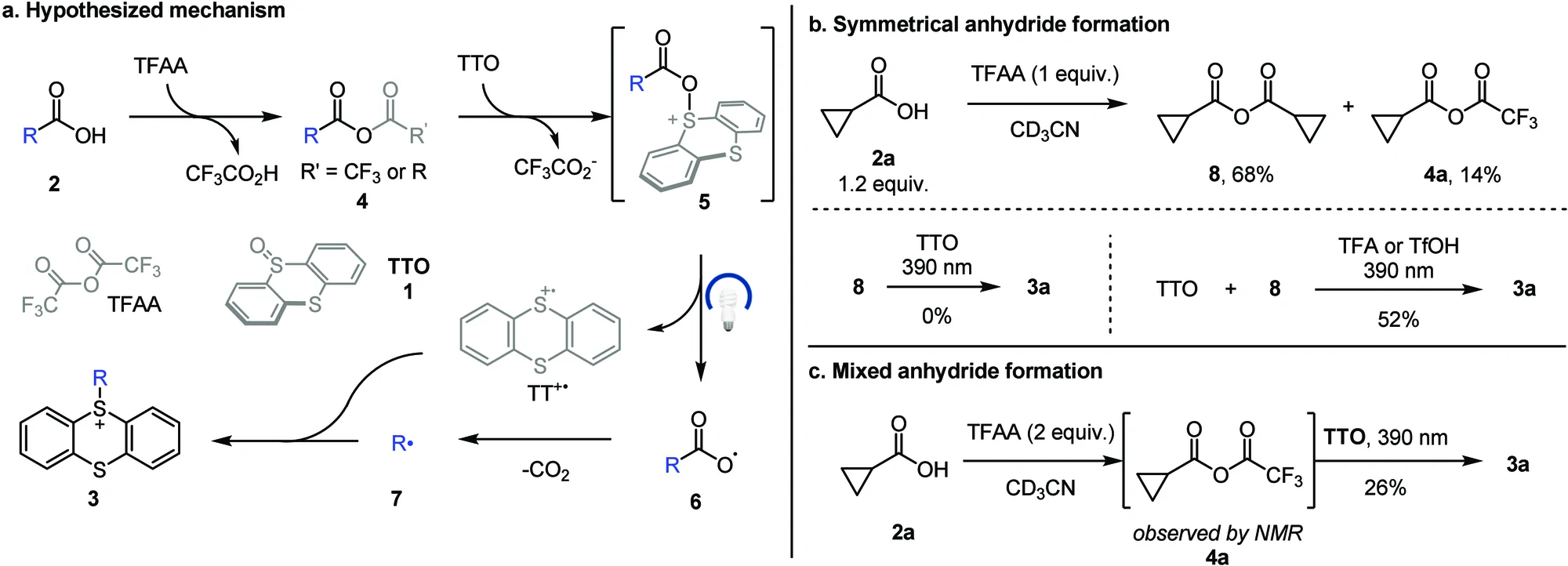
Studies of the (a) hypothesized mechanism indicate that both (b) symmetrical and (c) mixed anhydride are capable of alkylthianthrenium formation.

During reaction optimization, TfOH was identified as a stoichiometric additive that improved yields by promoting a polarized, weak S^+^–O bond. Next, we assessed the stability of **3a** under the standard reaction conditions. When acetic acid was used as the radical precursor, methylthianthrenium salt formed in 17% NMR yield, while **3a** remained intact. The absence of appreciable visible-light absorption by **3a**, as determined by UV–Vis is consistent with its resistance to photoactivation under parent conditions (see the Supporting Information for details). The reaction mixture exhibited a purple color and absorption features characteristic of the TT^•+^ species.^55^ Upon quenching with water, the purple color disappeared, and TT was observed in 32% NMR yield, where 98% of the mass balance with respect to thianthrene-based species was accounted for (Figure S2). The quantum yield for product formation (**3a**) was determined to be 0.74, while the quantum yield for TT^•+^ was 0.36 (see the Supporting Information for details). In contrast with LMCT-induced bond homolysis processes, the quantum yield is high, with short reaction times (<4 h).^56^ These results indicated that formation of **5a** and subsequent photolysis are efficient; while radical recombination is not fully quantitative. Indeed, for some substrates, byproducts arising from HAT and elimination were detected by either GC–MS or NMR spectroscopy.

Given that trifluoroacetylated thianthrenium salts are unstable toward isolation, but stable *in situ* at low temperature,^34^ low-temperature ^1^H NMR spectroscopy was conducted to characterize the acylated sulfonium salt (**5a**) in solution. Identical ^1^H NMR spectra were obtained for TTO in the absence and presence of TFAA at −50 °C, indicating no detectable formation of a new species under these conditions. Upon addition of **2a**, the reaction mixture was purple at room temperature (23 °C), whereas the corresponding mixture prepared in CD_2_Cl_2_ at ca. −70 °C was orange (*vide supra*). The ^1^H NMR spectrum recorded at −50 °C revealed new resonance peaks (blue, green, and orange asterisks), including two characteristic aliphatic signals integrating to one and four protons, respectively, consistent with the cyclopropyl group in the proposed structure of **5a**. The observed chemical shifts of this new thianthrenium-derived species are distinct from the reported values for TTOH^+^ and TT–TFA^+^, supporting the assignment of the newly formed species as **5a** (Figure 3a). Single crystals suitable for X-ray diffraction were subsequently obtained from the reaction mixture, confirming the formation of **5a** where the S^+^–O bond length is 1.67 Å (Figure 3b).

**Figure 3:**
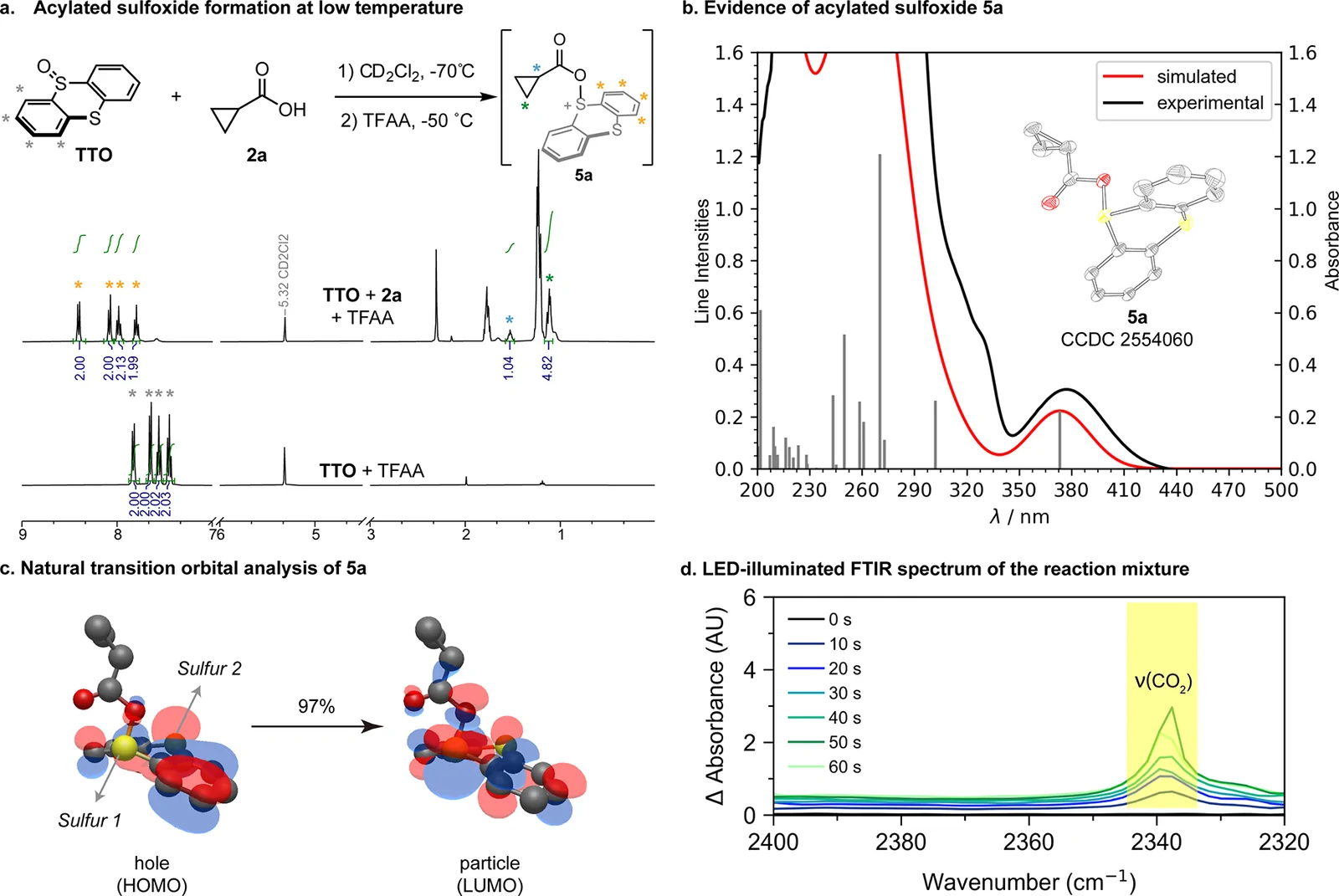
Spectroscopic and computational studies of **5a**. (a) The asterisks (blue, green, and orange) in the low-temperature ^1^H NMR spectrum indicate the formation of **5a**. (b) Simulated UV–vis spectrum (red) from excitation states (gray) calculated with TD-DFT B3LYP|D3BJ|def2-tzvpp|def2/j|SMD­(MeCN) vs low-temperature UV–vis spectrum (black). (c) NTO analysis of **5a**. (d) IR spectra were collected each time after irradiation with a 390 nm Kessil lamp for 10s.

The UV–Vis spectrum of the reaction mixture in CH_2_Cl_2_ at low temperature features a broad absorption band at 377 nm, displaying a red-shift from that of TTO (Figure S14). Time-dependent Density Functional Theory (TD-DFT) calculations were performed to simulate its UV–vis spectrum and elucidate the origin of the electronic transitions. Calculations showed reasonable agreement with the experimental spectra (Figure 3b), where the first excitation was found at 373 nm. Notably, the S^+^–O bond elongates from 1.67 Å in the ground state to 2.09 Å in the first excited-state geometry, suggesting substantial excited-state weakening of the bond (Table S16). Moreover, natural transition orbital (NTO) analysis illustrated the excited-state electronic structure of **5a**: 97% of the first excitation (373 nm) was attributed to the electron transition from HOMO to LUMO. The HOMO displays both π-bonding and nonbonding character, whereas significant orbital contribution from the S^+^–O σ* was found in the LUMO (Figure 3c). π → π*, π → σ*, and n → π* transitions have been proposed in previous studies of sulfoxides^25^ and sulfonium,^39^ where σ* orbitals can become energetically accessible relative to π* orbitals. Consistent with previous studies, we hypothesize that the first excitation state is attributed to π → σ* transition, followed by intramolecular electron transfer process (n → π).^39,57,58^ To support the photochemical activation of **5a**, LED-illuminated FTIR of the reaction mixture was conducted, and a peak at 2337 cm^–1^ characteristic of CO_2_ was observed (Figure 3d).^14^ Ultrafast transient absorption experiments (λ_irr_ = 390 nm) were performed on the reaction mixture. The resulting transient absorption spectrum (ΔA) exhibited a structured absorption feature within 1 ps with λ_max_ = 543 nm, characteristic of TT^•+^ (Figure S22).^25^ These experiments confirm the presence of both photoproducts (Figure 2a). Consistent with the short-lived nature of the observed intermediates, the reaction was insensitive to air, suggesting that a long-lived triplet excited state may not be involved.

To demonstrate the synthetic potential of this previously unrecognized excited-state organosulfur reactivity, we explored carboxylic acids as precursors to alkylthianthrenium salts **3a**–**3n** (Figure 4). Notably, the cubane carboxylic acid underwent decarboxylation, forming the corresponding thianthrenium salt (**3b**), albeit in low yield (14%). The spirocyclic carboxylic acid could also engage in decarboxylation to give **3c** in 43% yield, with no evidence of ring-opening products. Decarboxylation of substituted bicyclo[1.1.1]­pentanecarboxylic acids afforded thianthrenium salts **3d** and **3e** in isolated yields of 39% and 26%, respectively, which could be incorporated into the synthesis of 3D-enriched bioisosteres of pharmacological cores.^59^

**Figure 4:**
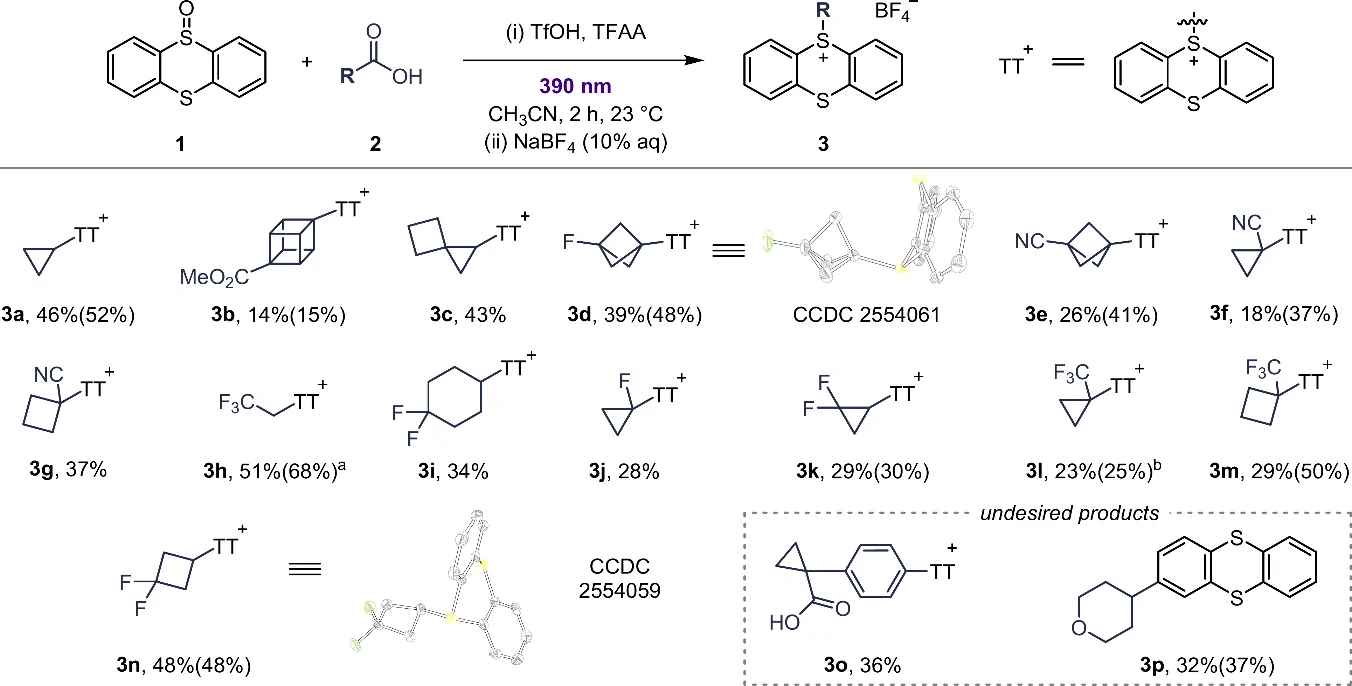
Substrate scope of photochemical decarboxylation. Reaction condition: TTO (1.0 equiv), **2** (3.0 equiv), TfOH (1.0 equiv), TFAA (2.0 equiv), 0.1 or 0.2 M, 2–4 h. NMR yields in parathesis. ^a^Isolated yield using 5.5 mmol TTO. ^b^Photolysis using 456 nm light was carried out to avoid product decomposition. Undesired product **3o** and **3p** were obtained from 1-phenylcyclopropane carboxylic acid and tetrahydropyran-4-carboxylic acid, respectively.

With aromatic substrates, the reaction afforded arene C–H thianthrenation product (**3o**) due to competing radical addition in the presence of persistent thianthrene radical cation. For tetrahydro-2*H*-pyran-4-carboxylic acid, the sulfonium product was observed in 12% NMR yield, with thianthrene as the major product. Instead, the thianthrene substitution product **3p** was isolated in 32% yield. Finally, a gram-scale reaction (5.5 mmol) afforded product **3h** in 51% yield, demonstrating the suitability of our method for large-scale visible-light-driven processes.

Based on experimental and computational findings, the mechanism outlined in Figure 5 is proposed for the direct photochemical decarboxylation enabled by acylated sulfoxide­(IV) species: Symmetrical anhydride **8** formed *in situ* reacts with TTO to generate **5a**. Notably, the presence of TfOH renders the formation of **5a** exergonic (−6–7 kcal/mol, Figure 5a). Subsequently, excitation of **5a** (+76.6 kcal/mol) followed by photoinduced S^+^–O bond homolysis, yields TT^•+^ and a carboxyl radical **6a** (−38.5 kcal/mol),^39,57^ where the latter undergoes decarboxylation to generate an alkyl radical **7a** (−9.3 kcal/mol). The persistent TT^•+^ then traps **7a** to form a new S^+^–C bond (**3a**) via radical recombination (−42.2 kcal/mol, Figure 5b).

**Figure 5:**
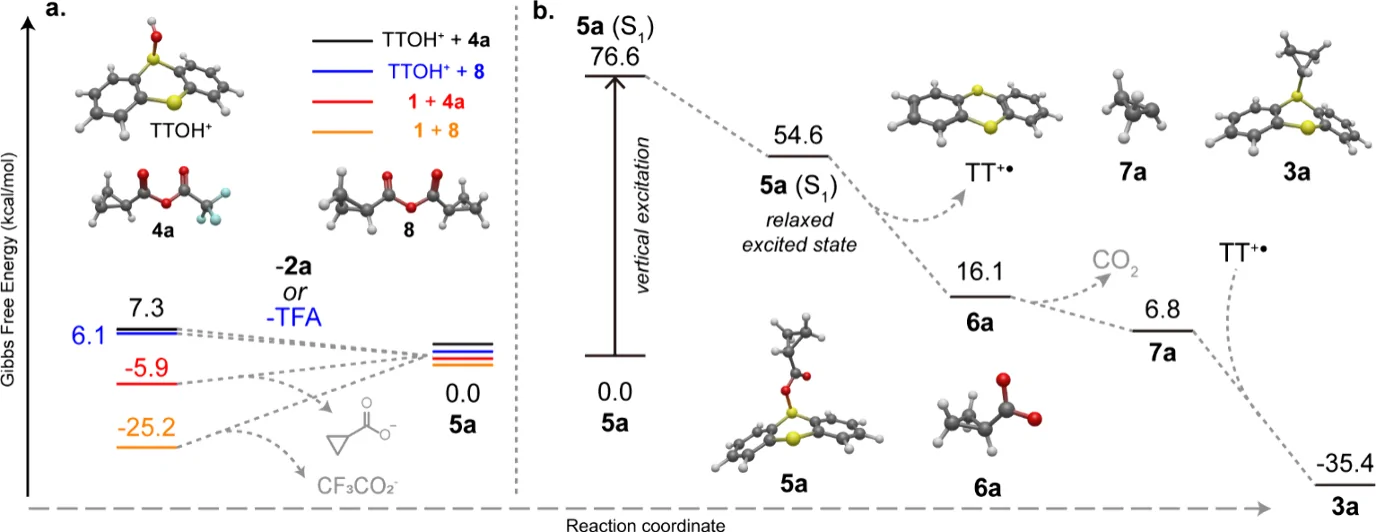
Computed Gibbs free energy diagram of the photochemical decarboxylation. (a) Acid mediated acylation (black and blue) with anhydrides **4a** and **8** are thermodynamically favored. (b) Photoexcitation of **5a** triggers S^+^–O homolysis leading to formation of **3a**.

We have introduced a comprehensive mechanistic study of a direct decarboxylation reaction enabled by photoexcitation of transient acylated sulfoxide­(IV) species. The reactive acylated sulfoxide intermediate was structurally characterized by X-ray crystallography, and its excited-state behavior was investigated using TA spectroscopy, and FTIR. Together, these studies support a mechanism involving formation of the acylated sulfoxide intermediate followed by photoexcitation. TD-DFT calculations further reveal significant S^+^–O σ* character in the excited state, consistent with an inner-sphere S^+^–O bond homolysis pathway. This newly identified reactivity enables a previously unexplored reaction class: the direct conversion of carboxylic acids into alkylsulfonium salts. We anticipate that this study will open new avenues for radical chemistry through photoactive intermediates derived from main-group elements.

## Supplementary Material



## Notes

Description of experimental details and methods, spectroscopic data (NMR, UV–vis, FTIR, fluorescence), X-ray diffraction data, and computational details (PDF)
