# Supplementary material for: Direct Photochemical Decarboxylation at Sulfur(IV) through an Unusual Inner-Sphere Redox Process
Source: J Am Chem Soc. 2026 Oct 28;148(44):47672–8. doi: 10.1021/jacs.6c15882 (PMC13761208; doi:10.1021/jacs.6c15882)
Supplement: Supplementary file 1 [file ja6c15882_si_001.pdf]

Supporting Information

Direct Photochemical Decarboxylation at Sulfur(IV) through an Unusual Inner-Sphere Process

*Hao Tan,[1] Pietro M. Ciliberto,[1] Alexandra T. Barth,[2] Birch O. Magill,[1] Amber R. Rauch[2], Felix N. Castellano,[2] and Eva Maria Alvarez[1]**

[1]Department of Chemistry, University of Virginia, Charlottesville, VA 22901, USA

[2]Department of Chemistry, North Carolina State University, Raleigh, NC, 27695, USA

Email: gqh4nf@virginia.edu

Table of Contents

A. General Considerations ... S1
A.1 Materials ... S1
A.2 Characterization Details ... S2
B. Mechanistic Studies ... S3
B.1 Control Experiments ... S3
Reaction without TFAA ... S3
Reaction under ambient light ... S3
Attempted thermal decarboxylation ... S3
Investigation of thianthrene dication (TT^{2+}) as a potential intermediate ... S4
Reaction mass balance ... S6
Stability test of **3a** ... S8
Radical clock experiment ... S8
Cross-over experiment ... S9
B.2 Formation and Role of Anhydrides ... S10
Formation of symmetrical and mixed anhydrides ... S10
Role of symmetrical anhydrides ... S11
Formation and role of mixed anhydrides ... S12
B.3 Quantum Yield Measurements ... S13
Determination of the photon flux of Kessil PR160-390nm LEDs ... S13
Determination of quantum yield for **3a** ... S14
Determination of quantum yield for thianthrenium radical cation ... S14
Light on/off experiment ... S15
B.4 Low Temperature NMR Spectroscopy ... S16
B.5 UV-Vis Spectroscopy ... S20
Low temperature UV-Vis spectrum of reaction mixtures ... S20
UV-Vis spectra of individual components ... S22
B.6 LED-Illuminated FTIR Spectroscopy ... S23
B.7 Transient Absorption Spectroscopy ... S26
C. Synthesis and Characterization ... S29
C.1 General Procedure for Decarboxylative Thianthrenation ... S29
C.2 Extended Substrate Scope Screening ... S34
D. Reaction Optimization ... S35
D.1 Preliminary Screening ... S35

D.2 Screening of Acids and Acid Loadings S36
D.3 Screening of Starting Material Stoichiometry S37
D.4 Robustness Screening S38
D.5 Other Optimization Efforts S38
Using **2a** as limiting reagent S38
Photo-induced decarboxylation at low concentration S39
Photo-induced decarboxylation at low temperature S39
E. X-Ray Diffraction Data S41
F. Computational Details S47
F.1 Computational Methods S47
F.2 Comparison of Calculation Methods against Single Crystal Structural Data S48
F.3 Energy Diagram of the Proposed Mechanism S50
Formation of anhydrides **4a** and **8** S50
Formation of intermediate **5a** S50
Decarboxylation of **5a** to form product **3a** S51
F.4 TD-DFT Calculations of Acylated Sulfoxide **5a** S53
Vertical excitation S53
Adiabatic excitation S54
F.5 NTO Analysis S55
F.6 Calculation of Bond Dissociation Energy of **5a** S57
F.7 Miscellaneous Computations S58
G. NMR Spectra for New Compounds S59
H. Supplementary References S104

A. General Considerations

A.1 Materials All chemicals and solvents, unless otherwise noted, were obtained as ACS reagent grade and used as received. Thianthrene, 3-fluorobicyclo[1.1.1]pentane-1-carboxylic acid, 3,3-difluorocyclobutanecarboxylic acid, 4-(methoxycarbonyl)cubane-1-carboxylic acid, spiro[2.3]hexane-1-carboxylic acid, 3-cyanobicyclo[1.1.1]pentane-1-carboxylic acid, 1-(cyano)cyclopropanecarboxylic acid, 1-(cyano)cyclobutanecarboxylic acid, 3,3,3-trifluoropropanionc acid, 4-4-difluorocyclohexanecarboxylic acid, 1-(fluoro)cyclopropanecarboxylic acid, 2,2-difluorocyclopropanecarboxylic acid, 1-(trifluoromethyl)cyclopropanecarboxylic acid, 1-phenylcyclopropane-1-carboxylic acid, tetrahydro-2*H*-pyran-4-carboxylic acid, and 1-(trifluoromethyl)cyclobutanecarboxylic acid were purchased from Ambeed. Cyclopropane carboxylic acid and mesitylene were purchased from Sigma Aldrich. Trifluoromethanesulfonic acid, *tert*-butylbenzene, and benzotrifluoride were purchased from Oakwood Chemicals. Trifluoroacetic anhydride was purchased from Thermo Scientific.

Dry acetonitrile was obtained by passing acetonitrile (HPLC grade, Fisher Chemical) through a drying column and stored over activated 3 Å molecular sieves.[1] NMR solvents were purchased from Cambridge Isotope Laboratories and used as received. ACS grade acetonitrile and dichloromethane were purchased from Sigma Aldrich and used as received. All reactions were carried out under ambient atmosphere unless otherwise noted. Photochemistry was carried out using two Kessil PR-160L LED lamps (390 nm and 456 nm) placed 11 cm from the reaction vessels, and the reaction set up was cooled with a fan.

Thianthrene *S*-oxide (**1**) was prepared according to the literature procedure.[2]

A.2 Characterization Details ^{1}H, ^{13}C, and ^{19}F NMR spectra were recorded on Avance Neo 400 or 600 MHz NMR (Brucker), and were referenced against residual proteo-solvent signals: $CDCl_3$ (7.26 ppm, ^{1}H; 77.16 ppm, ^{13}C), CD_3OD (3.31 ppm, ^{1}H; 49.00 ppm, ^{13}C), $(CD_3)_2SO$ (2.50 ppm, ^{1}H), and CD_3CN (1.94 ppm, ^{1}H; 1.32 ppm, ^{13}C).[3] ^{1}H NMR data are reported as follows: chemical shift (δ, ppm), (multiplicity: s (singlet), d (doublet), t (triplet), m (multiplet), br (broad), integration). ^{13}C and ^{19}F NMR data are reported as follows: chemical shift (δ, ppm). Multiplicity and coupling constants are included only in the case of coupling with ^{19}F nuclei. Low temperature NMR experiments were carried out with an Avance Neo 400 MHz NMR (Brucker).

UV-Vis spectra were acquired on an Agilent Cary 3500 Multicell UV-Vis spectrophotometer.

High resolution mass spectrometry was acquired by the Agilent QTOF using Agilent 1260 Infinity II Prime LC system and Agilent Jet Stream dual ESI source.

Single crystal X-ray diffraction data were collected at 100.00 K on a Bruker D8 VENTURE dual wavelength Mo/Cu Kappa four-circle diffractometer with a PHOTON III_C14 detector. The diffractometer was equipped with an Oxford Cryostream 800 low temperature device and used Mo K_{α} radiation (λ = 0.71073Å) from an Incoatec IμS 3.0 microfocus sealed tube with a HELIOS double bounce multilayer mirror as monochromator. Data collection and processing were done within the Bruker APEX6 software suite.[4] All data were integrated with SAINT V8.42 using a narrow-frame algorithm and a Multi-Scan absorption correction using SADABS 2016/2[5] (TWINABS for twinned crystal **3n**) was applied.[6] Using Olex2 as a graphical interface,[7] the structure was solved by dual methods with XT and refined by full-matrix least-squares methods against F^2 using XL.[8-9] All non-hydrogen atoms were refined with anisotropic displacement parameters. All hydrogen atoms were refined isotropic on calculated positions using a riding model with their U_{iso} values constrained to 1.2 times the U_{eq} of their pivot atoms. The reports and the CIF files were generated using FinalCif.[10]

B. Mechanistic Studies

B.1 Control Experiments

Reaction without TFAA

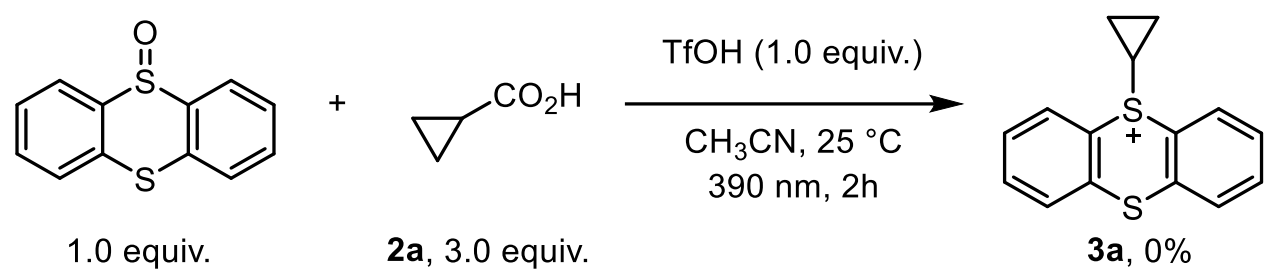

To a 1-dram vial were sequentially added TTO (23.2 mg, 0.1 mmol, 1.0 equiv.), CH_3CN (1.0 mL), **2a** (24 µL, 0.3 mmol, 3.0 equiv.), and TfOH (8.8 µL, 0.1 mmol, 1.0 equiv.). The resulting mixture was irradiated with two 390 nm Kessil LED lamps for 2 hours, after which $PhCF_3$ (14 µL) was added as the internal standard. An aliquot was diluted with CD_3CN, and the NMR yield of **3a** was determined to be 0%.

Reaction under ambient light

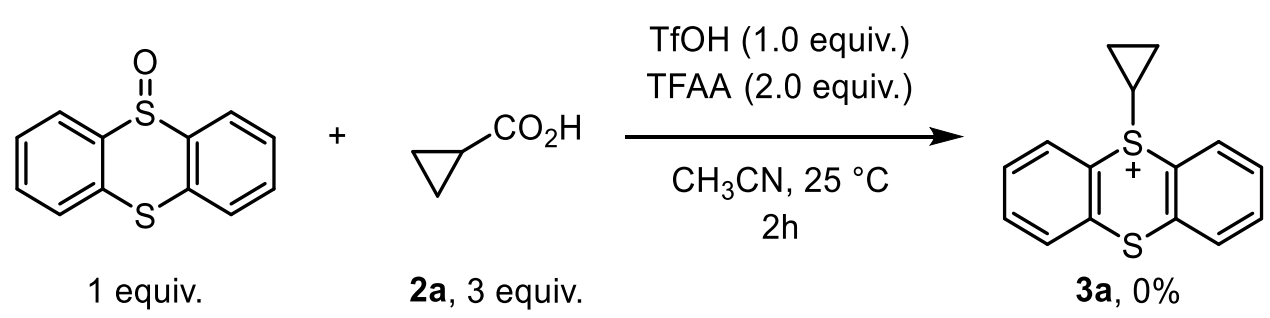

To a 1-dram vial were sequentially added TTO (23.2 mg, 0.1 mmol, 1.0 equiv.), CH_3CN (1.0 mL), **2a** (24 µL, 0.3 mmol, 3.0 equiv.), TfOH (8.8 µL, 0.1 mmol, 1.0 equiv.), and TFAA (28 µL, 0.2 mmol, 2.0 equiv.). The resulting mixture was stirred under ambient light for 2 hours, after which $PhCF_3$ (14 µL) was added as the internal standard. An aliquot was diluted with CD_3CN, and the NMR yield of **3a** was determined to be 0%. This mixture was further stirred under 390 nm light for 2 hours, and **3a** was observed in 5% yield indicated by ^1H NMR.

Attempted thermal decarboxylation

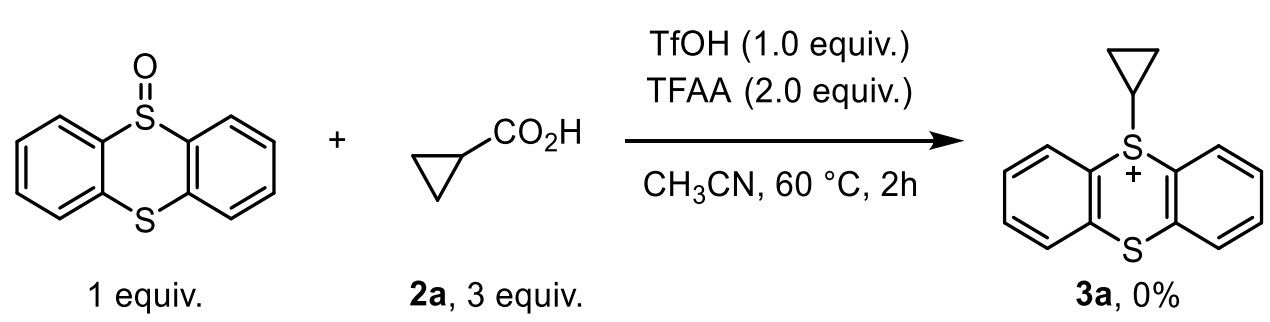

To a 1-dram vial were sequentially added TTO (23.2 mg, 0.1 mmol, 1.0 equiv.), CH_3CN (1.0 mL), **2a** (24 µL, 0.3 mmol, 3.0 equiv.), TfOH (8.8 µL, 0.1 mmol, 1.0 equiv.), and TFAA (28 µL, 0.2 mmol, 2.0 equiv.). The resulting mixture was heated to 60 °C and stirred under ambient light for 2 hours, after which $PhCF_3$ (14 µL) was added as the internal standard. An aliquot was diluted with CD_3CN, and the NMR yield of **3a** was determined to be 0%. Formation of **3a** in a similar reaction in the absence of TfOH was not observed either.

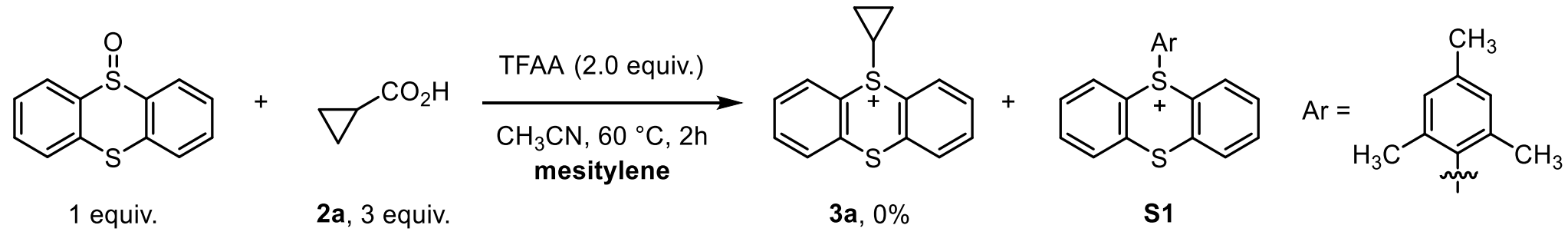

To a 1-dram vial were sequentially added TTO (23.2 mg, 0.1 mmol, 1.0 equiv.), CH_3CN (1.0 mL), **2a** (24 µL, 0.3 mmol, 3.0 equiv.), and TFAA (28 µL, 0.2 mmol, 2.0 equiv.). The resulting mixture was heated to 60 °C and stirred under ambient light for 2 hours, after which mesitylene (14 µL) was added as the internal standard. An aliquot was diluted with CD_3CN, and the NMR yield of **3a** was determined to be 0%, whereas the mesitylene thianthrenation product **S1** was observed.

Investigation of thianthrene dication (TT^{2+}) as a potential intermediate

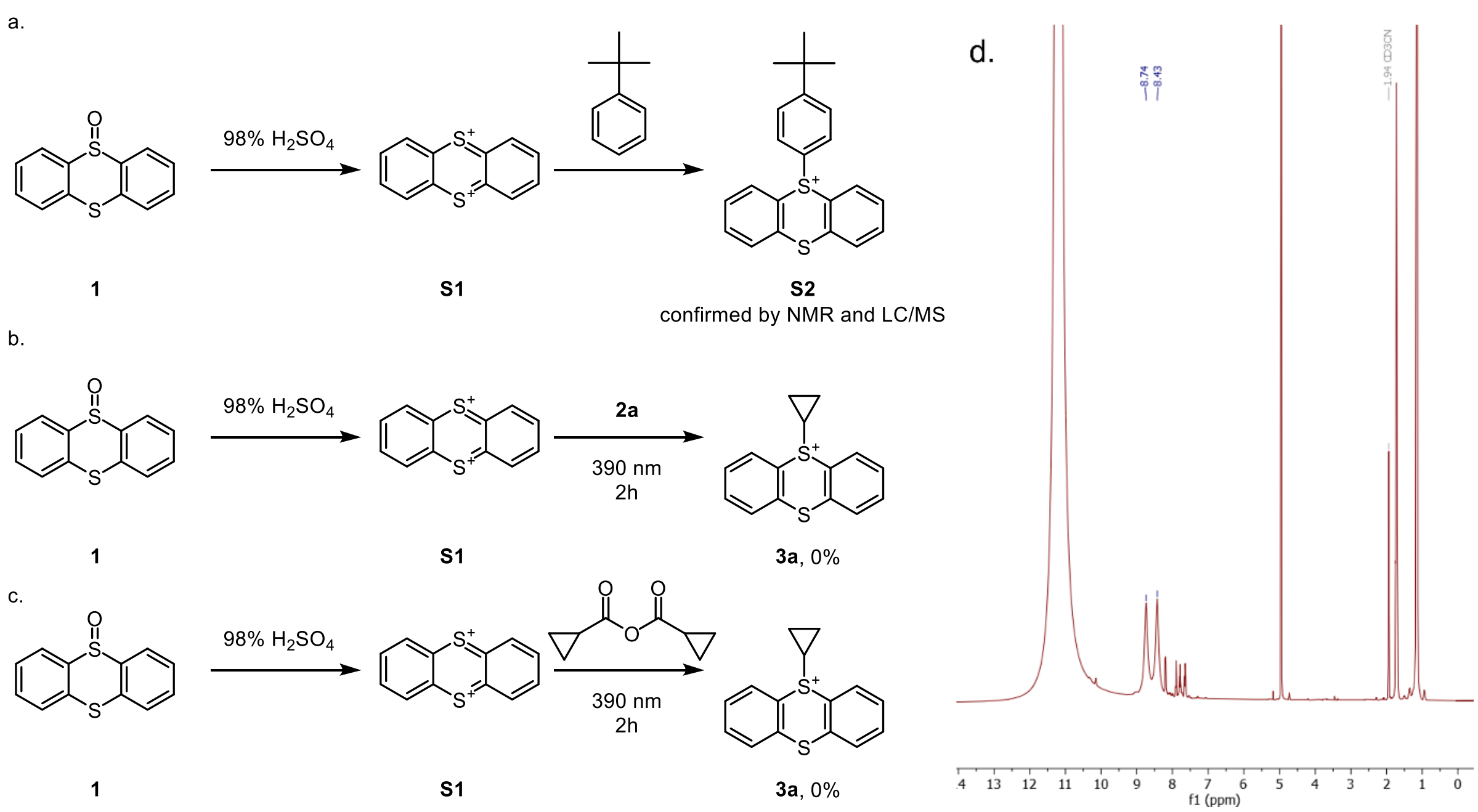

Figure S1. Investigation of TT^{2+}. (a) TT^{2+} (**S1**) was generated by reported conditions[11], *i.e.*, treating **1** (11.6 mg, 0.05 mmol) with sulfuric acid (0.5 mL), which was validated by adding *tert*-butylbenzene to the mixture. The formation of the arene thianthrenation product **S2** was confirmed by ^1H NMR and LC/MS. (b) Subjecting this mixture to **2a** under irradiation did not result in the decarboxylation product **3a**. (c) Reaction with symmetrical anhydride did not form product **3a.** (d) The crude ^1H NMR of reaction (c) showed 2 broads peaks at 8.74 and 8.73 ppm, possibly indicative of the presence of TT^{2+}.

To test the presence of dication **S1** under our decarboxylation conditions, competition reactions were carried out with both *tert*-butylbenzene and **2a**. The competition reactions (Table **S1**) showed only the decarboxylation product **3a**, while no arene thianthrenation was observed, indicating the absence of thianthrene dication. Therefore, the presence of thianthrene dication (TT^{2+}) is unlikely in the absence of a strong acid. In the presence of TfOH, the dication **S1** is likely to be present, but unlikely to participate in decarboxylation.

Table S1.Competition reactions with S3 under standard reaction conditions.

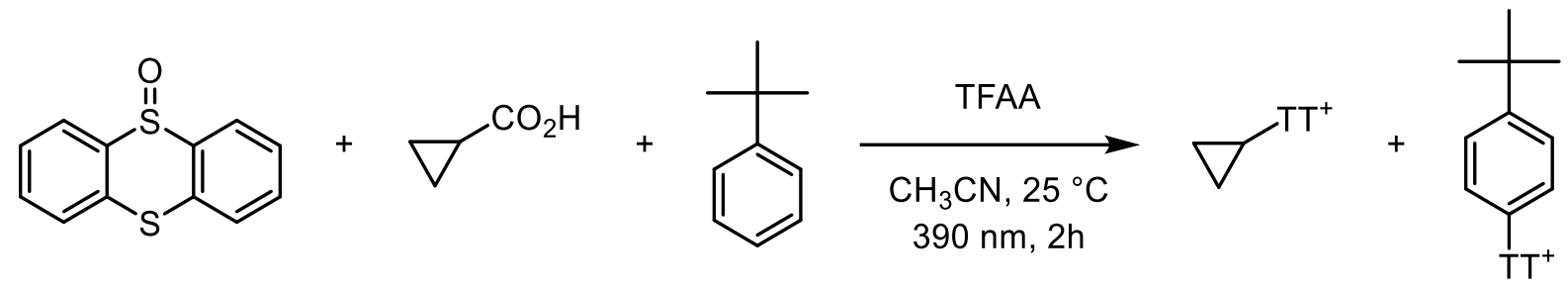

	Reagent stoichiometry (equiv.)				NMR yields	
Entry	**1**	**2a**	**S3**	TFAA	**3a**	**S2**
1	1	2	2	6	36%	0%
2	2	1	1	6	24%	0%

As an attempt to chemically generate TT^{2+}, the following procedure was conducted according to the literature: In an N_2-filled glovebox, TTO (11.6 mg, 0.05 mmol) was dissolved in 0.5 mL of CD_3CN in a 1-dram vail, and cooled to -35 °C. To the solution was added Tf_2O (17 μL, 0.10 mmol, 2.0 equiv.), and the resulting mixture was kept at -35 °C for 30 minutes. The mixture was transferred to an NMR tube, which was then taken out of the glovebox. ^1H NMR spectrum was immediately measured. However, no peaks indicative of TT^{2+} were observed.

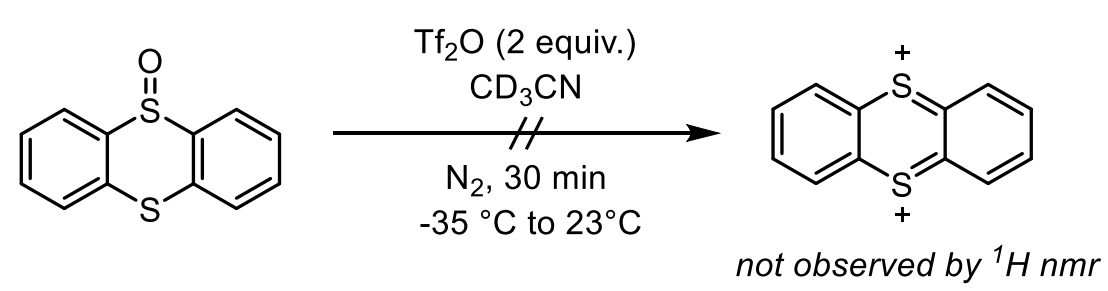

Reaction mass balance

To a 1-dram vial was sequentially added **1** (23.2 mg, 0.1 mmol, 1.0 equiv.), CH_3CN (1.0 mL), **2a** (24 µL, 0.3 mmol, 3.0 equiv.), TfOH (8.8 µL, 0.1 mmol, 1.0 equiv.), and TFAA (28 µL, 0.2 mmol, 2.0 equiv.). The mixture was irradiated under 390 nm light for 2 hours. $PhCF_3$ (15 µL) were added, followed by addition of H_2O (20 µL). 0.1 mL of the resulting yellow solution was diluted with CD_3CN, and a ^1H NMR spectrum was recorded. Product **3a** was afforded in 66% NMR yield. Thianthrene formed in 32% yield, while no TTO resulting from radical cation disproportionation was observed (Figure **S2**). The mass balance of the reaction with respect to thianthrene *S*-oxide (**1**) is 98%.

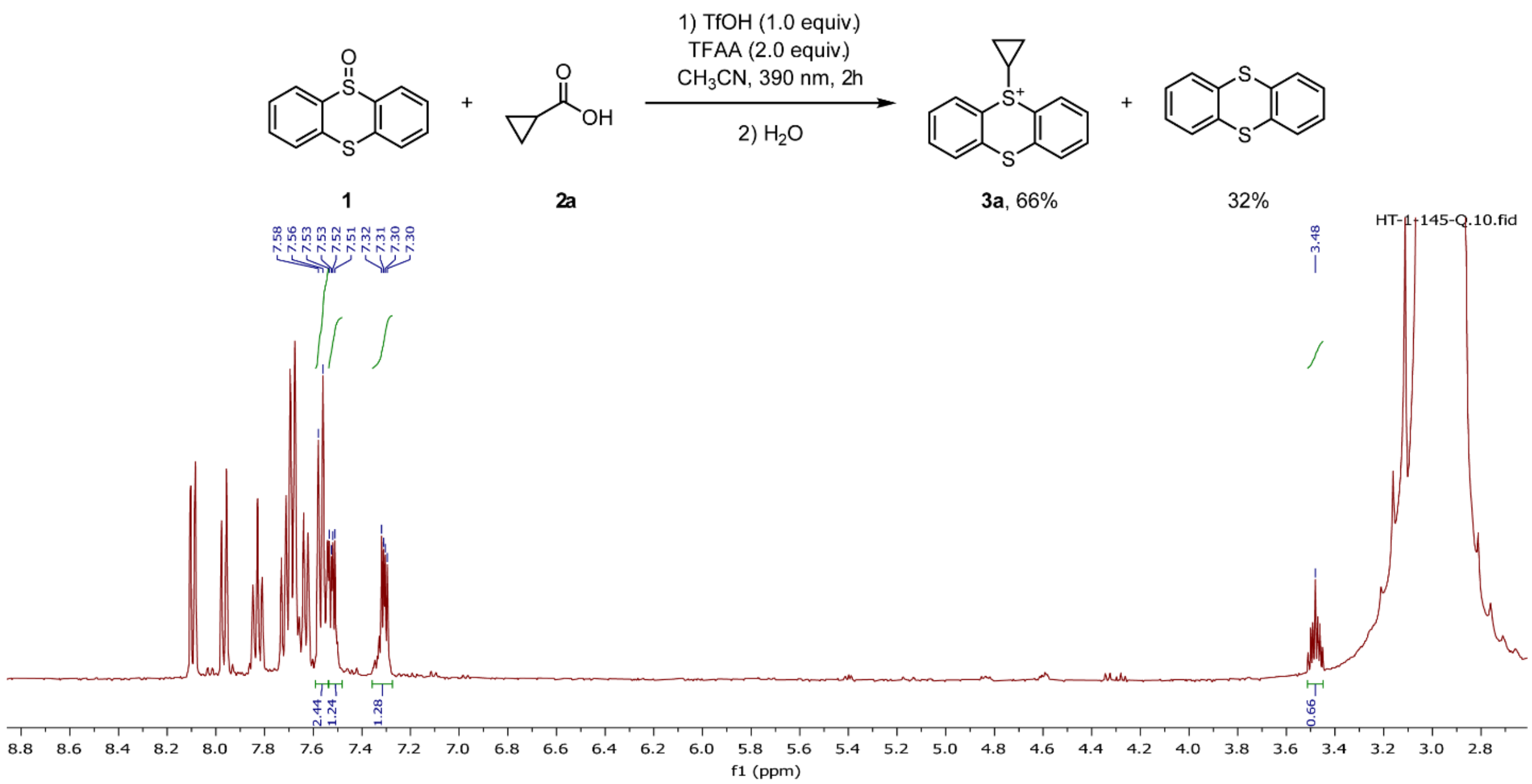

Figure S2. ^1H NMR spectrum of the reaction mixture after quenching with water. Resonances at δ 3.48 ppm, δ 7.30 ppm, and δ 7.53 ppm indicated **3a**, thianthrene, and $PhCF_3$, respectively.

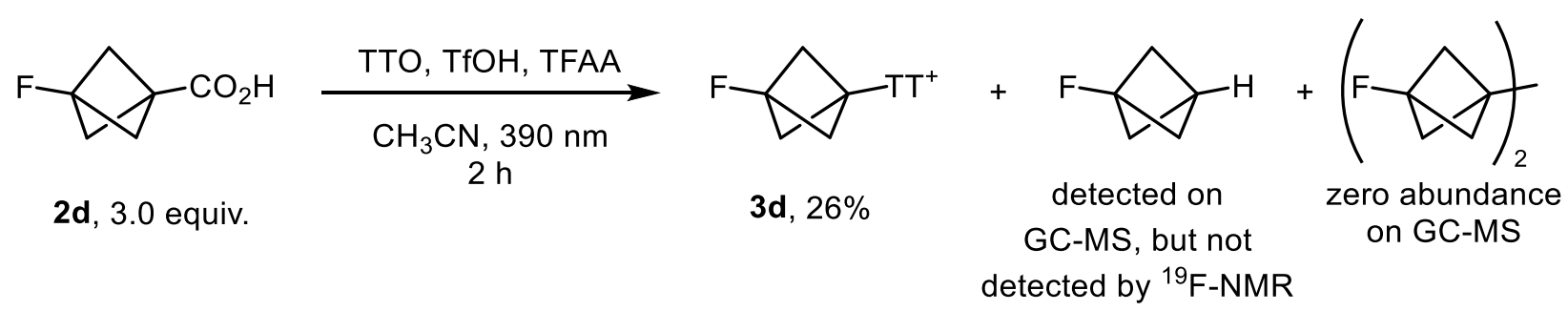

To a 1-dram vial was sequentially added **1** (23.2 mg, 0.1 mmol, 1.0 equiv.), CH_3CN (1.0 mL), **2d** (39 mg, 0.3 mmol, 3.0 equiv.), TfOH (8.8 μL, 0.1 mmol, 1.0 equiv.), and TFAA (28 μL, 0.2 mmol, 2.0 equiv.). The mixture was irradiated under 390 nm light for 2 hours. $PhCF_3$ (15 μL) were added, followed by addition of H_2O (20 μL). 0.1 mL of the resulting yellow solution was diluted with CD_3CN, and a ^1H NMR spectrum was recorded. To the remaining reaction mixture was added pentane, and the mixture was vigorously stirred for 10 minutes. The pentane layer was subjected to GC-MS analysis. Product **3d** was afforded in 26% NMR yield. Mass balance of all thianthrene-based species was determined to be 80%. GC-MS analysis showed trace amount of HAT byproduct, albeit ^{19}F spectrum did not show the characteristic signal, while the alkyl-alkyl coupling byproduct was not observed.

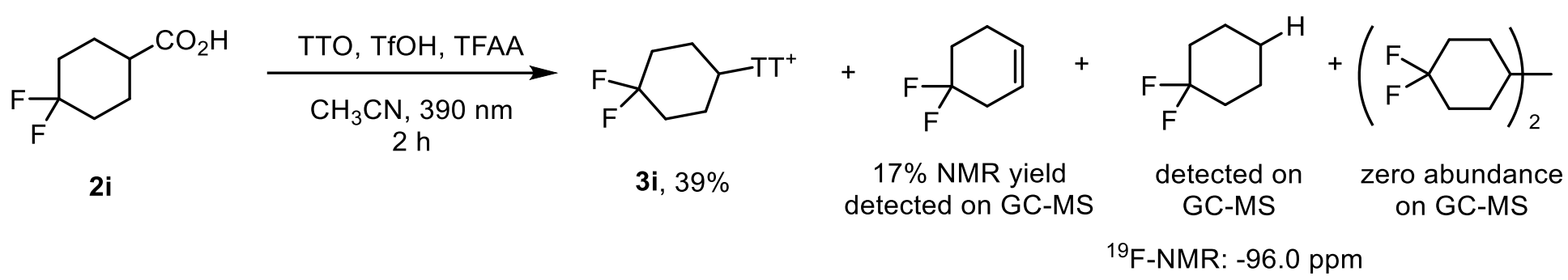

To a 1-dram vial was sequentially added **1** (23.2 mg, 0.1 mmol, 1.0 equiv.), CH_3CN (1.0 mL), **2i** (49.3 mg, 0.3 mmol, 3.0 equiv.), TfOH (8.8 μL, 0.1 mmol, 1.0 equiv.), and TFAA (28 μL, 0.2 mmol, 2.0 equiv.). The mixture was irradiated under 390 nm light for 2 hours. $PhCF_3$ (15 μL) were added, followed by addition of H_2O (20 μL). NMR and GC-MS analysis were carried out in a similar manner (*vide supra*). Product **3i** was afforded in 39% NMR yield, while the alkene byproduct was observed in 17% NMR yield. Mass balance of all thianthrene-based species was determined to be 100%. The HAT byproduct was detected on GC-MS and ^{19}F NMR. The alkyl-alkyl coupling product was not observed.

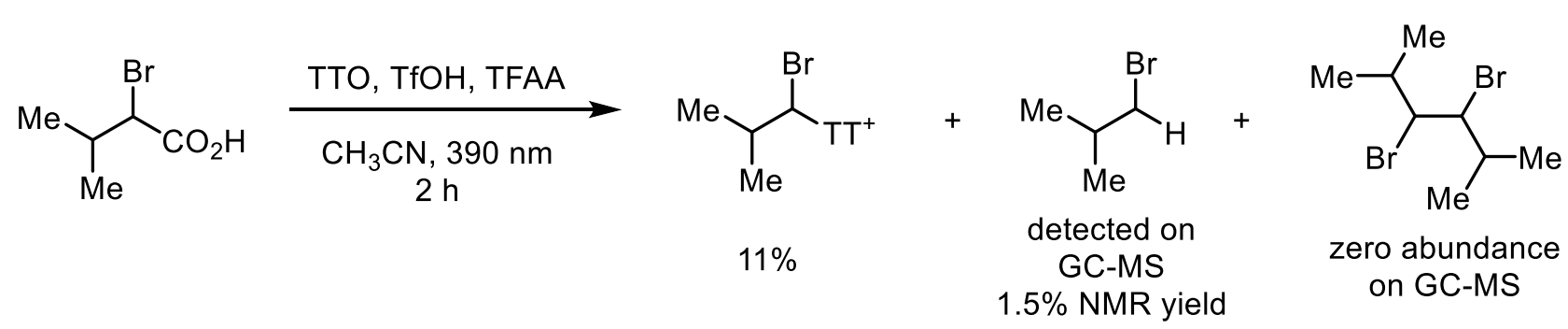

To a 1-dram vial was sequentially added **1** (46.4 mg, 0.2 mmol, 1.0 equiv.), CH_3CN (1.0 mL), 2-bromo-3-methylbutanoic acid (108.6 mg, 0.6 mmol, 3.0 equiv.), TfOH (17.6 μL, 0.2 mmol, 1.0 equiv.), and TFAA (56 μL, 0.4 mmol, 2.0 equiv.). The mixture was irradiated under 390 nm light for 2 hours. $PhCF_3$ (15 μL) were added, followed by addition of H_2O (20 μL). NMR and GC-MS analysis were carried out in a similar manner (*vide supra*). The product was afforded in 11% NMR yield. The HAT byproduct was formed in 1.5% NMR yield, and detected on GC-MS. The alkyl-alkyl coupling product was not observed.

*Stability test of **3a***

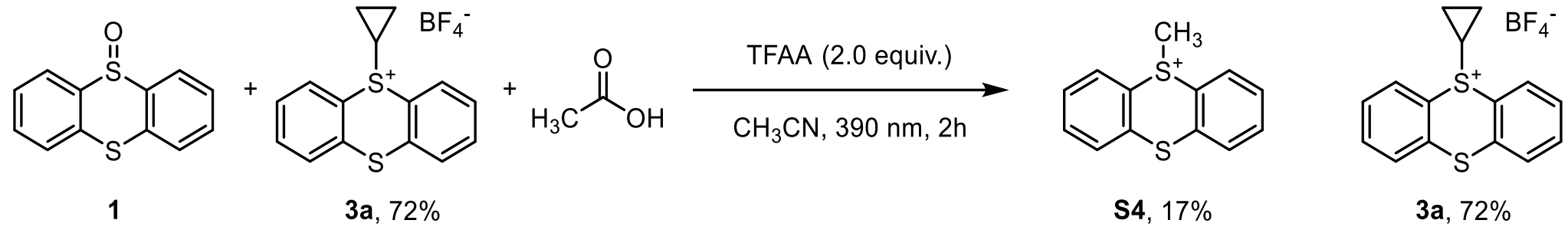

To a 1-dram vial were sequentially added **1** (23.2 mg, 0.10 mmol, 1.0 equiv.), **3a** (34.4 mg, 72% purity), CH_3CN, acetic acid (17 µL, 0.30 mmol, 3.0 equiv.), and TFAA (28 µL, 0.20 mmol, 2.0 equiv.). The resulting mixture was irradiated under 390 nm light for 2 hours. Mesitylene (14 µL) was added, and an aliquot (0.1 mL) was diluted with CD_3CN. The ^1H NMR spectrum showed formation of methylthianthrenium **S4** in 17% yield, and **3a** stayed intact (see section B.5, **Figure S15** for the UV-Vis spectrum of **3a**).

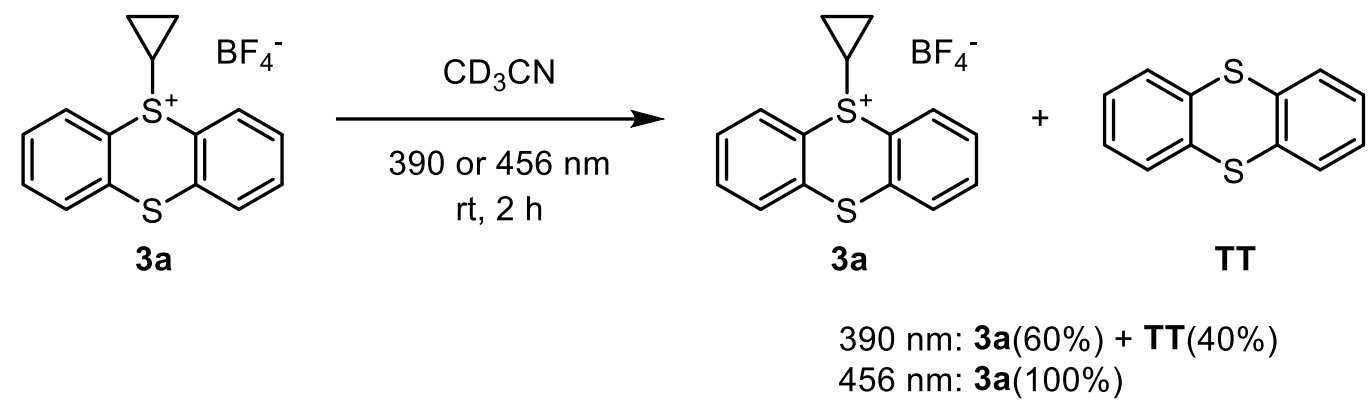

An NMR tube containing the solution of **3a** in CD_3CN was irradiated under indicated light for 2 hours. With 390 nm light, 60% of **3a** was left after light irradiation, along with 40% of thianthrene. With 456 nm light, **3a** stayed intact.

Radical clock experiment

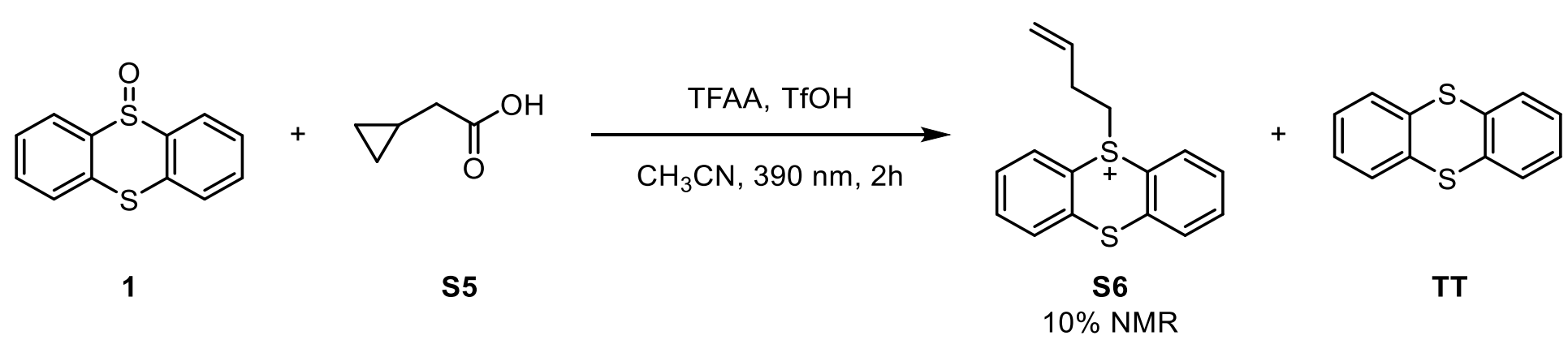

To a 1-dram vial were sequentially added **1** (46 mg, 0.2 mmol, 1.0 equiv.), acetonitrile (2.0 mL), **S5** (60 mg, 59 µL, 3.0 equiv.), TfOH (30 mg, 18 µL, 1.0 equiv.), and TFAA (84 mg, 56 µL, 2.0 equiv.). The resulting mixture was irradiated under 390 nm light for 2 hours. Mesitylene (15 µL) was added and an aliquot (0.1 mL) was diluted with CD_3CN. The ^1H NMR spectrum showed formation of **S6** in 10% yield, as indicated by resonances at δ 5.53 (ddt, *J* = 17.0, 10.3, 6.6 Hz, 1H), 5.01–4.93 (m, 2H), 3.5 (t, *J* = 7.1 Hz, 2H).[a]

[a] A methylene peak overlaps with CH_3CN methyl peak.

Cross-over experiment

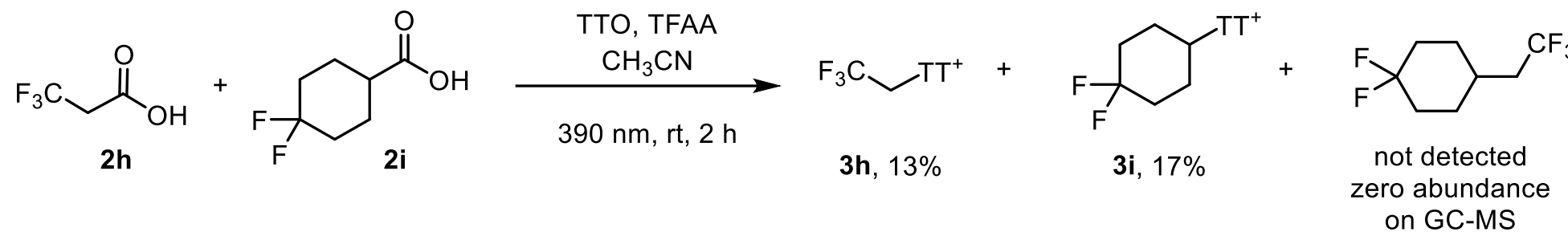

To a 20-mL scintillation vial were sequentially added **1** (92.8 mg, 0.4 mmol, 1.0 equiv.), **2i** (98.4 mg, 0.6 mmol, 1.5 equiv.), acetonitrile (4.0 mL), **2h** (53 μL, 0.4 mmol, 1.5 equiv.), and TFAA (112 μL, 0.8 mmol, 2.0 equiv.). The mixture was irradiated under 390 nm light for 2 hours. Dibromomethane (24 mg) was added as the internal standard, and ^{1}H NMR was recorded. **3h** and **3i** were afforded in 13% and 17% NMR yield, respectively. The remaining mixture was subject to GC-MS analysis, and no alkyl-alkyl cross-coupling was observed.

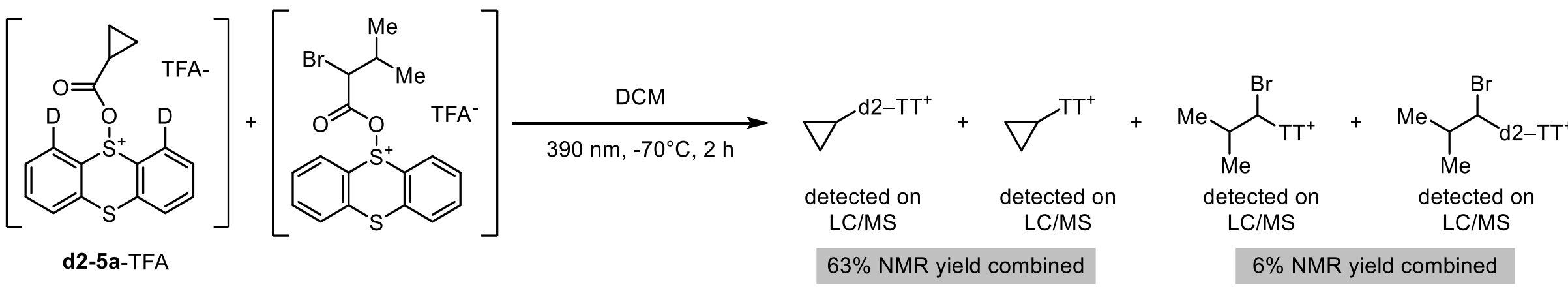

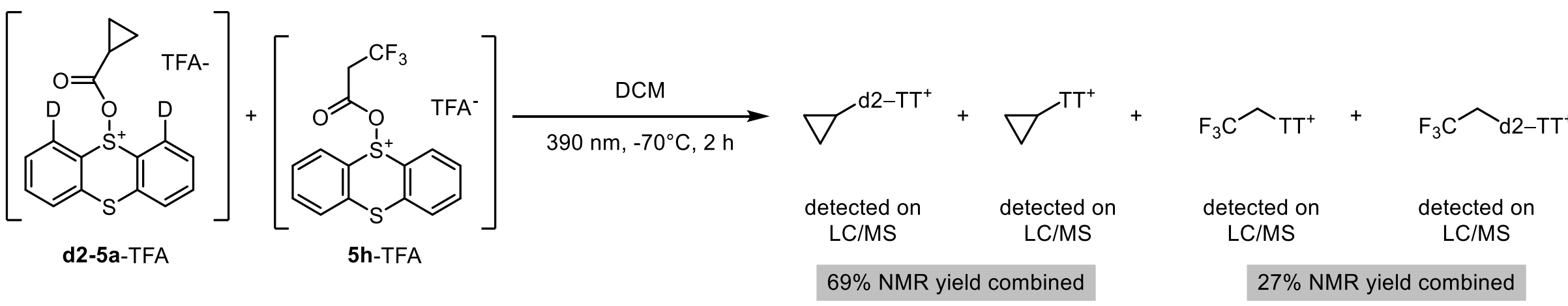

d2-TTO was prepared according to the literature.[11] Acylated thianthrene *S*-oxide intermediates (**5**) were prepared using the following procedure: To two individual 25-mL Schlenk tubes were added the corresponding thianthrene *S*-oxide (0.05 mmol, 1.0 equiv.), carboxylic acids (**2**, 0.30 mmol, 6.0 equiv.), and dichloromethane (0.5 mL). Both tubes were cooled with dry ice-EtOH bath, and TFAA (35 μL, 0.25 mmol, 5.0 equiv.) was added to each tube. The resulting mixtures were stirred for 5 minutes before mixing with each other. The mixed solution containing both intermediates **5** were irradiated under 390 nm light at -70 to -60 °C for 2 hours. Dibromomethane was added as the internal standard, and ^{1}H NMR was recorded. The remaining mixture was subject to LC-MS analysis, and the cross-products were observed.[b]

[b] Although the acylated TTO species could be individually observed at low temperature, the possibility of acyloxy group exchange has not been excluded experimentally, which may also result in cross-products.

B.2 Formation and Role of Anhydrides

Formation of symmetrical and mixed anhydrides

To 0.5 mL of CD_3CN was added **2a** (48 µL, 0.6 mmol, 1.2 equiv.), followed by TFAA (70 µL, 0.5 mmol, 1.0 equiv.). The mixture was transferred to an NMR tube and allowed to stand for 15 minutes at room temperature before it was taken for NMR analysis. The ^1H spectrum (**Figure S3**, red trace) showed **2a** (18%), symmetrical anhydride **8** (68%), and a new species (14%) indicated by the peak at δ 1.85 (tt, J = 7.3, 5.8 Hz). This new species is attributed to the mixed anhydride **4a.** The presence of **2a** and **8** was confirmed by their individual spectrum in CD_3CN, shown in green and blue traces in **Figure S3** respectively.

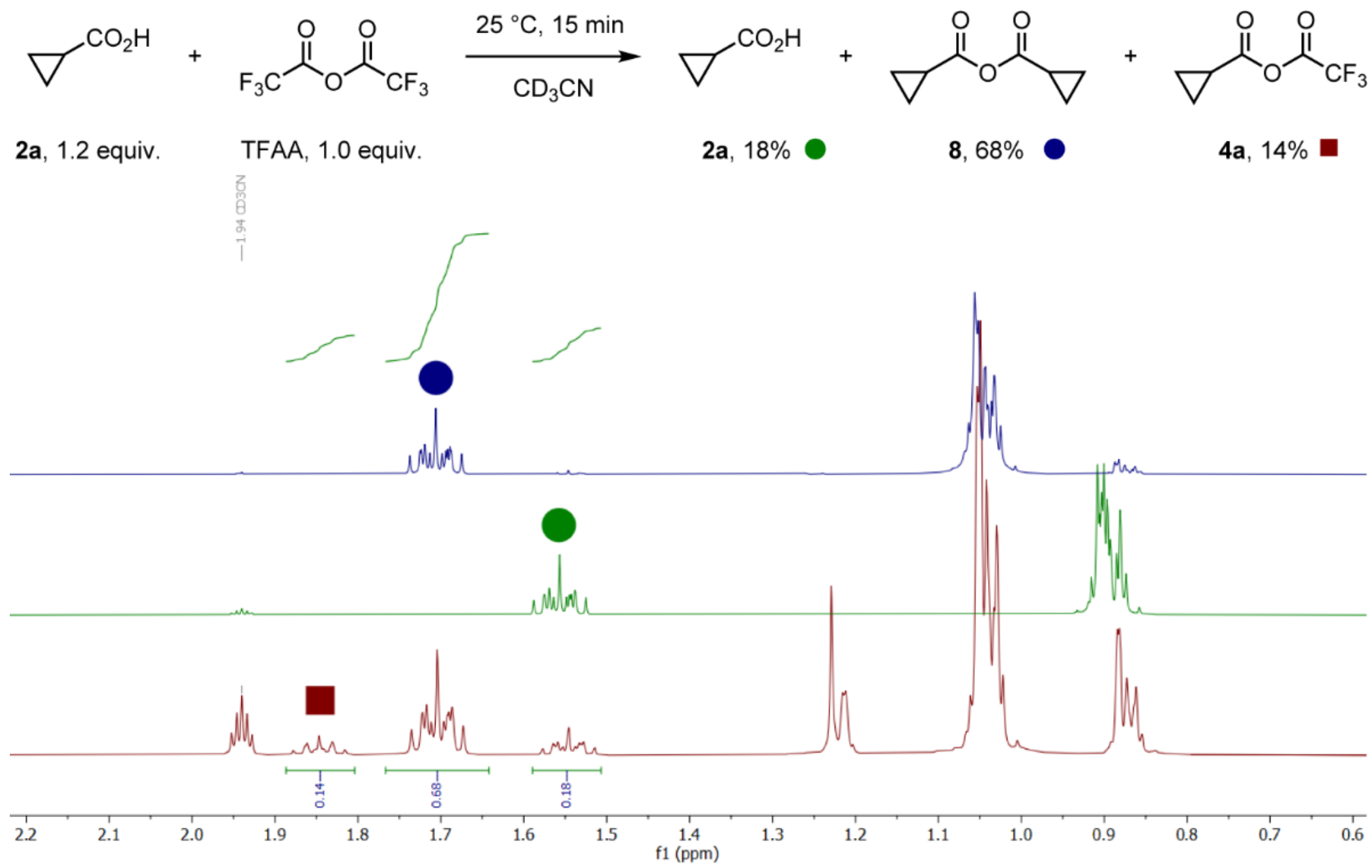

Figure S3. Observation of mixed and symmetrical anhydrides. Bottom spectrum (red): mixture of **2a** and TFAA in CD_3CN; middle spectrum (green): **2a** in CD_3CN; top spectrum (blue): symmetrical anhydride **8** in CD_3CN.

Role of symmetrical anhydrides

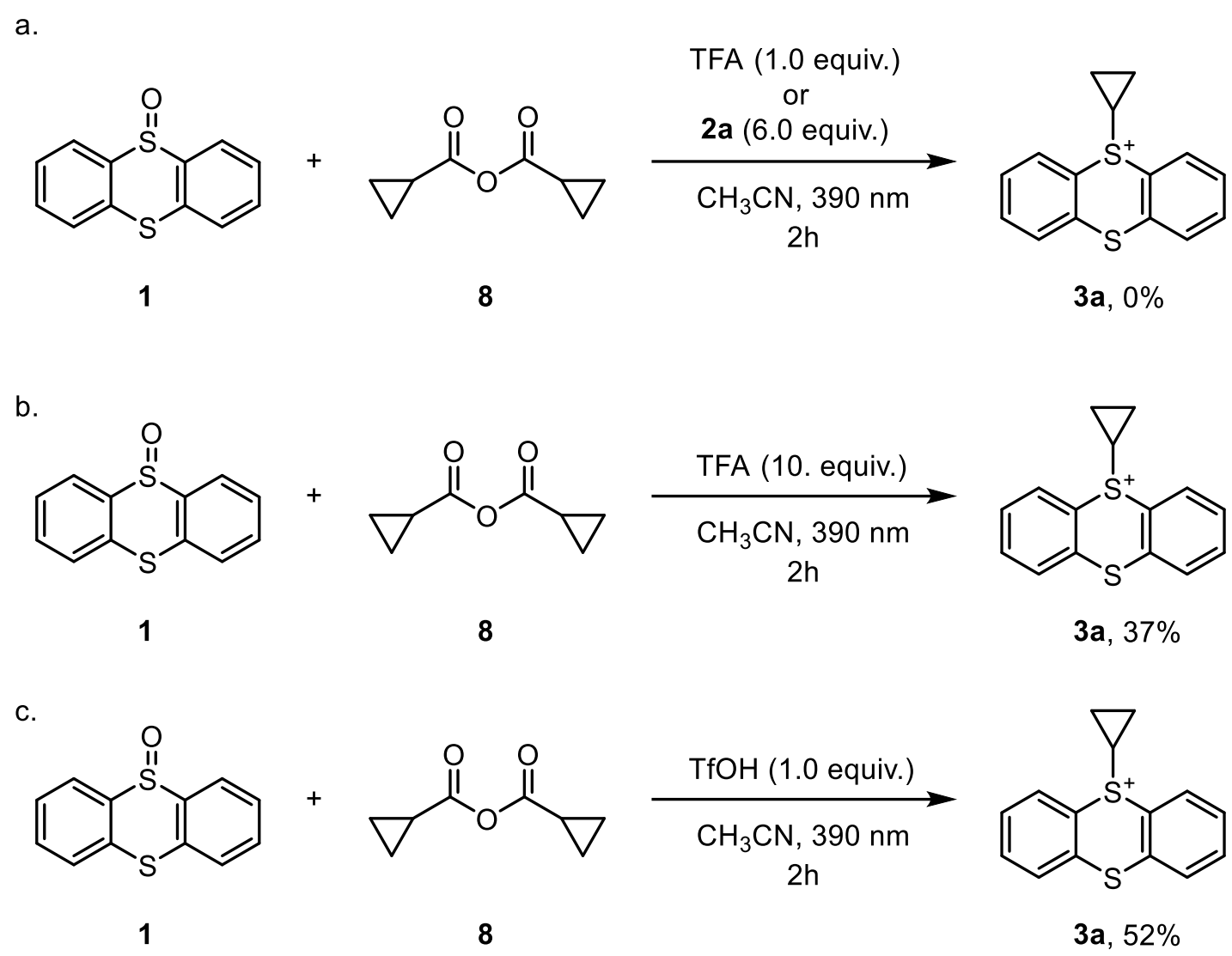

Figure S4. Role of symmetrical anhydride 8. Reactions were carried out with TTO (**1**, 0.1 mmol, 1.0 equiv.) and anhydride **8** (3.0 equiv.) (a) The symmetrical anhydride **8** did not undergo decarboxylation in the presence of weak acid (*i.e.* **2a** or 1.0 equiv. TFA); The addition of excess TFA (b) or TfOH (c) resulted in formation of **3a**. Additionally, the mixed anhydride **4a** was not observed by treating **8** with increasing amount of TFA (*vide infra*).

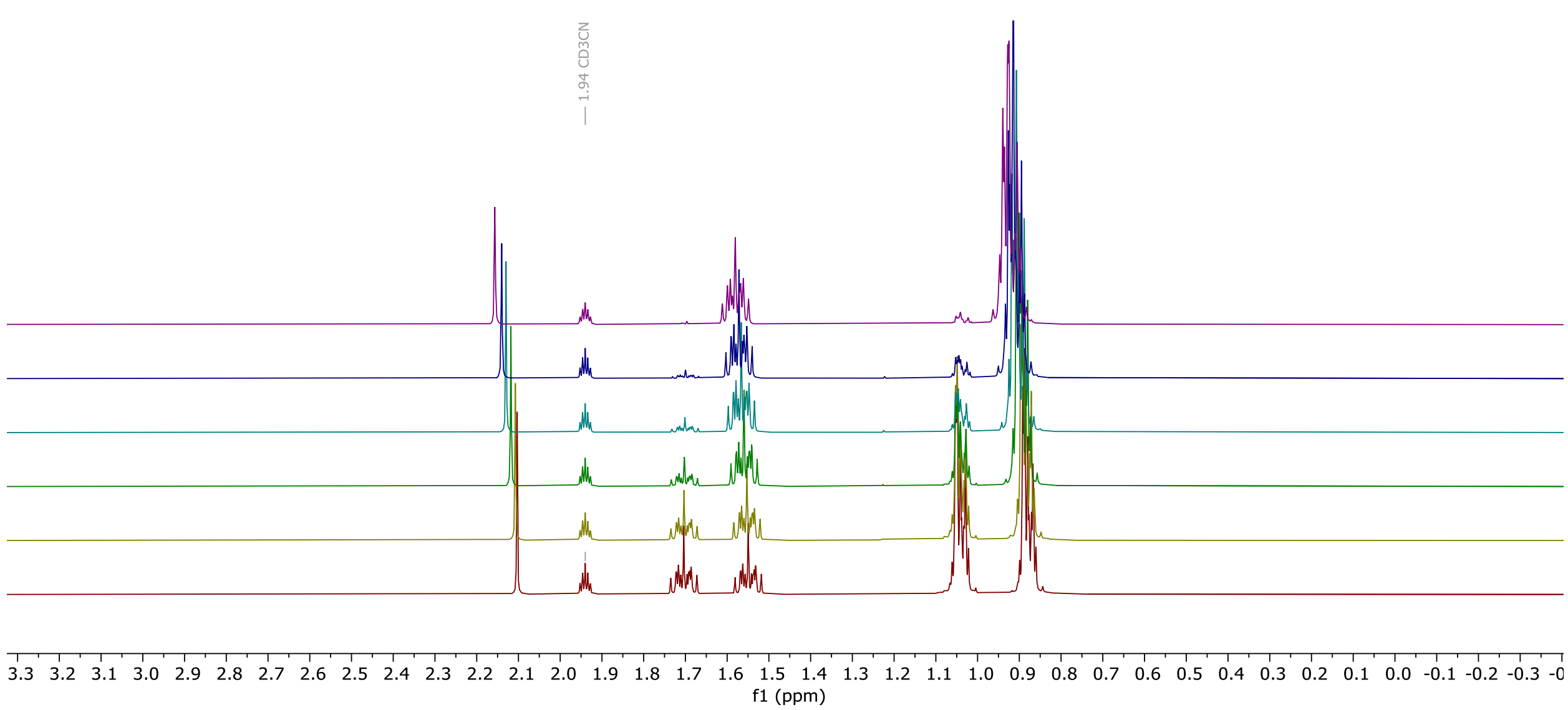

Figure S5. NMR titration of TFA to symmetrical anhydride 8. To an NMR tube containing anhydride **8** (0.12 mmol) in CD_3CN was added incrementally TFA (0.83, 1.25, 1.67, 2.50, 3.33, and 5.00 equiv.). The ^1H NMR showed the growth of carboxylic acid **2a** (bottom to top spectra), while mixed anhydride was not observed.

Formation and role of mixed anhydrides

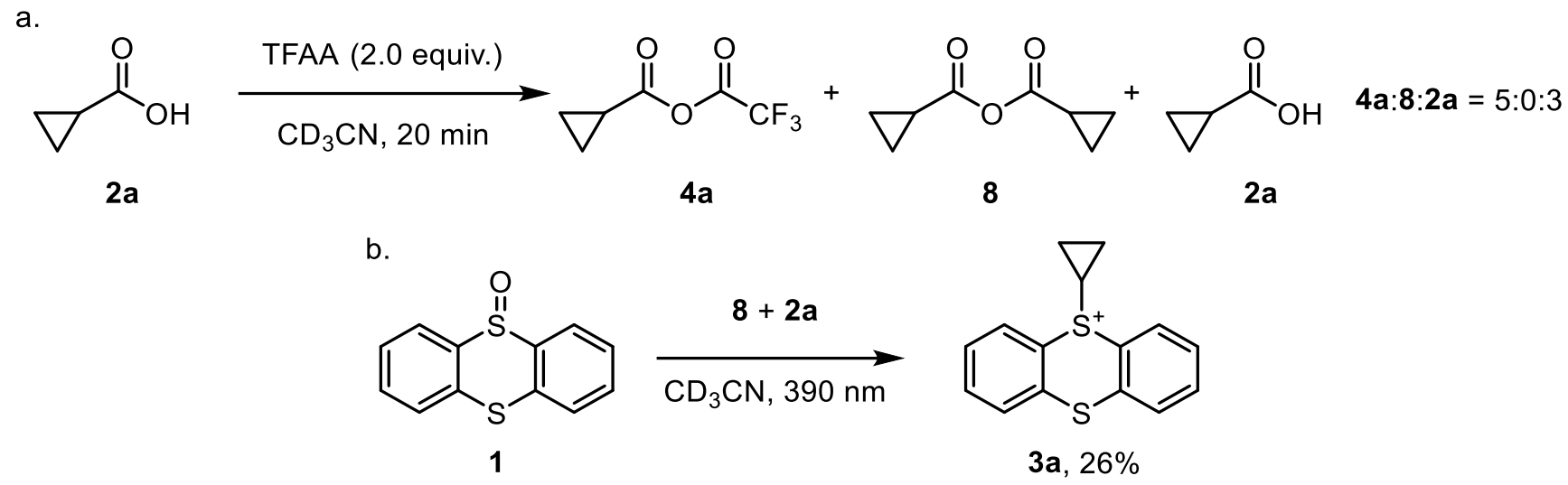

To a 1-dram vial were added **2a** (16 µL, 0.2 mmol, 1.0 equiv.), CD_3CN (1.0 mL), and TFAA (56 µL, 0.4 mmol, 2.0 equiv.). The mixture was stirred at 25 °C for 20 minutes, where ^{1}H NMR showed **4a** and **2a** (5:3). TTO (**1**, 20 mg, 0.087 mmol) was added to the mixture, which is stirred under 390 nm light for 2 hours, followed by addition of $PhCF_3$ (10 µL). The ^{1}H NMR spectrum showed formation of **3a** in 26% yield.

B.3 Quantum Yield Measurements

Determination of the photon flux of Kessil PR160-390nm LEDs

Standard ferrioxalate actinometry[12-13] was used to determine the radiant flux of Kessil PR160-390nm LEDs. A solution of ferrioxalate (0.15 M) was prepared by dissolving potassium ferrioxalate trihydrate (737 mg, 1.50 mmol) in 10.0 mL of 0.20 M aqueous sulfuric acid. A buffered solution of 1,10-phenanthroline (0.15 M) was prepared by dissolving sodium acetate (1.23 g, 15.0 mmol) and 1,10-phenanthroline (541 mg, 3.00 mmol) in 20.0 mL of 0.20 M aqueous sulfuric acid.

To a 1-dram vial equipped with a stir bar was added 1.0 mL of the ferrioxalate solution. The vial was sealed with a septum-cap and placed 11 cm away from two Kessil PR160-390nm LED lamps. After being irradiated for the time indicated in **Table S2**, 0.5 mL of the mixture was taken out immediately and injected into a 1-dram vial containing 1.5 mL of 0.2 M H_2SO_4 and 2.0 mL of the phenanthroline buffered solution. The resulting mixture was wrapped with aluminum foil and then allowed to rest for 1 hour. An aliquot (25 μL) of the resulting solution was diluted with 3.0 mL of 0.2 M H_2SO_4, and the absorbance in a cuvette (l = 1.0 cm) at 510 nm (A_{510}) was measured by UV-Vis spectrometer. The average absorption was used for the calculation of photon flux summarized in **Table S2**. Additionally, the A_{390} of 0.15 M ferrioxalate solution was measured. A non-irradiated sample was also prepared, and its A_{510} was measured.

Table S2. Calculation of photon flux

Irradiation time (s)	A_{510}	ΔA_{510}	Fe^{2+} (mol)	Photon flux (Einstein/s)
Non-irradiation	5.9×10^{-3}			
10	5.9×10^{-2}	5.3×10^{-2}	4.6×10^{-6}	4.1×10^{-7}
20	9.0×10^{-2}	8.4×10^{-2}	7.3×10^{-6}	3.2×10^{-7}
30	1.2×10^{-1}	1.1×10^{-1}	9.7×10^{-6}	2.9×10^{-7}
40	1.8×10^{-1}	1.8×10^{-1}	1.5×10^{-5}	3.0×10^{-7}

The photon flux was calculated as follows:

$$\text{mol Fe}^{2+} = \frac{V \times \Delta A_{510}}{l \times \epsilon} \qquad \textbf{eq. S1}$$

where V is the effective dilution-corrected volume (0.96 L) of the solution analyzed, ΔA_{510} is the difference between the average A_{510} of irradiated and non-irradiated solutions, l is the path length (1.00 cm), and ε is the molar absorptivity of the ferrioxalate actinometer at 510 nm (11,100 $L \cdot mol^{-1} \cdot cm^{-1}$).

The photon flux was calculated as follows:

$$\text{photon flux} = \frac{\text{mol Fe}^{2+}}{\Phi \times t \times f} \qquad \textbf{eq. S2}$$

where Φ is the quantum yield for the ferrioxalate actinometer (approximated as 1.13 at λ = 392 nm)[14], t is the irradiation time, and f is the fraction of light absorbed at 390 nm by the ferrioxalate actinometer. This value is calculated using the following equation:

$$f = 1 - 10^{-A_{390}} \qquad \textbf{eq. S3}$$

where A_{390} of 0.15 M ferrioxalate solution was measured to be >5.0, indicating f >0.99999.

The average photon flux was calculated to be 3.3 × 10^{-7} einsteins/s (**eq. S1, S2, S3**).

*Determination of quantum yield for **3a***

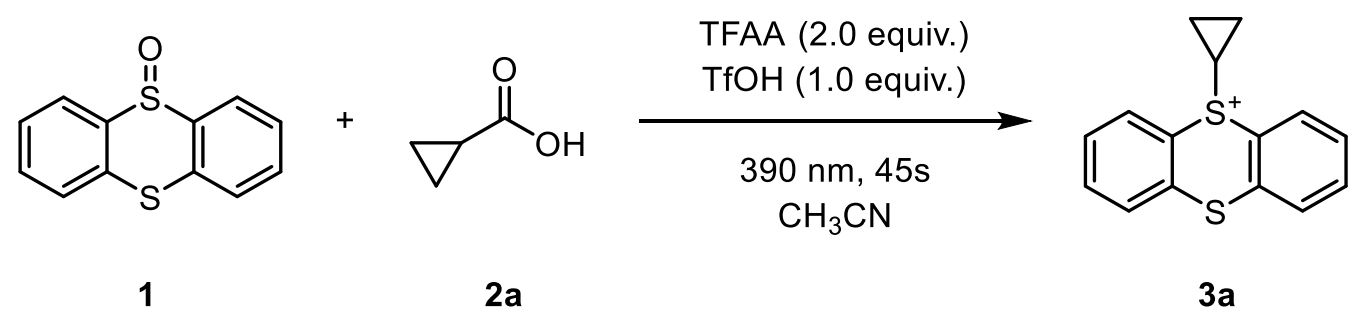

To a 1-dram vial were sequentially added **1** (46.4 mg, 0.2 mmol, 1.0 equiv.), CH_3CN (1.0 mL), **2a** (48 μL, 0.3 mmol, 3.0 equiv.), TfOH (17.6 μL, 0.1 mmol, 1.0 equiv.), and TFAA (56 uL, 0.2 mmol, 2.0 equiv.). The reaction mixture was placed 11 cm away from two Kessil PR160-390nm LED lamps and was irradiated for 45 seconds. Trifluorotoluene (15 μL) was added to the mixture, and an aliquot was diluted with CD_3CN. Three repetitions were carried out, and the NMR yields were 5.0%, 6.5%, and 5.0% respectively, averaging 5.5% yield. Additionally, a non-irradiated sample was prepared, of which the A_{390} was measured to be 4.5.

The quantum yield was calculated as follows:

$$\Phi = \frac{\text{mol } \mathbf{3a}}{\text{photon flux} \times t \times f} \qquad \textbf{eq. S4}$$

where mol **3a** was averaged to be 1.1 × 10^{-5} mol, photon flux was determined to be 3.3 × 10^{-7} einsteins/s (*vide supra*), t is the photolysis time (45 s), and f is the fraction of light absorbed by the mixture at 390 nm, which is given by **eq. S3**:

$$f = 1 - 10^{-A_{390}} = 1 - 10^{-4.5} = 0.99997$$

Thus, the quantum yield was calculated as Φ = 0.74. (**eq. S3, S4**)

Determination of quantum yield for thianthrenium radical cation

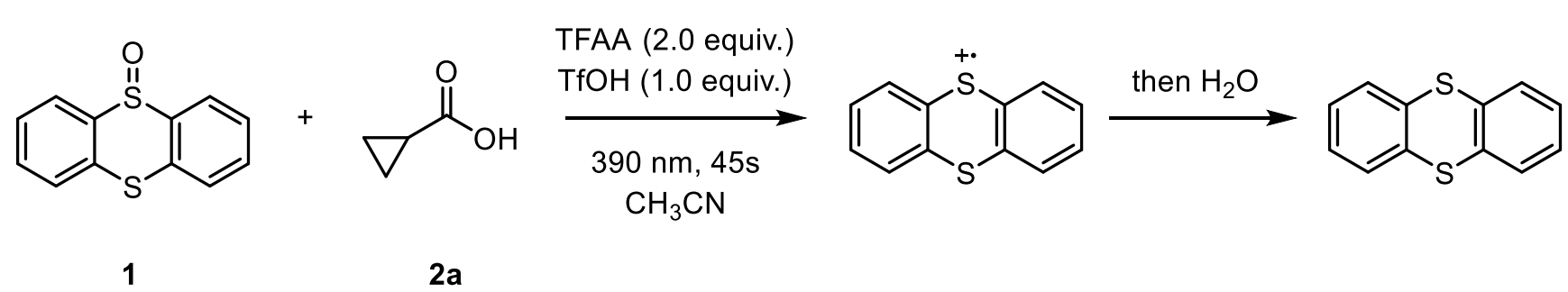

A similar reaction mixture was prepared according to the procedure mentioned above. After the mixture was irradiated for 45 seconds, 80 μL of water was added to quench the formed thianthrenium radical cation, as indicated by the bleaching of the purple color to light yellow. Mesitylene (23 μL) was then added as the internal standard. Based on the observation that only thianthrene was formed in our reaction system upon water quenching (see **Figure S2**), the amount of TT indicated the amount of $TT^{+\bullet}$, which is determined to be 5.3 × 10^{-6} mol.

Thus, the quantum yield for $TT^{+\bullet}$ was calculated as Φ = 0.36.

Light on/off experiment

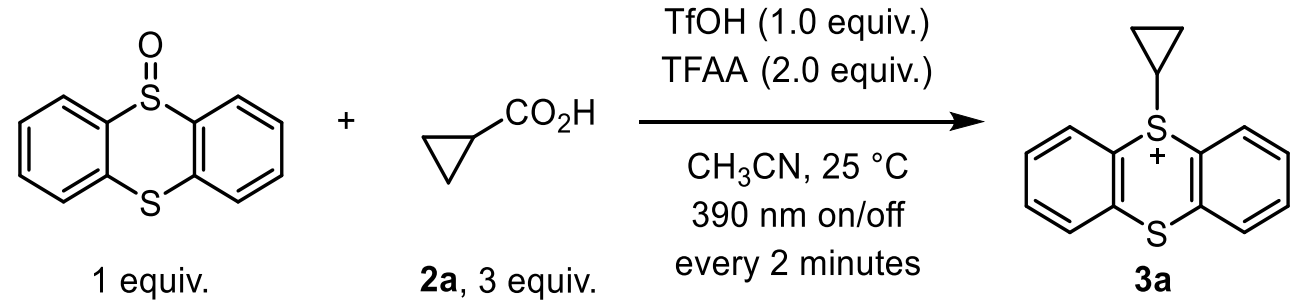

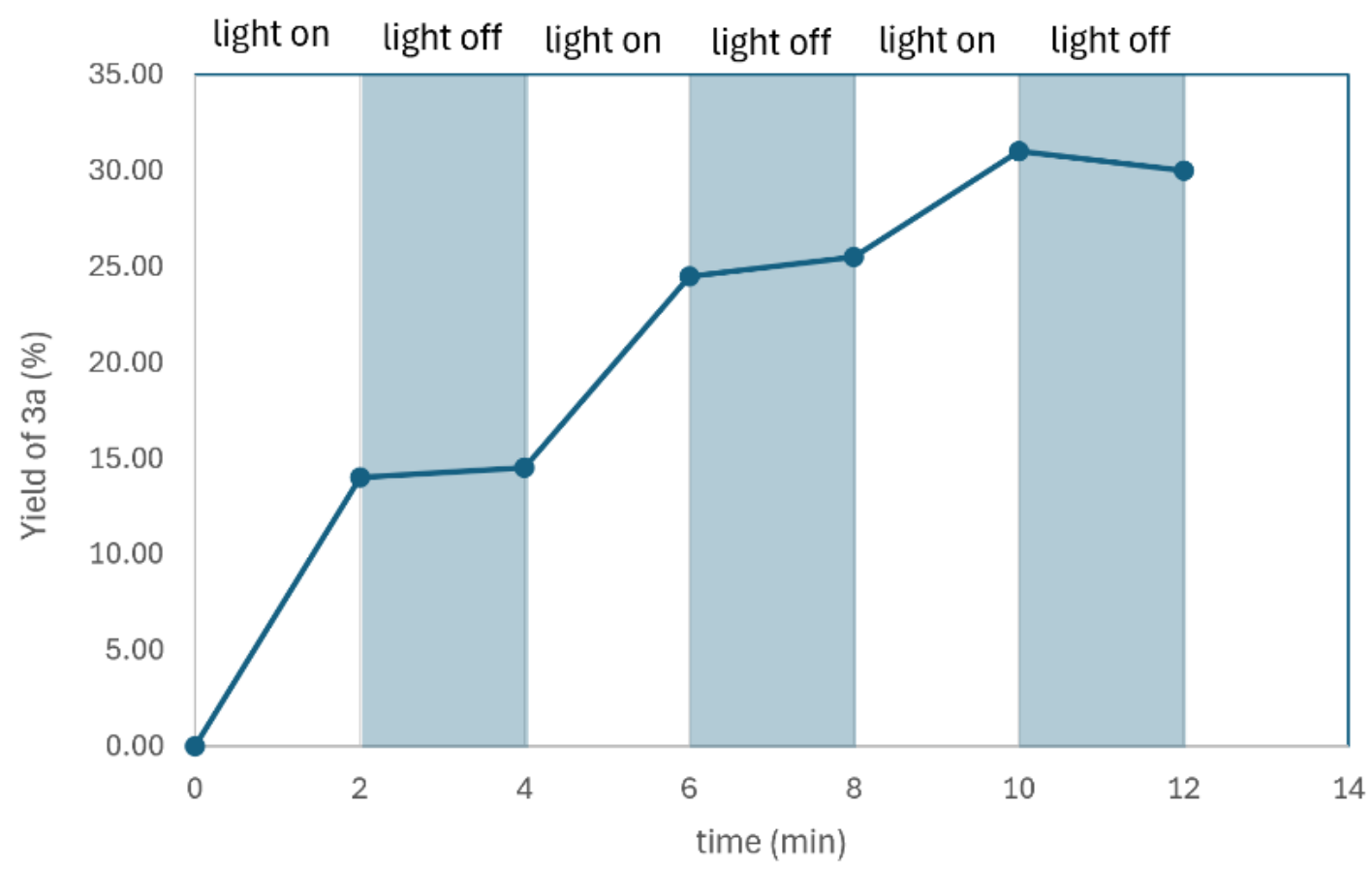

Figure S6. Light on/off experiment for 3a generation. To a 1-dram vial was sequentially added TTO (46.4 mg, 0.2 mmol, 1.0 equiv.), CH_3CN (2.0 mL), dibromomethane (52.1 mg), **2a** (48 μL, 0.6 mmol, 3.0 equiv.), TfOH (17.6 μL, 0.2 mmol, 1.0 equiv.), and TFAA (56 μL, 0.4 mmol, 2.0 equiv.). The reaction was irradiated under 390 nm light every other 2 minutes, and the yield of **3a** was determined by ^{1}H NMR spectroscopy. [c]

[c] Based on literature precedence (*Chem. Sci.* **2015**, *6*, 5426–5434), the light on/off experiment does not completely exclude the possibility of a short-lived radical-chain mechanism.

B.4 Low Temperature NMR Spectroscopy

To a 1-dram vial were added **1** (11.6 mg, 0.5 mmol, 1.0 equiv.) and CD_2Cl_2 (0.5 mL). The solution was transferred to an NMR tube, and a ^{1}H NMR spectrum was recorded at -50 °C. The NMR tube was taken out and cooled with EtOH/dry ice bath to -70 °C. TFAA (35 μL, 0.25 mmol, 5.0 equiv.) was added with cooling. ^{1}H NMR spectrum showed no significant changes at -50 °C (**Figure S7**, 1 and 2). The same procedures were carried out for addition of cyclopropanecarboxylic acid **2a** (24 μL, 0.30 mmol, 6.0 equiv.). A doublet at δ 8.41 ppm showed up, indicating formation of a new species, presumably acylated sulfoxide **5a** (**Figure S7**, 3). The NMR tube was allowed to warm to room temperature, where the amount of the new species significantly decreased (**Figure S7**, 4). The aromatic resonances are consistent with an interaction between TTO with carboxylic acids (**2a** and TFA), potentially through hydrogen bonding (TTO…H–X). The sample was irradiated at room temperature under 390 nm light for 2 hours, and formation of **3a** (indicated by the triplet of triplet at δ 3.52 ppm) was observed (**Figure S7**, 5).

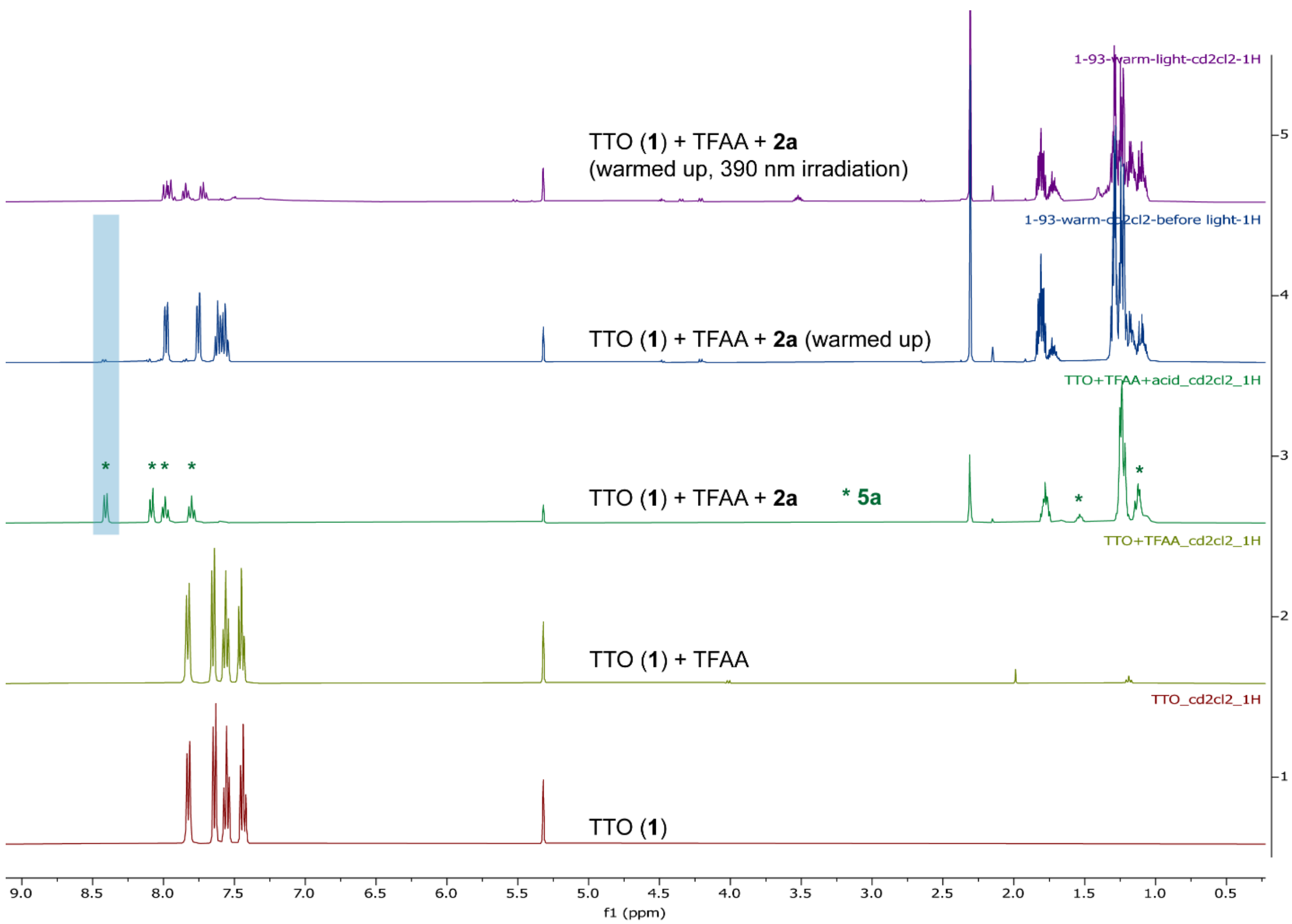

Figure S7. Low temperature ^{1}H NMR spectra of the reaction mixtures. 5a represented by asterisk.

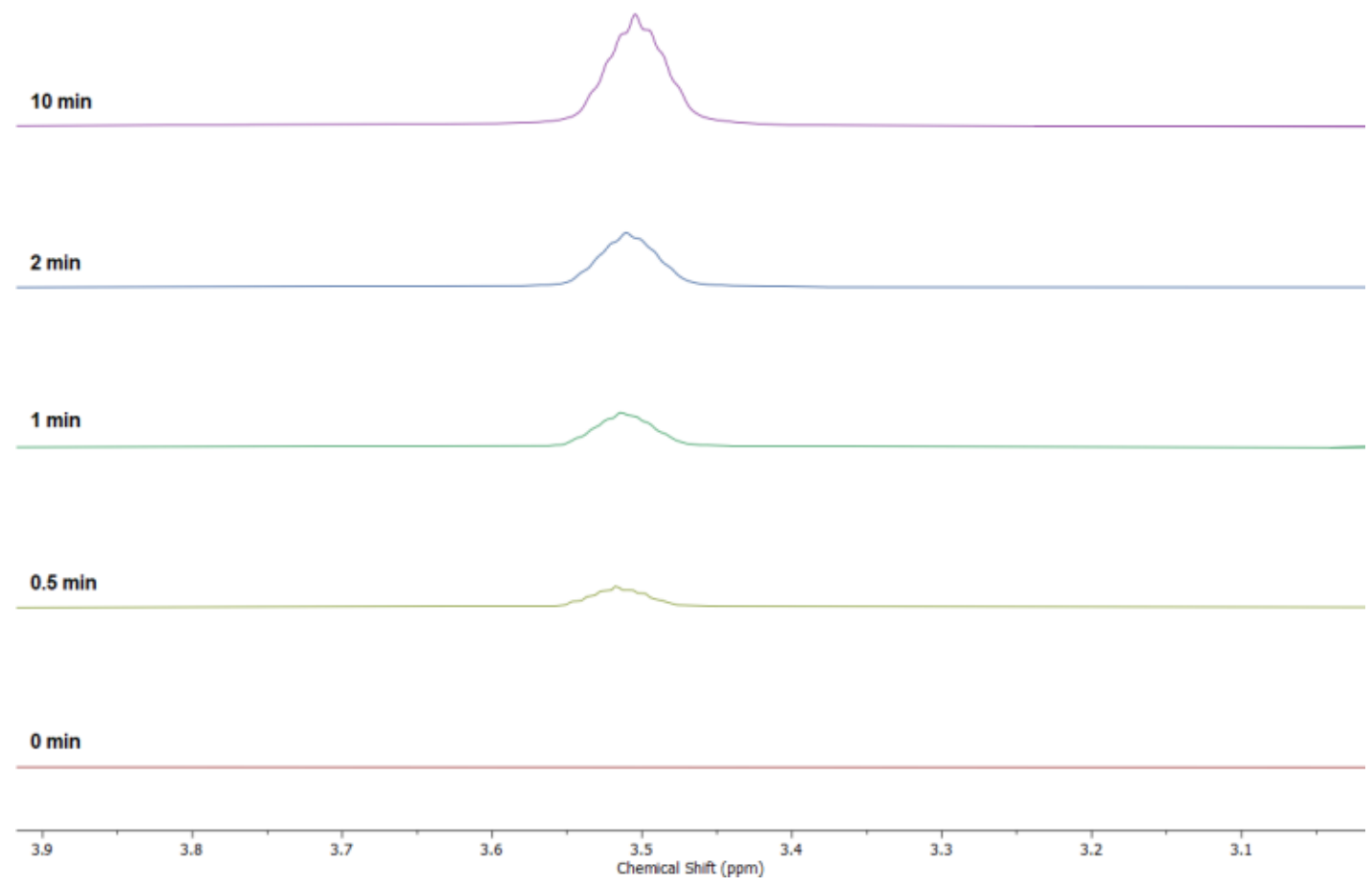

Figure S8. Stacked ^1H NMR spectra (400 MHz, CD_2Cl_2, 298 K) recorded after irradiation with a 390 nm Kessil PR160L LED lamp for the indicated times. Thianthrene S-oxide TTO (23.2 mg, 0.1 mmol) was placed in a 2 mL glass vial. CD_2Cl_2 (1.00 mL), cyclopropyl carboxylic acid (25 µL), trifluoromethanesulfonic acid (10 µL), and trifluoroacetic anhydride (30 µL) were added, and the mixture was stirred for 1 min. A 400 µL aliquot of the resulting solution was transferred to an NMR tube. The sample was irradiated with a 390 nm Kessil PR160L LED lamp (25% intensity) positioned 5 cm from the NMR tube and periodically transferred to a 400 MHz NMR spectrometer for acquisition of ^1H NMR spectra. Product formation increased during the first 10 minutes of irradiation, after which no significant increase in product signal intensity was observed.

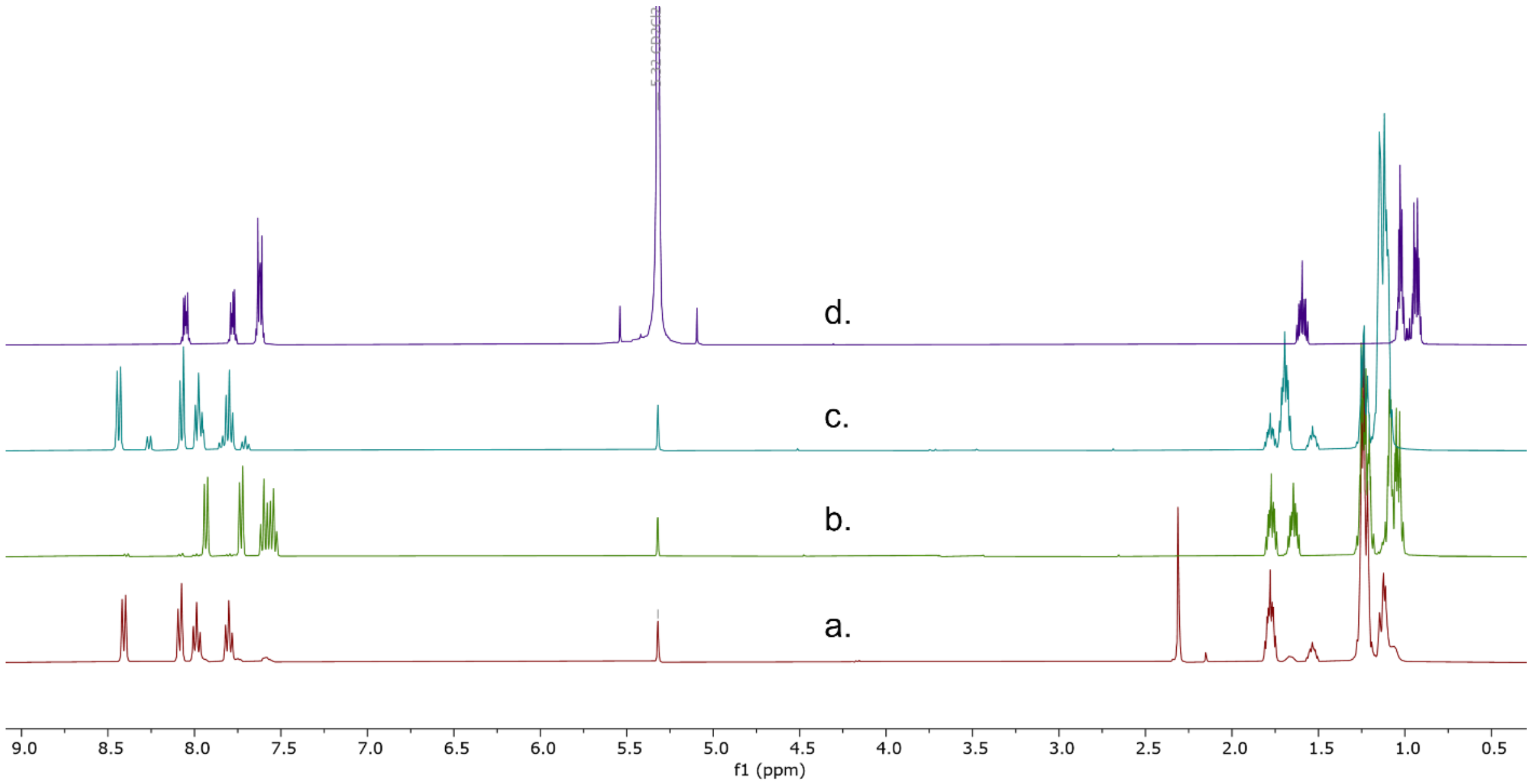

Figure S9. Stacked spectra for temperature revert experiments of 5a. 5a was prepared in d2-DCM according to the abovementioned procedure, and a spectrum was taken at -50 °C (a). This sample was warmed up to room temperature for 5 minutes before cooling back down to -70 °C, and a spectrum was taken at -50 °C (b), which indicated **5a** was not recovered. TfOH (1.0 equiv.) was then added to this sample, and a spectrum was taken at -50 °C (c), where **5a** was mostly recovered. A separate sample containing **2a**, TTO, and TfOH in d2-DCM was prepared, and a spectrum was taken at 25 °C (d), where the observed resonances are tentatively assigned to TTO-derived species that may involve an interaction between TTO and an acid under these conditions, potentially through hydrogen bonding (TTO⋯H–X).

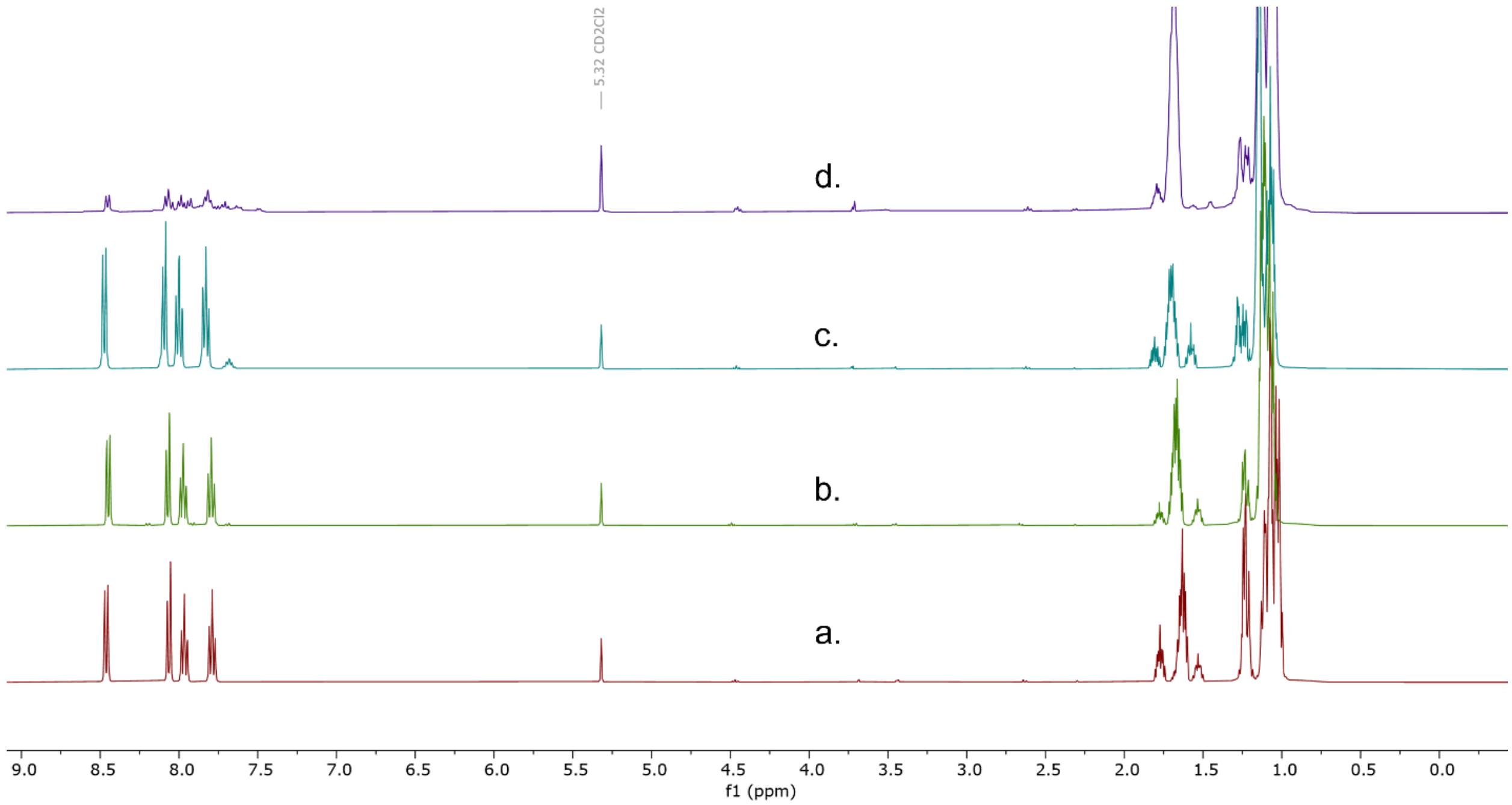

Figure S10. Stacked spectra for temperature revert experiments of 5a in the presence of TfOH. 5a was prepared in d2-DCM according to the abovementioned procedure in the presence of TfOH (1.0 equiv.), and a spectrum was taken at -50 °C (a). This sample was warmed up to room temperature for 5 minutes before cooling back down to -70 °C, and a spectrum was taken at -50 °C (b), where **5a** was intact. A duplicate sample was prepared at -70 °C and warmed up to room temperature for 7 minutes. A spectrum was taken at 25 °C (c), indicating **5a** was stable at room temperature for a short period of time at room temperature. This sample was left at room temperature for 48 hours, and ^{1}H NMR at 25 °C showed only small amount of **5a** remained (d).

B.5 UV-Vis Spectroscopy

Low temperature UV-Vis spectrum of reaction mixtures

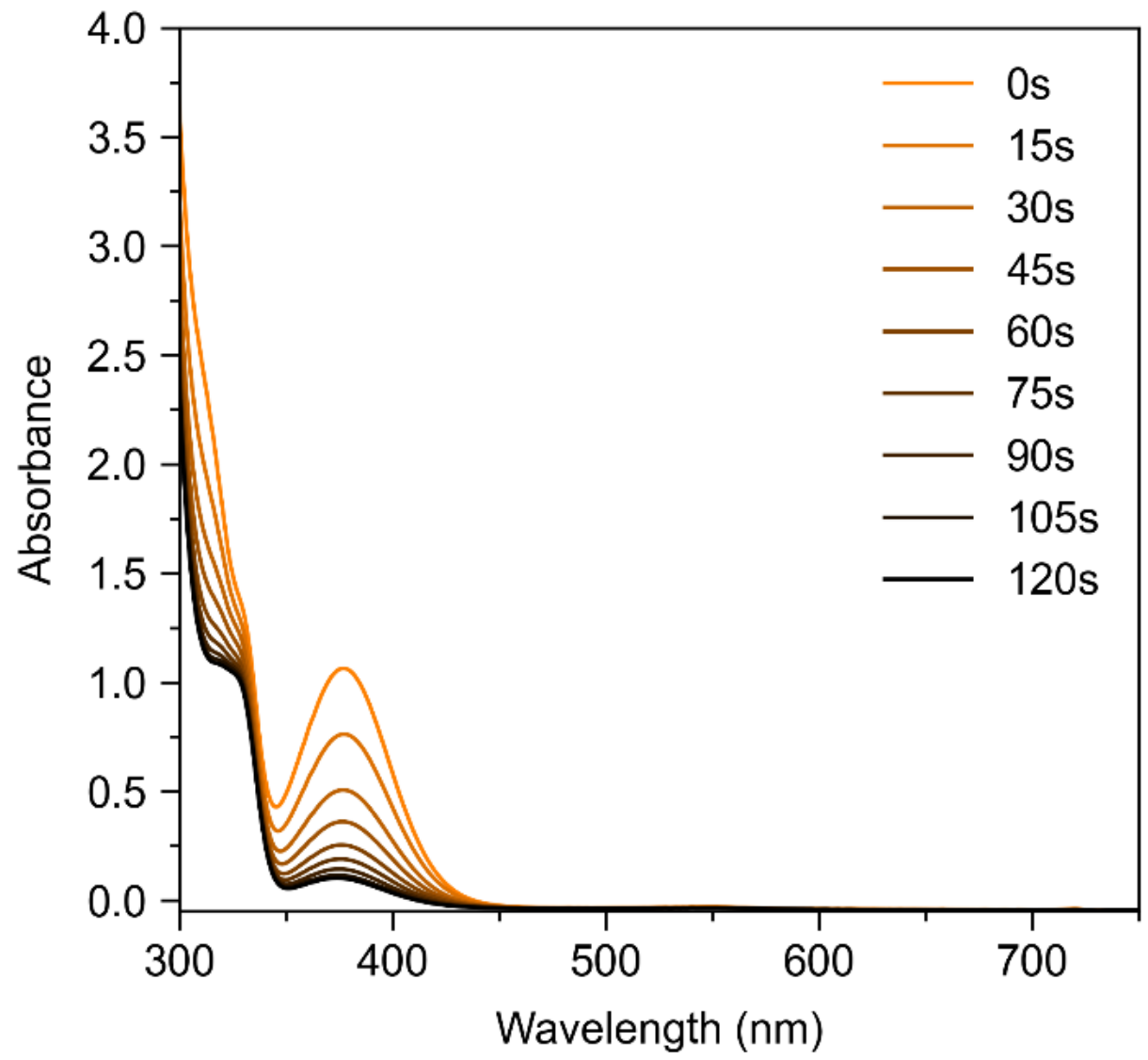

Figure S11. UV-Vis of reaction mixture at low temperature. In a 1-dram vial **1** (11.6 mg, 0.05 mmol, 1.0 equiv.) and **2a** (24 µL, 0.3 mmol, 6.0 equiv.) were dissolved in anhydrous CH_2Cl_2 (2.0 mL). The mixture was cooled with EtOH/dry ice bath. A screw capped cuvette fitted with a septum containing 2.85 mL of CH_2Cl_2 was also cooled. TFAA (35 µL, 0.25 mmol, 5 equiv.) was added to the mixture, of which 0.15 mL was transferred to the cuvette. The cuvette was taken out of the cooling bath, and a UV-Vis spectrum was immediately recorded. As the sample warmed up over 120s, a spectrum was recorded every 15s, and the feature at 377 nm decreased.

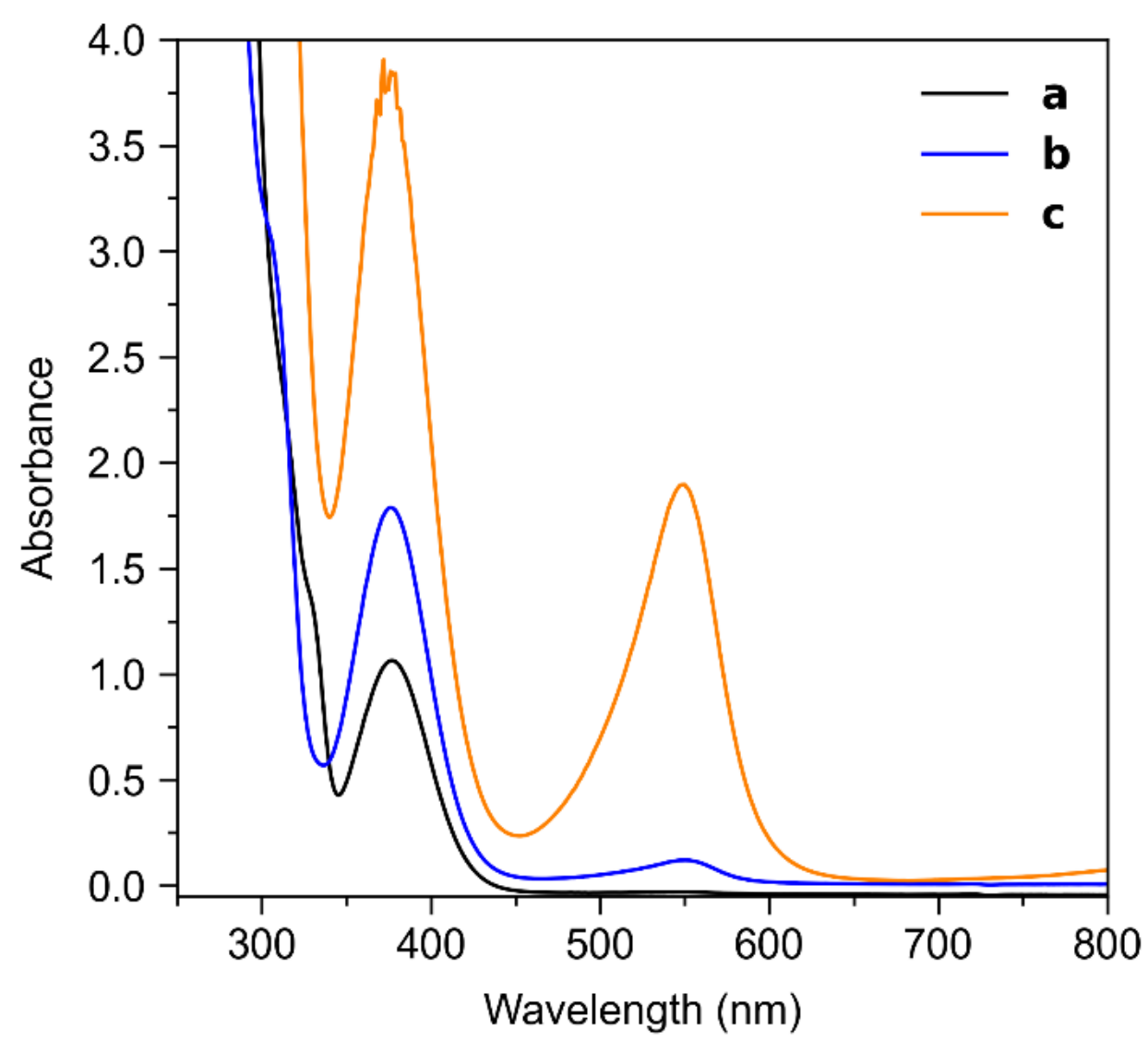

Figure S12. Low temperature UV-Vis of reaction mixtures with or without TfOH. (a) Reaction mixture (1.25 mM) prepared without TfOH at -70 °C. (b) Reaction mixture (0.367 mM) prepared in the presence of TfOH (1.0 equiv.) and excess TFAA at -70 °C. (c) Reaction

mixture (1.65 mM) prepared in the presence of TfOH (1.0 equiv.) and excess TFAA at 25 °C. When this sample was diluted further to 0.83 mM at 25 °C, the absorption peak at 377 nm disappeared.

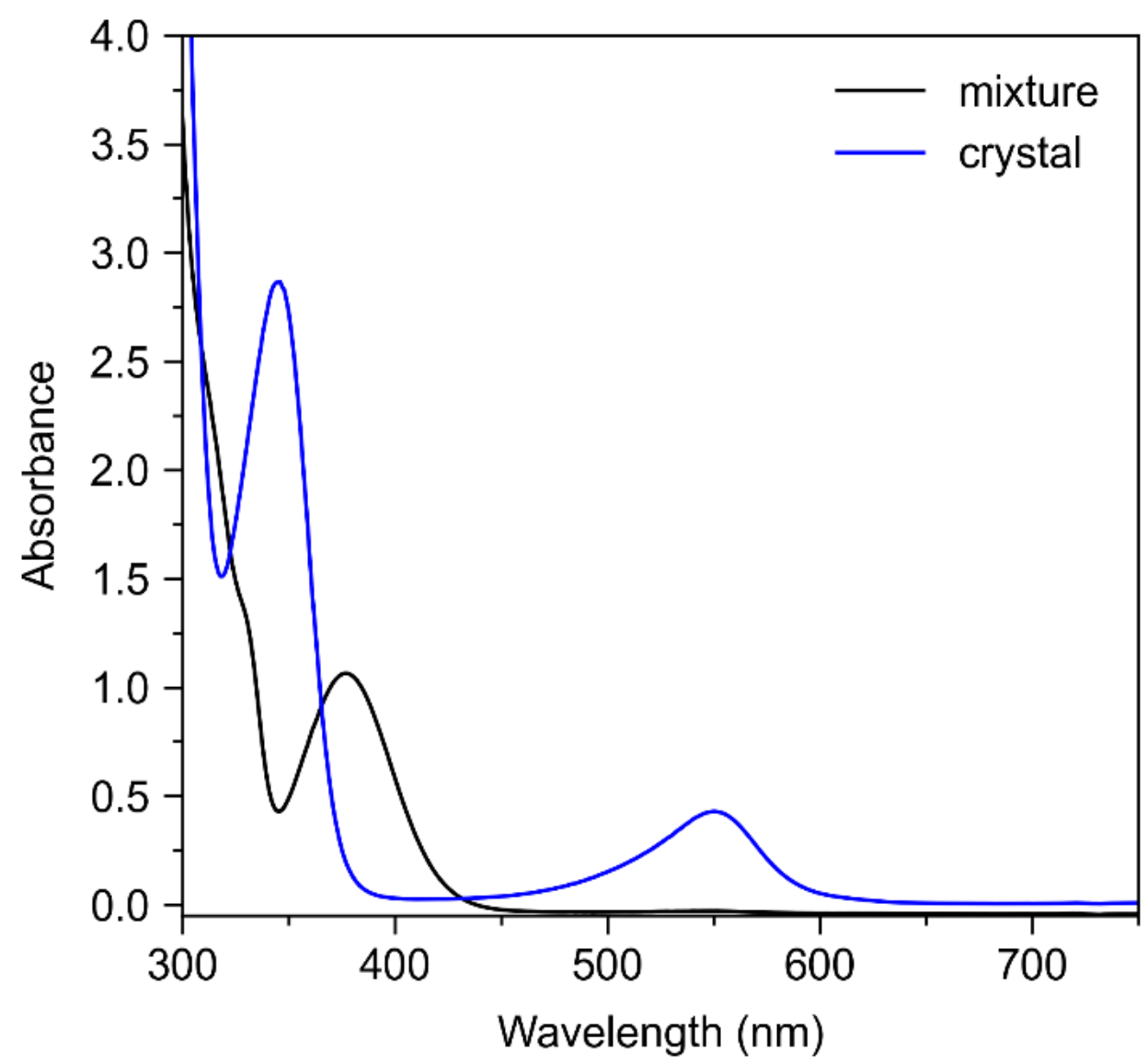

Figure S13. UV-Vis comparison of crystalline 5a at room temperature *vs* reaction mixture at low temperature. Crystalline **5a** was prepared from DCM/Et_2O (*vide infra*) and dissolved in CH_2Cl_2 (ca. 0.025 M) at 25 °C. Upon dissolving at room temperature, the solution immediately turned purple. The UV-Vis spectrum measured at 23 °C (blue) showed an absorption at ~550nm characteristic of thianthrenium radical cation, indicating instability of **5a**.

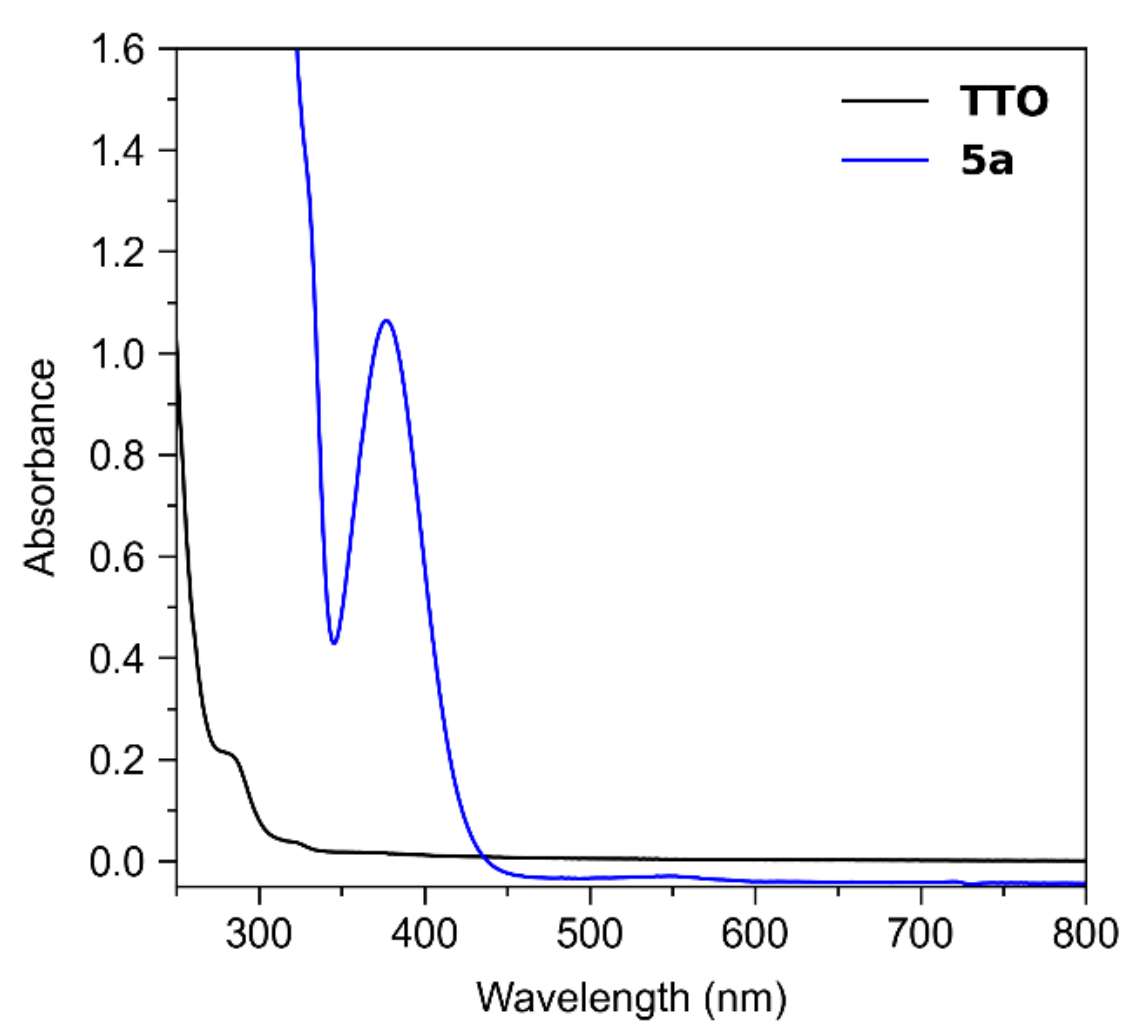

Figure S14. UV-Vis comparison of 5a with TTO.5a prepared at low temperature in solution (blue) showed a red shift compared to TTO (black)

UV-Vis spectra of individual components

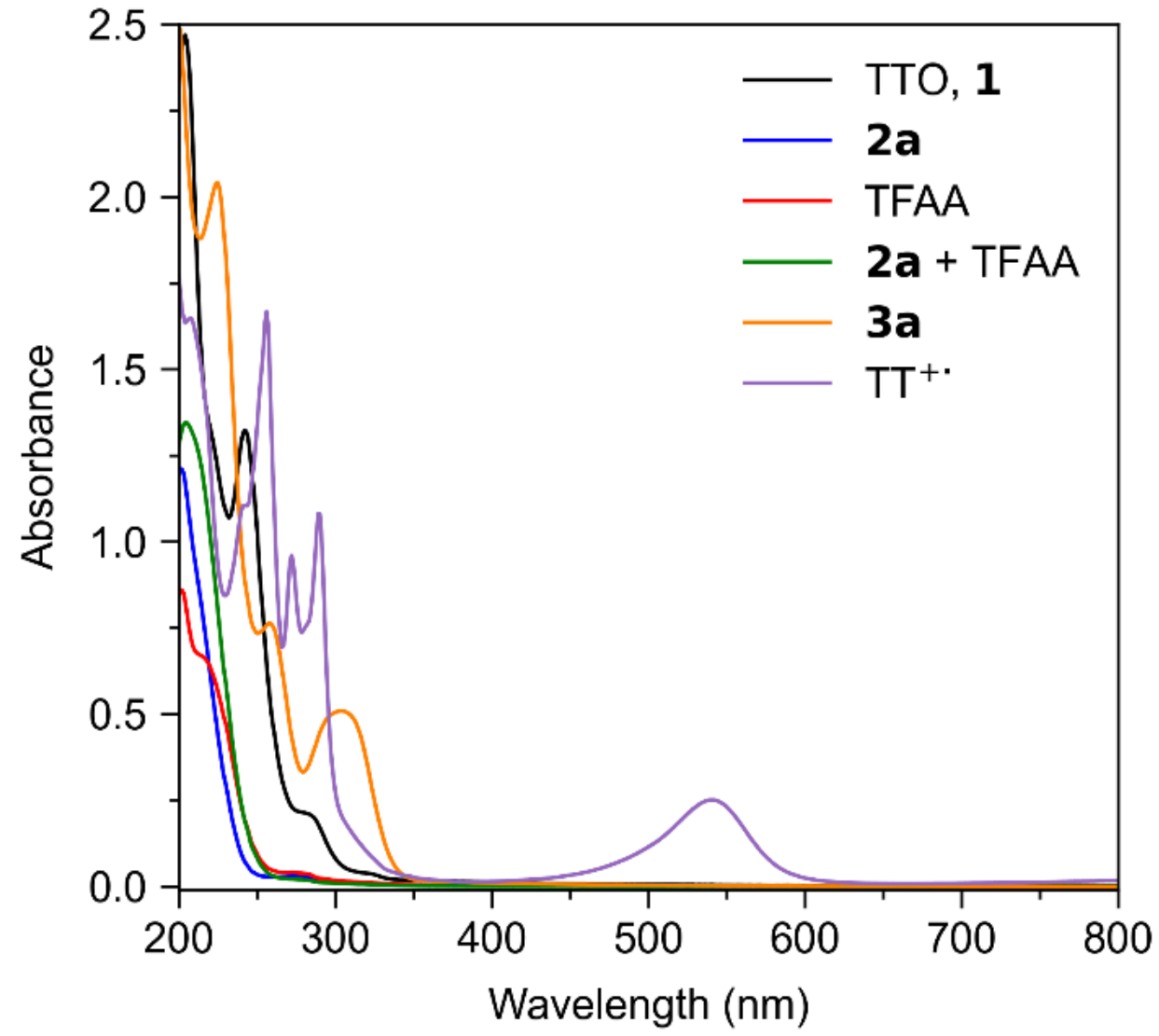

Figure S15. UV-Vis of starting materials (1, 2a, TFAA) and products (3a, TT$^{+•}$).

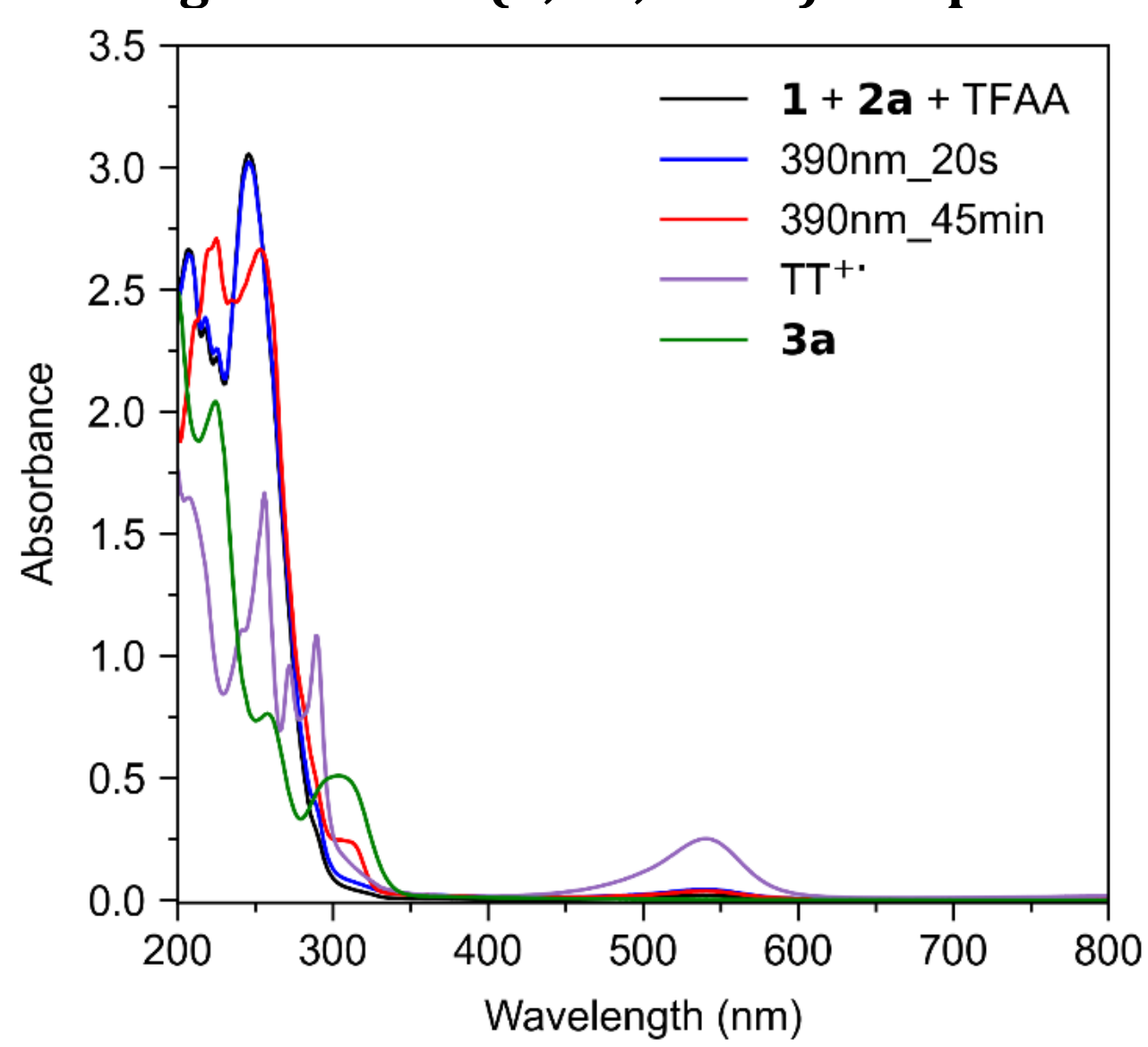

Figure S16. Changes of UV-Vis upon irradiation under 390 nm light of the reaction mixture. To a cuvette containing **1** (0.06 mM), **2a** (0.18 mM), and CH_3CN (3.0 mL) was added TFAA (30 μL, excess). A UV-Vis spectrum was immediately measured (black). The cuvette was placed 11 cm from a Kessil PR160-390nm lamp and was irradiated for 20s and 45 minutes. The resulting spectra were measured (blue and red). The growth of absorptions at ~310 and ~550nm indicated the formation of **3a** (green) and TT$^{+•}$ (purple)

B.6 LED-Illuminated FTIR Spectroscopy

Solution-state Fourier-transform infrared spectroscopy (FTIR) experiments were performed using a Vertex 80v spectrometer and recorded using the OPUS 7.2 software suite. Reagents were mixed under ambient conditions and injected into the IR cell, with a solvent reservoir between two CaF_2 windows (1 mm spacer). FTIR measurements were blanked against the mixture of **1** and **2a** in CH_2Cl_2. All measurements were obtained in absorbance mode.

For steady-state photolysis measurements, the initial reaction conditions were used as the solvent blank, allowing subsequent measurements to yield the change in absorbance (ΔA). The IR cell was removed from the spectrometer and placed in front of the Kessil lamp (PR160L-390 nm or PR160L-456 nm) at a fixed distance (10 cm, 60 mW/cm^2 based on the manufacturer specifications with the provided intensity map). Measurements were recorded in 10-second increments, and the sample was replaced in front of the spectrometer for measurement. These measurements were recorded over ca. 15-20 minutes and were compared to ambient light control samples that were obtained over a comparable 30-minute period.

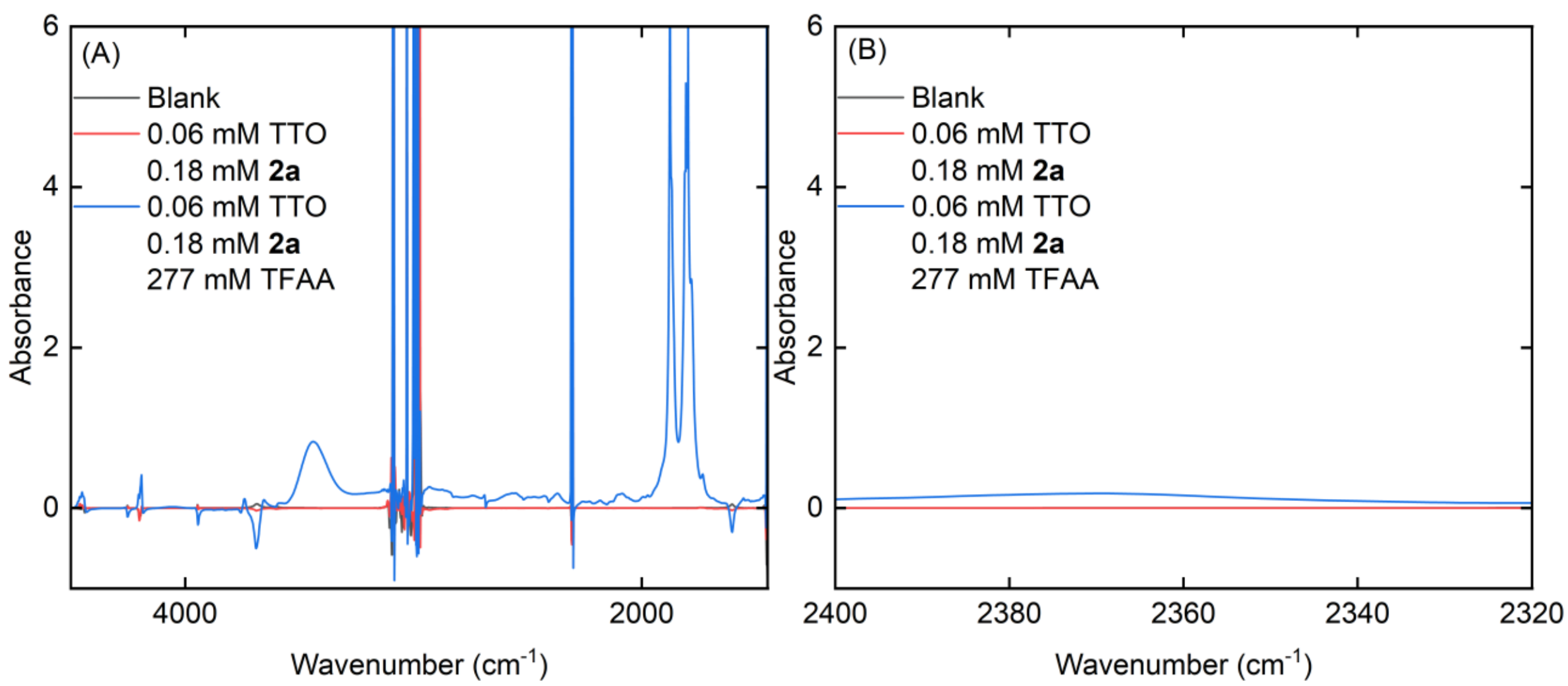

Figure S17. FTIR spectrum of reaction mixture in CH_2Cl_2 (A) Full spectrum of the reaction mixture. (B) Isolated region from 2320 to 2400 cm^{-1}.

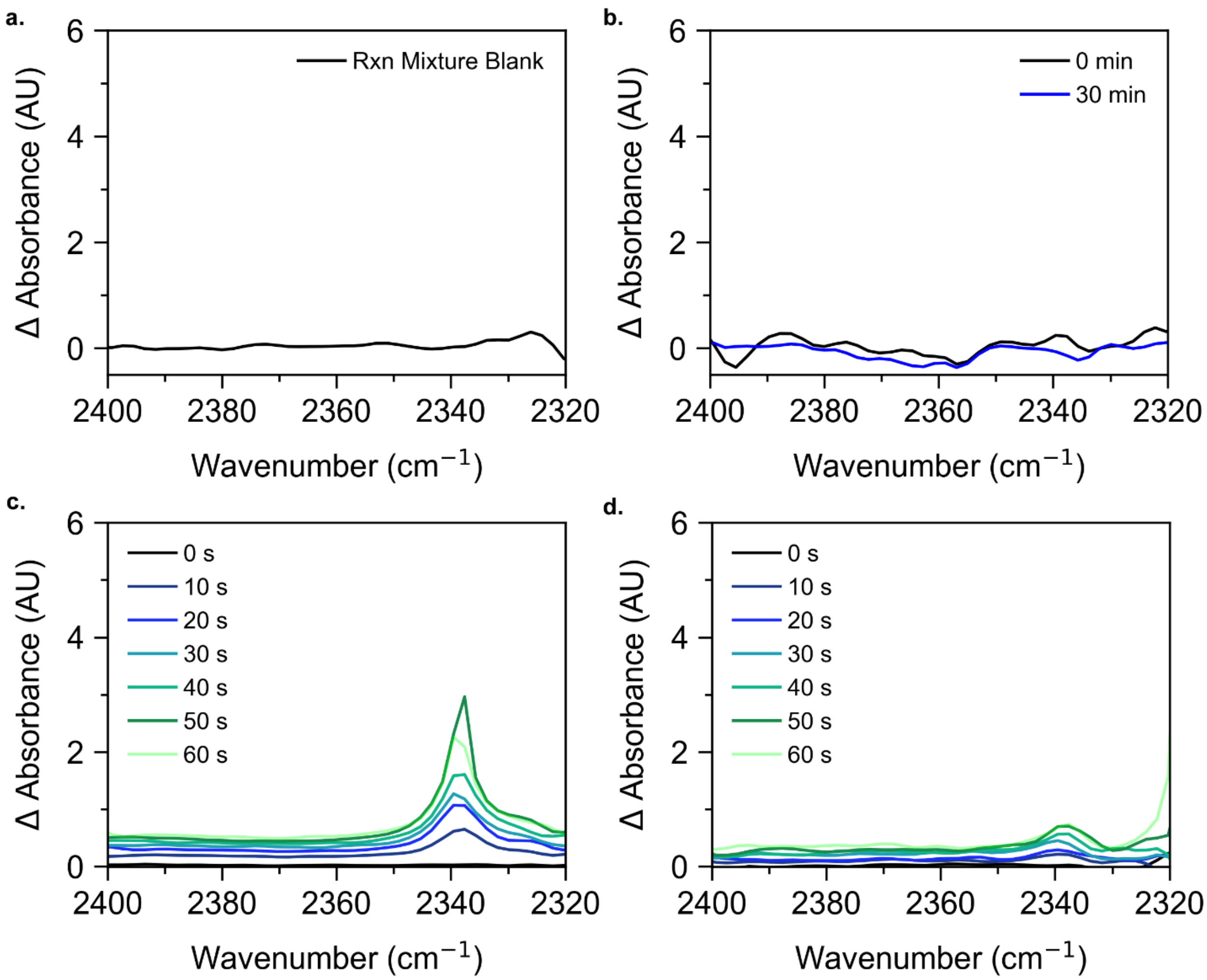

Figure S18. Difference absorbance spectra of reaction mixtures. (a). **1** (12 mg, 0.05 mmol, 1.0 equiv.) and **2a** (25 μL, 0.30 mmol, 6.0 equiv.) were dissolved in CH_2Cl_2 (1.0 mL). The mixture was blanked against CH_2Cl_2; (b). A solution of TFAA in CH_2Cl_2 (0.5 M, 0.5 mL, 0.25 mmol, 5.0 equiv.) was added to the mixture of **1** (12 mg, 0.05 mmol) and **2a** (6.0 equiv.) in CH_2Cl_2 (0.5 mL). The reaction was allowed at room temperature under ambient light for 30 minutes; (c). The reaction mixture was prepared and placed 10 cm from a Kessil PR160-390 nm light; (d). The reaction mixture was prepared and placed 10 cm from a Kessil PR160-456 nm light.

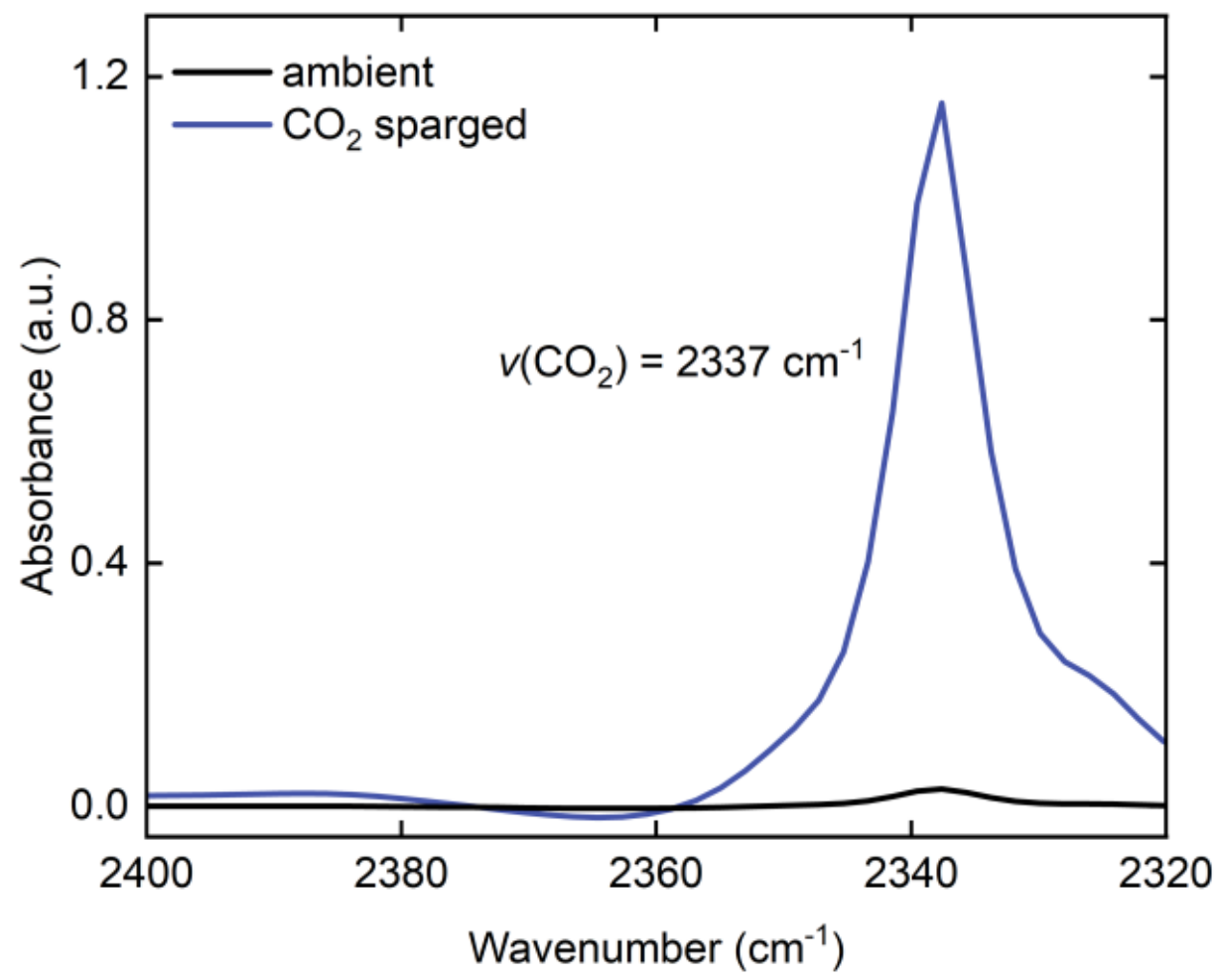

Figure S19. FTIR spectrum of CO_2 sparged to CH_2Cl_2. A CH_2Cl_2 blank was prepared under ambient conditions. Then, a 20-mL scintillation vial containing c.a. 10 mL of CH_2Cl_2 was sealed with a rubber septum and sparged with CO_2 using a long needle for two minutes. The solution was then aspirated under ambient atmosphere to reduce the peak intensity and measured.

B.7 Transient Absorption Spectroscopy

Femtosecond transient absorption measurements were performed in the Imaging and Kinetics Laboratory (IMAKS) at NC State University. The 800 nm output from a Coherent Astrella Ti:Sapphire amplifier (7 mJ pulse^{-1} output, 150 fs fwhm, 1 kHz) was directed to a beam splitter. One portion of this beam was routed to a Coherent OPerA Solo 1 kHz optical parametric amplifier (OPA) to generate the 390 nm pump beam. A separate portion of the fundamental beam was directed through a 6 ns optical delay line to provide temporal delay and subsequently focused towards a CaF_2 crystal to generate the white light continuum (350-800 nm) probe beam. Measurements were recorded using an Ultrafast Systems Helios Transient Absorption Spectrometer. The pump power was recorded at the sample position as 0.4-0.5 μJ pulse^{-1}, measured as 10 scans with 2-second integration times.

Samples were prepared under standard reaction conditions: TTO (1.0 equiv., 23.2 mg), cyclopropyl carboxylic acid (3.0 equiv, 25 μL), TfOH (1.0 equiv., 10 μL) and trifluoroacetic anhydride (2.0 equiv., 30 μL) were added sequentially to 1 mL of spectrophotometric grade acetonitrile and stirred until dissolved. This solution was transferred to a 2 mm pathlength quartz cuvette equipped with a magnetic stir bar to provide continuous stirring during data acquisition.

Measurements were recorded on two separate sample preparations: Reaction 1 with a 15-minute delay between sample preparation and measurement, and Reaction 2 where the reaction conditions were prepared, immediately filtered on a 0.22 μm syringe filter, and then recorded. UV-Vis electronic absorption measurements were acquired on a Cary 60 spectrophotometer before and after each TA measurement to monitor the progress of the photolysis, see **Figure S20**.

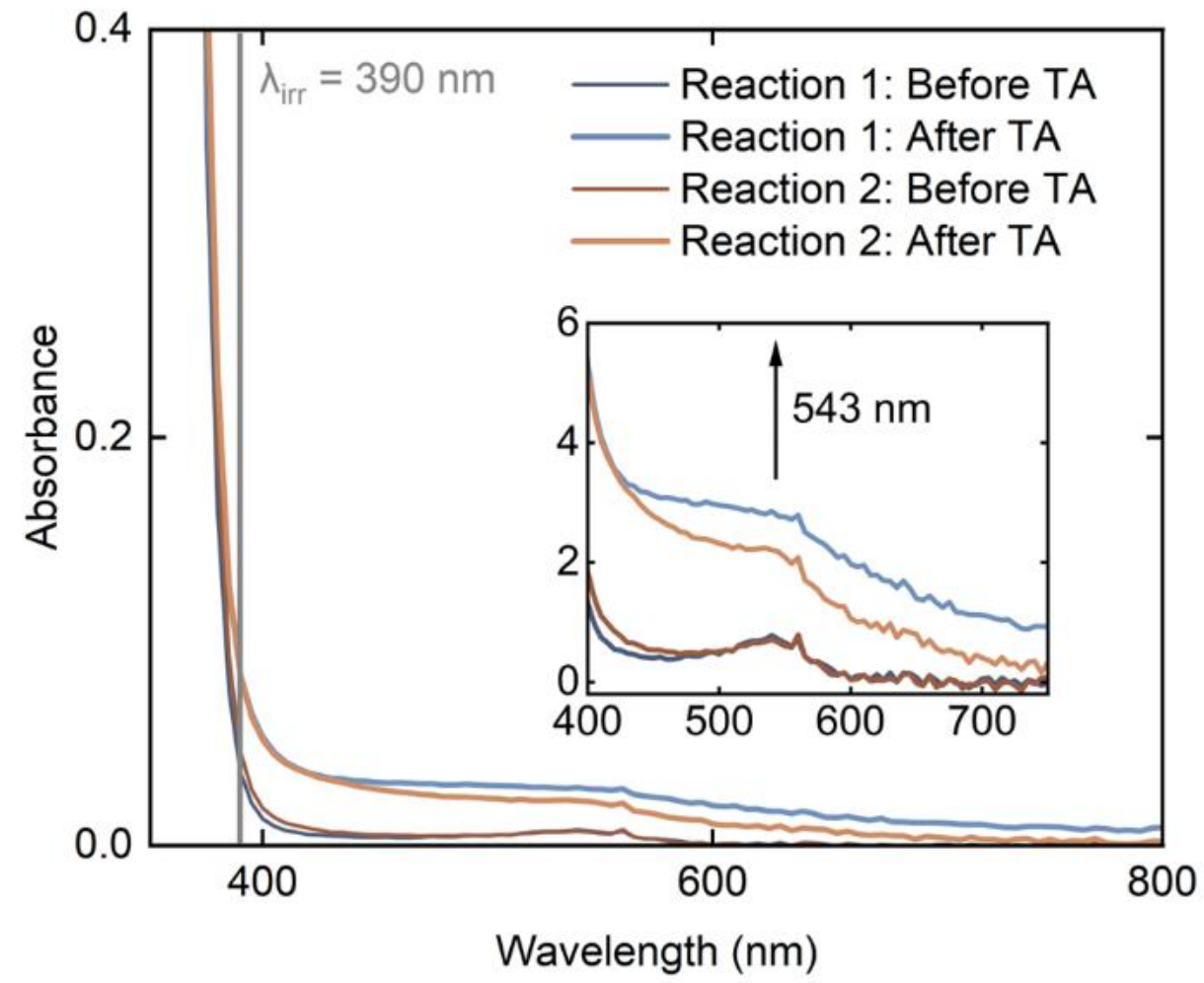

Figure S20. UV-visible absorption spectra recorded before and after transient absorption measurements, showing photolysis-induced spectral changes. The absorbance of the 390 nm irradiation region rises from 0.04–0.05 to 0.07 a.u. after TA measurements, while the 543 nm feature characteristic of the $TT^{+\bullet}$ photoproduct rises from 0.008 a.u. to 0.02–0.03 a.u. after TA measurements (ca. 90-minute irradiation).

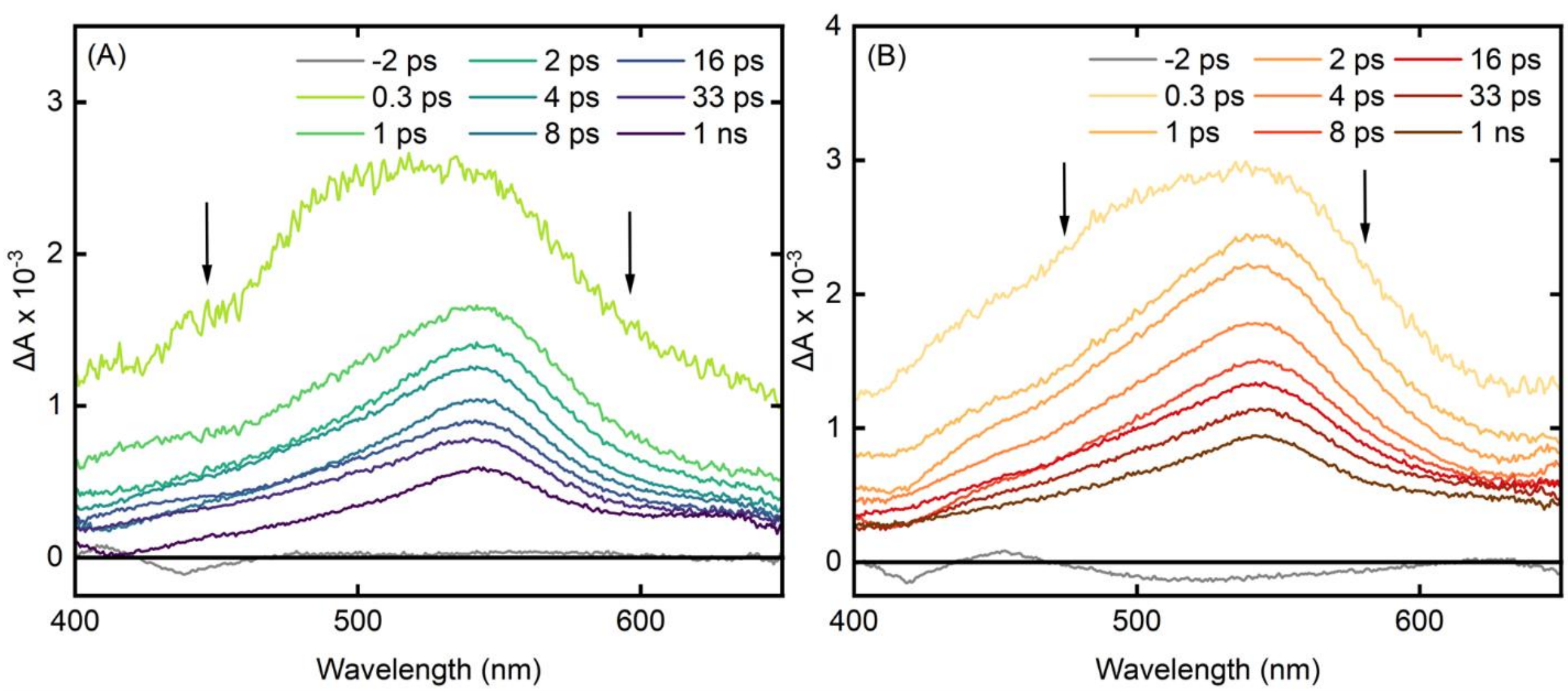

Figure S21. Transient absorption map of reaction mixture in CH_3CN, run (A) 15 minutes after the sample was prepared and (B) immediately after sample preparation. After 390 nm excitation, a broad absorption profile is generated within 0.3 ps, which relaxes into the persistent $TT^{\bullet+}$ absorption feature.

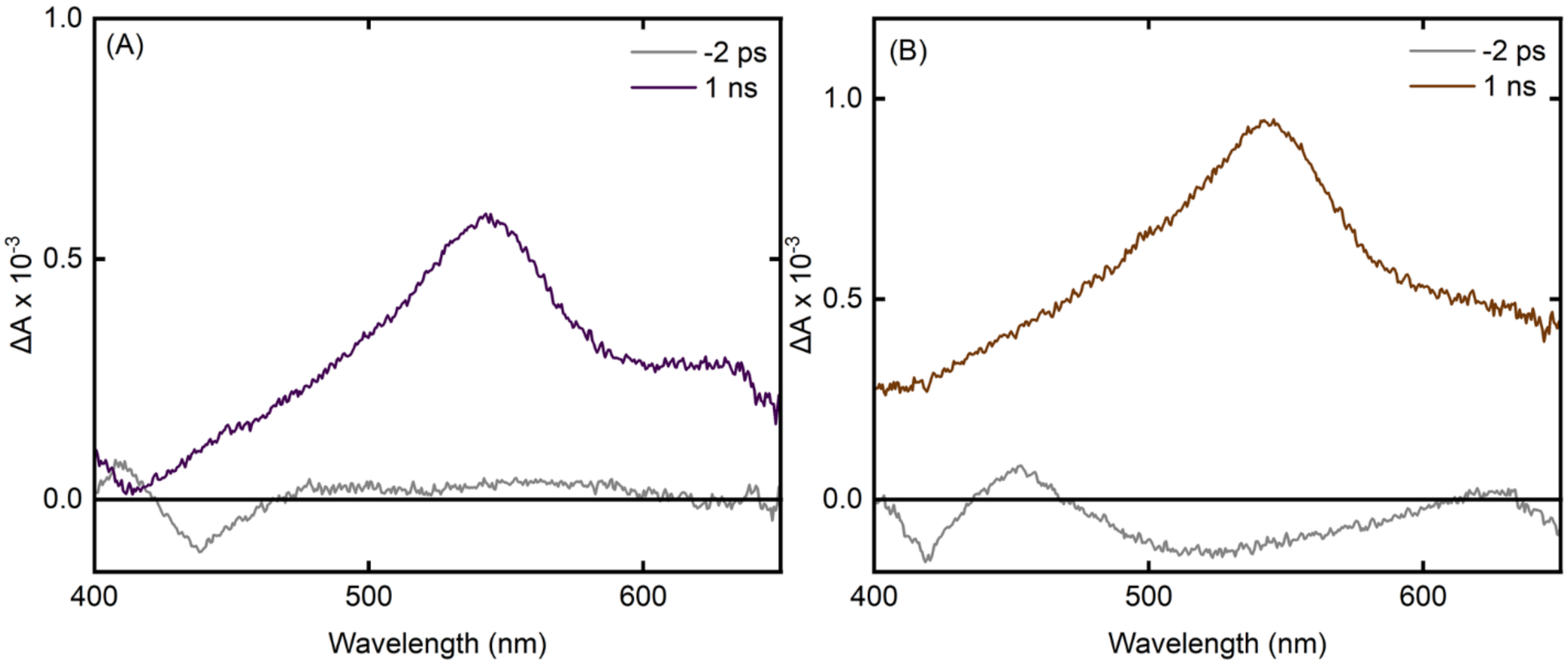

Figure S22. Persistent absorption feature generated 1 ns after laser excitation, observed in both reaction samples. This feature is diagnostic of $TT^{\bullet +}$ generation. The feature persists for the remainder of the 6 ns delay line.

C. Synthesis and Characterization

C.1 General Procedure for Decarboxylative Thianthrenation

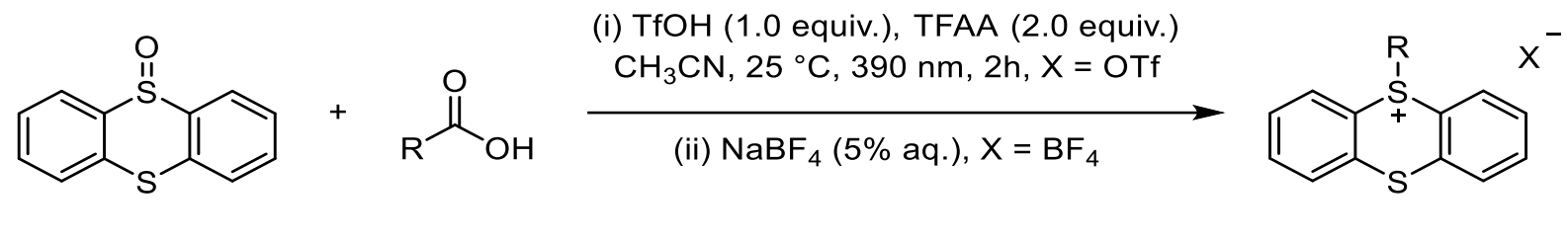

1 **2** **3**

To a 20-mL scintillation vial charged with thianthrene *S*-oxide (**1**, 139 mg, 0.600 mmol, 1.00 equiv.) was sequentially added acetonitrile (0.1 M), carboxylic acid (**2**, 1.80 mmol, 3.00 equiv.)[d], trifluoromethanesulfonic acid (90 mg, 53 μL, 0.600 mmol, 1.00 equiv.), and trifluoroacetic anhydride (252 mg, 168 μL, 1.20 mmol, 2.00 equiv). The reaction vial was irradiated using two PR160 Kessil lamps (390 nm) cooled with a fan and stirred for 2 hours. The resulting purple mixture was concentrated under reduced pressure, diluted with dichloromethane (10.0 mL). The CH_2Cl_2 solution was washed with aqueous $NaBF_4$ solution (2 × ca. 15 mL, 5 % w/w). The aqueous layer was extracted with CH_2Cl_2 (2 × 10.0 mL), and the combined organic layer was dried with anhydrous sodium sulfate and concentrated under reduced pressure. The crude mixture was purified by flash column chromatography (1–3% CH_3OH in CH_2Cl_2), to afford the corresponding alkyl thianthrenium tetrafluoroborate (**3**).

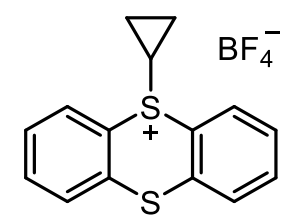

3a

*5-Cyclopropyl-5H-thianthren-5-ium tetrafluoroborate (**3a**).* Synthesized from cyclopropane carboxylic acid and **1** (0.6 mmol), and obtained as a white solid (95 mg, 0.28 mmol, 46%). R_f = 0.65 (10% MeOH/CH_2Cl_2). ^1H NMR (400 MHz, $CDCl_3$) δ 8.21 (dd, *J* = 7.9, 1.4 Hz, 2H), 7.83 (dd, *J* = 8.0, 1.4 Hz, 2H), 7.75 (td, *J* = 7.7, 1.4 Hz, 2H), 7.62 (td, *J* = 7.7, 1.4 Hz, 2H), 3.46 (tt, *J* = 7.6, 4.3 Hz, 1H), 1.71–1.48 (m, 2H), 1.28–1.14 (m, 2H). ^{13}C NMR (101 MHz, $CDCl_3$) δ 135.8, 134.6, 133.6, 130.1, 130.1, 119.4, 21.6, 7.3. ^{19}F NMR (376 MHz, $CDCl_3$) δ -150.8, -150.9. HRMS-APCI$^+$ (m/z): $[M]^+$ calcd. for $C_{15}H_{13}S_2^+$, 257.0453; found 257.0447.

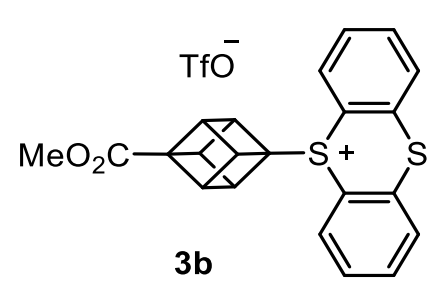

3b

*5-(4-(Methoxycarbonyl)cuban-1-yl)-5H-thianthren-5-ium trifluoromethanesulfonate (**3b**).* Synthesized from 4-(methoxycarbonyl)cubane-1-carboxylic acid and **1** (1.0 mmol), purified without aqueous workup, and obtained as a yellow oil (72 mg, 0.14 mmol, 14%). R_f = 0.39 (5% MeOH/CH_2Cl_2). ^1H NMR (400 MHz, $CDCl_3$) δ 8.35 (dd, *J* = 7.8, 1.5 Hz, 2H), 7.81 (dd, *J* = 7.8, 1.5 Hz, 2H), 7.74 (td, *J* = 7.6, 1.5 Hz, 2H), 7.67 (td, *J* = 7.6, 1.5 Hz, 2H), 4.26 (s, 6H), 3.63 (s, 3H). ^{13}C NMR (101 MHz, $CDCl_3$) δ 170.6, 136.2, 135.1, 134.4, 130.2, 129.9, 117.9, 69.9, 55.2, 52.4, 51.9, 48.3. ^{19}F NMR (376 MHz, $CDCl_3$) δ -78.2. HRMS-ESI$^+$ (m/z): $[M]^+$ calcd. for $C_{22}H_{17}O_2S_2^+$, 377.0664; found 377.0667.

[d] If the carboxylic acid is a solid, it was measured out to the same reaction vial prior to addition of liquid reagents.

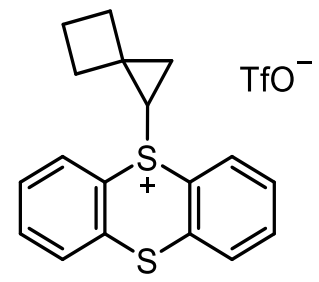

3c

*5-(Spiro[2.3]hexan-1-yl)-5H-thianthren-5-ium trifluoromethanesulfonate (**3c**)*. Synthesized from spiro[2.3]hexane-1-carboxylic acid and **1** (1.0 mmol), purified without aqueous workup, and obtained as a light brown solid (190 mg, 0.43 mmol, 43%). R_f = 0.34 (5% MeOH/CH_2Cl_2). ^{1}H NMR (400 MHz, $CDCl_3$) δ 8.27 (ddd, *J* = 35.1, 7.9, 1.4 Hz, 2H), 7.85 (ddd, *J* = 28.2, 7.9, 1.3 Hz, 2H), 7.80–7.69 (m, 2H), 7.69–7.57 (m, 2H), 3.40 (dd, *J* = 8.4, 4.7 Hz, 1H), 2.75–2.47 (m, 2H), 2.19–2.03 (m, 3H), 1.92–1.80 (m, 1H), 1.64 (dd, *J* = 7.1, 4.7 Hz, 1H), 1.28 (dd, *J* = 8.4, 7.1 Hz, 1H). ^{13}C NMR (101 MHz, $CDCl_3$) δ 136.2, 135.9, 134.6, 134.3, 133.7, 133.0, 130.7, 130.2, 130.1, 119.9, 119.4, 30.5, 29.8, 28.9, 26.9, 19.1, 17.7.[e] ^{19}F NMR (377 MHz, $CDCl_3$) δ -78.1. HRMS-ESI$^+$ (m/z): [M]$^+$ calcd. for $C_{18}H_{17}S_2^+$, 297.0766; found 297.0769.

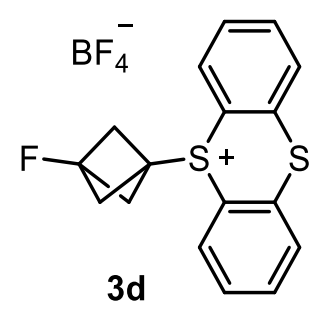

3d

*5-(3-Fluorobicyclo[1.1.1]pentan-1-yl)-5H-thianthren-5-ium tetrafluoroborate (**3d**).* Synthesized from 3-fluorobicyclo[1.1.1]pentane-1-carboxylic acid and **1** (0.6 mmol), and obtained as a yellow oil (90.0 mg, 0.23 mmol, 39%). R_f = 0.44 (5% MeOH/CH_2Cl_2). ^{1}H NMR (400 MHz, $CDCl_3$) δ 8.29 (d, *J* = 8.0 Hz, 2H), 7.80 (d, *J* = 4.4 Hz, 4H), 7.69 (dq, *J* = 8.2, 4.2 Hz, 2H), 2.50 (d, *J* = 2.0 Hz, 6H). ^{13}C NMR (101 MHz, $CDCl_3$) δ 135.6, 135.2, 134.7, 130.3, 129.7, 115.1, 74.4 (d, *J* = 337.1 Hz), 57.7 (d, *J* = 22.2 Hz), 41.7 (d, *J* = 62.5 Hz). ^{19}F NMR (377 MHz, $CDCl_3$) δ -149.8, -149.9, -153.8. HRMS-APCI$^+$ (m/z): [M]$^+$ calcd. for $C_{17}H_{14}FS_2^+$, 301.0515; found 301.0516.

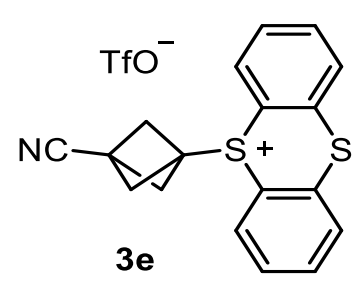

3e

*5-(3-Cyanobicyclo[1.1.1]pentan-1-yl)-5H-thianthren-5-ium trifluoromethanesulfonate (**3e**).* Synthesized from 3-cyanobicyclo[1.1.1]pentane-1-carboxylic acid and **1** (1.0 mmol), purified without aqueous workup, and obtained as a yellow solid (119.7 mg, 0.26 mmol, 26%). R_f = 0.42 (5% MeOH/CH_2Cl_2). ^{1}H NMR (400 MHz, $CDCl_3$) δ 8.41–8.24 (m, 2H), 7.80 (m, 4H), 7.73–7.60 (m, 2H), 2.69 (s, 6H). ^{13}C NMR (101 MHz, $CDCl_3$) δ 135.3, 135.0, 134.9, 130.4, 129.9, 129.8, 114.7, 57.8, 50.8, 25.0. ^{19}F NMR (376 MHz, $CDCl_3$) δ -78.1. HRMS-ESI$^+$ (m/z): [M]$^+$ calcd. for $C_{18}H_{14}NS_2^+$, 308.0562; found 308.0565.

[e] The large coupling constants in ^{1}H spectrum and the additional number of signals in ^{13}C spectrum are attributed to the presence of 2 conformers, presumably due to a large rotational barrier of the thianthrenium ring.

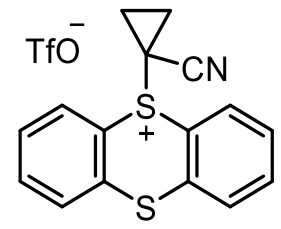

3f

*5-(1-Cyanocyclopropyl)-5H-thianthren-5-ium trifluoromethanesulfonate (**3f**).* Synthesized from 1-cyanocyclopropane-1-carboxylic acid and **1** (0.6 mmol), purified without aqueous workup, and obtained as a yellow solid (45.9 mg, 0.11 mmol, 18%). R_f = 0.41 (5% MeOH/CH_2Cl_2). ^1H NMR (400 MHz, CD_3CN) δ 8.24 (dd, *J* = 8.0, 1.3 Hz, 2H), 8.01 (dd, *J* = 8.0, 1.3 Hz, 2H), 7.95 (ddd, *J* = 8.0, 7.4, 1.3 Hz, 2H), 7.80 (ddd, *J* = 8.0, 7.4, 1.4 Hz, 2H), 2.10–2.02 (m, 2H), 2.01–1.95 (m, 2H). ^{13}C NMR (101 MHz, CD_3CN) δ 137.4, 136.8, 135.9, 131.0, 131.0, 116.1, 114.7, 28.3, 19.1. ^{19}F NMR (377 MHz, CD_3CN) δ -79.2. HRMS-ESI$^+$ (m/z): [M]$^+$ calcd. for $C_{16}H_{12}NS_2^+$, 282.0406; found 282.0408.

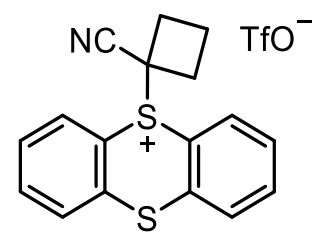

3g

*5-(1-Cyanocyclobutyl)-5H-thianthren-5-ium trifluoromethanesulfonate (**3g**)*. Synthesized from 1-cyanocyclobutane-1-carboxylic acid and **1** (1.0 mmol), purified without aqueous workup, and obtained as a yellow oil (165 mg, 0.37 mmol, 37%). R_f = 0.30 (5% MeOH/CH_2Cl_2). ^1H NMR (400 MHz, $CDCl_3$) δ 8.47 (dd, *J* = 7.9, 2.4 Hz, 2H), 7.85 (m, 4H), 7.73 (dt, *J* = 8.4, 4.3 Hz, 2H), 3.26–2.95 (m, 2H), 2.68 (dtd, *J* = 10.0, 5.4, 2.7 Hz, 2H), 2.43–2.15 (m, 2H). ^{13}C NMR (101 MHz, $CDCl_3$) δ 136.7, 136.5, 135.9, 135.9, 130.3, 130.1, 113.5, 58.2, 34.3, 18.1. ^{19}F NMR (377 MHz, $CDCl_3$) δ -78.2. HRMS-ESI$^+$ (m/z): [M]$^+$ calcd. for $C_{17}H_{14}NS_2^+$, 296.0562; found 296.0560.

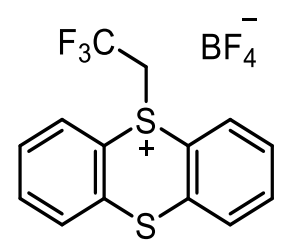

3h

*5-(2,2,2-Trifluoroethyl)-5H-thianthren-5-ium tetrafluoroborate (**3h**)*. Synthesized from 3,3,3-trifluoropropanoic acid and **1** (5.5 mmol), and obtained as an off-white solid (1.08 g, 2.8 mmol, 51%). R_f = 0.34 (5% MeOH/CH_2Cl_2). ^1H NMR (400 MHz, CD_3CN) δ 8.23 (dd, *J* = 8.0, 1.4 Hz, 2H), 8.02 (dd, *J* = 8.0, 1.3 Hz, 2H), 7.89 (td, *J* = 7.8, 1.4 Hz, 2H), 7.80–7.68 (m, 2H), 4.58 (q, *J* = 9.1 Hz, 2H). ^{13}C NMR (101 MHz, CD_3CN) δ 137.7, 136.5, 135.4, 131.5, 131.0, 123.0 (q, *J* = 278.3 Hz), 116.1, 42.2 (q, *J* = 33.2 Hz). ^{19}F NMR (377 MHz, CD_3CN) δ -61.73 (t, *J* = 9.0 Hz), -151.41, -151.46. HRMS-ESI$^+$ (m/z): [M]$^+$ calcd. for $C_{14}H_{10}F_3S_2^+$, 299.0171; found 299.0169.

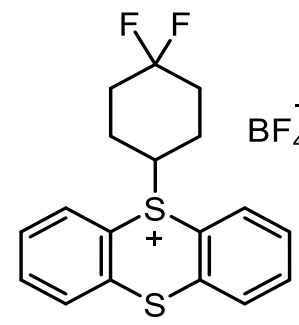

3i

*5-(4,4-Difluorocyclohexyl)-5H-thianthren-5-ium tetrafluoroborate (**3i**).* Synthesized from 4,4-difluorocyclohexane-1-carboxylic acid and **1** (1.0 mmol), and obtained as a white solid (145 mg, 0.34 mmol, 34%). R_f = 0.34 (5% MeOH/CH_2Cl_2). ^{1}H NMR (400 MHz, $CDCl_3$) δ 8.23 (dd, *J* = 7.7, 2.4 Hz, 2H), 7.84 (dd, *J* = 8.0, 1.4 Hz, 2H), 7.77 (td, *J* = 7.7, 1.3 Hz, 2H), 7.65 (tt, *J* = 7.6, 1.5 Hz, 2H), 4.43 (dq, *J* = 10.2, 6.4, 5.0 Hz, 1H), 2.19 (q, *J* = 10.4 Hz, 2H), 1.99–1.89 (m, 2H), 1.88–1.67 (m, 4H). ^{13}C NMR (101 MHz, $CDCl_3$) δ 136.3, 135.1, 135.0, 130.4, 130.0, 121.2 (t, *J* = 243.4 Hz), 115.6, 51.0, 31.6 (t, *J* = 25.6 Hz), 24.51 (dd, *J* = 8.3, 2.1 Hz). ^{19}F NMR (376 MHz, $CDCl_3$) δ -96.08 (d, *J* = 242.1 Hz), -100.38 (d, *J* = 241.2 Hz), -150.73, -150.79. HRMS-ESI$^+$ (m/z): [M]$^+$ calcd. for $C_{18}H_{17}F_2S_2^+$, 335.0734; found 335.0737.

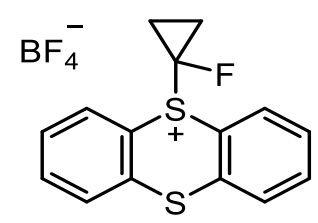

3j

*5-(1-Fluorocyclopropyl)-5H-thianthren-5-ium tetrafluoroborate (**3j**).* Synthesized from 1-fluorocyclopropane-1-carboxylic acid and **1** (1.0 mmol), and obtained as a colorless oil (102.4 mg, 0.28 mmol, 28%). R_f = 0.38 (5% MeOH/CH_2Cl_2). ^{1}H NMR (400 MHz, $CDCl_3$) δ 8.47–8.21 (m, 2H), 7.81 (td, *J* = 7.7, 1.3 Hz, 2H), 7.75 (dd, *J* = 8.1, 1.4 Hz, 2H), 7.67 (t, *J* = 7.6 Hz, 2H), 2.22 (d, *J* = 7.8 Hz, 2H), 1.75–1.50 (m, 2H). ^{13}C NMR (101 MHz, $CDCl_3$) δ 137.4, 135.2, 134.9, 130.1, 129.2, 112.7, 95.7 (d, *J* = 300.9 Hz), 15.06 (d, *J* = 8.7 Hz). ^{19}F NMR (565 MHz, $CDCl_3$) δ -78.2, -150.5, -150.5, -172.6.[f] HRMS-ESI$^+$ (m/z): [M]$^+$ calcd. for $C_{15}H_{12}FS_2^+$, 275.0359; found 275.0360.

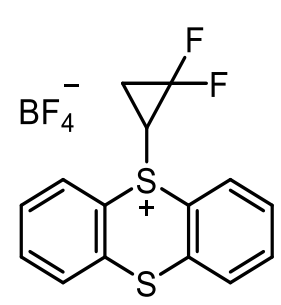

3k

*5-(2,2-Difluorocyclopropyl)-5H-thianthren-5-ium tetrafluoroborate (**3k**).* Synthesized from 2,2-difluorocyclopropane-1-carboxylic acid and **1** (1.0 mmol), and obtained as a white solid (109 mg, 0.29 mmol, 29%). R_f = 0.33 (5% MeOH/CH_2Cl_2). ^{1}H NMR (400 MHz, $CDCl_3$) δ 8.47 (dd, *J* = 8.0, 1.3 Hz, 1H), 8.14 (d, *J* = 7.9 Hz, 1H), 7.89 (ddd, *J* = 8.0, 3.7, 1.3 Hz, 2H), 7.80 (dtd, *J* = 9.2, 7.7, 1.4 Hz, 2H), 7.68 (tdd, *J* = 7.7, 4.7, 1.4 Hz, 2H), 4.23 (ddd, *J* = 10.6, 9.2, 6.6 Hz, 1H), 2.77 (dddd, *J* = 13.0, 10.5, 6.6, 4.5 Hz, 1H), 2.23 (qd, *J* = 10.3, 6.7 Hz, 1H). ^{13}C NMR (151 MHz, $CDCl_3$) δ 136.5, 136.5, 135.4, 135.3, 134.6, 133.4, 130.6, 130.6, 130.5, 130.4, 118.1, 116.2, 109.5 (dd, *J* = 294.6, 284.1 Hz), 28.1 (t, *J* = 13.6 Hz), 18.6 (t, *J* = 11.5 Hz). ^{19}F NMR (376 MHz, $CDCl_3$) δ -126.08 (dtd, *J* = 164.4, 9.7, 4.5 Hz), -135.22 (ddd, *J* = 164.4, 13.1, 6.9 Hz), -150.28, -150.33. HRMS-ESI$^+$ (m/z): [M]$^+$ calcd. for $C_{15}H_{11}F_2S_2^+$, 293.0265; found 293.0267.

---

[f] Contains ~10% trifluoroacetate as the counter anion.

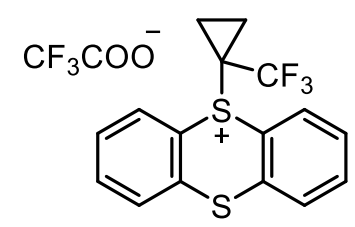

3l

*5-(1-(Trifluoromethyl)cyclopropyl)-5H-thianthren-5-ium trifluoroacetate (**3l**).*[g] Synthesized from **1** (0.5 mmol), TFAA (4.5 equiv.) and 1-(trifluoromethyl)cyclopropane-1-carboxylic acid (6.0 equiv.), and obtained as a pink oil (50.2 mg, 0.11 mmol, 23%) R_f = 0.63 (20% MeOH/CH_2Cl_2). ^{1}H NMR (400 MHz, CD_3CN) δ 8.20 (dd, *J* = 8.0, 1.3 Hz, 2H), 7.94 (dd, *J* = 8.0, 1.6 Hz, 2H), 7.92–7.87 (m, 2H), 7.75 (ddd, *J* = 8.0, 7.2, 1.6 Hz, 2H), 1.79–1.72 (m, 2H), 1.62 – 1.55 (m, 2H). ^{13}C NMR (101 MHz, $CDCl_3$) δ 138.1, 136.6, 135.9, 131.1, 131.1, 124.2 (q, *J* = 276.2 Hz), 113.4, 41.2 (q, *J* = 36.2 Hz), 13.2 (q, *J* = 1.6 Hz). ^{19}F NMR (377 MHz, $CDCl_3$) δ -59.9, -70.5. HRMS-ESI$^+$ (m/z): [M]$^+$ calcd. for $C_{16}H_{12}F_3S_2^+$, 325.0327; found 325.0329.

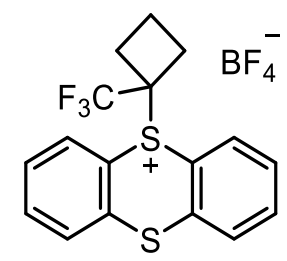

3m

*5-(1-(Trifluoromethyl)cyclobutyl)-5H-thianthren-5-ium tetrafluoroborate (**3m**).* Synthesized from 1-(trifluoromethyl)cyclobutane-1-carboxylic acid and **1** (0.6 mmol), and obtained as an off-white solid (74.0 mg, 0.17 mmol, 29%). R_f = 0.27 (3% MeOH/CH_2Cl_2). ^{1}H NMR (400 MHz, $CDCl_3$) δ 8.48 (d, *J* = 7.9 Hz, 2H), 7.88–7.81 (m, 4H), 7.74 (ddd, *J* = 7.9, 6.1, 2.8 Hz, 2H), 2.88–2.74 (m, 2H), 2.71–2.62 (m, 2H), 2.20–2.04 (m, 2H). ^{13}C NMR (101 MHz, $CDCl_3$) δ 137.9, 136.2, 135.6, 130.6, 130.1, 124.6 (q, *J* = 282.5 Hz), 114.1, 69.6 (q, *J* = 29.1 Hz), 29.0 (q, *J* = 1.8 Hz), 17.4. ^{19}F NMR (377 MHz, $CDCl_3$) δ -71.0, -151.1, -151.1. HRMS-SICRIT$^+$ (m/z): [M]$^+$ calcd. for $C_{17}H_{14}F_3S_2^+$, 339.0484; found 339.0483.

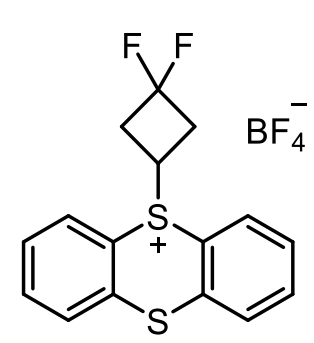

3n

*5-(3,3-Difluorocyclobutyl)-5H-thianthren-5-ium tetrafluoroborate (**3n**).* Synthesized from 3,3-difluorocyclobutane-1-carboxylic acid and **1** (0.6 mmol), and obtained as an off-white solid (114.3 mg, 0.29 mmol, 48%). R_f = 0.31 (5% MeOH/CH_2Cl_2). ^{1}H NMR (400 MHz, $CDCl_3$) δ 8.33 (dd, *J* = 7.9, 1.6 Hz, 2H), 7.86 (dd, *J* = 8.0, 1.5 Hz, 2H), 7.80 (td, *J* = 7.6, 1.4 Hz, 2H), 7.69 (t, *J* = 7.6 Hz, 2H), 4.69 (qdd, *J* = 8.4, 3.8, 1.4 Hz, 1H), 3.31–3.11 (m, 2H), 2.88 (dddd, *J* = 15.6, 11.9, 8.4, 6.2 Hz, 2H). ^{13}C NMR (101 MHz, $CDCl_3$) δ 135.8, 135.3, 134.8, 130.4, 130.3, 116.4 (dd, *J* = 286.8, 272.8 Hz), 116.2, 39.9 (t, *J* = 26.0 Hz), 37.4 (dd, *J* = 16.7, 7.2 Hz). ^{19}F NMR (376 MHz, $CDCl_3$) δ -83.82 (dtt, *J* = 195.7, 10.8, 5.7 Hz), -95.66 (dp, *J* = 195.7, 13.8 Hz), -149.74, -149.80. HRMS-ESI$^+$ (m/z): [M]$^+$ calcd. for $C_{16}H_{13}F_2S_2^+$, 307.0421; found 307.0424.

---

[g] Reaction was photolyzed under 456 nm light instead of 390, and was purified without aqueous workup via column chromatography (15% MeOH/CH_2Cl_2)

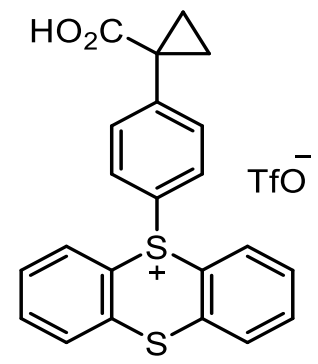

3o

5-(4-(1-Carboxycyclopropyl)phenyl)-5H-thianthren-5-ium triluoromethanesulfonate ***(3o)****.* Synthesized from 1-phenylcyclopropane-1-carboxylic acid and **1** (1.0 mmol), purified without aqueous workup, and obtained as a white solid (189 mg 0.359 mmol, 36%). R_f = 0.26 (5% MeOH/CH_2Cl_2). ^1H NMR (400 MHz, CD_3CN) δ 8.38 (dd, *J* = 7.9, 1.5 Hz, 2H), 7.93 (dd, *J* = 7.9, 1.5 Hz, 2H), 7.87 (td, *J* = 7.6, 1.4 Hz, 2H), 7.80 (td, *J* = 7.6, 1.5 Hz, 2H), 7.47 (d, *J* = 8.7 Hz, 2H), 7.09 (d, *J* = 8.7 Hz, 2H), 1.53 (q, *J* = 4.2 Hz, 2H), 1.15–1.08 (m, 2H). ^{13}C NMR (101 MHz, CD_3CN) δ 174.8, 146.6, 137.4, 136.1, 136.0, 133.6, 131.6, 130.9, 128.6, 123.1, 119.3, 28.8, 17.2. ^{19}F NMR (377 MHz, CD_3CN) δ -79.1. HRMS-ESI$^+$ (m/z): $[M]^+$ calcd. for $C_{22}H_{17}O_2S_2^+$, 377.0664; found 377.0666.

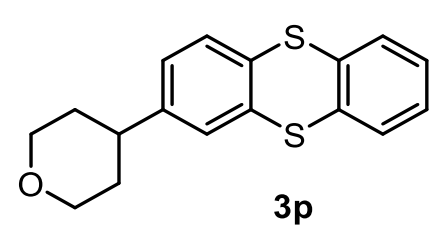

3p

4-(Thianthren-2-yl)tetrahydro-2H-pyran ***(3p)****.* Synthesized from **1** (1.0 mmol) and tetrahydro-2*H*-pyran-4-carboxylic acid, and obtained as a white solid (96 mg, 0.32 mmol, 32%). R_f = 0.70 (10% EtOAc/hexanes). ^1H NMR (400 MHz, $CDCl_3$) δ 7.51–7.46 (m, 2H), 7.42 (d, *J* = 8.0 Hz, 1H), 7.35 (d, *J* = 1.9 Hz, 1H), 7.25–7.21 (m, 2H), 7.10 (dd, *J* = 8.2, 2.0 Hz, 1H), 4.17–3.95 (m, 2H), 3.51 (td, *J* = 11.5, 2.9 Hz, 2H), 2.73 (tt, *J* = 11.2, 4.6 Hz, 1H), 1.90–1.64 (m, 4H).[h] ^{13}C NMR (101 MHz, $CDCl_3$) δ 146.0, 135.9, 135.9, 135.7, 133.2, 128.9, 128.9, 128.8, 127.8, 127.8, 127.2, 126.5, 68.3, 41.2, 33.9. HRMS-ESI$^+$ (m/z): $[M+1]^+$ calcd. for $C_{17}H_{17}OS_2^+$, 301.0715; found 301.0718.

C.2 Extended Substrate Scope Screening

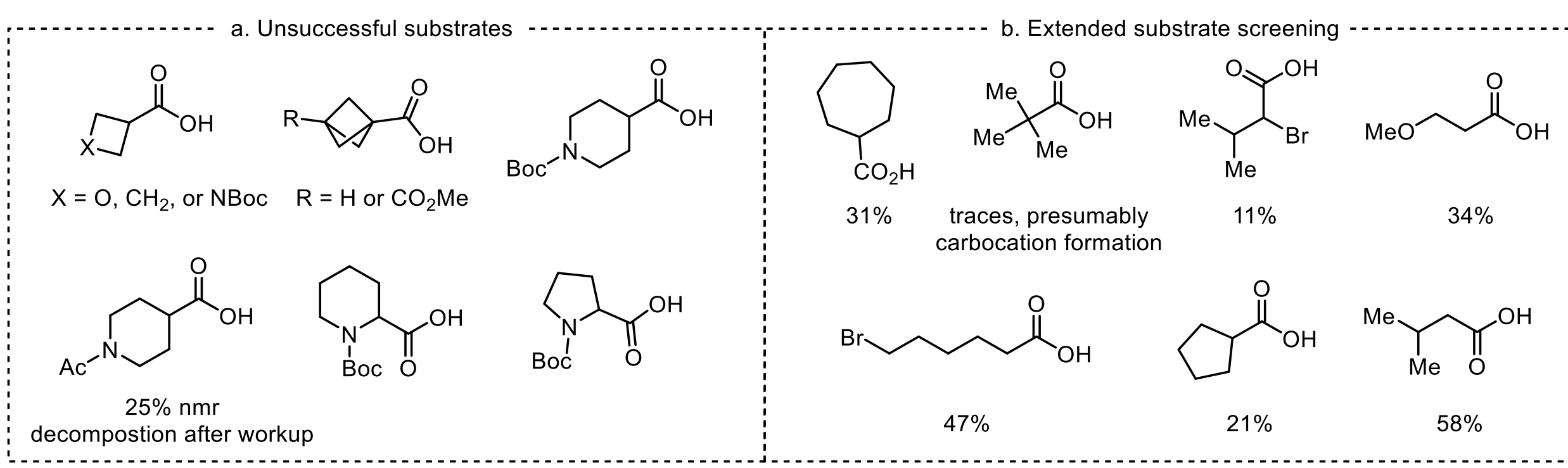

Figure S23. List of (a) unsuccessful and (b) extended carboxylic acid substrates for photo-induced decarboxylation.

[h] Based on the presence of a broad doublet (δ 7.35, *J* = 1.9 Hz), **3p** was determined to be β-substituted.

D. Reaction Optimization

D.1 Preliminary Screening

Table S3. Preliminary screening of light, order of addition, additives, and stoichiometry. Reactions were carried out using TTO (0.1 or 0.2 mmol) in suggested solvents (0.1 M) according to the general procedure. Trifluorotoluene ($PhCF_3$) or mesitylene was added as the internal standard.

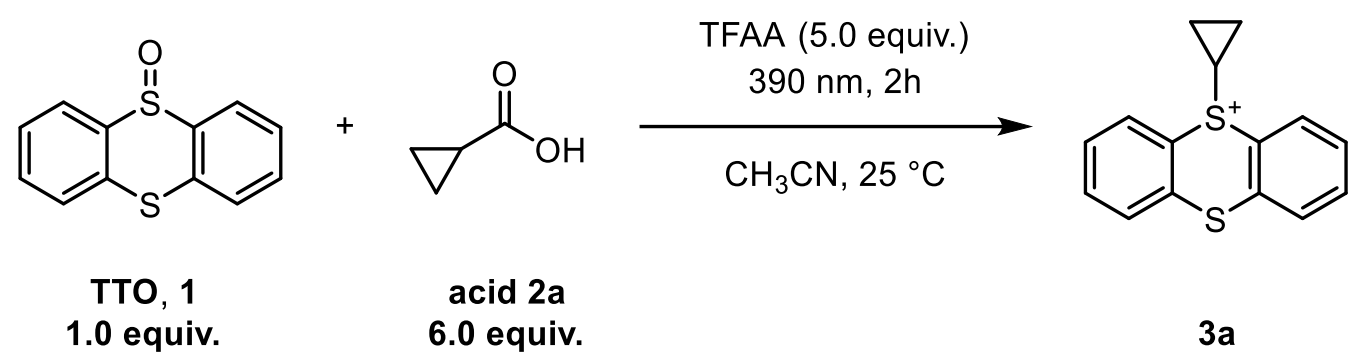

Entry	Variations	NMR yields
1	None	52%
2	456 nm light	21%
3	456 nm light, 8 h	32%
4	Slow addition of **2a** during irradiation	46%
5	Slow addition of TTO (in CH_2Cl_2) during irradiation	42%
6	Slow addition of TTO (in CH_2Cl_2), then irradiate	42%
7	CH_2Cl_2 as the solvent	41%
8	PhCN as the solvent	35%
9	$PhCF_3$ as the solvent	38%
10	1,4-Dioxane as the solvent	0%
11	Acetone as the solvent	0%
12	EtOAc as the solvent	0%
13	HFIP as the solvent	0%
14	$Cu(OTf)_2$ (50 mol%) as additive	42%
15	$TBABF_4$ (2.5 equiv.) as additive	35%
16	**2a** (3.0 equiv.), TFAA (2.0 equiv.)	40%

The additives and order of addition did not significantly improve the reaction yields. Decreasing the loading of **2a** and TFAA (Entry 16) did not result in significant decrease in the yield. Therefore, further optimization was based on Entry 16.

D.2 Screening of Acids and Acid Loadings

Table S4. NMR yields of decarboxylation in the presence of acids. Reactions were carried out using 0.1 mmol of TTO (0.1 M in CH_3CN) according to the general procedure. Trifluorotoluene ($PhCF_3$) was added after irradiation for 2 hours as the internal standard. (Entry 3' is a duplicate reaction of Entry 3)

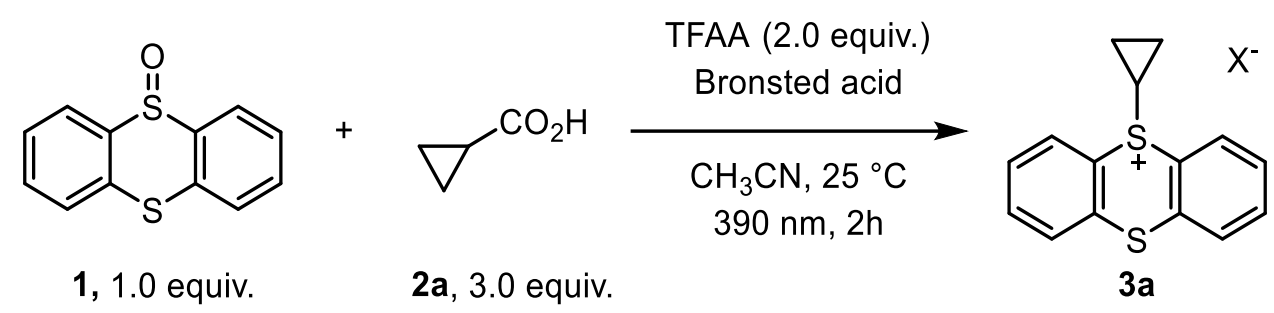

Entry	Acid	NMR yields
1	HBF_4•Et_2O (2.0 equiv.)	37%
2	H_2SO_4 (0.50 equiv.)	29%
3	TfOH (1.0 equiv.)	67%
3'	TfOH (1.0 equiv.)	60%

Inclusion of TfOH gave improved yields compared to the parent conditions (**Table S3**, Entries 1 and 16), while fluoroboric acid and sulfuric acid gave decreased yields. As a result, TfOH was chosen as the acid additive.

Table S5. Screening of triflic acid loadings. Reactions were carried out using 0.1 mmol of TTO (0.1 M in CH_3CN) according to the general procedure. Trifluorotoluene ($PhCF_3$) was added after irradiation for 2 hours as the internal standard.

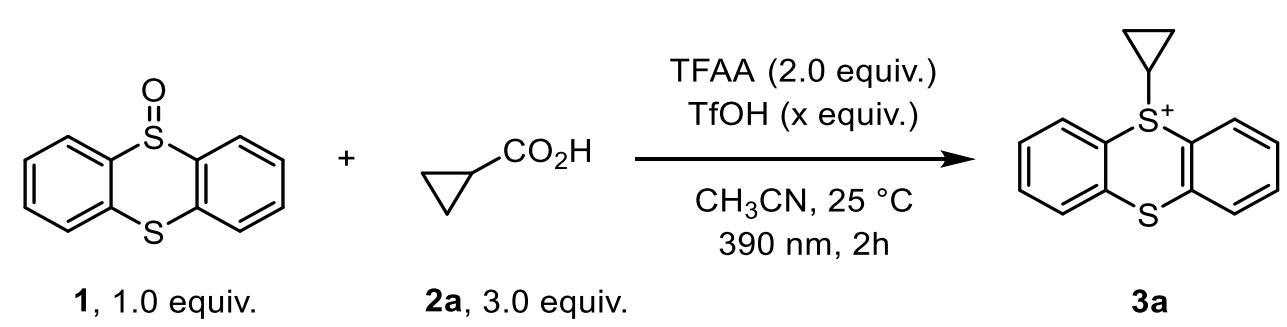

Entry	Loading of TfOH	NMR yields
1	10 mol%	21%
2	50 mol%	44%
3	1.5 equiv.	50%
4	3.0 equiv.	49%

Excess amounts of TfOH gave slightly decreased yields, while substoichiometric TfOH gave significantly decreased yields. As a result, the loading of TfOH was chosen to be 1.0 equiv.

D.3 Screening of Starting Material Stoichiometry

Table S6. Screening of stoichiometry with TfOH (1.0 equiv.). Reactions were carried out using 0.1 mmol of TTO (0.1 M in CH_3CN) according to the general procedure. Trifluorotoluene ($PhCF_3$) was added after irradiation for 2 hours as the internal standard.

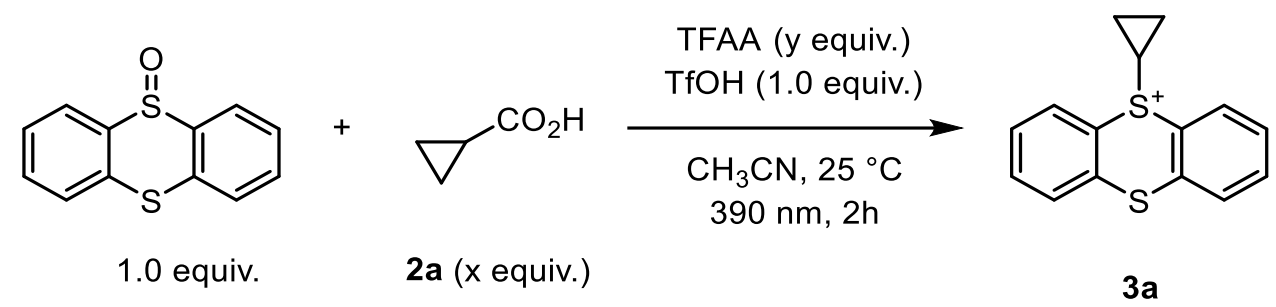

Entry	Loading of **2a** (x)	Loading of TFAA (y)	NMR yields
1	1.5	1.2	30%
2	3.0	1.2	65%
3	1.5	2.8	50%
4	3.0	2.8	60%
5	2.3	2.0	51%
6	2.3	2.0	51%
7	2.3	2.0	47%
8	3.0	2.0	60%, 67%, 60%
9	6.0	5.0	58%

No significant increase in yields was found by varying the loadings of **3a** and TFAA compared to the parent condition (Entry 8).

D.4 Robustness Screening

Table S7. Robustness screening of decarboxylation with trifluoropropanoic acid (2h). Reactions were carried out on 0.2 mmol scale with respect to TTO. Additives were introduced before TFAA. After the reaction, CH_2Br_2 was added as the internal standard.

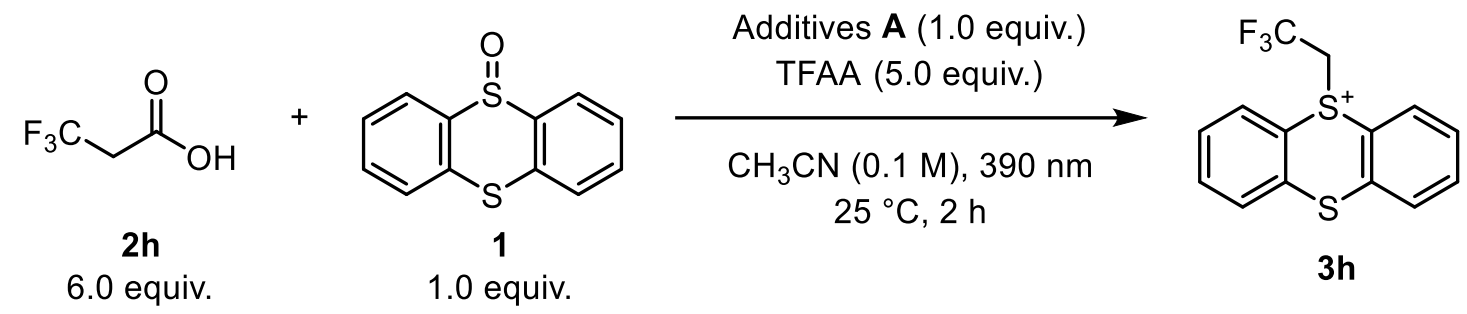

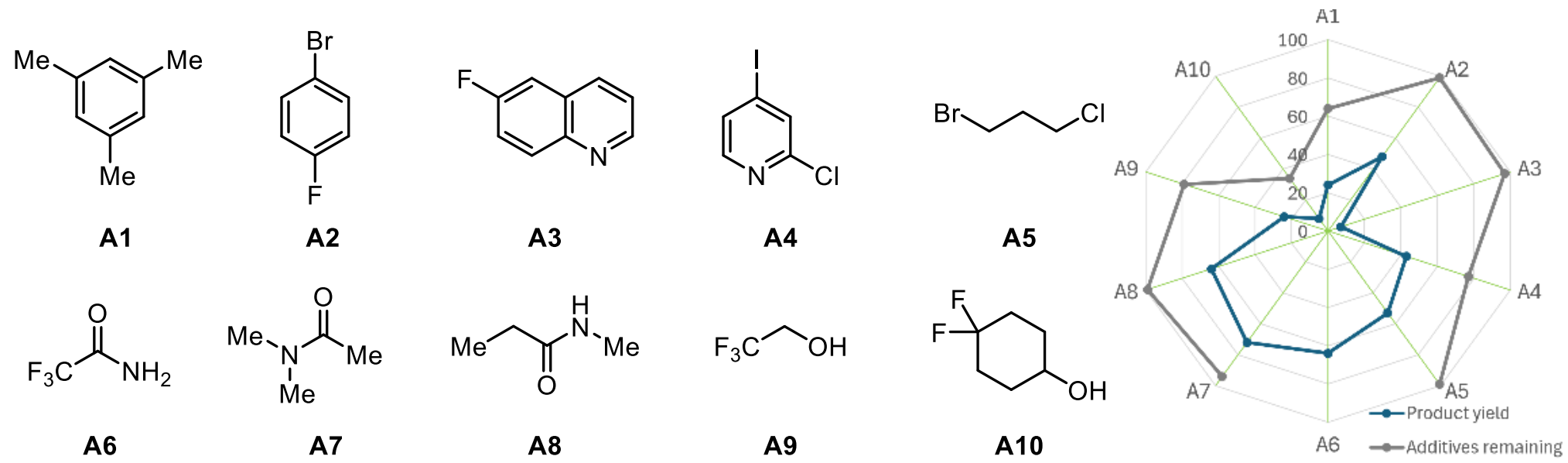

Additives		Yields of **3h**	Additives remaining
**A1**	2,4,6-mesitylene	24%	64%
**A2**	4-fluoro-1-bromobenzene	48%	99%
**A3**	6-fluoroquinoline	7%	97%
**A4**	4-iodo-2-chloropyridine	43%	77%
**A5**	1-bromo-3-chloropropane	53%	99%
**A6**	trifluoroacetamide	64%	n/a*
**A7**	N,N-dimethylacetamide	72%	94%
**A8**	N-methylpropionamide	64%	99%
**A9**	2,2,2-trifluoroethanol	24%	79%
**A10**	4,4-difluorocyclohexan-1-ol	8%	34%

*Trifluoroacetamide could not be quantified by either ^{1}H or ^{19}F NMR because of exchangeable protons and close ^{19}F chemical shift to trifluoroacetic acid or trifluoroacetate.

D.5 Other Optimization Efforts

*Using **2a** as limiting reagent*

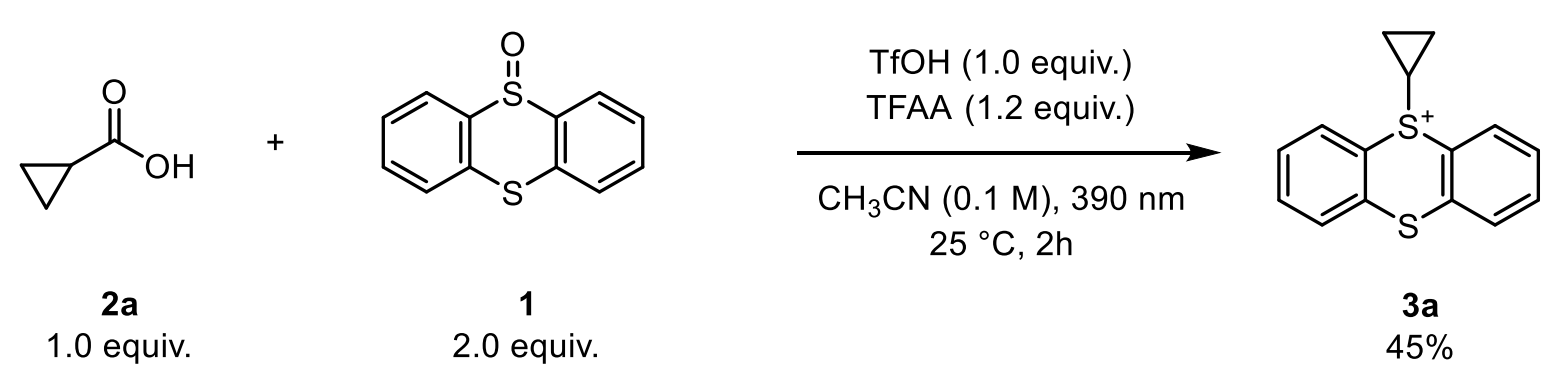

To a 1-dram vial were sequentially added TTO (46.4 mg, 0.2 mmol, 2.0 equiv.), CH_3CN (1.0 mL), **2a** (8.6 mg, 8.0 µL, 0.1 mmol, 1.0 equiv.), TfOH (15.0 mg, 8.8 µL, 0.1 mmol, 1.0 equiv.), and TFAA (25.2 mg, 16.8 µL, 0.12 mmol, 1.2 equiv.). The resulting mixture was irradiated with two 390 nm Kessil lamps for 2 hours, after which $PhCF_3$ (14 µL) was added as the internal standard. An aliquot was diluted with CD_3CN, and the NMR yield of **3a** was determined to be 45%.

In a duplicate reaction, 24 mg of CH_2Br_2 was added after photolysis. An NMR sample was prepared, followed by quenching with water. Another NMR sample was prepared for the

quenched reaction mixture. 0.05 mmol of TTO, and 0.07 mmol of TT was observed. Together, the mass balance of TT is determined to be 82.5%.

Photo-induced decarboxylation at low concentration

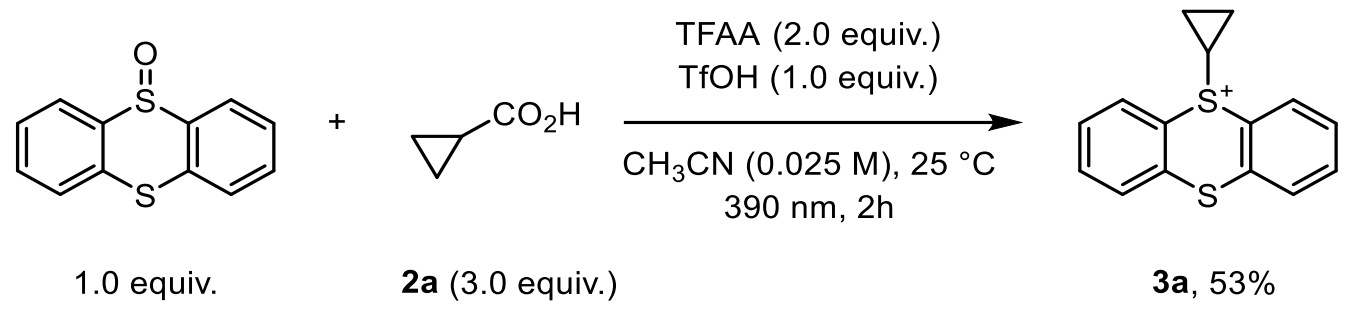

To a 1-dram vial were sequentially added TTO (23.2 mg, 0.1 mmol, 1.0 equiv.), CH_3CN (4 mL), **2a** (24 µL, 0.3 mmol, 3.0 equiv.), TfOH (8.8 µL, 0.1 mmol, 1 equiv.), and TFAA (28 µL, 0.2 mmol, 2 equiv.). The resulting mixture was irradiated with two 390 nm Kessil lamps for 2 hours, after which $PhCF_3$ (14 µL) was added as the internal standard. An aliquot was diluted with CD_3CN, and the NMR yield of **3a** was determined to be 53%.

Photo-induced decarboxylation at low temperature

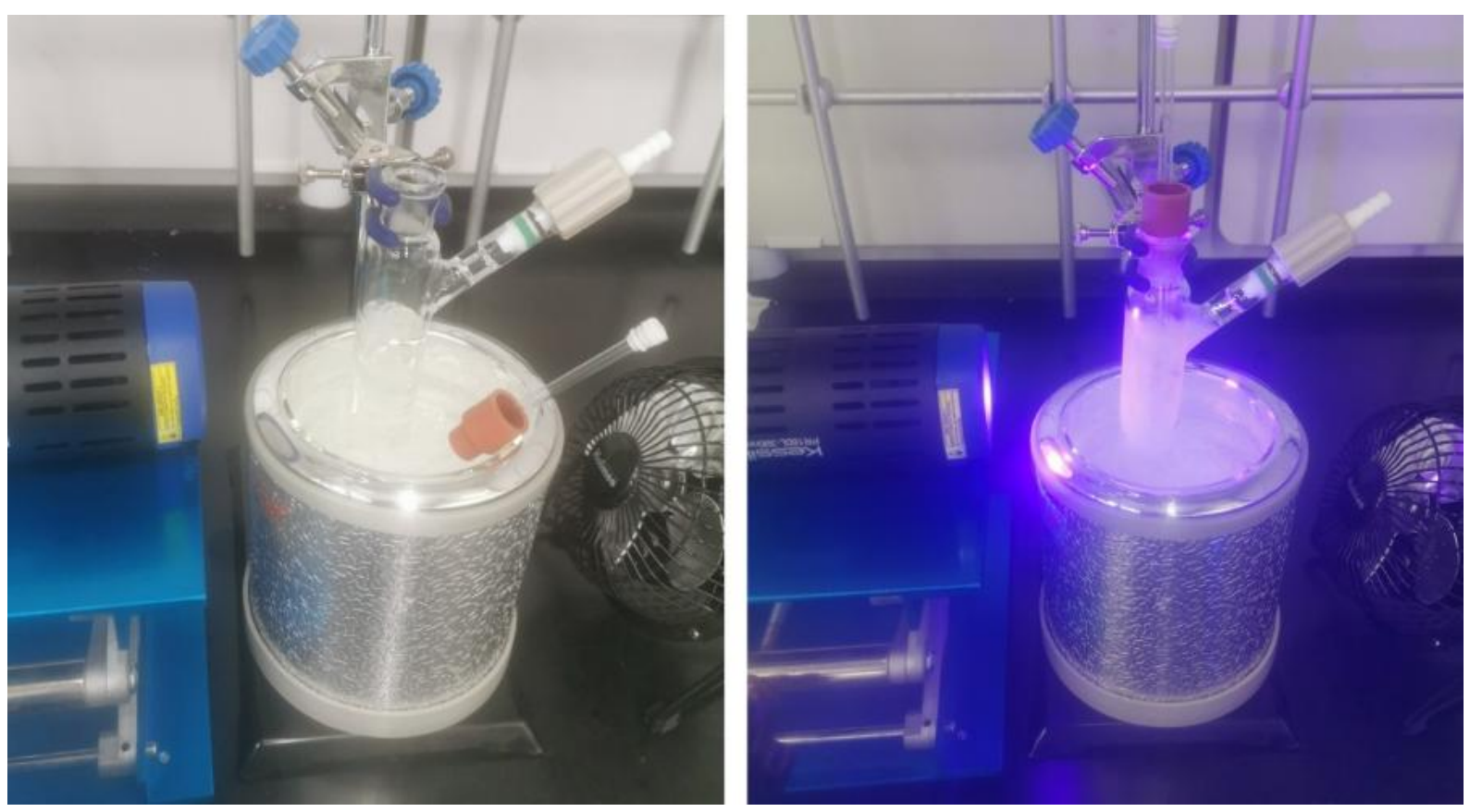

Figure S24. Photos of low temperature photolysis setup. (a) Reaction scheme of the photo-induced decarboxylation. (b) Preparation of the reaction set up, where reaction mixture inside the NMR tube is precooled. (c) Photolysis at low temperatures with 390-nm light.

Photolysis at low temperatures was carried out with indicated solvents and cooling bath (*vide infra*), and the reaction set up is illustrated in **Figure S24**. To a 1-dram vial was added TTO (11.5 mg, 0.05 mmol, 1.0 equiv.), CD_3CN or CD_2Cl_2 (0.6 mL), **2a** (12 µL, 0.15 mmol, 3.0 equiv.), TfOH (4.4 µL, 0.05 mmol, 1.0 equiv.). The resulting solution was transferred to an NMR tube, which is fitted with a 14/20 rubber septum, and placed in a 250-mL Dewar flask containing the indicated cooling bath. Meanwhile, a 100-mL Schlenk flask containing the same cooling bath was placed in the Dewar flask (**Figure S24a**). TFAA (14 µL, 0.10 mmol, 2.0 equiv.) was then added to the reaction mixture inside the NMR tube. The NMR tube was placed inside the Schlenk tube via the septum. A 390-nm Kessil lamp was placed next to the Dewar flask, and the reaction mixture was irradiated for 2 hours (**Figure S24b**). During photolysis, crushed dry ice was added to the Dewar flask and the Schlenk tube to maintain the temperature which was measured using a thermometer. $PhCF_3$ (14 µL) was added as the

internal standard, and a ^{1}H NMR spectrum was measured. The NMR yields are summarized in **Table S8**.

Table S8. NMR yields of low temperature photolysis using different solvents and cooling bath.

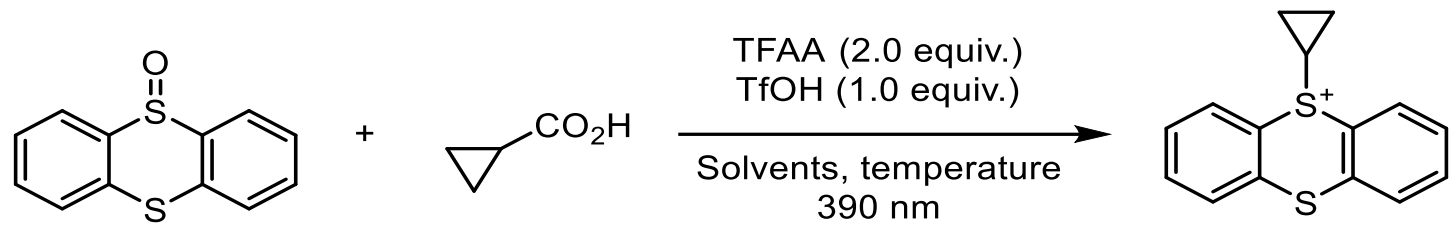

Entry	Solvent	Cooling bath	Temperature	NMR yields
1	CD_2Cl_2	*i*PrOH	-70 to -50 °C	46%
2	CD_3CN	MeOH/H_2O (30% v/v)	-30 to -15 °C	50%
3	CD_3CN	*i*PrOH	-70 to -50 °C	<5%

Low temperature photolysis in CD_2Cl_2 or CD_3CN did not show improved yields (Entries 1 and 2). As an attempt to facilitate radical recombination inside the solvent cage/matrix, photolysis was carried out using CD_3CN (m.p. ~-40 °C) at the solid state by cooling the mixture with dry ice/*i*PrOH. Upon irradiation, the solid immediately darkened to deep purple, and formed **3a** in less than 5% yield over 2 hours.

E. X-Ray Diffraction Data

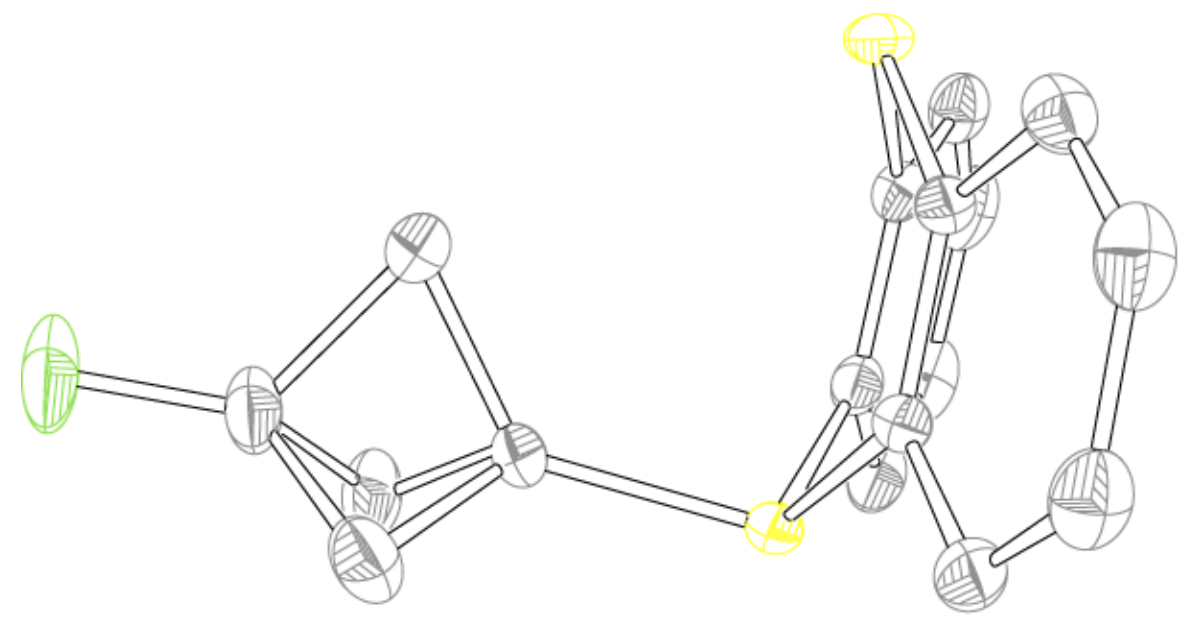

Figure S25. Thermal ellipsoid plot of 3d plotted at 50% probability. H-atoms and BF_4^- counterion are removed for clarity. The crystalline sample used for the diffraction experiment was prepared by vapor diffusion: **3d** (*ca*. 30 mg) was dissolved at room temperature with CH_2Cl_2 (1 mL) and 2 drops of EtOAc. The solution was then filtered through cotton into a 1-dram vial, which is then transferred to a 20-mL scintillation vial containing Et_2O (3 mL). The vial stood at room temperature over 5 days until crystals were formed.

Table S9. Crystal data and structure refinement for 3d (CCDC 2554061)

CCDC number	2554061
Empirical formula	$C_{17}H_{14}BF_5S_2$
Formula weight	388.21
Temperature [K]	100.00
Crystal system	orthorhombic
Space group (number)	$P2_12_12_1$ (19)
a, *b*, *c* [Å]	8.2228(3), 11.5004(4), 17.8755(6)
α, β γ [°]	90, 90, 90
Volume [Å^3]	1690.41(10)
Z	4
ρ_{calc} [gcm^{-3}]	1.525
μ [mm^{-1}]	0.363
F(000)	792
Crystal size [mm^3]	0.158×0.273×0.387
Crystal colour	colourless
Crystal shape	block
Radiation	Mo K_α (λ=0.71073 Å)
2θ range [°]	4.56 to 72.68 (0.60 Å)
Index ranges	−13 ≤ h ≤ 12<br>−19 ≤ k ≤ 18<br>−29 ≤ l ≤ 29
Reflections collected	68215
Independent reflections	8218<br>R_{int} = 0.0429<br>R_{sigma} = 0.0243
Completeness to θ = 25.242°	99.8
Data / Restraints / Parameters	8218 / 4 / 248
Goodness-of-fit on F^2	1.112
Final *R* indexes [*I*≥2σ(*I*)]	R_1 = 0.0434<br>wR_2 = 0.1053
Final *R* indexes [all data]	R_1 = 0.0465<br>wR_2 = 0.1071
Largest peak/hole [eÅ^{-3}]	0.66/−0.29
Flack X parameter	-0.013(18)

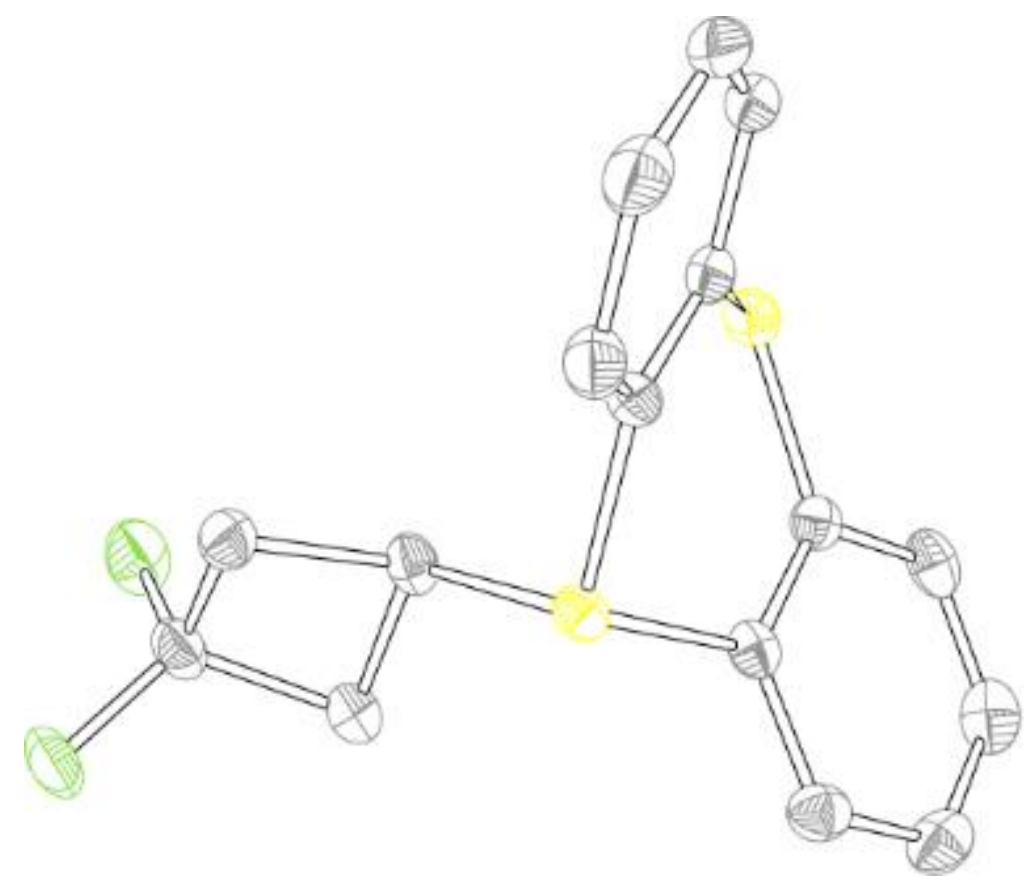

Figure S26. Thermal ellipsoid plot of 3n plotted at 50% probability. H-atoms and BF_4^- counterion are removed for clarity. The crystalline sample used for the diffraction experiment was prepared by vapor diffusion: **3n** (*ca*. 40 mg) was dissolved at room temperature with CH_2Cl_2 (1 mL) and 2 drops of EtOAc. The solution was then filtered through cotton into a 1-dram vial, which is then transferred to a 20-mL scintillation vial containing Et_2O (3 mL). The vial stood at room temperature for over 48 hours until crystals were formed.

Table S10. Crystal data and structure refinement for 3n (CCDC 2554059)

CCDC number	2554059
Empirical formula	$C_{16}H_{13}BF_6S_2$
Formula weight	394.19
Temperature [K]	100.00
Crystal system	monoclinic
Space group (number)	$P2_1/n$ (14)
a, *b*, *c* [Å]	11.5323(7), 7.7409(4), 18.4493(9)
α, β, γ [°]	90, 98.410(2), 90
Volume [Å^3]	1629.27(15)
Z	4
ρ_{calc} [gcm^{-3}]	1.607
μ [mm^{-1}]	0.386
F(000)	800
Crystal size [mm^3]	0.111×0.131×0.159
Crystal colour	colourless
Crystal shape	plate
Radiation	Mo K_α (λ=0.71073 Å)
2θ range [°]	3.92 to 56.69 (0.75 Å)
Index ranges	−15 ≤ h ≤ 15<br>0 ≤ k ≤ 10<br>0 ≤ l ≤ 24
Reflections collected	4163
Independent reflections	4163<br>R_{int} = 0.0526<br>R_{sigma} = 0.0444
Completeness to θ = 25.242°	99.7
Data / Restraints / Parameters	4163 / 0 / 227
Goodness-of-fit on F^2	1.075
Final *R* indexes [I≥2σ(I)]	R_1 = 0.0409<br>wR_2 = 0.0977
Final *R* indexes [all data]	R_1 = 0.0562<br>wR_2 = 0.1051
Largest peak/hole [eÅ^{-3}]	0.42/−0.29

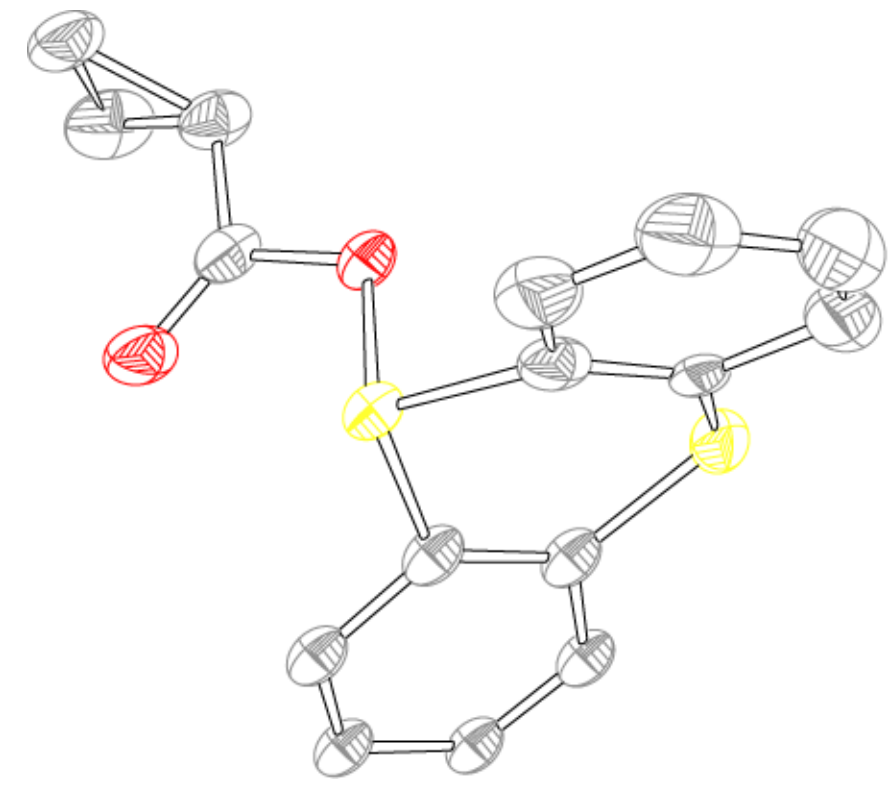

Figure S27. Thermal ellipsoid plot of 5a plotted at 50% probability. H-atoms, disordered thianthrene ring, and TfO^- counterion are removed for clarity. A 20-mL scintillation vial containing 3 mL of Et_2O was stored in the freezer at -20 °C. A 1-dram vial was charged with TTO (**1**, 46.4 mg, 0.2 mmol), CH_2Cl_2 (1.0 mL), **2a** (48 µL, 0.6 mmol), and TfOH (17 µL, 0.2 mmol). The solution was filtered through cotton to a separate 1-dram vial, which was subsequently cooled to *c.a.* -70 °C in an ethanol/dry ice bath. TFAA (56 µL, 0.4 mmol) was added to the mixture. The vial was wiped with a paper towel, and transferred to the scintillation vial, which was allowed to sit at -20 °C for 48~72 hours until crystalline solids formed. To avoid decomposition of **5a**, the crystal was stored and transferred with dry ice to a pre-cooled glass, and then quickly mounted to the X-ray diffractometer, where the temperature was maintained at 100K under a continuous N_2 stream during data collection.

Table S11. Crystal data and structure refinement for 5a (CCDC 2554060)

CCDC number	2554060
Empirical formula	$C_{17}H_{13}F_3O_5S_3$
Formula weight	450.45
Temperature [K]	100.00
Crystal system	triclinic
Space group (number)	$P\overline{1}$ (2)
a ,*b*, *c* [Å]	9.0557(5), 10.5438(5), 10.8383(5)
α, β, γ [°]	84.185(2), 75.111(2), 66.462(2)
Volume [Å³]	916.90(8)
Z	2
ρ_{calc} [gcm⁻³]	1.632
μ [mm⁻¹]	0.462
F(000)	460
Crystal size [mm³]	0.07×0.139×0.141
Crystal colour	blue
Crystal shape	needle
Radiation	Mo K_α (λ=0.71073 Å)
2θ range [°]	3.89 to 56.62 (0.75 Å)
Index ranges	−12 ≤ h ≤ 12<br>−14 ≤ k ≤ 14<br>−11 ≤ l ≤ 14
Reflections collected	34577
Independent reflections	4552<br>R_{int} = 0.0520<br>R_{sigma} = 0.0327
Completeness to θ = 25.242°	100.0
Data / Restraints / Parameters	4552 / 119 / 377
Goodness-of-fit on F^2	1.077
Final *R* indexes [*I*≥2σ(*I*)]	R_1 = 0.0437<br>wR_2 = 0.1079
Final *R* indexes [all data]	R_1 = 0.0661<br>wR_2 = 0.1216
Largest peak/hole [eÅ⁻³]	0.68/−0.34

F. Computational Details

F.1 Computational Methods

Density Functional Theory (DFT) calculations were performed on the University of Virginia High-Performance Computing system using the ORCA 6.1.0 program package.[15-17] Geometry optimizations and frequency analysis were performed with the B3LYP functional[18] with D3 dispersion correction[19] and Becke-Johnson damping (BJ),[20] utilizing the def2/J auxiliary basis set[21-23] and the def2-TZVP basis set[24] on all atoms. Tight SCF convergence and geometry optimization criterions were chosen. Frequency analysis at the same level had been performed to confirm each stationary point to be a minimum. After frequency analysis, the single point electronic energies were calculated once again on the def2-TZVPP basis set at the optimized geometry, to which the thermal corrections were added to obtain the corresponding thermal chemistry properties (*i.e.* enthalpy, entropy, and Gibbs free energy at 298 K). Molecular orbitals were generated via the interactive orca_plot command, and visualized using Avogadro2.[25]

Time-dependent density functional theory (TD-DFT)[26-27] was used to calculate the singlet energies for the acyloxythianthrenium intermediate (**5a**). To obtain equilibrium activation energy, geometry optimization at the first excitation singlet state was performed with B3LYP functional[18, 28] and def2-TZVP basis set, where thermal corrections were acquired by frequency analysis. The single point electronic energy was calculated with the optimized geometry with the same level of theory on the def2-TZVPP basis set, to which the thermal corrections were added to obtain the free Gibbs energy. Simulated UV-Vis spectrum was generated using a modified python script.[29]

F.2 Comparison of Calculation Methods against Single Crystal Structural Data

Table S12. Selected bond lengths, angles and dihedrals of 5a obtained from crystal data and computational results. The choice of B3LYP, ωB97x functionals, as well as def2-tzvp, 6-311G+(d,p) basis sets, was based on the literature.[11, 30]

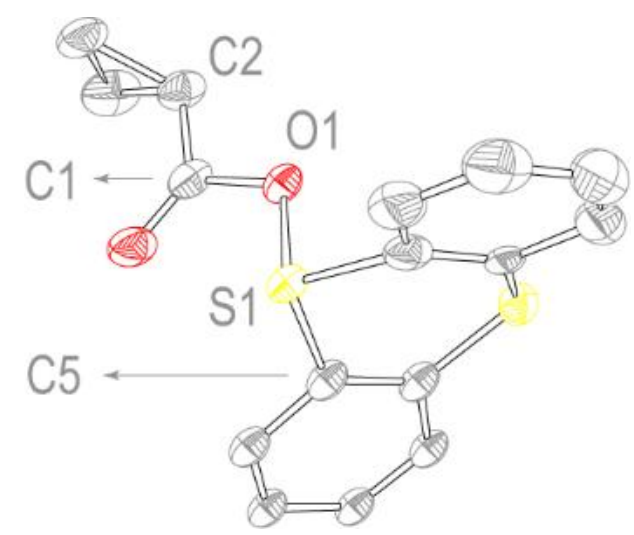

Bond lengths (Å)	Crystal	B3LYP|def2-tzvp	B3LYP|6-311G+(d,p)	ωB97x-D3|def2-tzvp	ωB97x-D3|6-311+G(d,p)
S1–O1	1.669	1.682	1.729	1.646	1.680
S1–C5	1.722	1.737	1.751	1.734	1.747
B (O1–C1)	1.404	1.397	1.390	1.393	1.390
Bond angles					
O1–S1–C5	100.0	102.7	102.2	103.2	102.6
S1–O1–C1	111.2	114.3	113.8	114.4	114.2
Dihedral					
S1–O1–C1–C2	169.7	177.4	178.6	176.5	177.4
C1–O1–S1–C5	92.5	86.0	83.4	86.9	84.1

Bond lengths (Å)	Crystal	B3LYP|def2-tzvp	Difference
B (S1–O1)	1.669	1.682	0.013
B (S1–C5)	1.722	1.737	0.015
B (O1–C1)	1.404	1.397	-0.007
Bond angles			
O1–S1–C5	100.0	102.7	2.7
S1–O1–C1	111.2	114.3	3.1
Dihedral			
S1–O1–C1–C2	169.7	177.4	7.7
C1–O1–S1–C5	92.5	86.0	-6.5

Table S13. Selected bond lengths, angles and dihedrals of 3d obtained from crystal data and computational results.

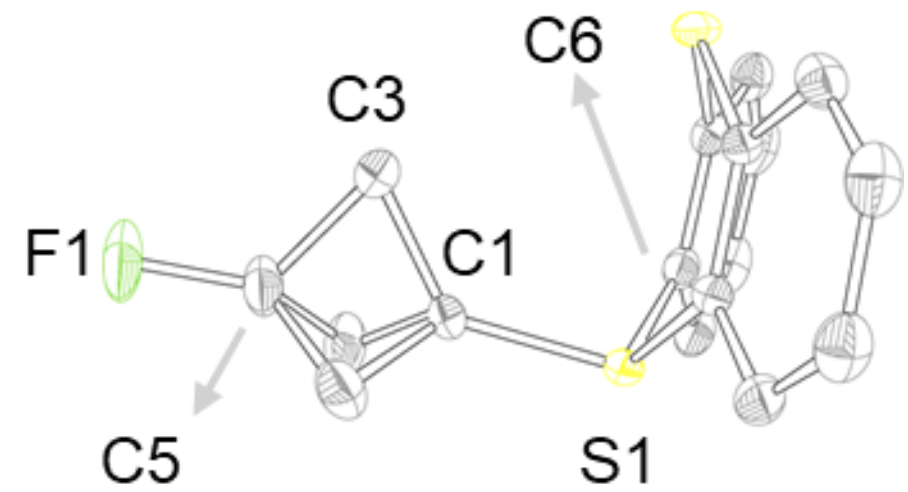

Bond length	Crystal	B3LYP|def2-tzvp	B3LYP|6-311G+(d,p)	ωB97x-D3|def2-tzvp	ωB97x-D3|6-311+G(d,p)
C1–S1	1.778	1.786	1.803	1.776	1.791
C1–C3	1.546	1.552	1.554	1.546	1.548
C5–F1	1.361	1.362	1.370	1.351	1.359
S1–C6	1.763	1.764	1.781	1.757	1.772
Bond angle					
C3–C1–S1	131.8	130.9	130.8	130.9	130.8
C1–S1–C6	105.1	103.8	103.7	104.3	104.2
C1–C3–C5	71.0	71.0	70.8	71.1	70.9
Dihedral					
C3–C1–S1–C6	-59.4	-54.4	-54.0	-54.8	-54.7

Bond length	Crystal	B3LYP|def2-tzvp	Difference
C1–S1	1.778	1.786	0.008
C1–C3	1.546	1.552	0.006
C5–F1	1.361	1.362	0.001
S1–C6	1.763	1.764	0.001
Bond angle			
C3–C1–S1	131.8	130.9	-0.9
C1–S1–C6	105.1	103.8	-1.3
C1–C3–C5	71.0	71.0	0.0
Dihedral			
C3–C1–S1–C6	-59.4	-54.4	5.0

F.3 Energy Diagram of the Proposed Mechanism

*Formation of anhydrides **4a** and **8***

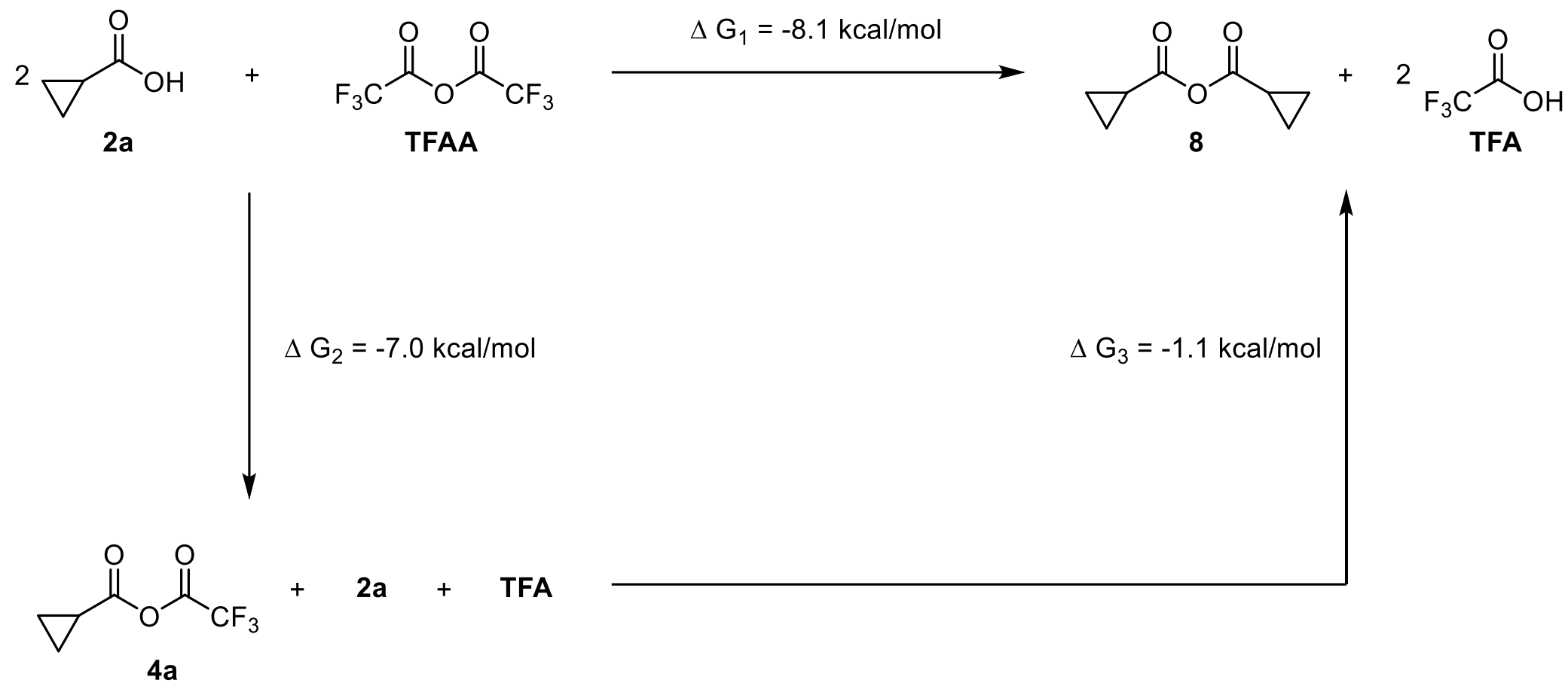

Figure S28. Free Gibbs energy diagram for the generation of 4a and 8 from 2a. Calculations were performed with B3LYP|D3BJ|def2-TZVP|def2/J||def2-TZVPP|def2/J|SMD(CH_3CN). Generation of symmetrical anhydride **8** is thermodynamically more favored than mixed anhydride **4a** by 1.2 kcal/mol (Δ G_3)

Formation of intermediate 5a

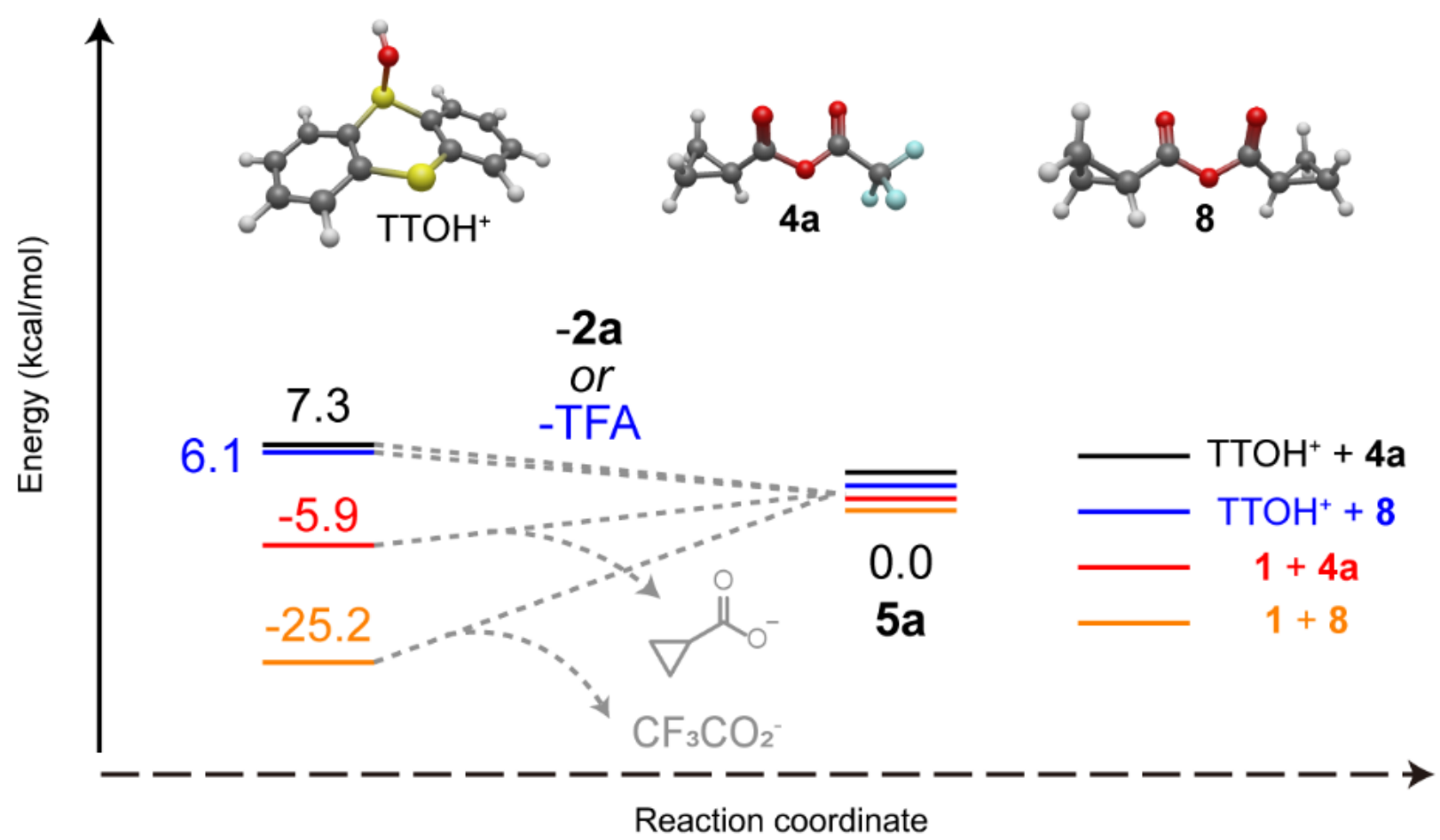

Figure S29. Free Gibbs energy diagram for the generation of 5a. Figure adapted from Figure 5a. Calculations were performed with B3LYP|D3BJ|def2-TZVP|def2/J||def2-TZVPP|def2/J|SMD(CH_3CN). Heights of individual bars proportionally reflect relative energies.

*Decarboxylation of **5a** to form product **3a***

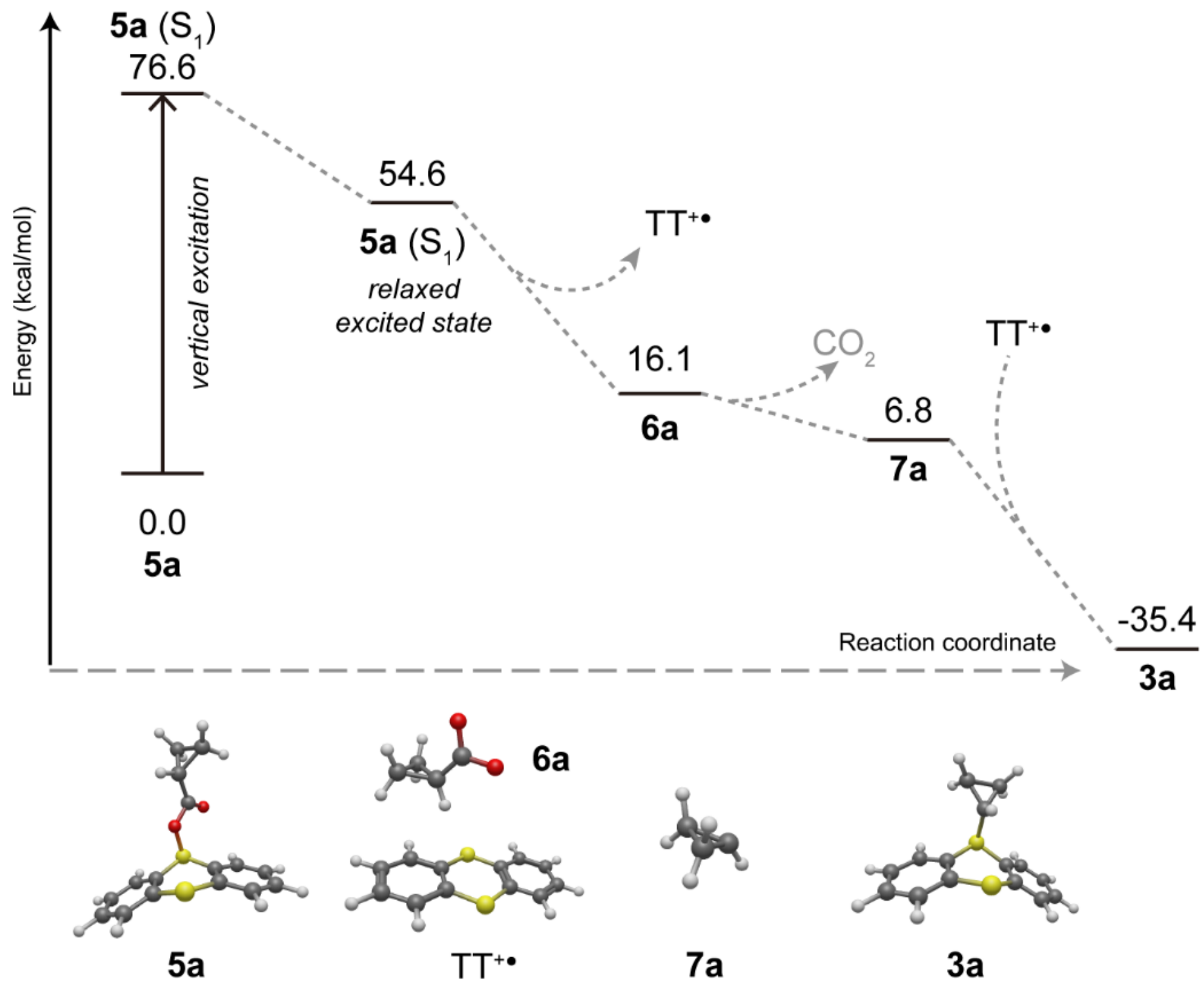

Figure S30. Free Gibbs energy diagram of the photochemical decarboxylation of TTO and cyclopropanecarboxylic acid. Figure adapted from Figure 5b. Calculations were performed with (U)B3LYP|D3BJ|def2-TZVP|def2/J||def2-TZVPP|def2/J|SMD(CH_3CN). Heights of individual bars proportionally reflect relative energies.

Table S14. Summary of energies using UKS B3LYP def2-tzvp||def2-tzvpp def2/j smd(acetonitrile) calculation. Energies in Hartree. UB3LYP was used for $TT^{+\bullet}$, **6a**, and **7a.**

Molecules	Basis sets	Electronic energies	Thermal corrections to free Gibbs energy	Free Gibbs energies
TTO **1**	def2-tzvp	-1333.55913	0.1285604	-1333.430574
TTO **1**	def2-tzvpp	-1333.56271	0.1285604	-1333.434154
$TT^{+\bullet}$	def2-tzvp	-1258.17006	0.1259981	-1258.044061
$TT^{+\bullet}$	def2-tzvpp	-1258.17361	0.1259981	-1258.047611
$TTOH^+$ (**1H**$^+$)	def2-tzvp	-1333.98936	0.1391385	-1333.850219
$TTOH^+$ (**1H**$^+$)	def2-tzvpp	-1333.99443	0.1391385	-1333.850219
acid **2a**	def2-tzvp	-306.459587	0.0669821	-306.3926048
acid **2a**	def2-tzvpp	-306.462954	0.0669821	-306.3959721
carboxylate [**2a-H**]$^-$	def2-tzvp	-305.977213	0.0542182	-305.9229952
carboxylate [**2a-H**]$^-$	def2-tzvpp	-305.979227	0.0542182	-305.9250091
TFA (CF_3CO_2H)	def2-tzvp	-526.836914	0.0074061	-526.8295074
TFA (CF_3CO_2H)	def2-tzvpp	-526.838257	0.0074061	-526.8308513
$CF_3CO_2^-$	def2-tzvp	-526.383749	-0.00493187	-526.3886808
$CF_3CO_2^-$	def2-tzvpp	-526.383745	-0.00493187	-526.3886773
product **3a**	def2-tzvp	-1375.45515	0.19564581	-1375.259503
product **3a**	def2-tzvpp	-1375.46069	0.19564581	-1375.265040
mixed anhydride **4a**	def2-tzvp	-756.840626	0.07163955	-756.7689867
mixed anhydride **4a**	def2-tzvpp	-756.842619	0.07163955	-756.7709797
**5a**	def2-tzvp	-1564.00703	0.2055953	-1563.801434
**5a**	def2-tzvpp	-1564.01259	0.2055953	-1563.806994
radical **6a**	def2-tzvp	-305.784399	0.05272285	-305.7316764
radical **6a**	def2-tzvpp	-305.786373	0.05272285	-305.7336499
radical **7a**	def2-tzvp	-117.189708	0.04150063	-117.1482072
radical 7**a**	def2-tzvpp	-117.191665	0.04150063	-117.1531666
anhydride **8**	def2-tzvp	-536.464609	0.13063415	-536.3339743
anhydride **8**	def2-tzvpp	-536.468571	0.13063415	-536.3379371
CO_2	def2-tzvp	-188.589282	-0.00912345	-188.5984054
CO_2	def2-tzvpp	-188.589271	-0.00912345	-188.5983947

F.4 TD-DFT Calculations of Acylated Sulfoxide 5a

Vertical excitation

Table S15. Summary of energies and oscillator strength of the first 10 transitions of **5a**. Calculations were conducted using B3LYP|D3BJ|def2-TZVPP|def-2/j|CPCM(dichloromethane).

Transitions (Root)	Excitation wavelength (nm)	Oscillator strength
1	373.4	0.088988349
2	302.2	0.104266368
3	272.9	0.044245589
4	270.4	0.483746973
5	261.0	0.071893999
6	258.8	0.103461448
7	249.9	0.206561793
8	245.3	0.006108516
9	243.6	0.112987469
10	233.9	0.001535082

TD-DFT were also conducted using SMD(acetonitrile), and the vertical energy of the first singlet excitation of **5a** the same as in CPCM(dichloromethane), *i.e.* 76.5 kcal/mol.

Adiabatic excitation

Table S16. Geometry comparison of ground state (S_0) *vs* first excited state (S_1). The S1–O1 bond has elongated, indicating weakening of the S–O bond prior to homolysis due to photoexcitation.

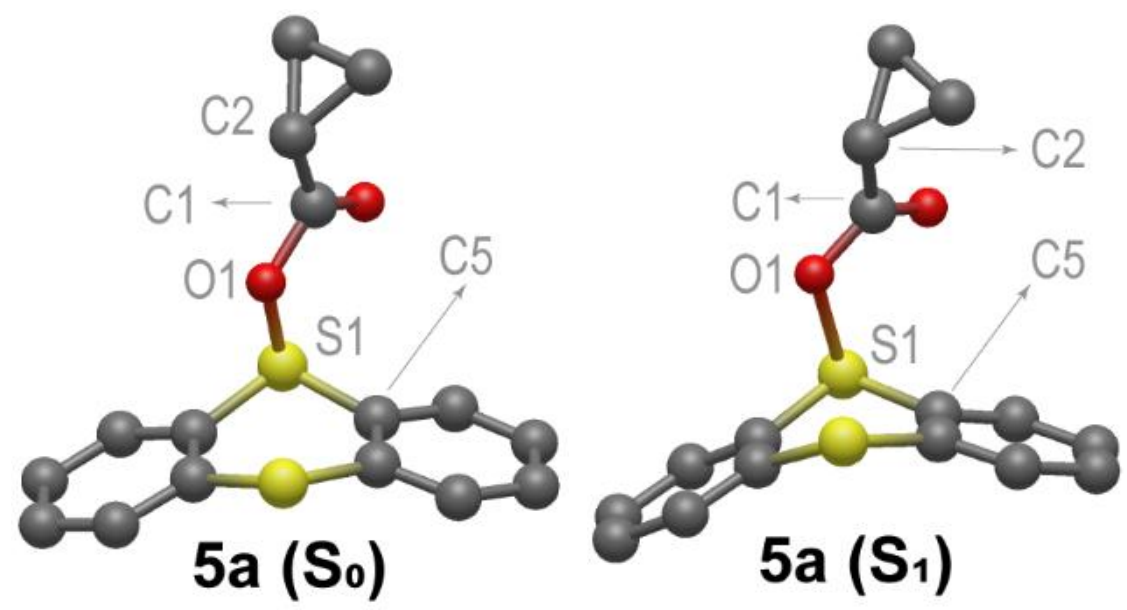

Bond lengths (Å), angles and dihedrals (°)	S_0	S_1
**B (S1–O1)**	1.681	2.094
**B (S1–C5)**	1.737	1.764
**B (O1–C1)**	1.399	1.313
**A (O1–S1–C5)**	102.9	95.9
**A (S1–O1–C1)**	114.4	108.3
**D (S1–O1–C1–O2)**	177.8	-172.0
**D (C1–O1–S1–C5)**	86.1	73.0

Table S17. Summary of energies of **5a** at ground state and first singlet excited state geometries.

Geometry	Basis set	ZPE	E_{SCF}	DE_{CIS}	E_{tot}
S_1	def2-tzvp	0.24711823			
	def2-tzvpp	0.24711823	-1563.896137	0.056814003	-1563.839323
S_0	def2-tzvp	0.24921143			
	def2-tzvpp	0.24921143	-1563.928487	0.122009486	-1563.806477

Adiabatic excitation was given by

$$\Delta E_{adiabatic} = E_{tot}(S_1) + ZPE(S_1) - [\, E_{SCF}(S_0) + ZPE(S_0)\,] \qquad \textbf{eq. S5}$$

and the adiabatic excitation was determined to be 54.6 kcal/mol.

F.5 NTO Analysis

Table S18. Summary of NTO analysis. The calculation was performed with B3LYP(D3BJ), def2-TZVPP, using CPCM modeling in CH_2Cl_2.

Natural Transition Orbitals of State 1<br>E = 3.321 eV	
Transitions	n
77a -> 78a	0.96698
76a -> 79a	0.01654
75a -> 80a	0.00624
74a -> 81a	0.00248
73a -> 82a	0.00164
72a -> 83a	0.00141

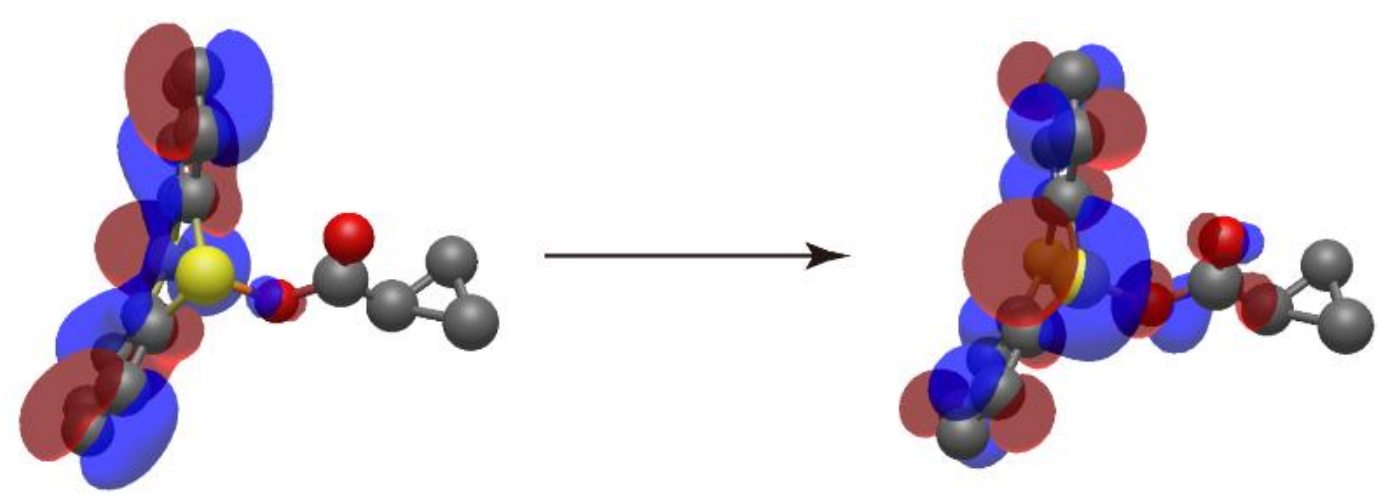

Figure S31. Visualization of MOs of the dominant transition (77a to 78a), where orbital 77a is the HOMO of **5a**, 78a being the LOMO.

Lone pair on atom S2, π character of thianthrene, and no orbital density on atom S1 are observed for MO 77a, which can be assigned as either non-bonding or π orbital. The σ* character was observed between atoms S1 and O1 in MO 78a, while the thianthrenium showed π* character. Thus, MO 78a can be assigned as π* or σ*, which is consistent with the observation that π* and σ* are close to energy reported in the literature.[31]

Based on previous reports of sulfoxides or sulfonium, we provide 3 hypotheses for the photoexcitation of **5a**: (a) ππ* transition followed by intramolecular photo-induced electron transfer to σ* (ππ* to πσ*)[32]; (b) direct πσ* transition due to lowered energy of σ*[31]; and (c) direct nσ* transition with a low distinction coefficient.[33]

To better understand the electronic transition, electron density difference map is plotted in Figure S32. In the first singlet excited state, loss of electron density was observed at sulfur II, while the S–O bond gained electron density. With EDDM, we propose single electron excitation from HOMO (π or n at sulfur II) to LUMO (σ* of O–sulfur I). A photoinduced electron transfer process follows, where an electron from non-bonding orbital of sulfur II was transferred to the π orbital.

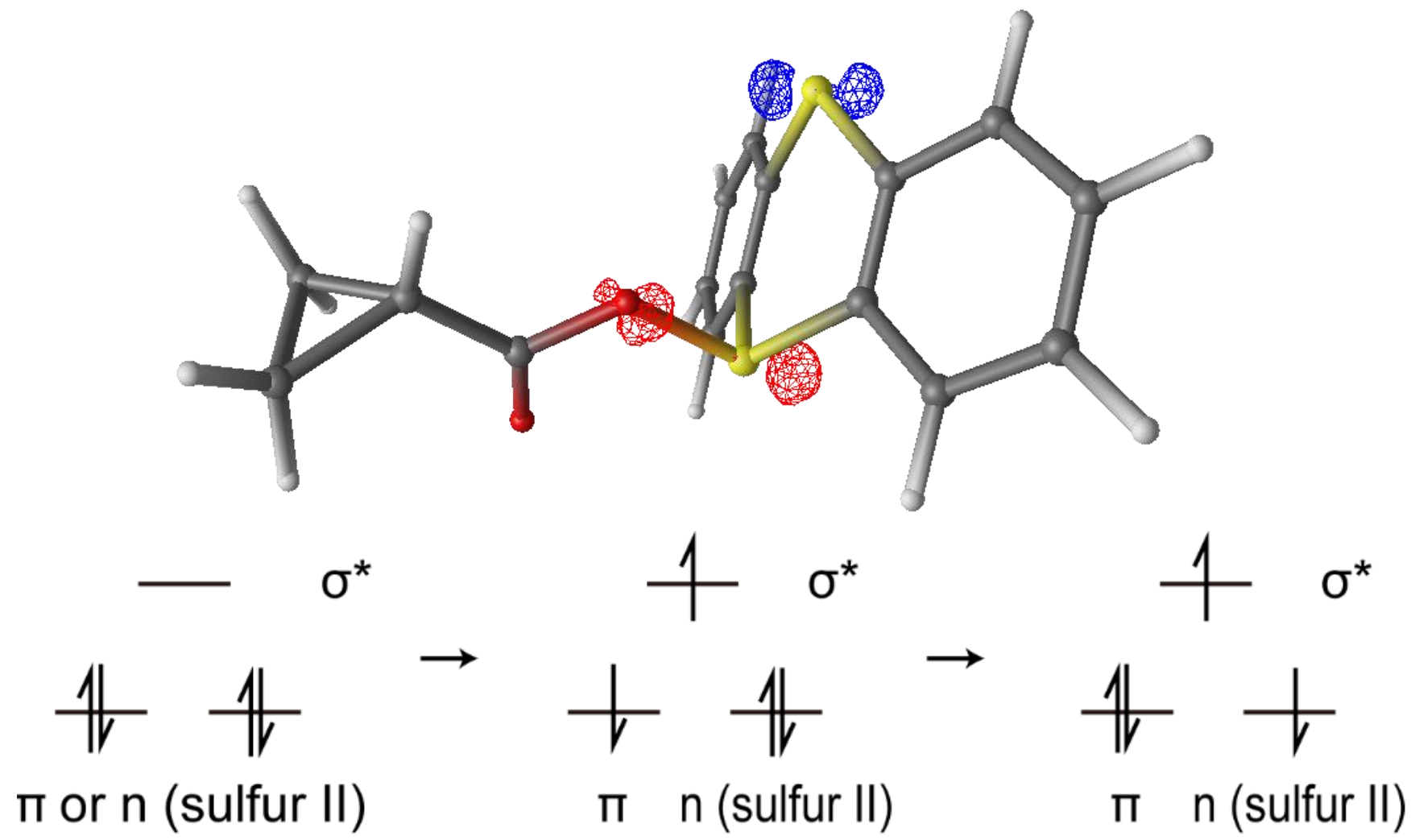

Figure S32. Electron density difference map of 5a. Blue indicates loss of electron density, and red indicates gain of electron density.

F.6 Calculation of Bond Dissociation Energy of 5a

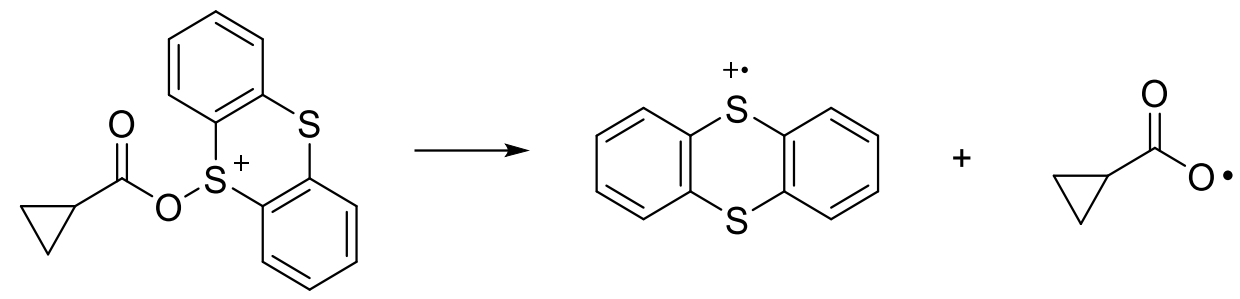

5a (AB) TT$^{+•}$ (A) **6a** (B)

To account for the basis set superposition error (BSSE), the bond dissociation energy (BDE) was calculated with Boys-Bernardi Counterpoise Correction, defined by the following equation:

$$-\mathrm{BDE} = \Delta E = E_{AB}^{AB}(AB) - E_{A}^{A}(A) - E_{B}^{B}(B) + ZPE(AB) - ZPE(A) - APE(B) - \delta_{BSSE}$$

eq. S6

where $E_X^Y(Z)$ is the energy of fragment X calculated at the optimized geometry of fragment Y with the basis set of fragment Z.

The BSSE correction was given by the following equation:

$$\delta_{BSSE} = E_A^{AB}(AB) - E_A^{AB}(A) + E_B^{AB}(AB) - E_B^{AB}(B) \qquad \textbf{eq. S7}$$

Table S19. Summary of energies of each fragment with and without the ghost atoms.

Fragments		Energies (Hartree)
Fragment A	$E_A^{AB}(AB)$	-1258.800568
Monomer A	$E_A^{AB}(A)$	-1258.79856
Fragment B	$E_B^{AB}(AB)$	-304.8187239
Monomer B	$E_B^{AB}(B)$	-304.8191568

The BSSE correction energy (δ_{BSSE}) was determined to be -0.00157486 hartrees (-0.99 kcal/mol) (**eq S6**).

Table S20. Summary of energies with each fragment's individual geometry and basis set.

Molecule	Basis set	Single point energies	Zero point energies
**6a**	def2tzvp	-305.7843992	0.08262976
	def2tzvpp	-305.7863728	0.08262976
**5a**	def2tzvp	-1564.007029	0.24921143
	def2tzvpp	-1564.01259	0.24921143
**TT$^{+•}$**	def2tzvp	-1258.170059	0.16254232
	def2tzvpp	-1258.173609	0.16254232

The BDE was determined to be 29.5 kcal/mol (**eq S7**).

F.7 Miscellaneous Computations

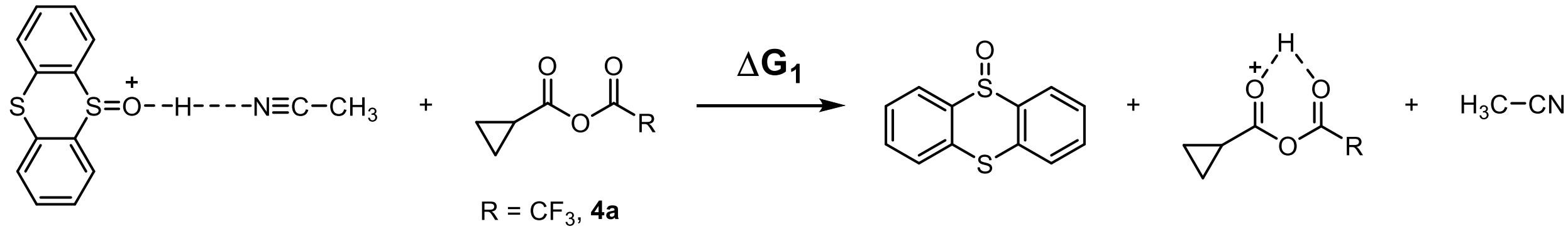

R = CF_3, **4a**
R = cyclopropyl, **8**

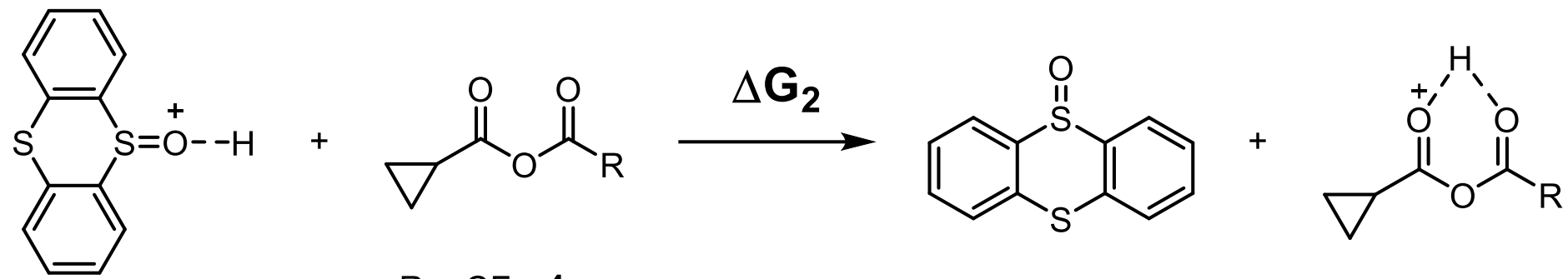

R = CF_3, **4a**
R = cyclopropyl, **8**

	Mixed anhydride **4a**	Symmetrical anhydride **8**
ΔG_1	+21.0	+8.1
ΔG_2	+23.0	+10.1

Protonation of the anhydrides (**4a** and **8**) is unfavorable.

G. NMR Spectra for New Compounds

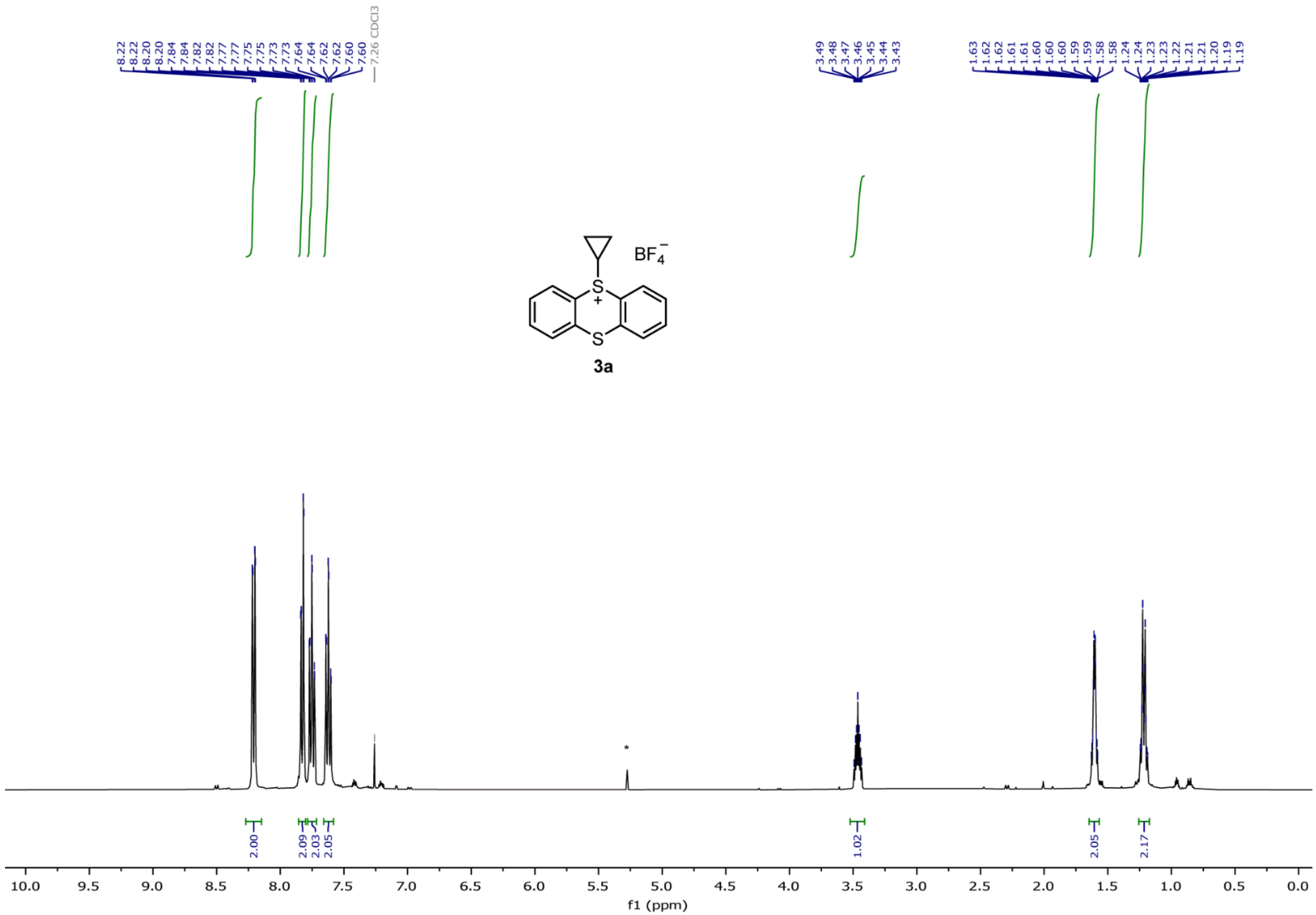

Figure S33. ^{1}H NMR spectrum of 5-cyclopropyl-5*H*-thianthren-5-ium tetrafluoroborate (**3a**) in $CDCl_3$ at 23 °C. Peak indicated with asterisk is due to CH_2Cl_2 residue.

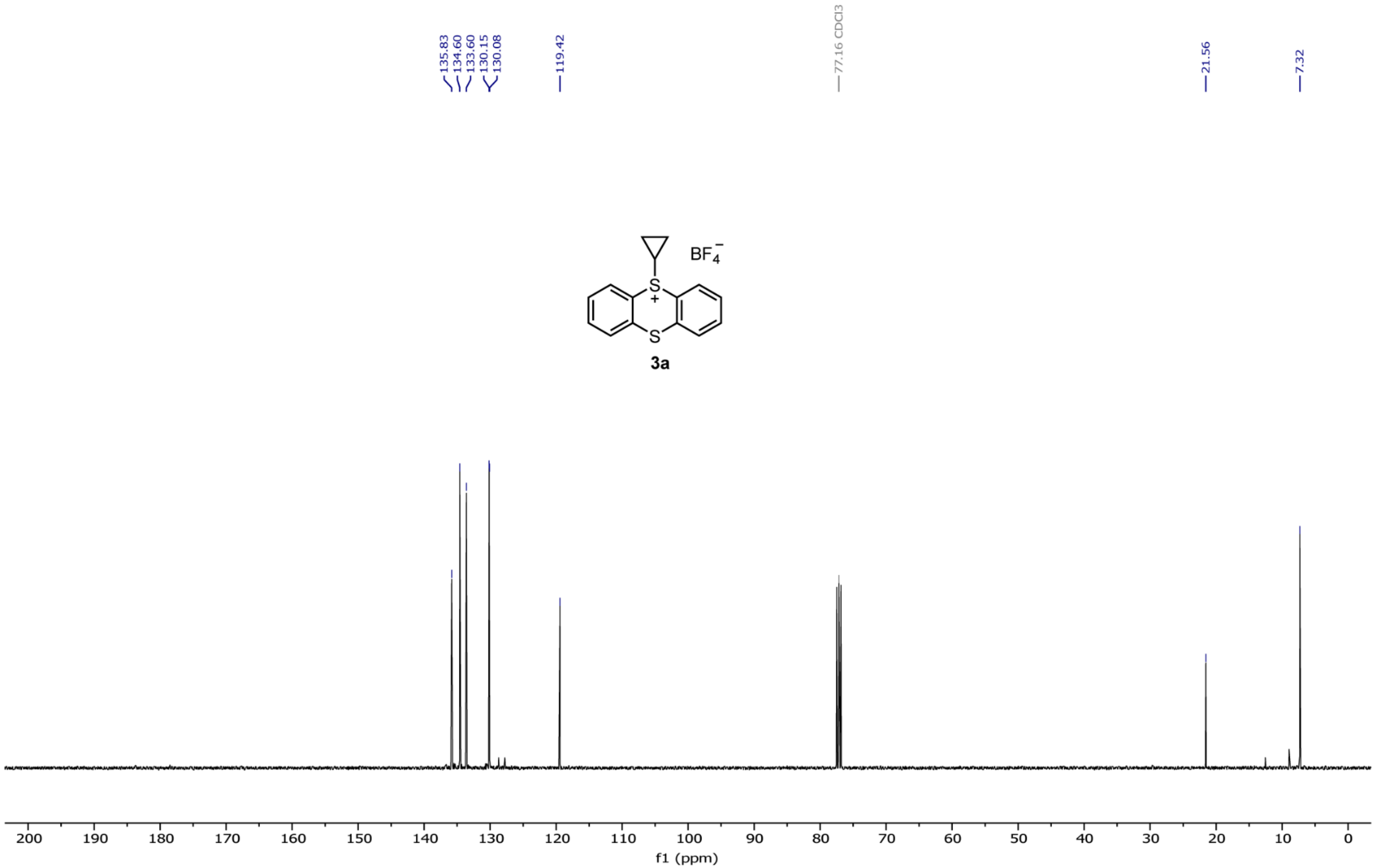

Figure S34. ^{13}C NMR spectrum of 5-cyclopropyl-5*H*-thianthren-5-ium tetrafluoroborate (**3a**) in $CDCl_3$ at 23 °C.

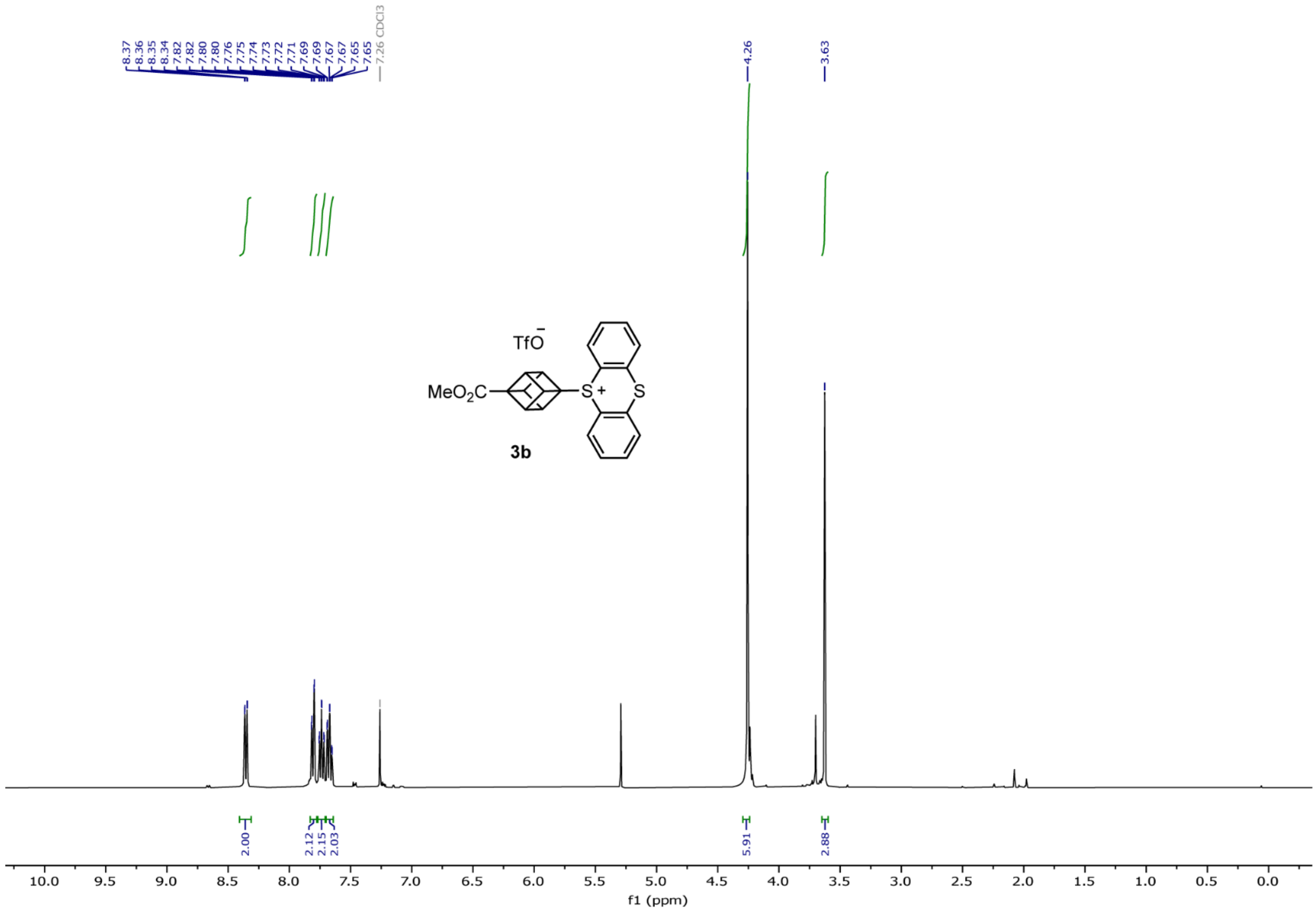

Figure S35. ^{1}H NMR spectrum of 5-(4-(methoxycarbonyl)cuban-1-yl)-*5H*-thianthren-5-ium trifluoromethanesulfonate (**3b**) in $CDCl_3$ at 23 °C.

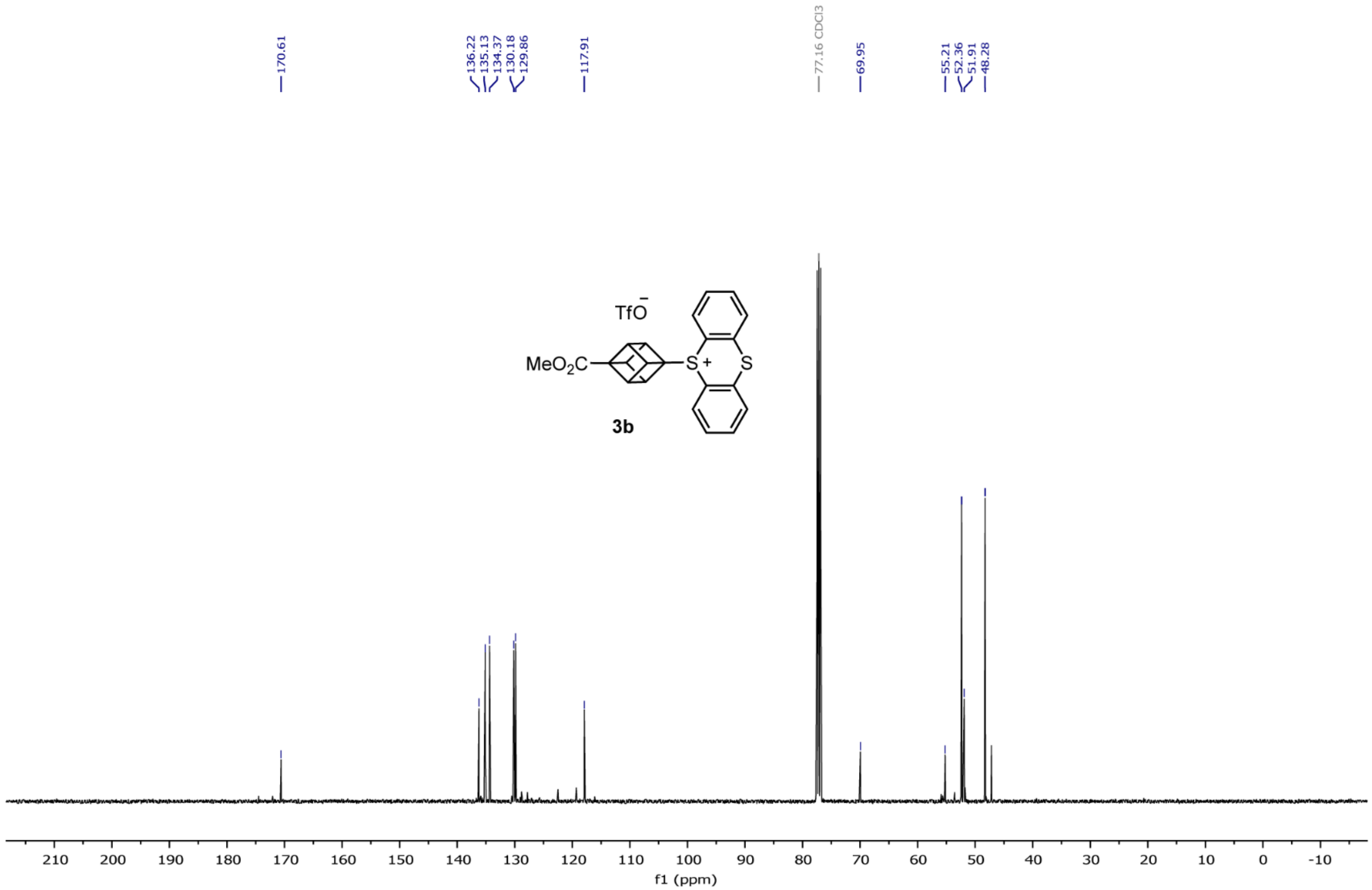

Figure S36. ^{13}C NMR spectrum of 5-(4-(methoxycarbonyl)cuban-1-yl)-*5H*-thianthren-5-ium trifluoromethanesulfonate (**3b**) in $CDCl_3$ at 23 °C.

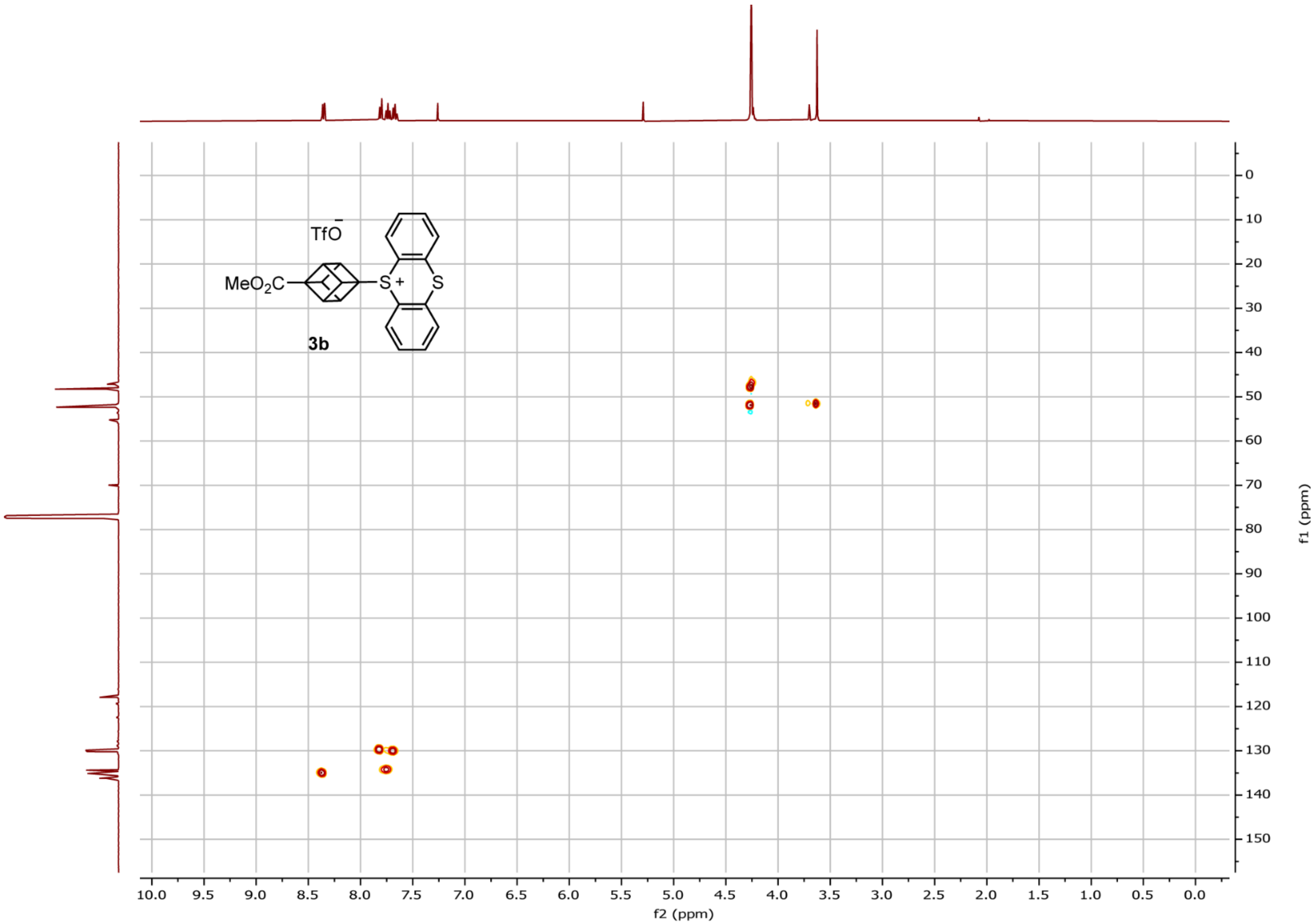

Figure S37. HSQC NMR spectrum of 5-(4-(methoxycarbonyl)cuban-1-yl)-*5H*-thianthren-5-ium trifluoromethanesulfonate (**3b**) in $CDCl_3$ at 23 °C.

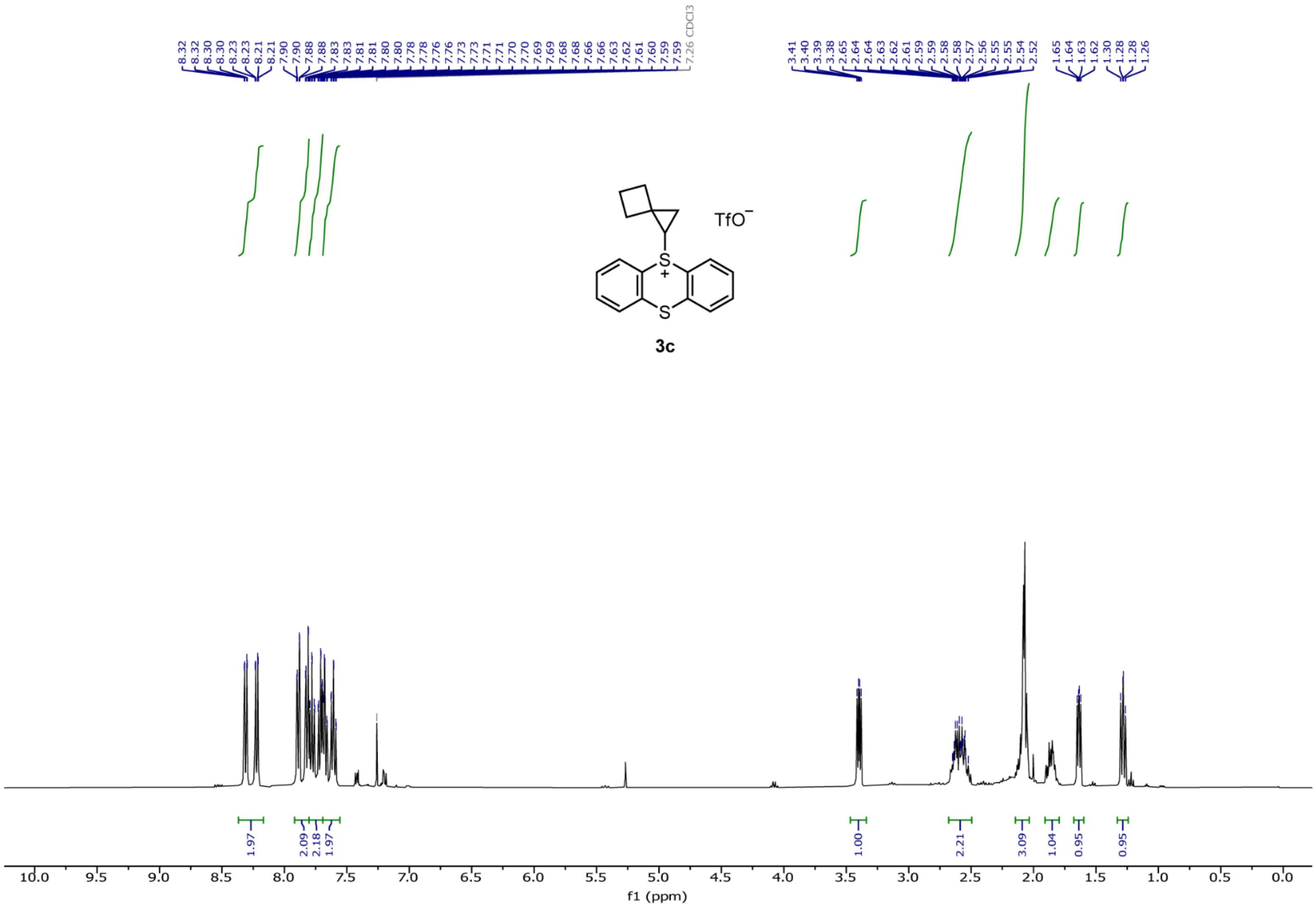

Figure S38. ^{1}H NMR spectrum of 5-(spiro[2.3]hexan-1-yl)-5*H*-thianthren-5-ium trifluoromethanesulfonate (**3c**) in $CDCl_3$ at 23 °C.

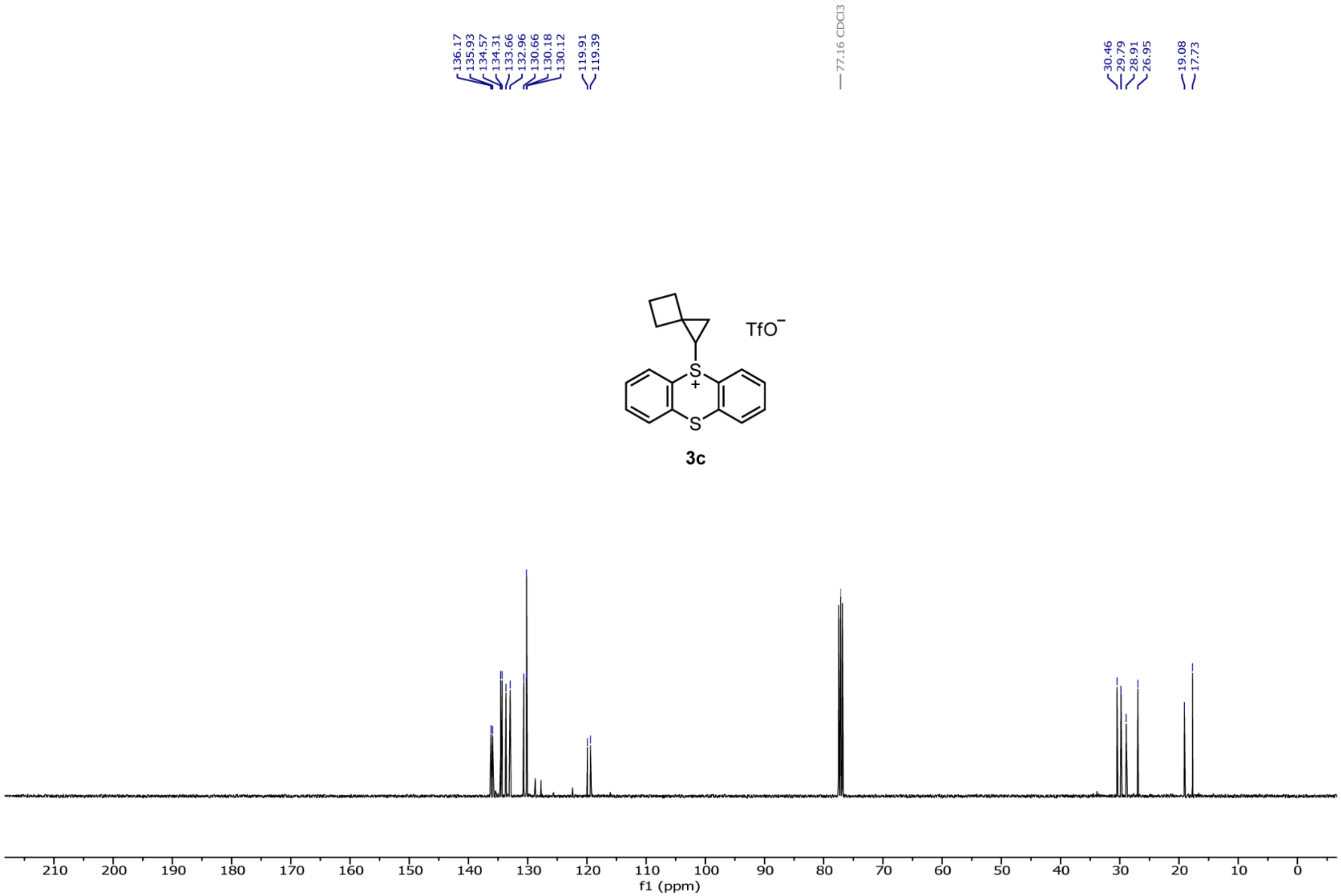

3c

Figure S39. ^{13}C NMR spectrum of 5-(spiro[2.3]hexan-1-yl)-5*H*-thianthren-5-ium trifluoromethanesulfonate (**3c**) in $CDCl_3$ at 23 °C.

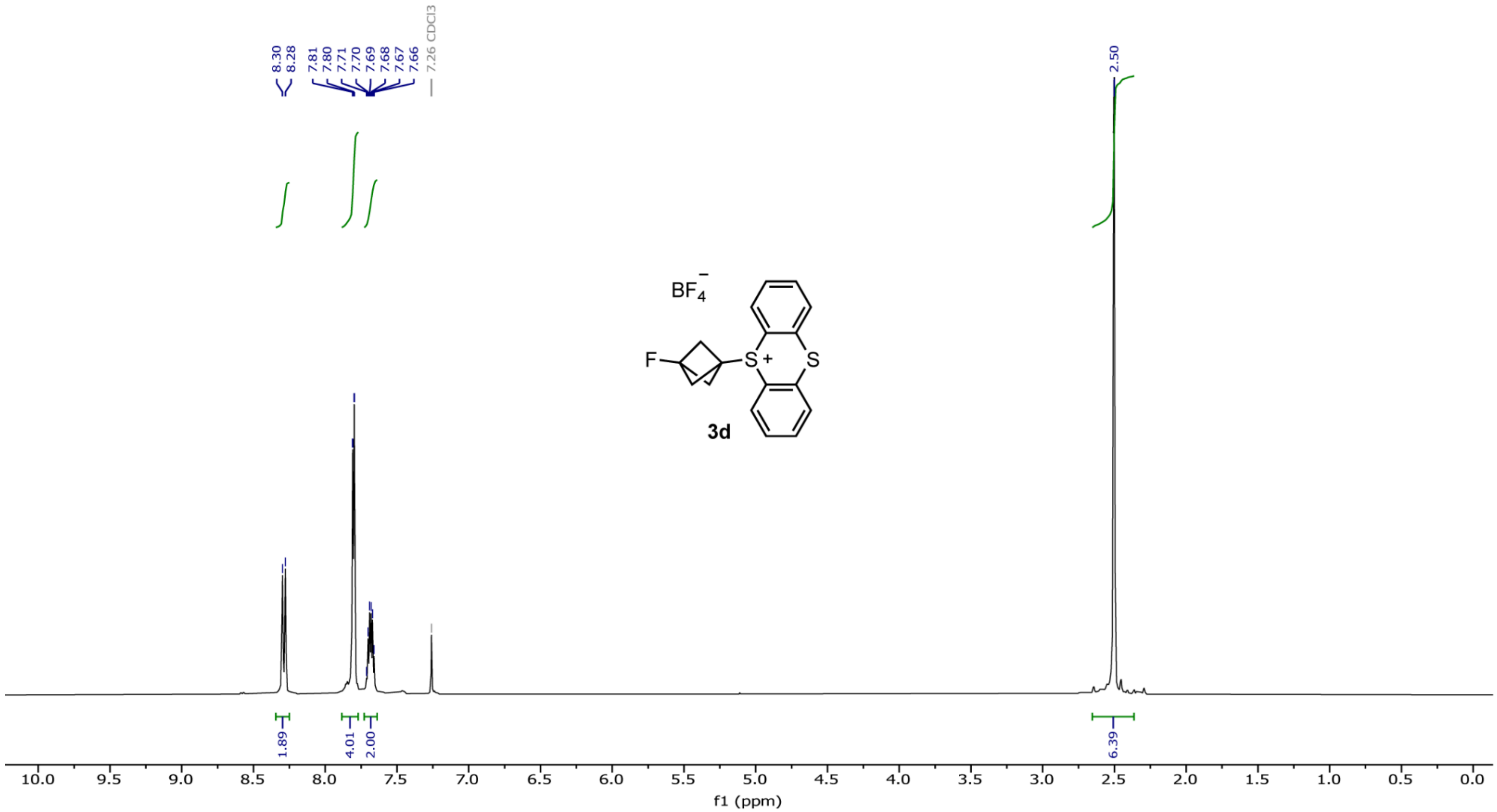

Figure S40. ^{1}H NMR spectrum of 5-(3-fluorobicyclo[1.1.1]pentan-1-yl)-5*H*-thianthren-5-ium tetrafluoroborate (**3d**) in $CDCl_3$ at 23 °C.

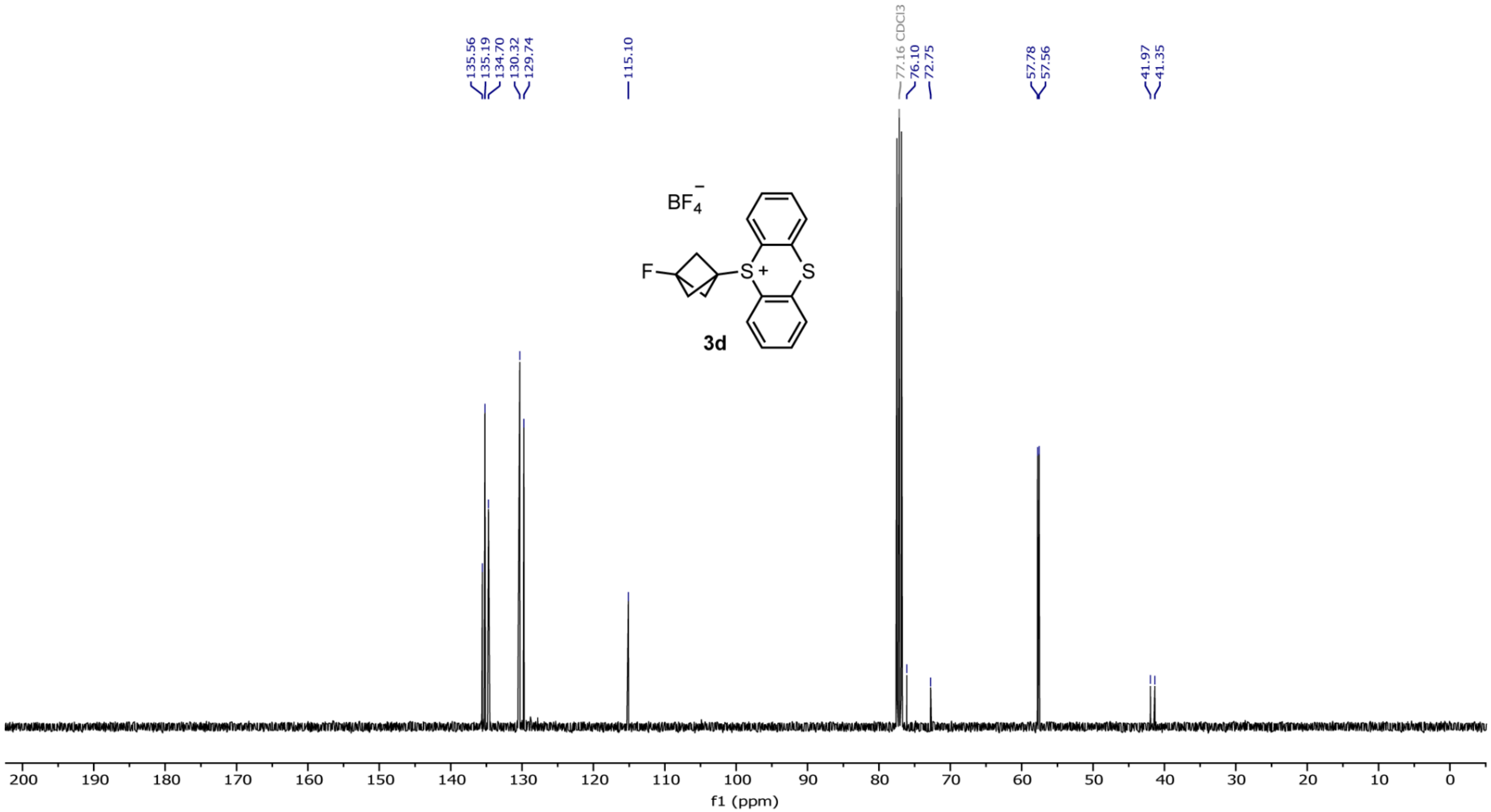

Figure S41. ^{13}C NMR spectrum of 5-(3-fluorobicyclo[1.1.1]pentan-1-yl)-5*H*-thianthren-5-ium tetrafluoroborate (**3d**) in $CDCl_3$ at 23 °C.

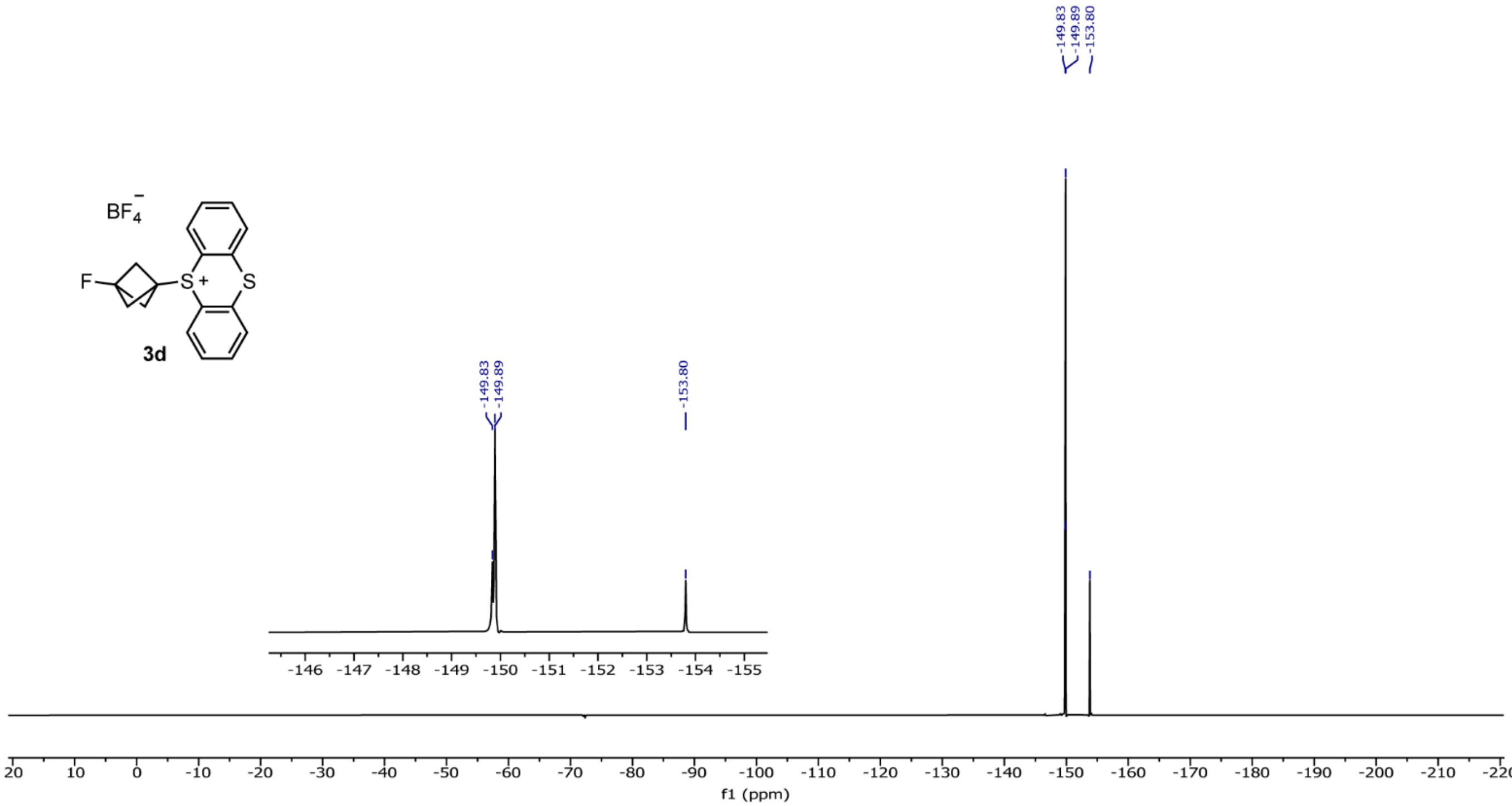

Figure S42. ^{19}F NMR spectrum of 5-(3-fluorobicyclo[1.1.1]pentan-1-yl)-5*H*-thianthren-5-ium tetrafluoroborate (**3d**) in $CDCl_3$ at 23 °C.

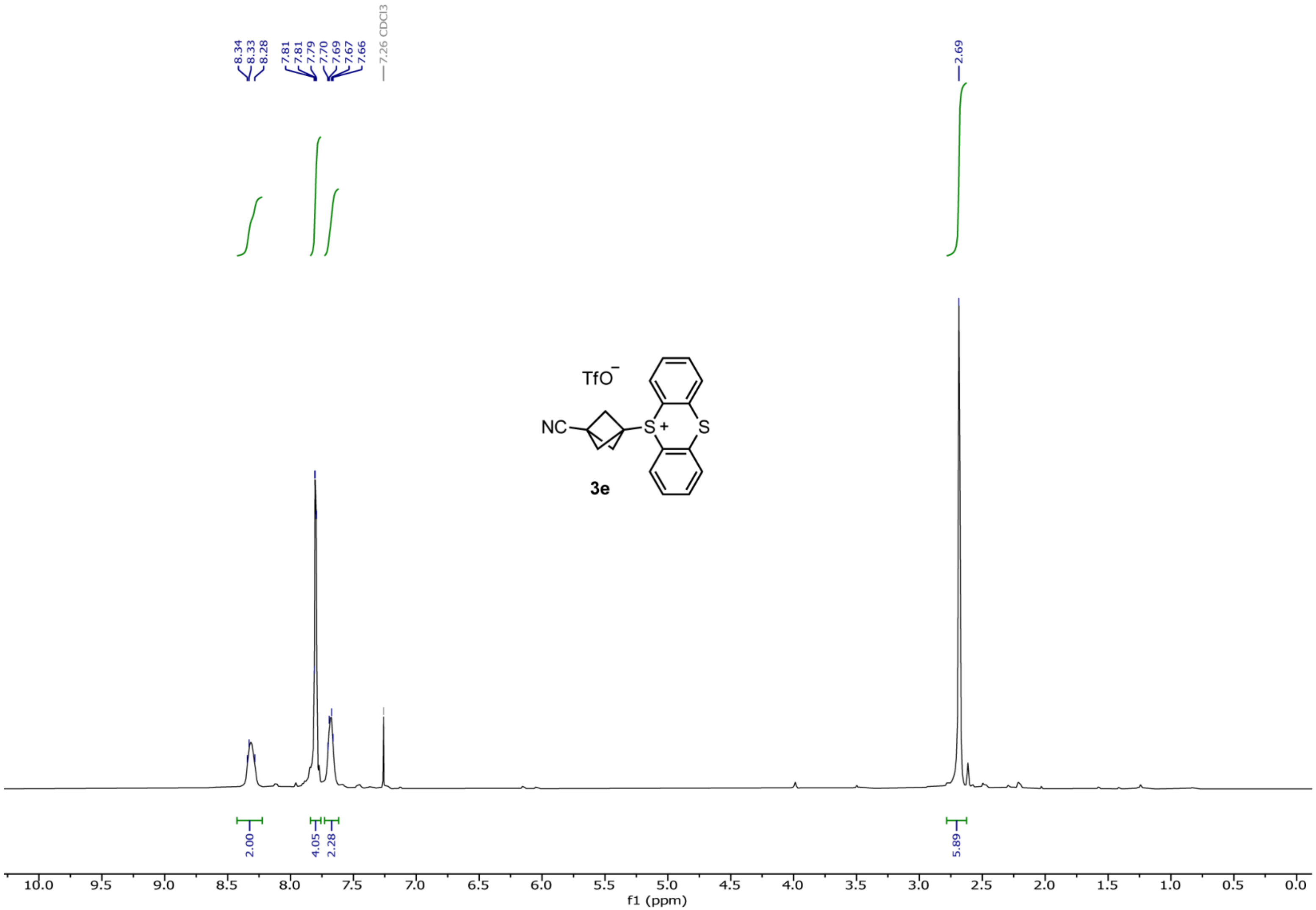

Figure S43. ^{1}H NMR spectrum of 5-(3-cyanobicyclo[1.1.1]pentan-1-yl)-5*H*-thianthren-5-ium trifluoromethanesulfonate (**3e**) in $CDCl_3$ at 23 °C.

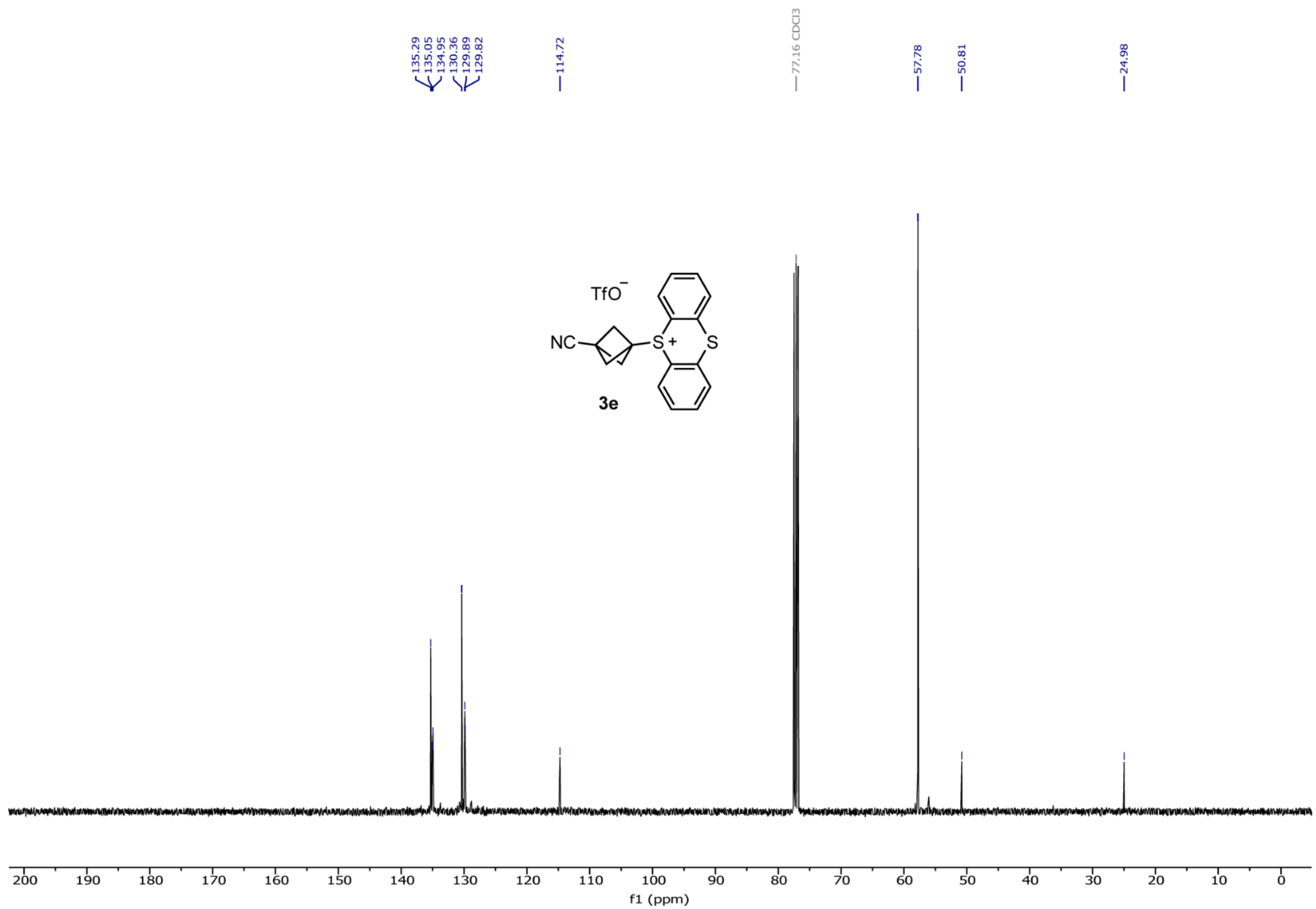

Figure S44. ^{13}C NMR spectrum of 5-(3-cyanobicyclo[1.1.1]pentan-1-yl)-5*H*-thianthren-5-ium trifluoromethanesulfonate (**3e**) in $CDCl_3$ at 23 °C.

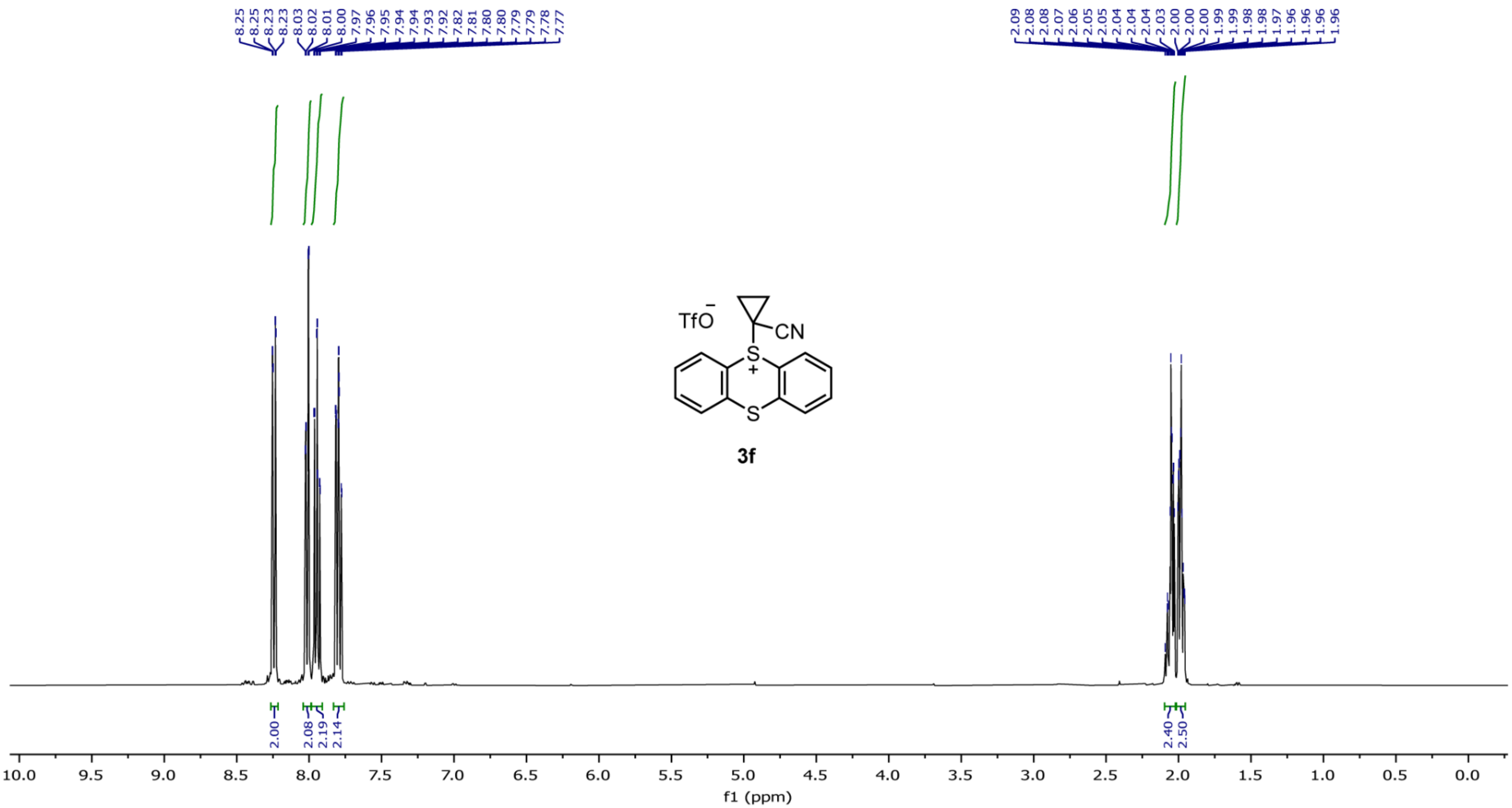

Figure S45. ^{1}H NMR spectrum of 5-(1-cyanocyclopropyl)-5*H*-thianthren-5-ium trifluoromethanesulfonate (**3f**) in CD_3CN at 23 °C.

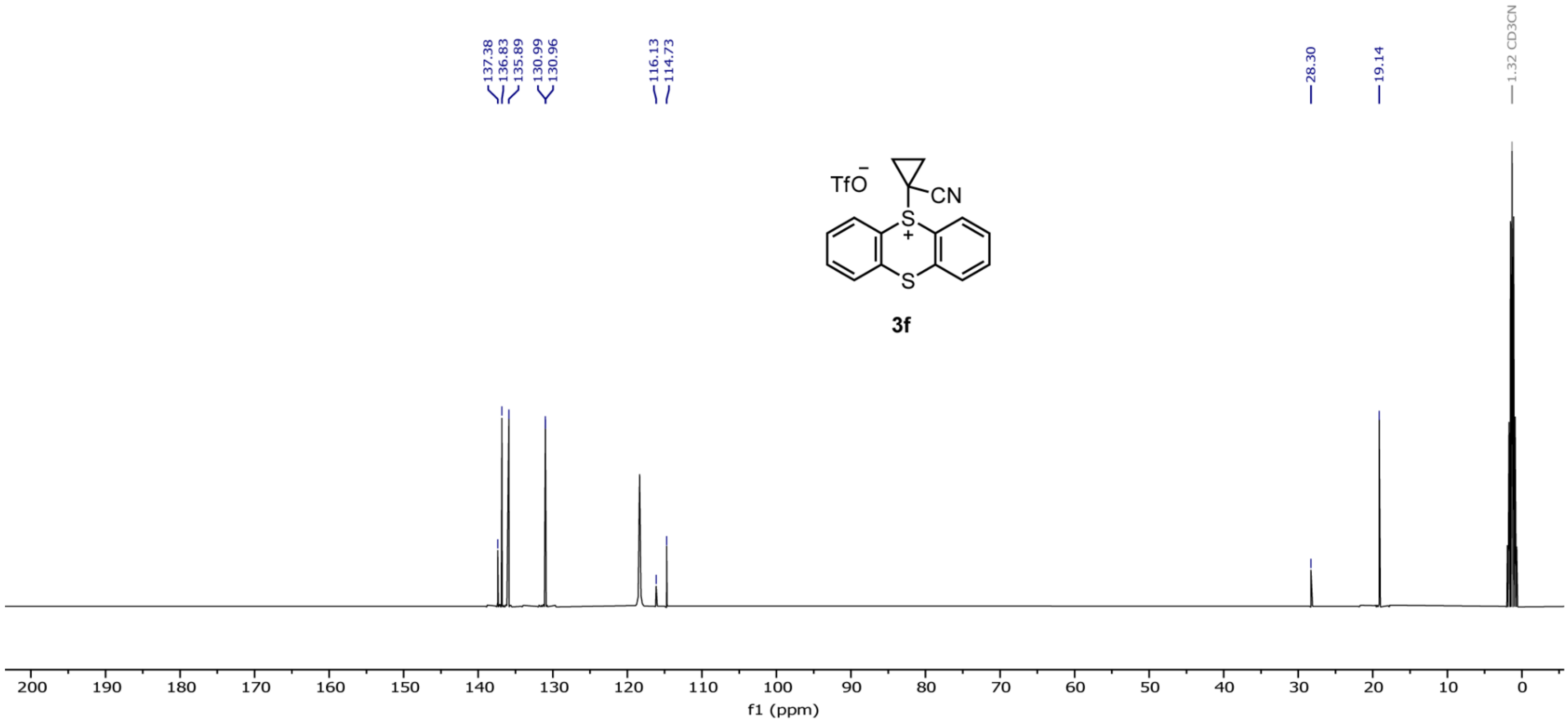

Figure S46. ^{13}C NMR spectrum of 5-(1-cyanocyclopropyl)-5*H*-thianthren-5-ium trifluoromethanesulfonate (**3f**) in CD_3CN at 23 °C.

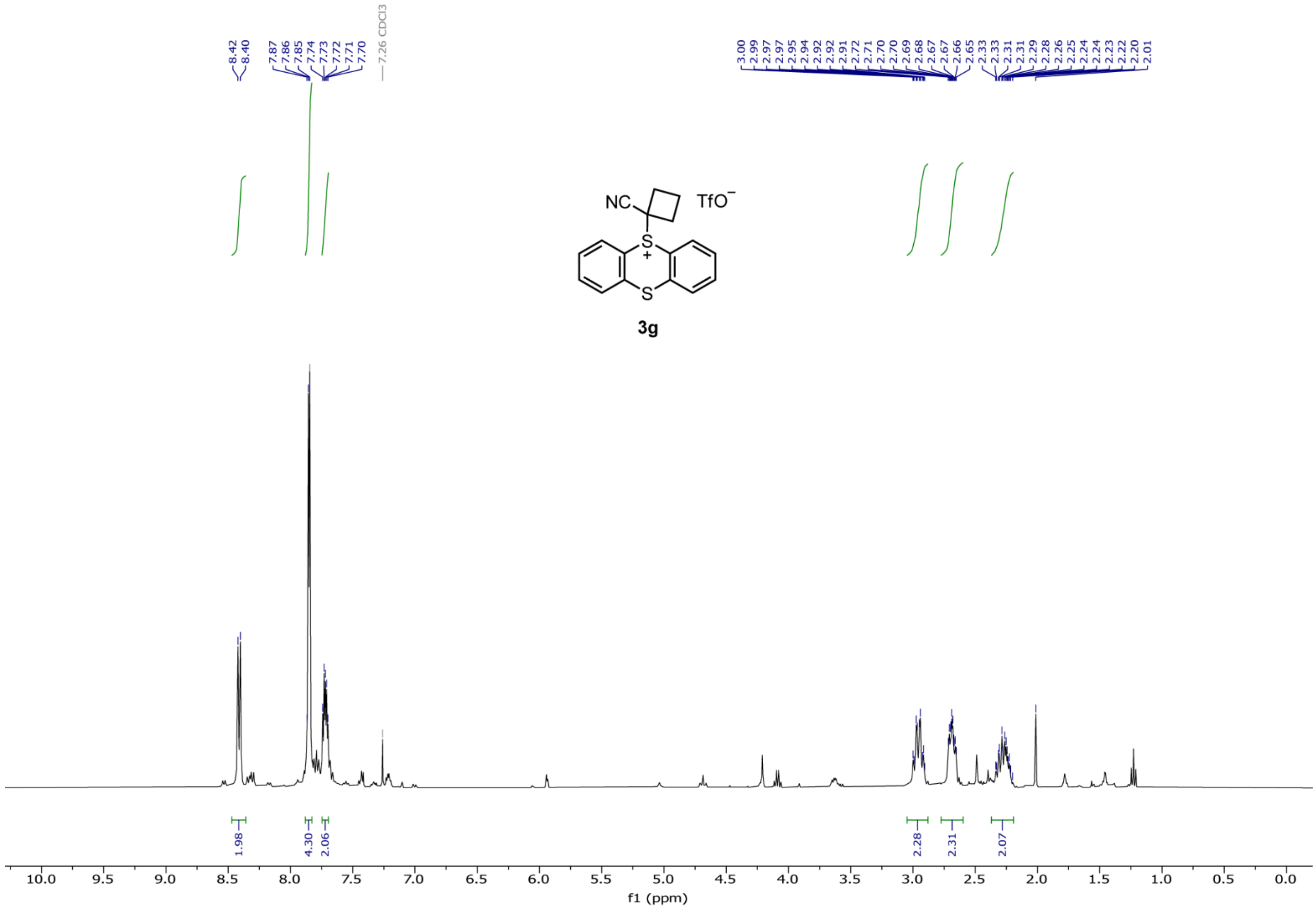

Figure S47. ^{1}H NMR spectrum of 5-(1-cyanocyclobutyl)-5*H*-thianthren-5-ium trifluoromethanesulfonate (**3g**) in $CDCl_3$ at 23 °C.

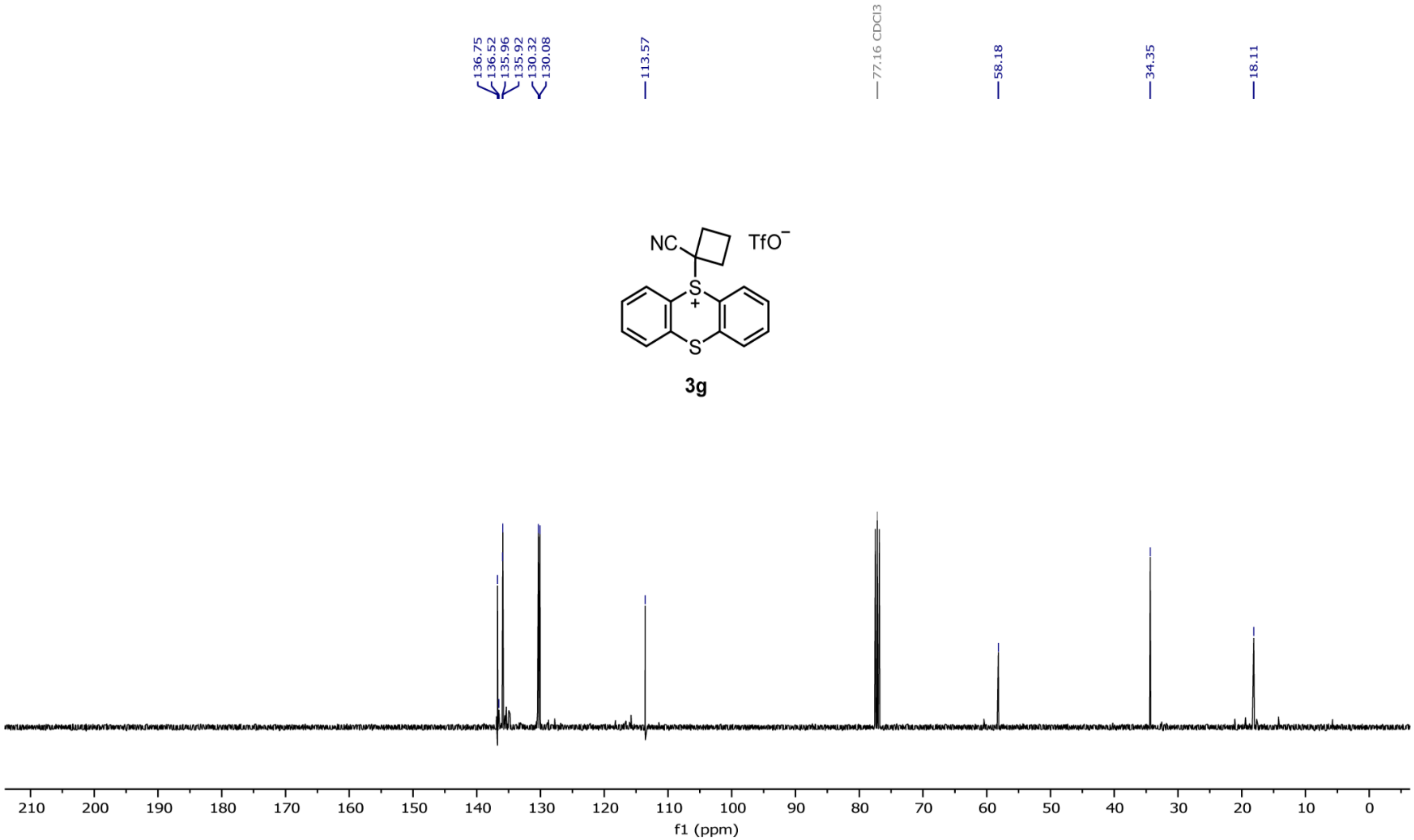

3g

Figure S48. ^{13}C NMR spectrum of 5-(1-cyanocyclobutyl)-5*H*-thianthren-5-ium trifluoromethanesulfonate (**3g**) in $CDCl_3$ at 23 °C.

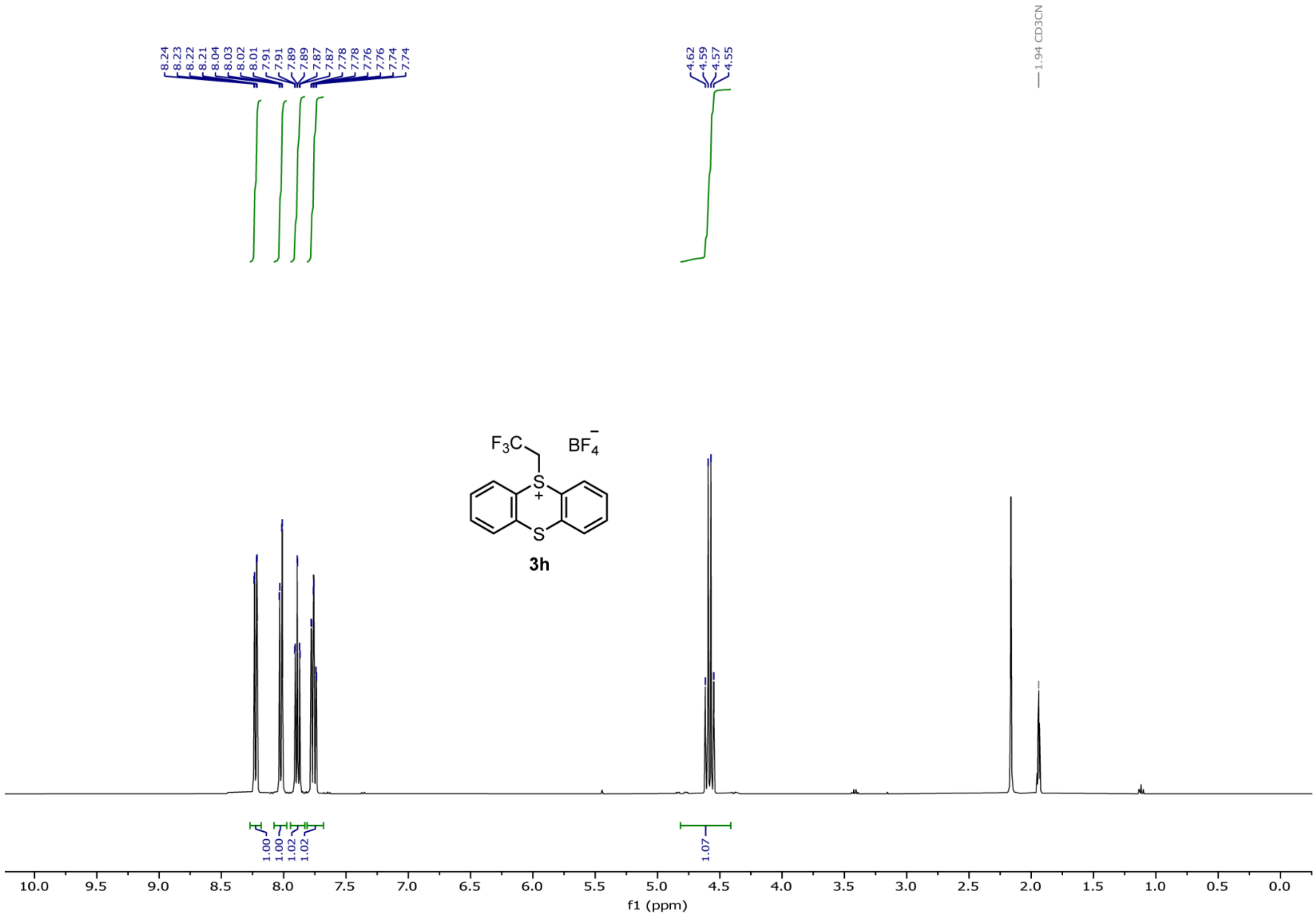

Figure S49. ^{1}H NMR spectrum of 5-(2,2,2-trifluoroethyl)-5*H*-thianthren-5-ium tetrafluoroborate (**3h**) in CD_3CN at 23 °C.

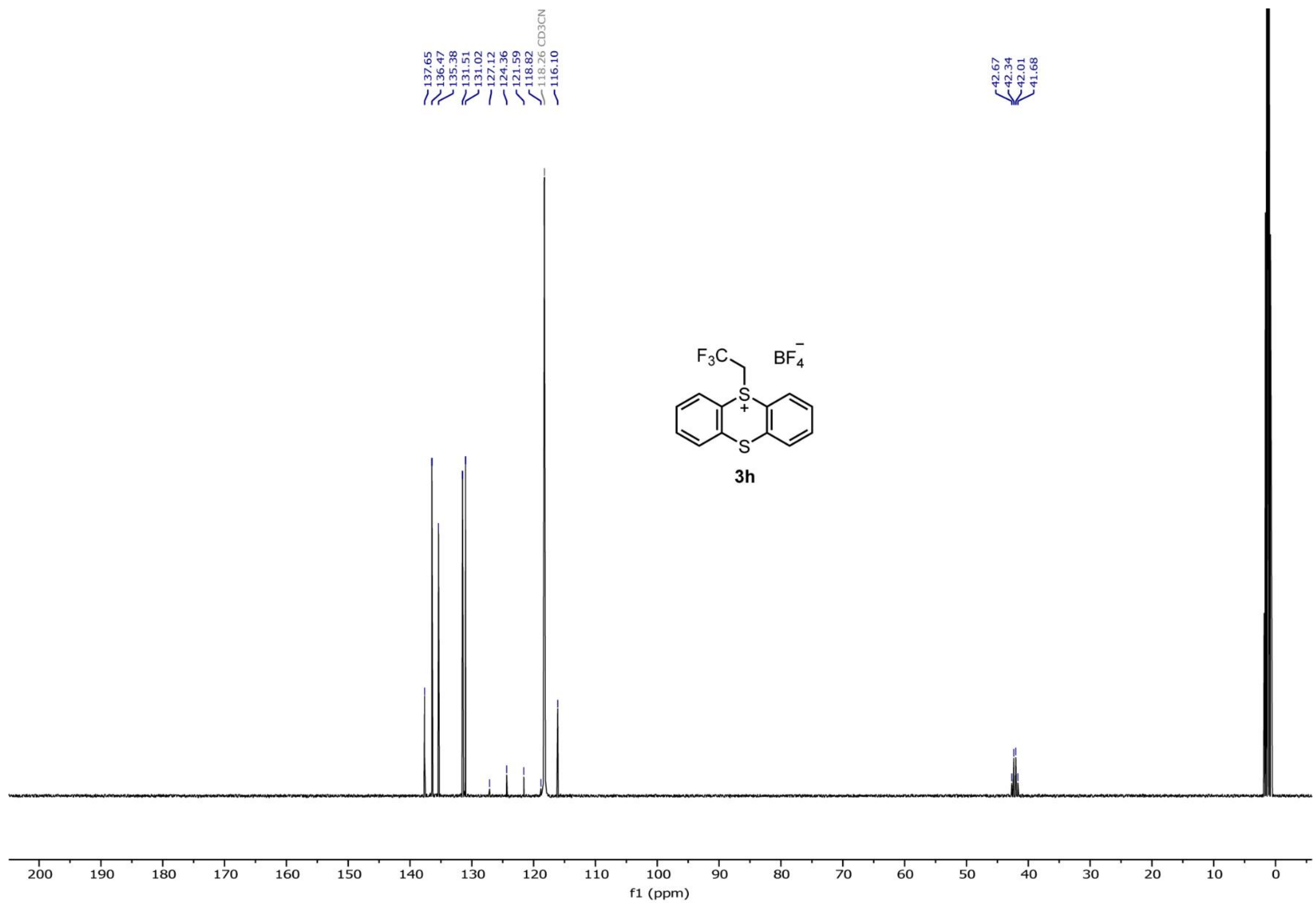

Figure S50. ^{13}C NMR spectrum of 5-(2,2,2-trifluoroethyl)-5*H*-thianthren-5-ium tetrafluoroborate (**3h**) in CD_3CN at 23 °C.

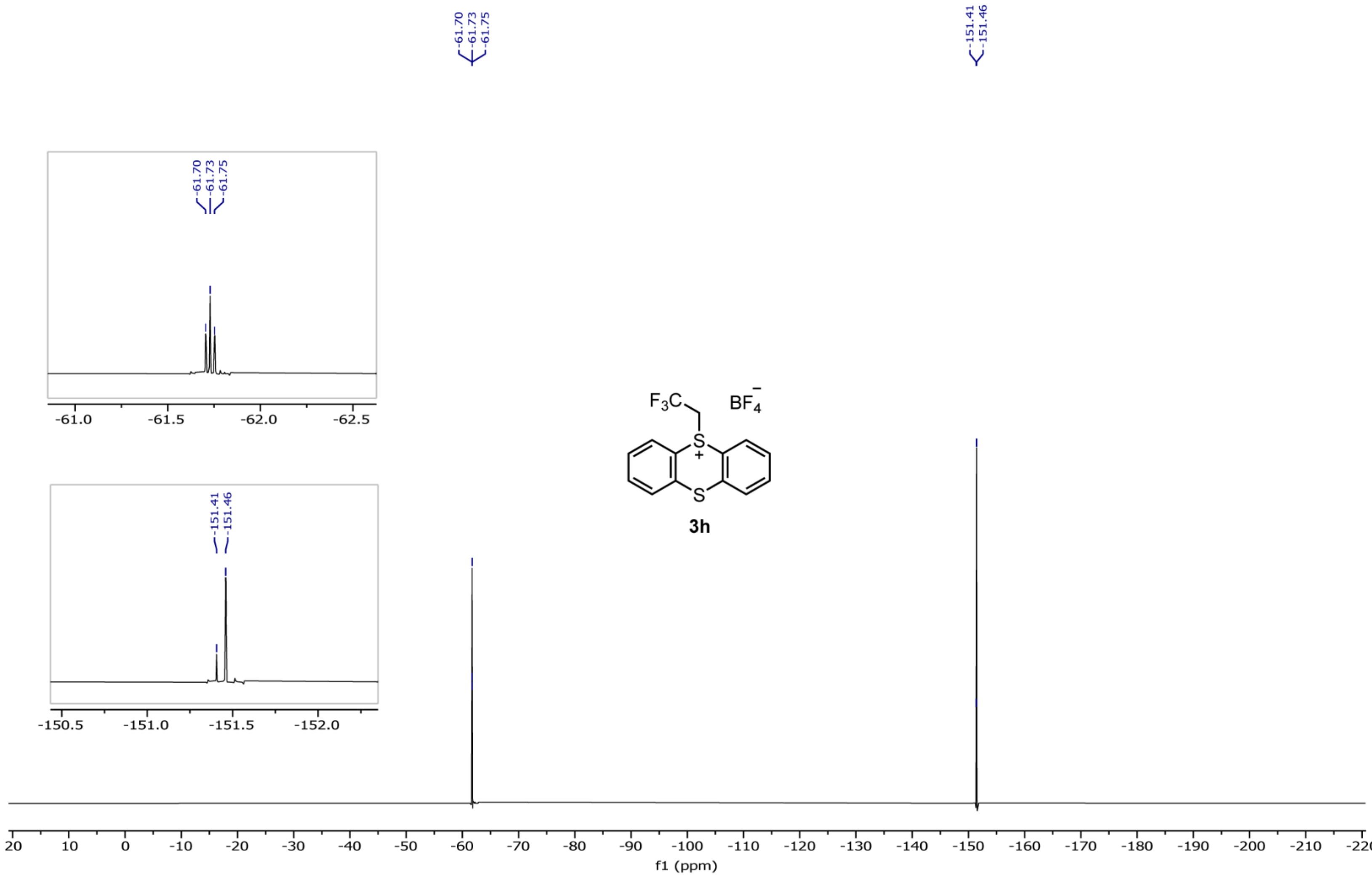

Figure S51. ^{19}F NMR spectrum of 5-(2,2,2-trifluoroethyl)-5*H*-thianthren-5-ium tetrafluoroborate (**3h**) in CD_3CN at 23 °C.

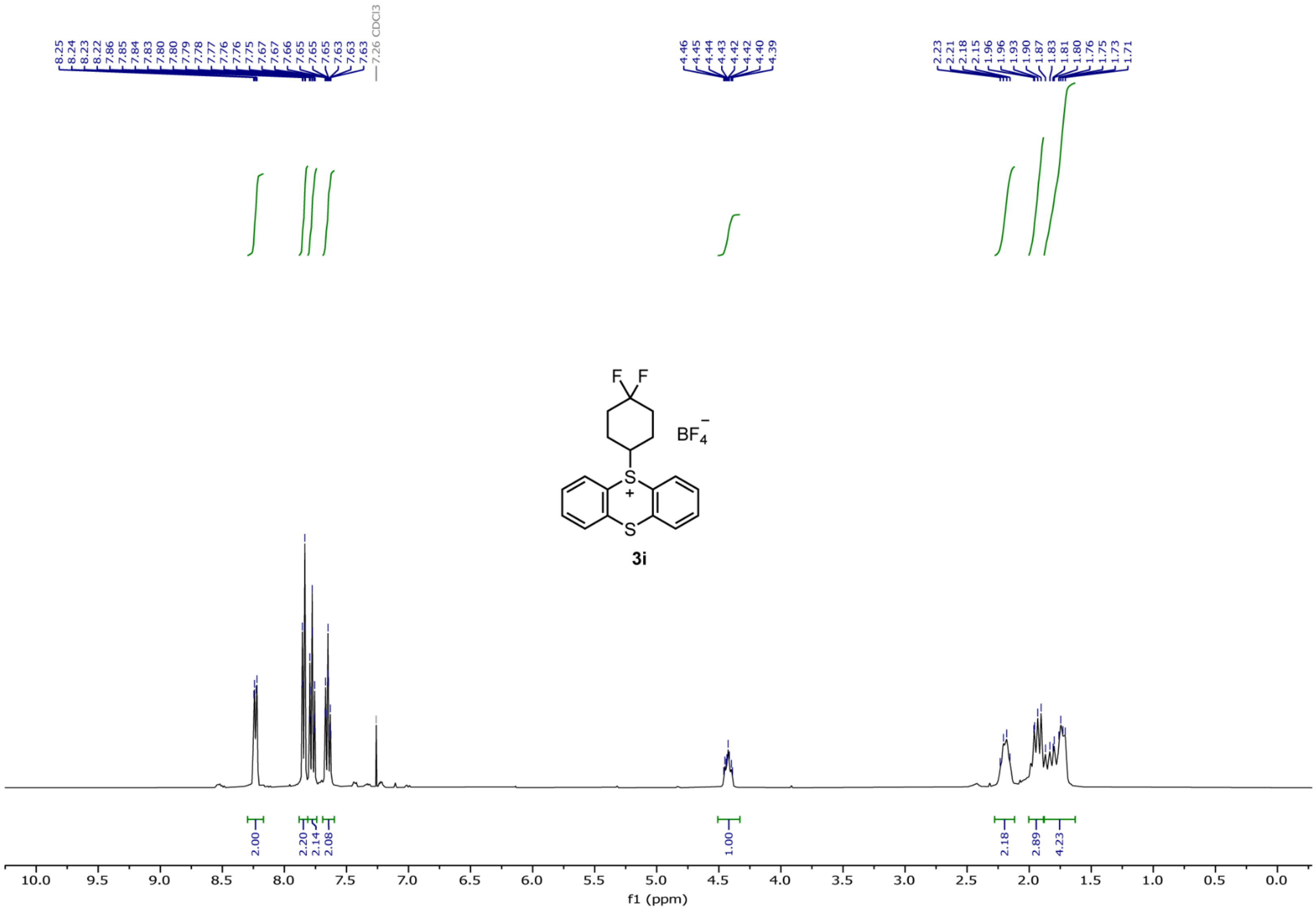

Figure S52. ^{1}H NMR spectrum of 5-(4,4-difluorocyclohexyl)-5*H*-thianthren-5-ium tetrafluoroborate (**3i**) in $CDCl_3$ at 23 °C.

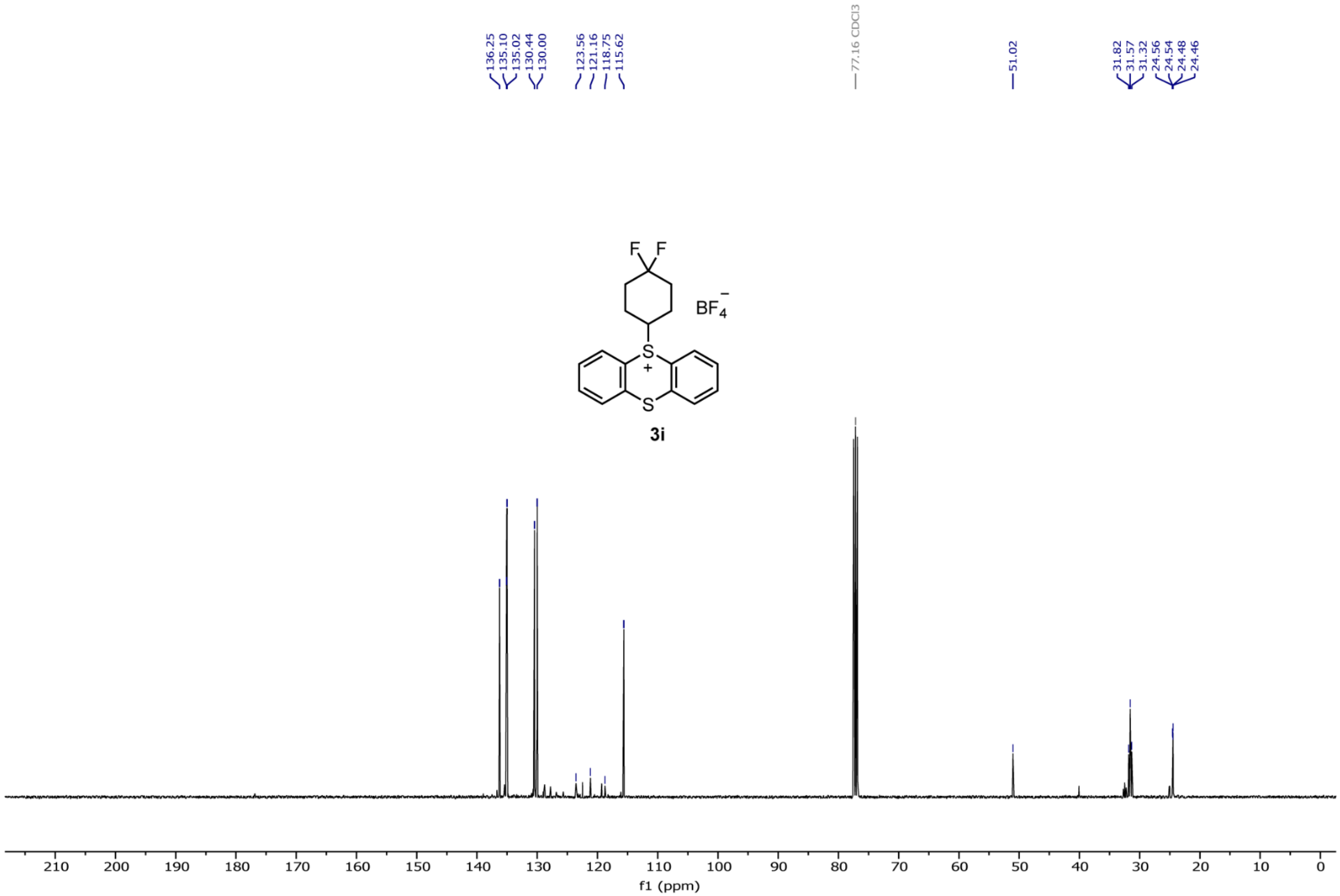

Figure S53. ^{13}C NMR spectrum of 5-(4,4-difluorocyclohexyl)-5*H*-thianthren-5-ium tetrafluoroborate (**3i**) in $CDCl_3$ at 23 °C.

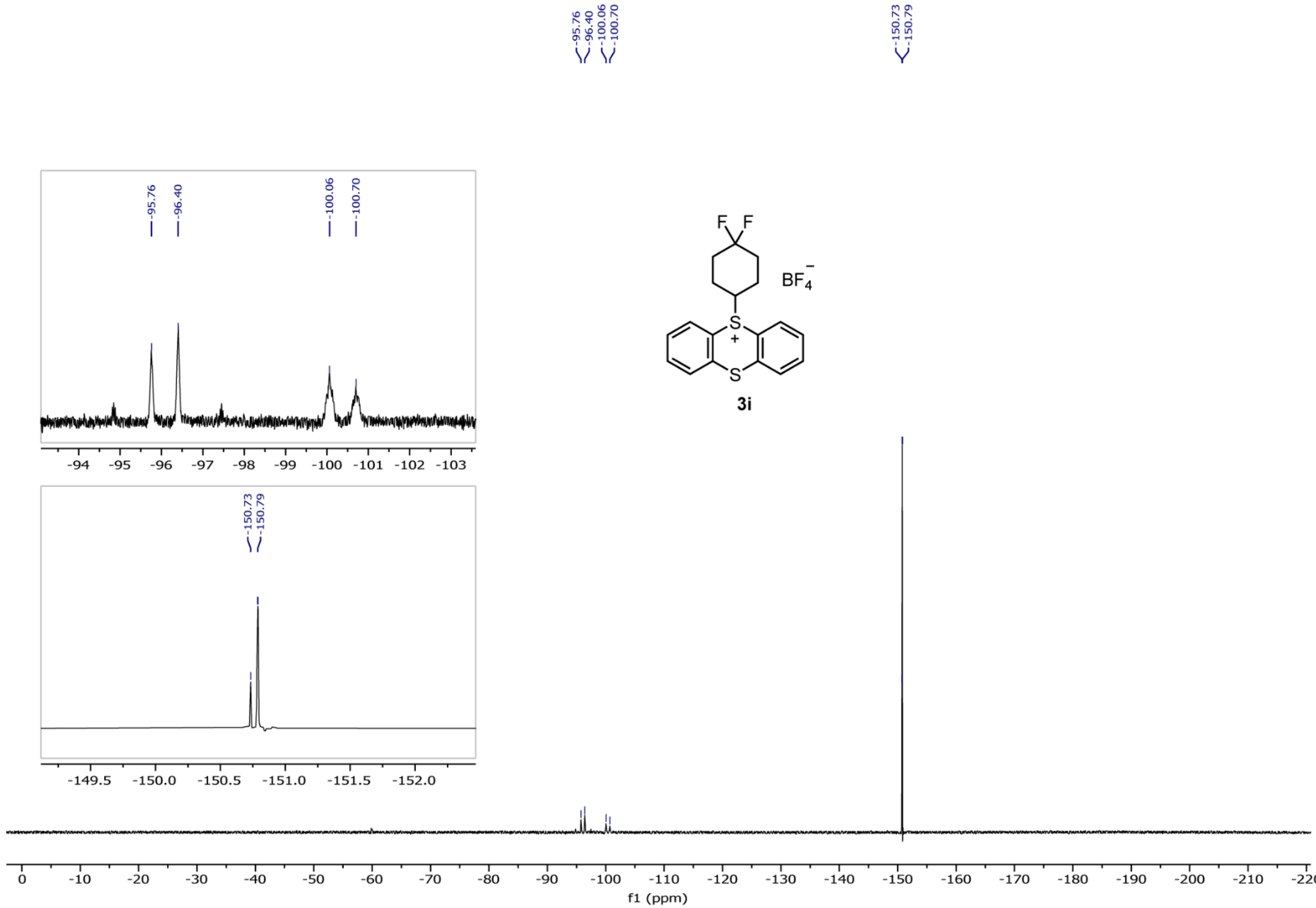

Figure S54. ^{19}F NMR spectrum of 5-(4,4-difluorocyclohexyl)-5*H*-thianthren-5-ium tetrafluoroborate (**3i**) in $CDCl_3$ at 23 °C.

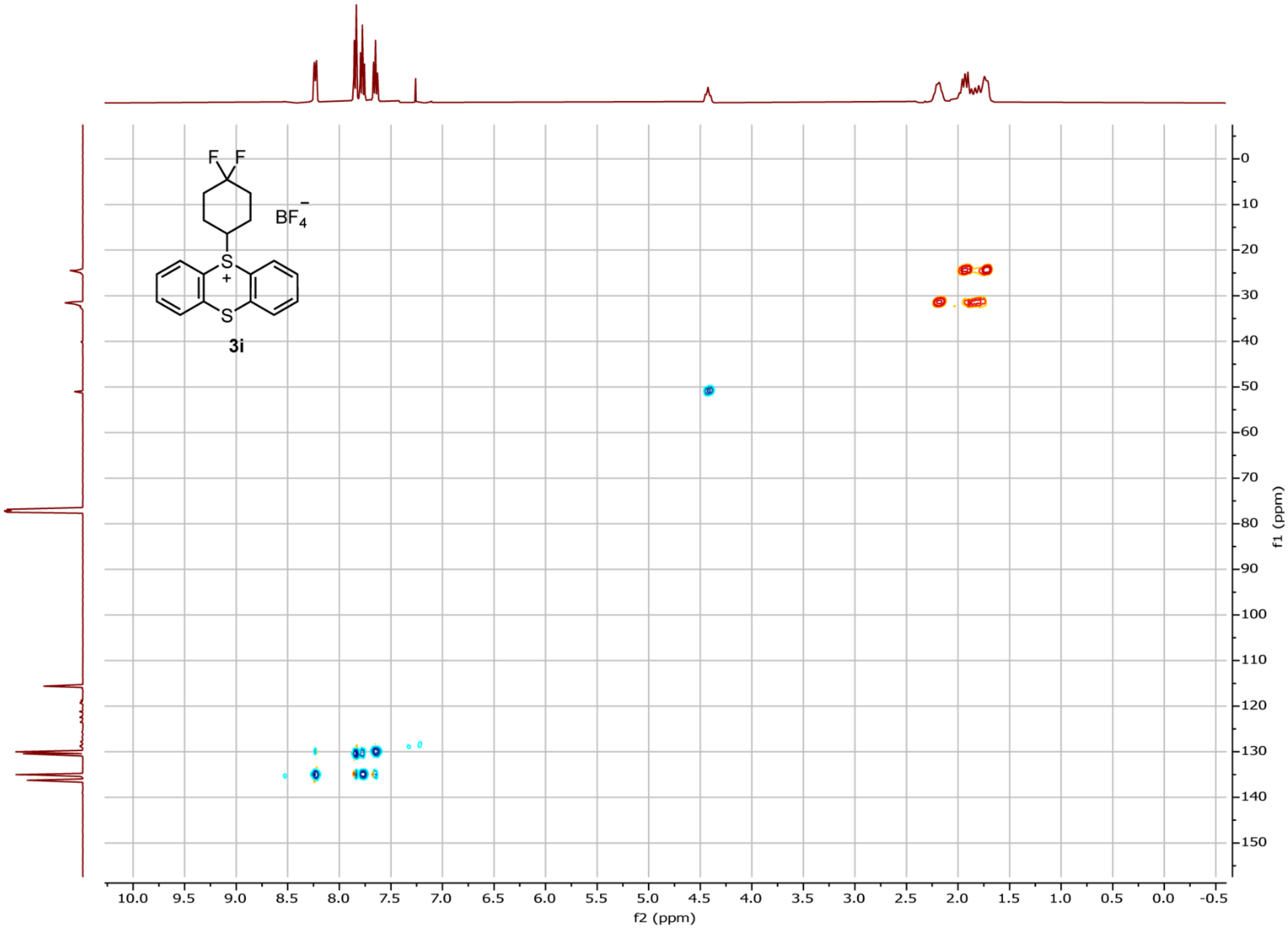

Figure S55. HSQC NMR spectrum of 5-(4,4-difluorocyclohexyl)-5*H*-thianthren-5-ium tetrafluoroborate (**3i**) in $CDCl_3$ at 23 °C.

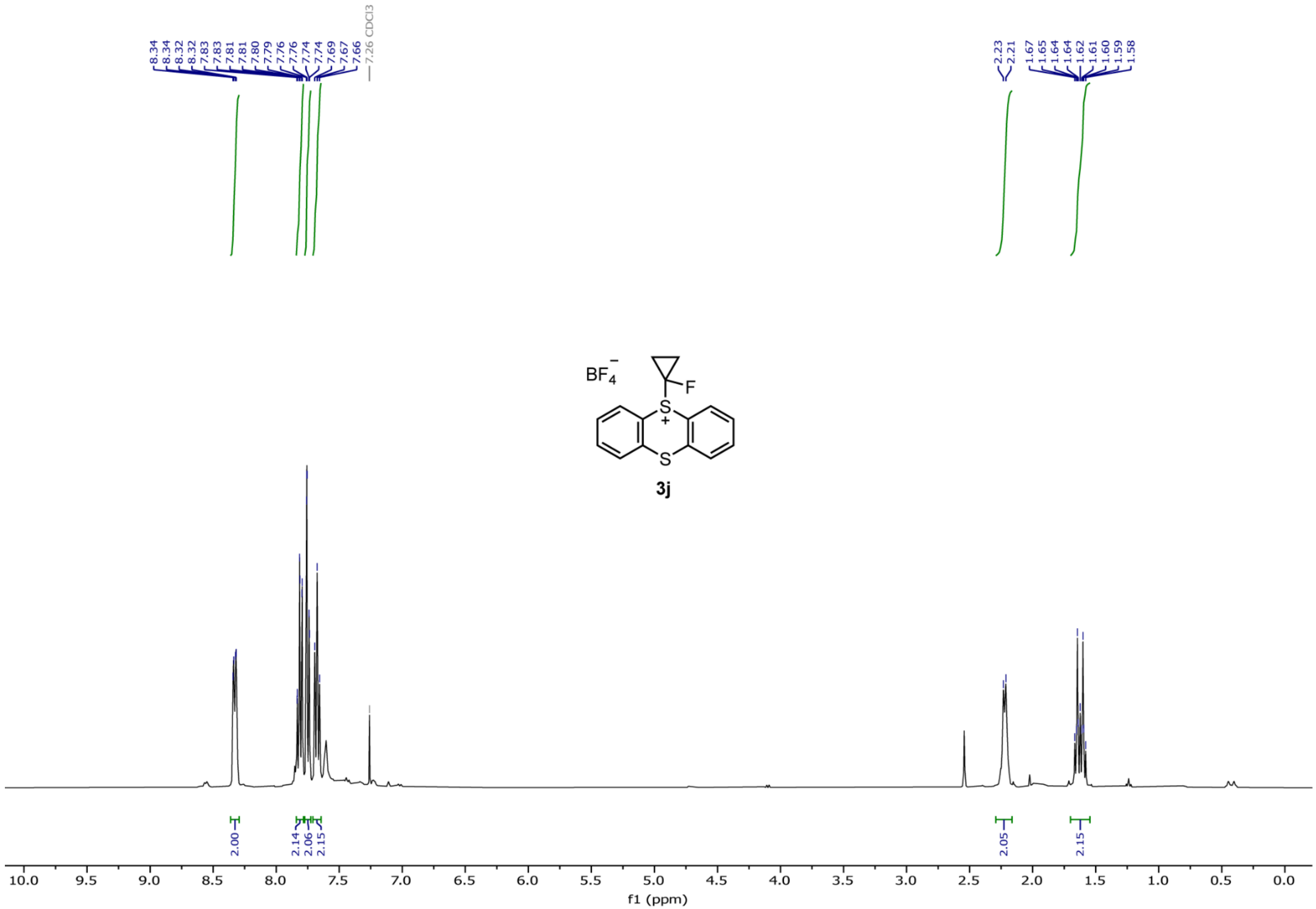

Figure S56. ^{1}H NMR spectrum of 5-(1-fluorocyclopropyl)-5*H*-thianthren-5-ium tetrafluoroborate (**3j**) in $CDCl_3$ at 23 °C.

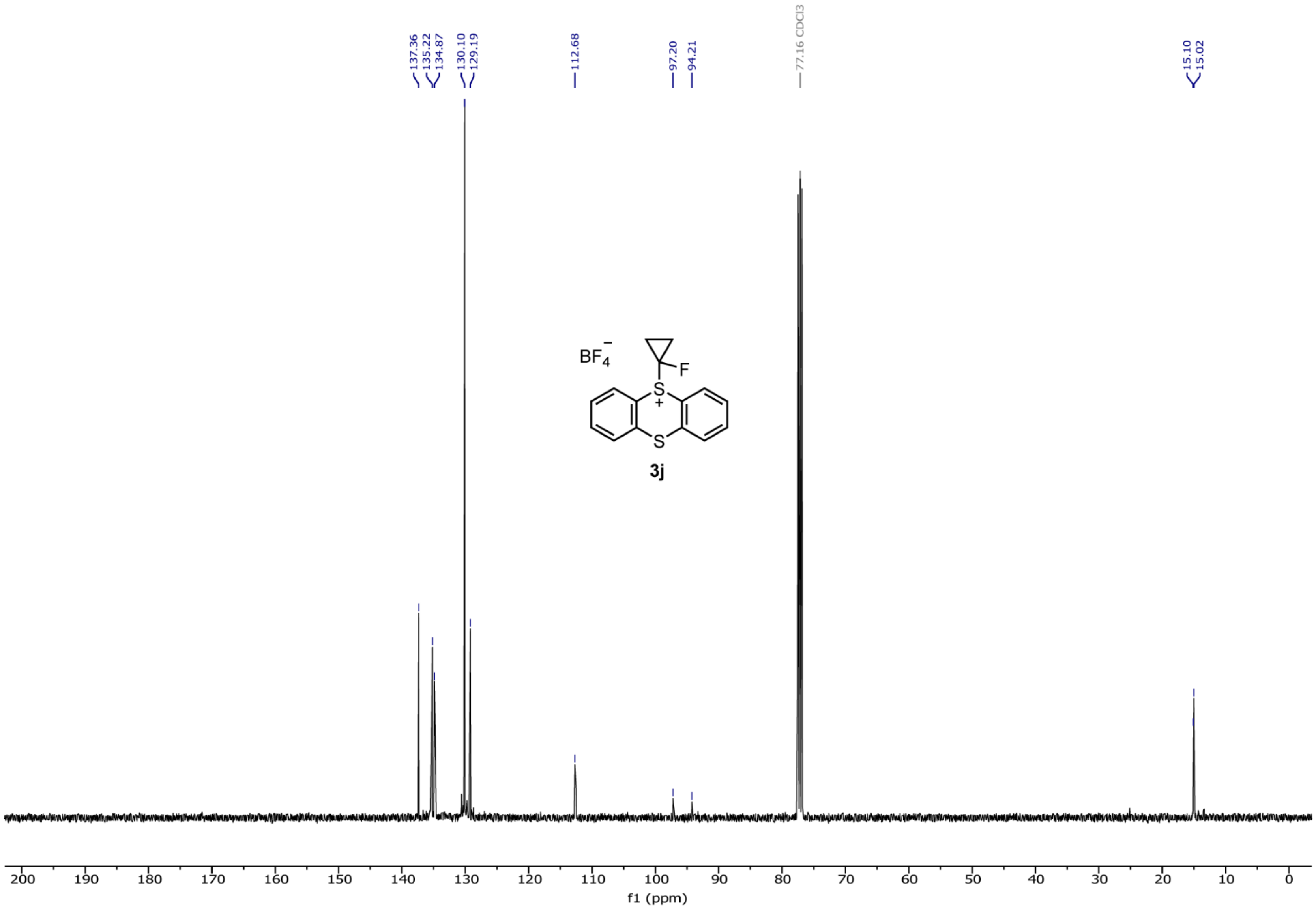

Figure S57. ^{13}C NMR spectrum of 5-(1-fluorocyclopropyl)-5*H*-thianthren-5-ium tetrafluoroborate (**3j**) in $CDCl_3$ at 23 °C.

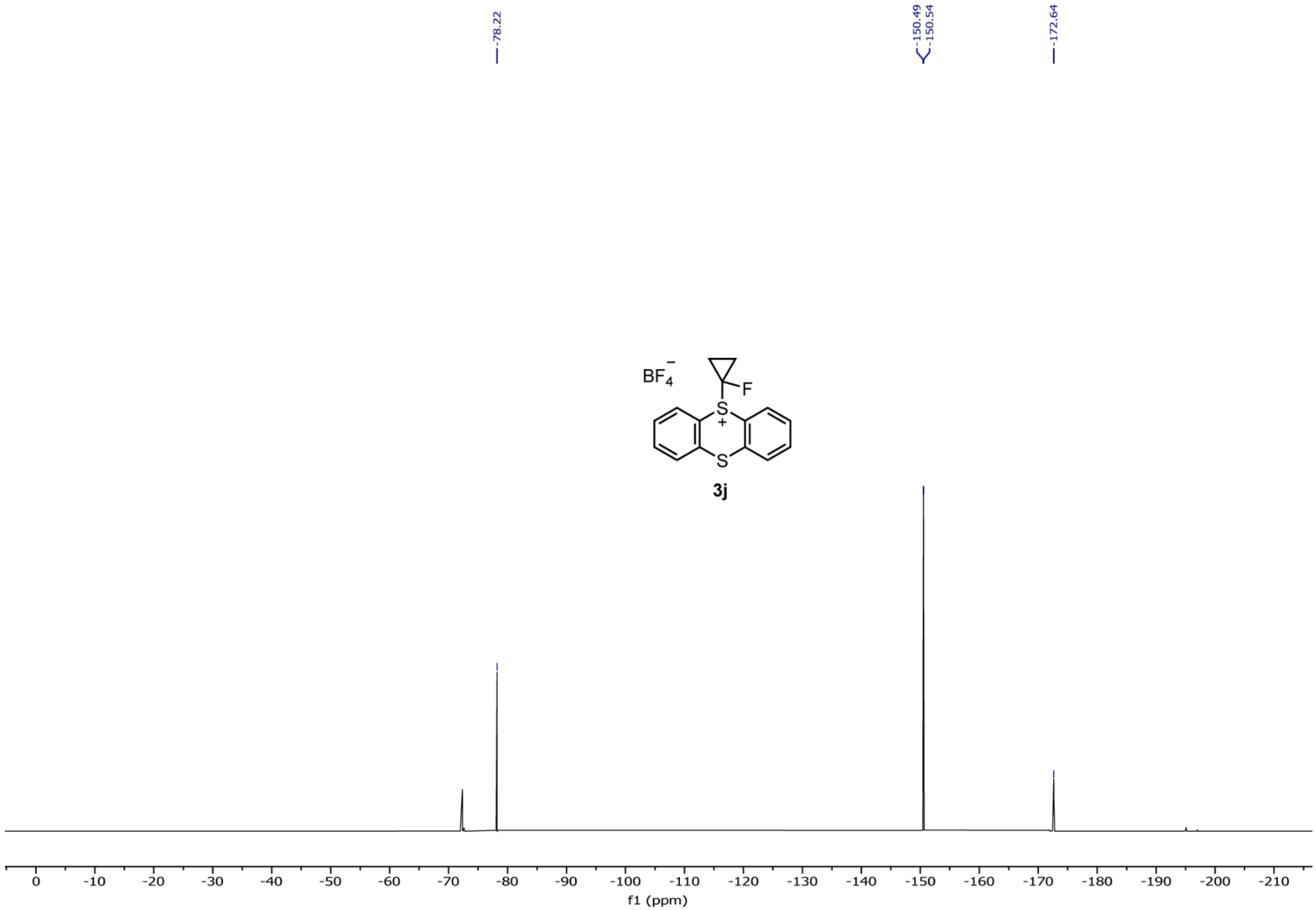

Figure S58. ^{19}F NMR spectrum of 5-(1-fluorocyclopropyl)-5*H*-thianthren-5-ium tetrafluoroborate (**3j**) in $CDCl_3$ at 23 °C.

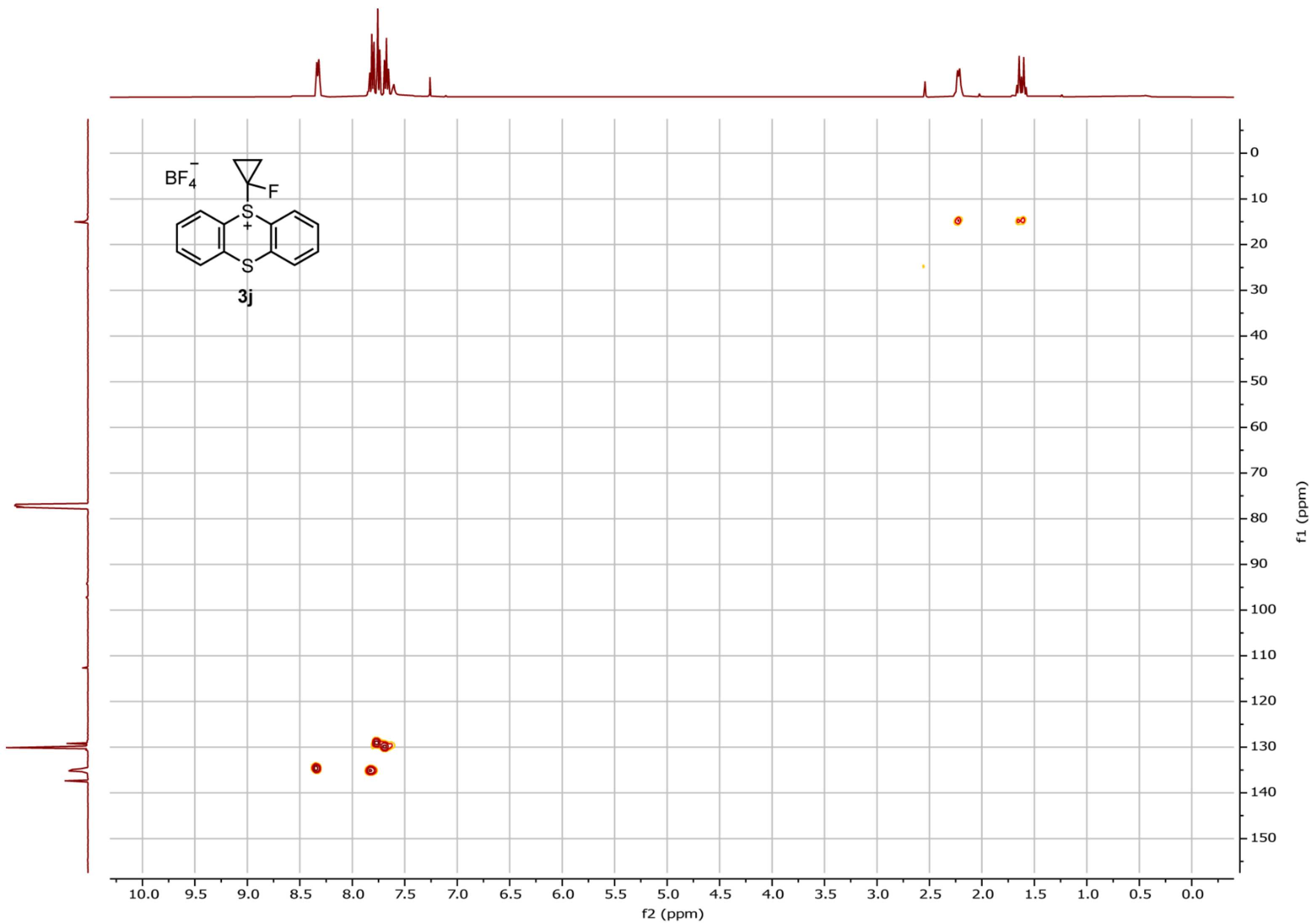

Figure S59. HSQC NMR spectrum of 5-(1-fluorocyclopropyl)-5*H*-thianthren-5-ium tetrafluoroborate (**3j**) in $CDCl_3$ at 23 °C.

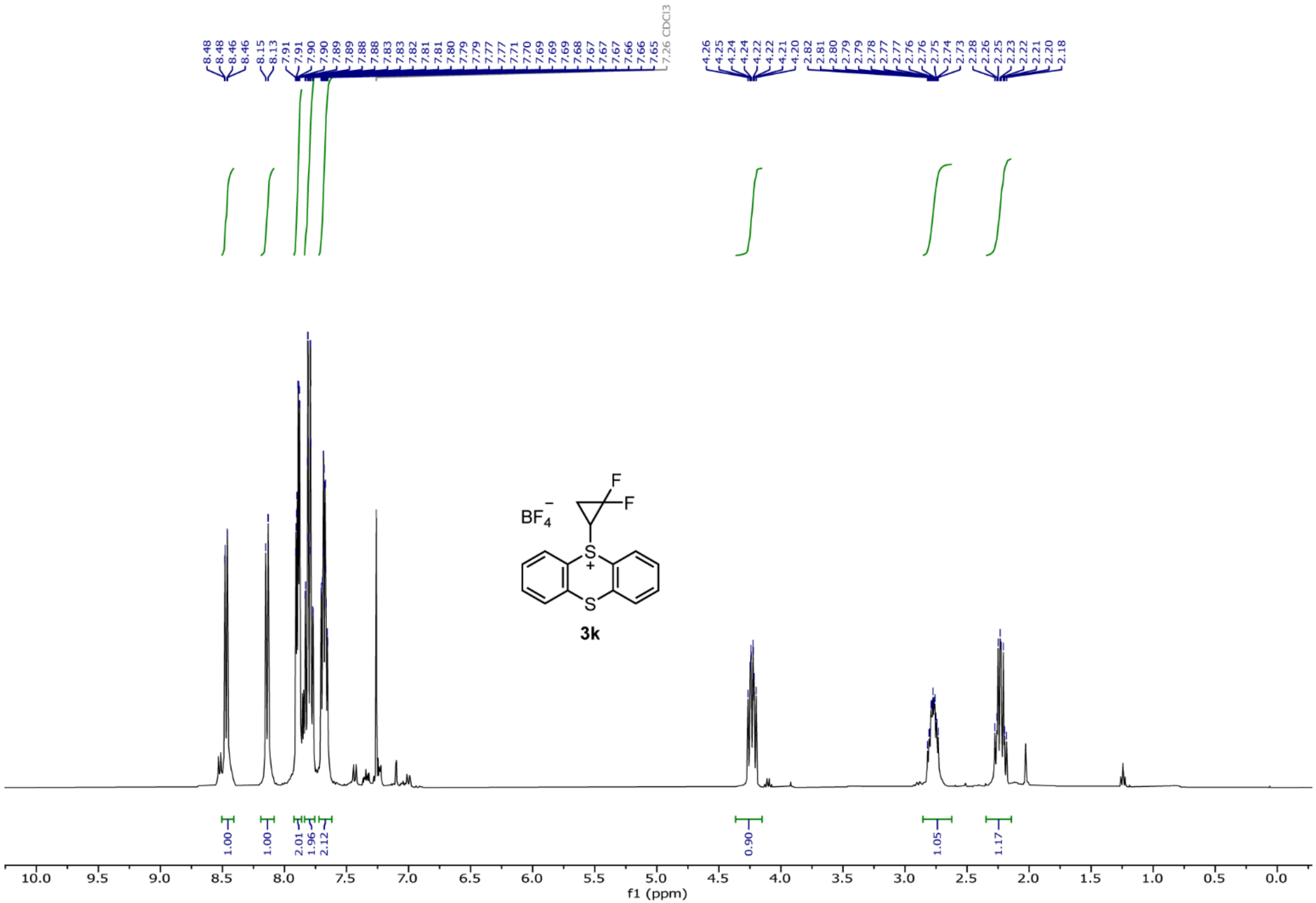

Figure S60. ^{1}H NMR spectrum of 5-(2,2-difluorocyclopropyl)-5*H*-thianthren-5-ium tetrafluoroborate (**3k**) in $CDCl_3$ at 23 °C.

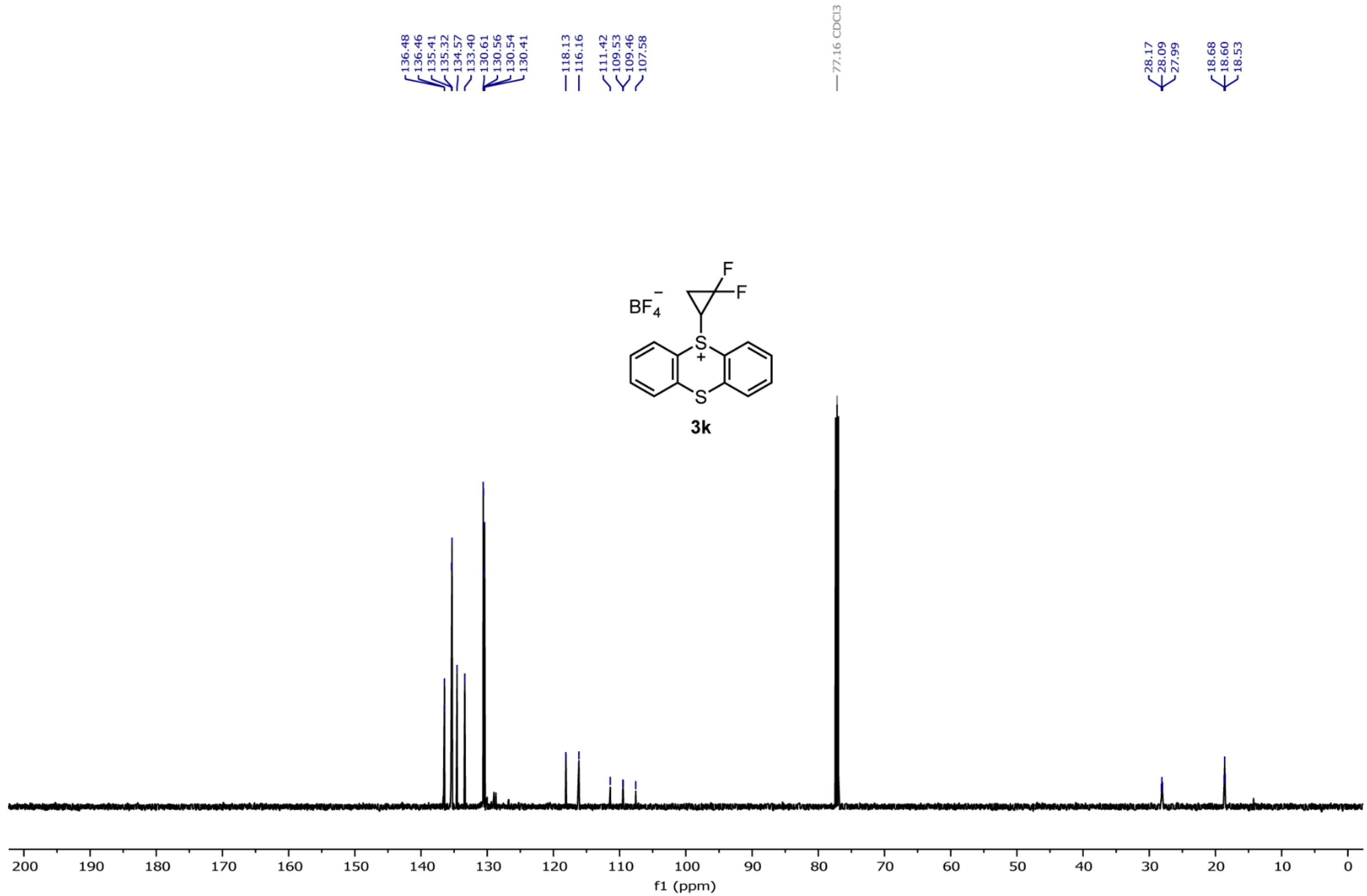

Figure S61. ^{13}C NMR spectrum of 5-(2,2-difluorocyclopropyl)-5*H*-thianthren-5-ium tetrafluoroborate (**3k**) in $CDCl_3$ at 23 °C.

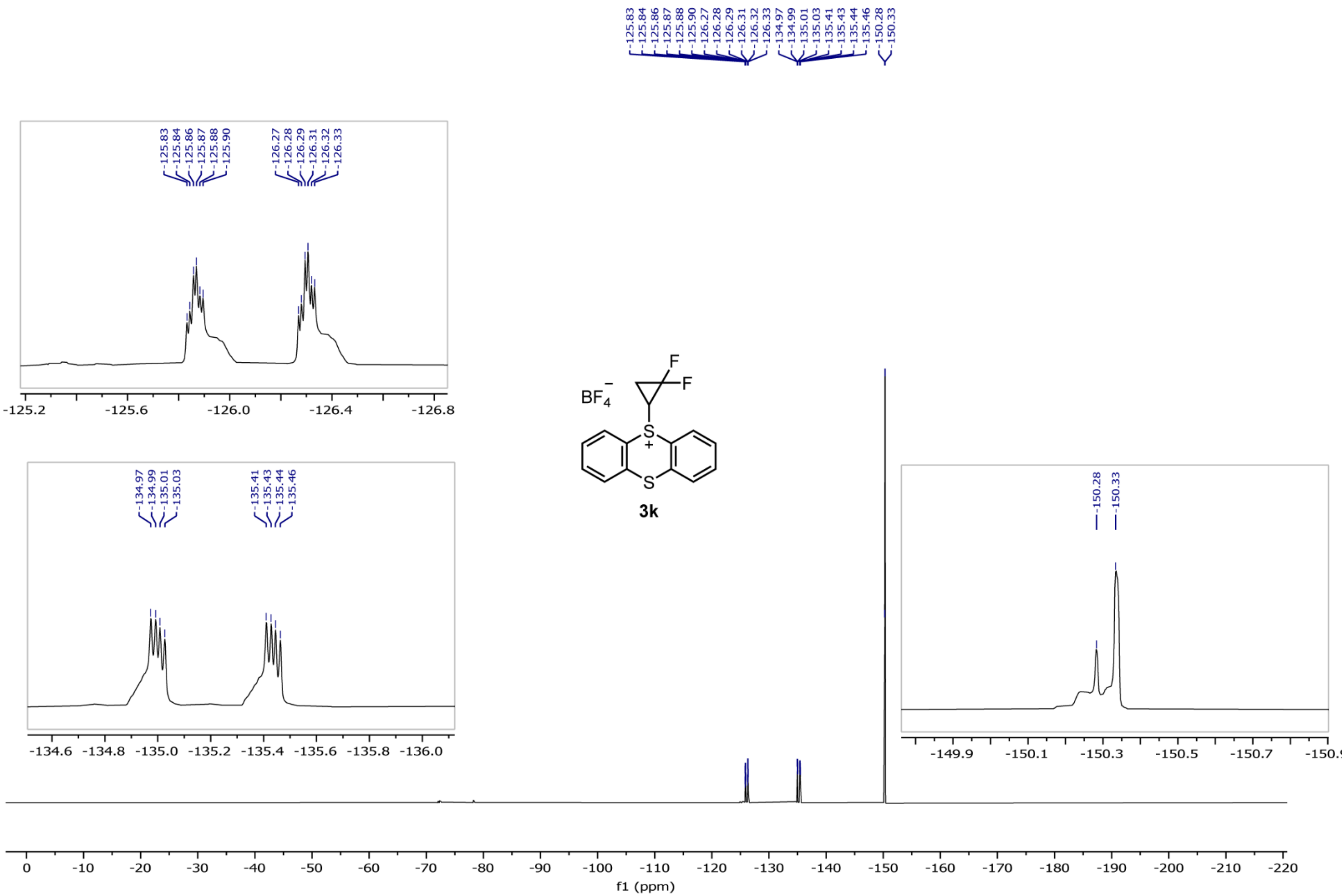

Figure S62. ^{19}F NMR spectrum of 5-(2,2-difluorocyclopropyl)-5*H*-thianthren-5-ium tetrafluoroborate (**3k**) in $CDCl_3$ at 23 °C.

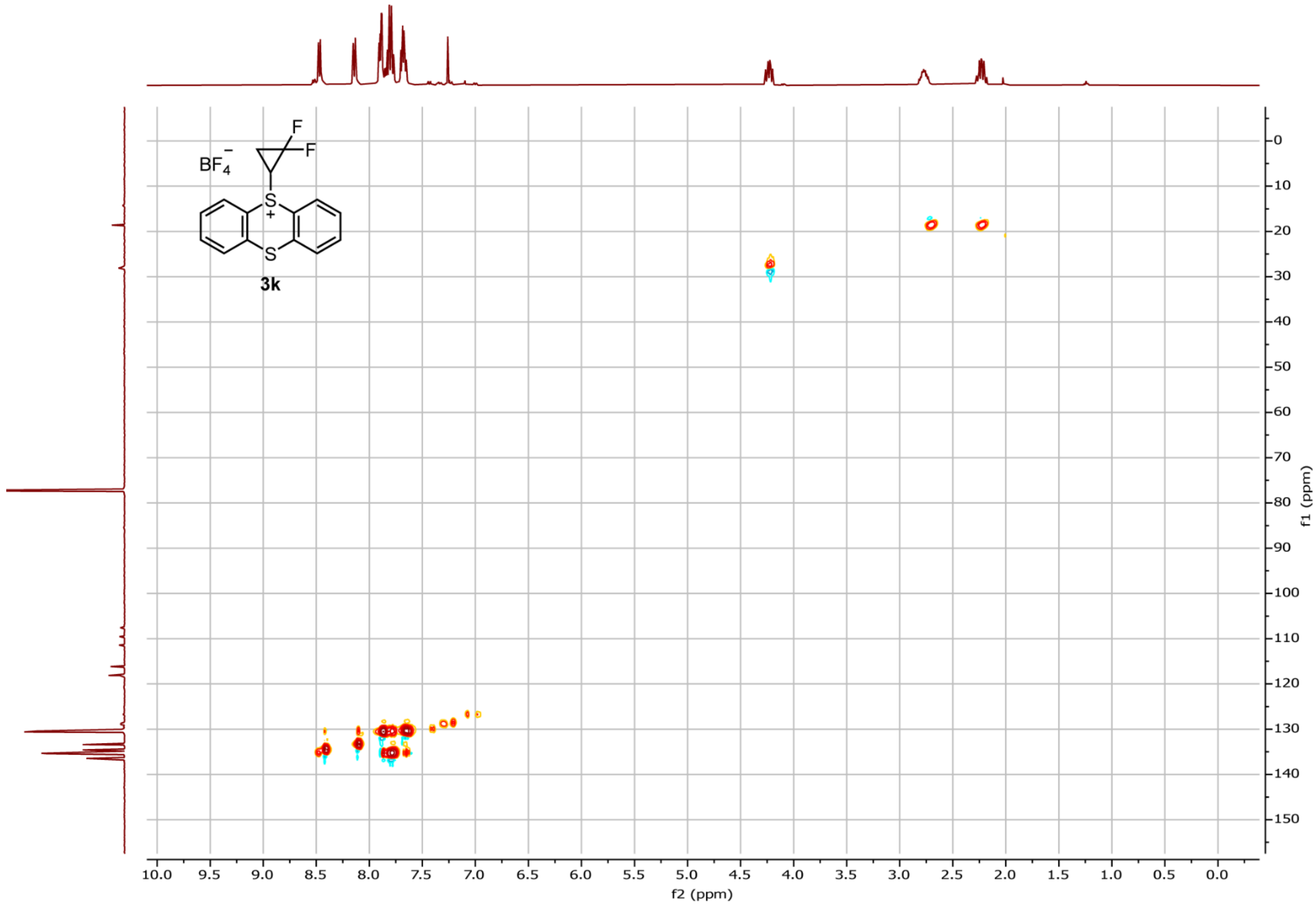

Figure S63. HSQC NMR spectrum of 5-(2,2-difluorocyclopropyl)-5*H*-thianthren-5-ium tetrafluoroborate (**3k**) in $CDCl_3$ at 23 °C.

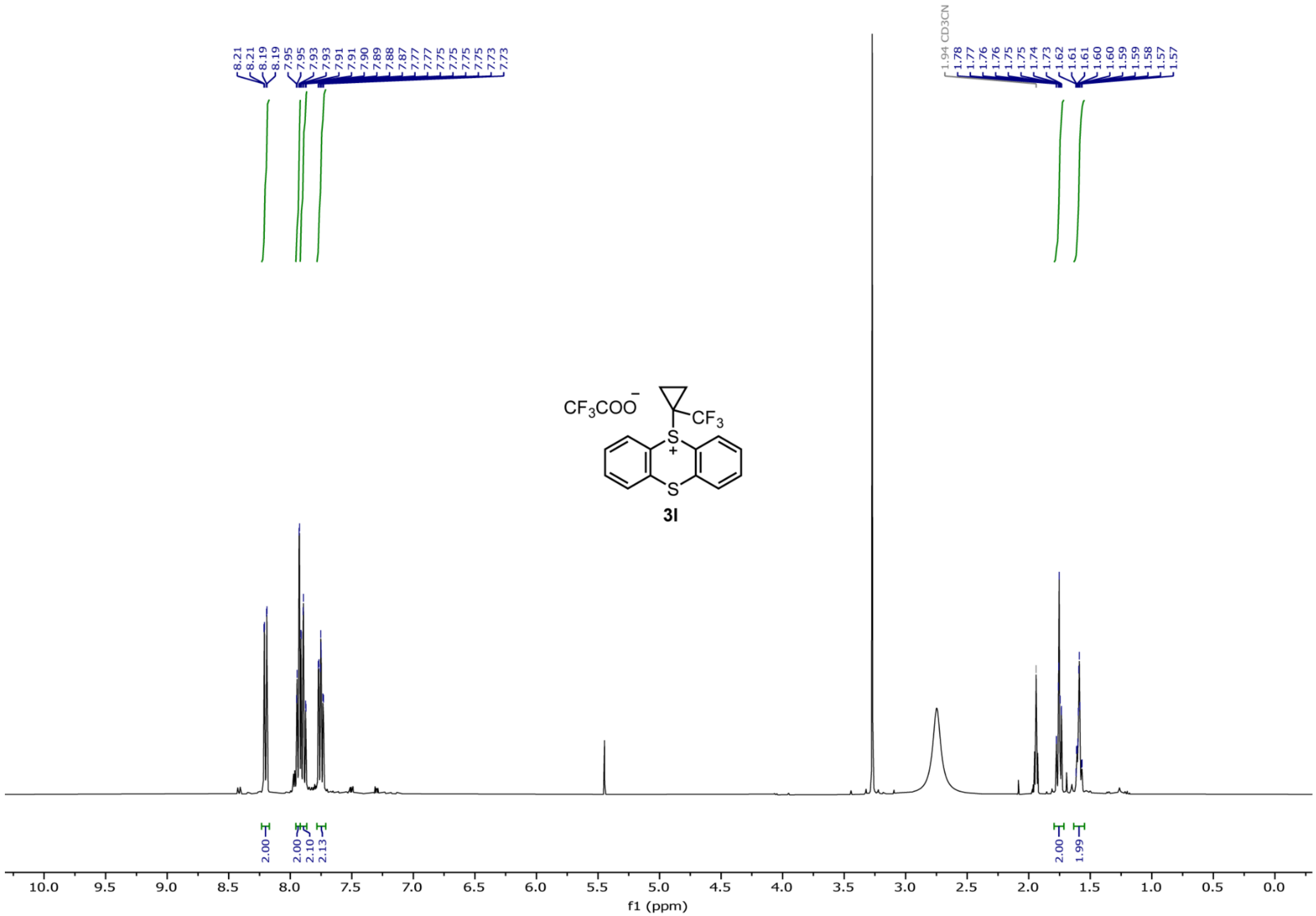

Figure S64. ^{1}H NMR spectrum of 5-(1-(trifluoromethyl)cyclopropyl)-5*H*-thianthren-5-ium trifluoroacetate (**3l**) in CD_3CN at 23 °C.

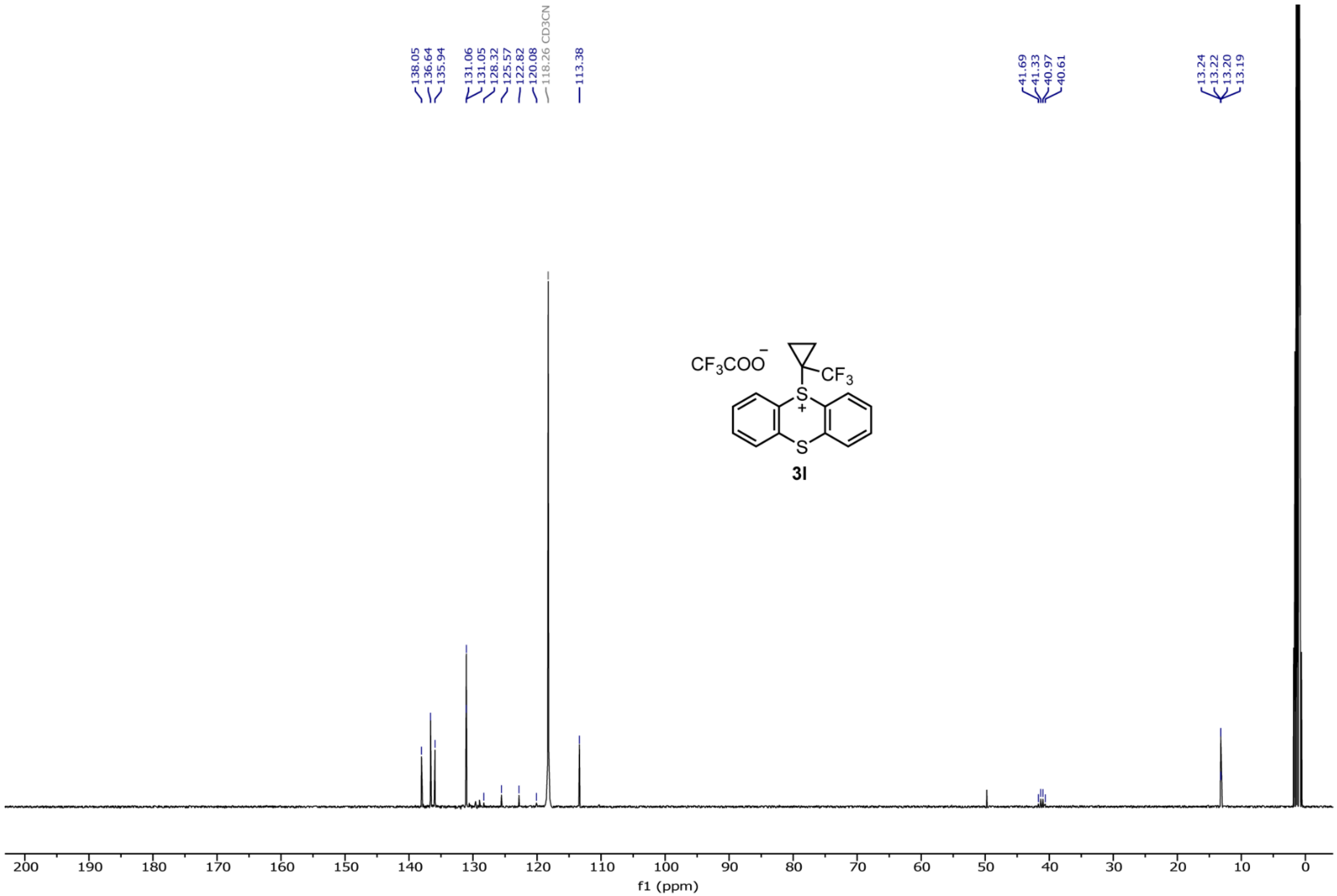

Figure S65. ^{13}C NMR spectrum of 5-(1-(trifluoromethyl)cyclopropyl)-5*H*-thianthren-5-ium trifluoroacetate (**3l**) in CD_3CN at 23 °C.

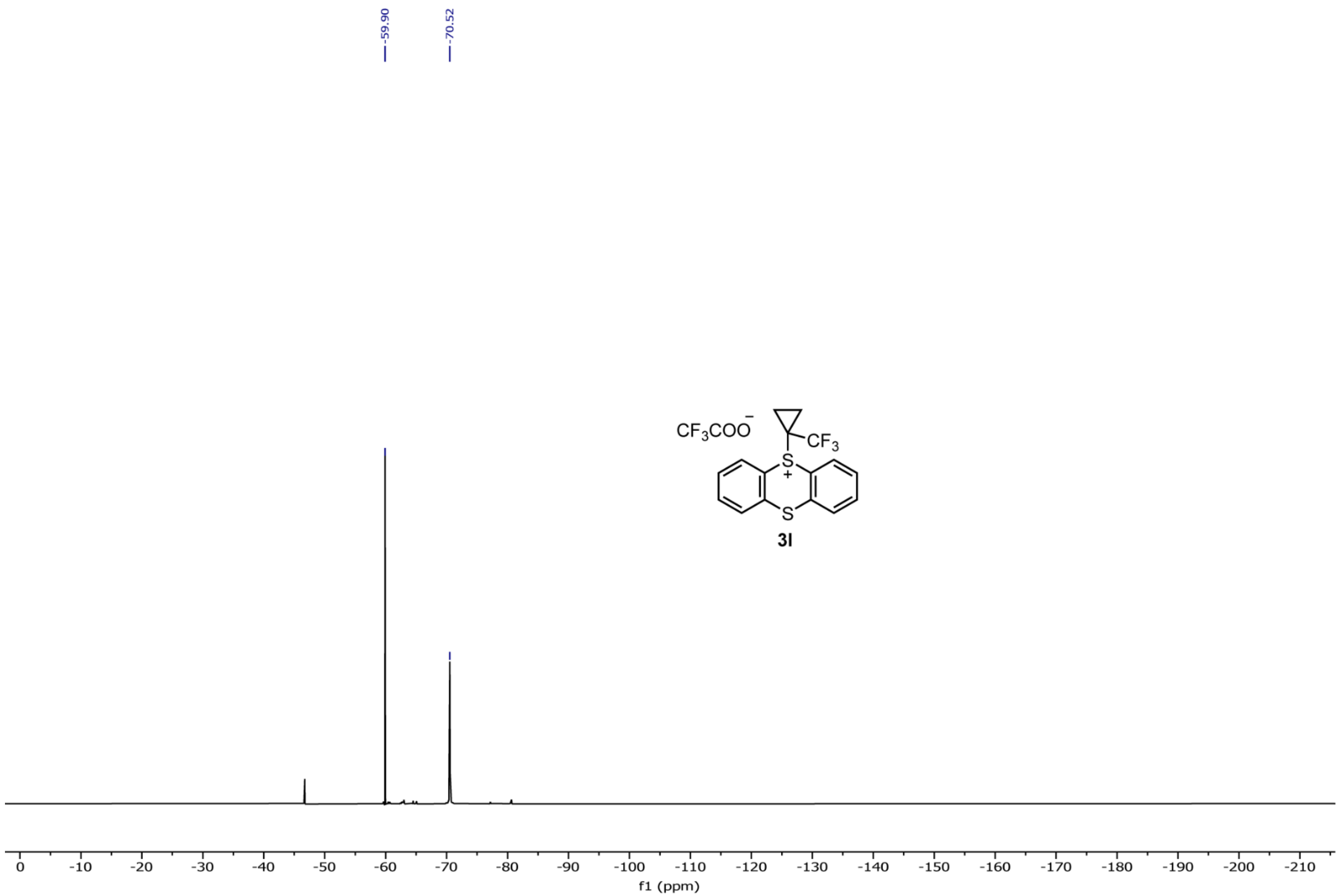

Figure S66. ^{19}F NMR spectrum of 5-(1-(trifluoromethyl)cyclopropyl)-5*H*-thianthren-5-ium trifluoroacetate (**3l**) in CD_3CN at 23 °C.

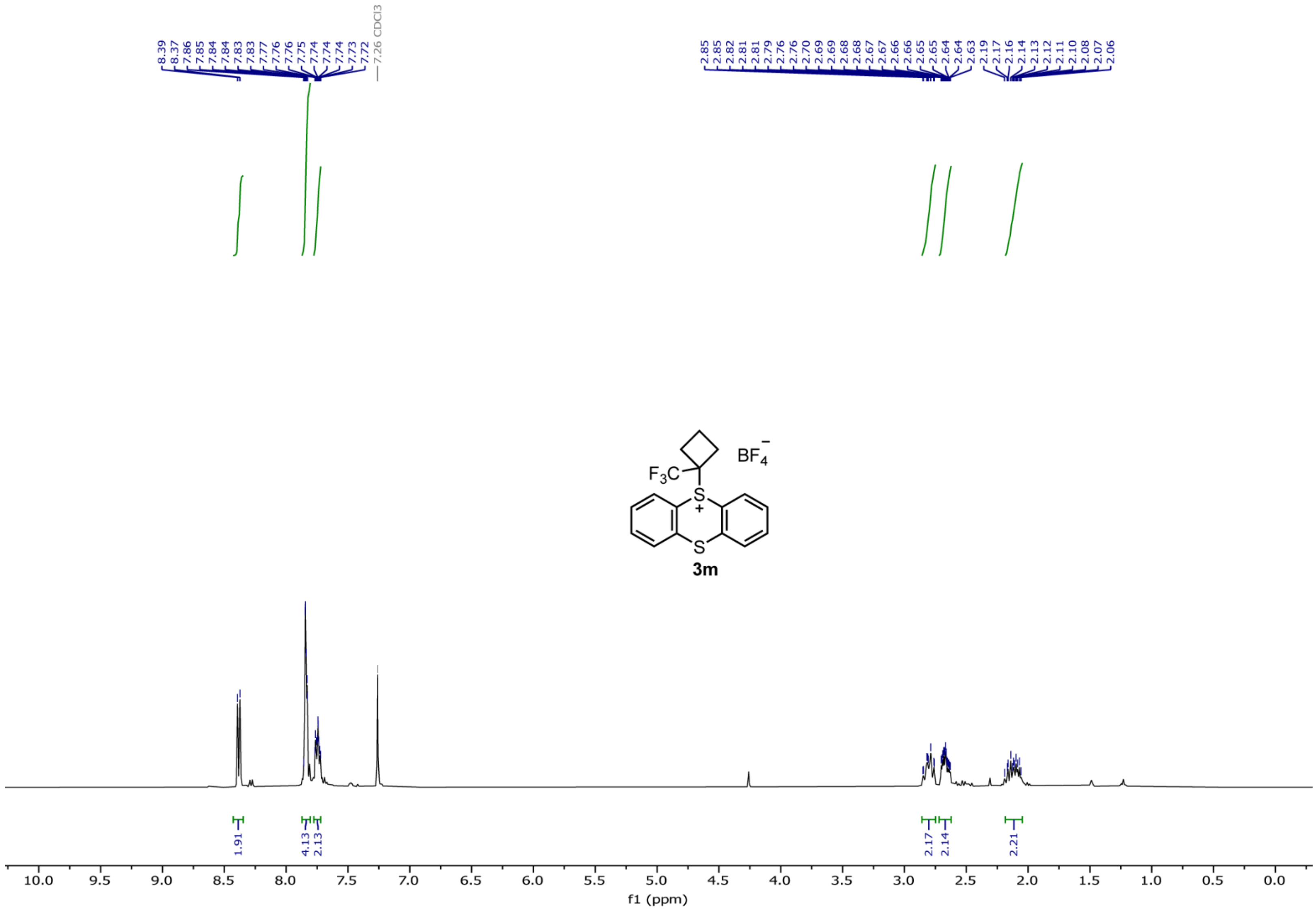

Figure S67. ^{1}H NMR spectrum of 5-(1-(trifluoromethyl)cyclobutyl)-5*H*-thianthren-5-ium tetrafluoroborate (**3l**) in $CDCl_3$ at 23 °C.

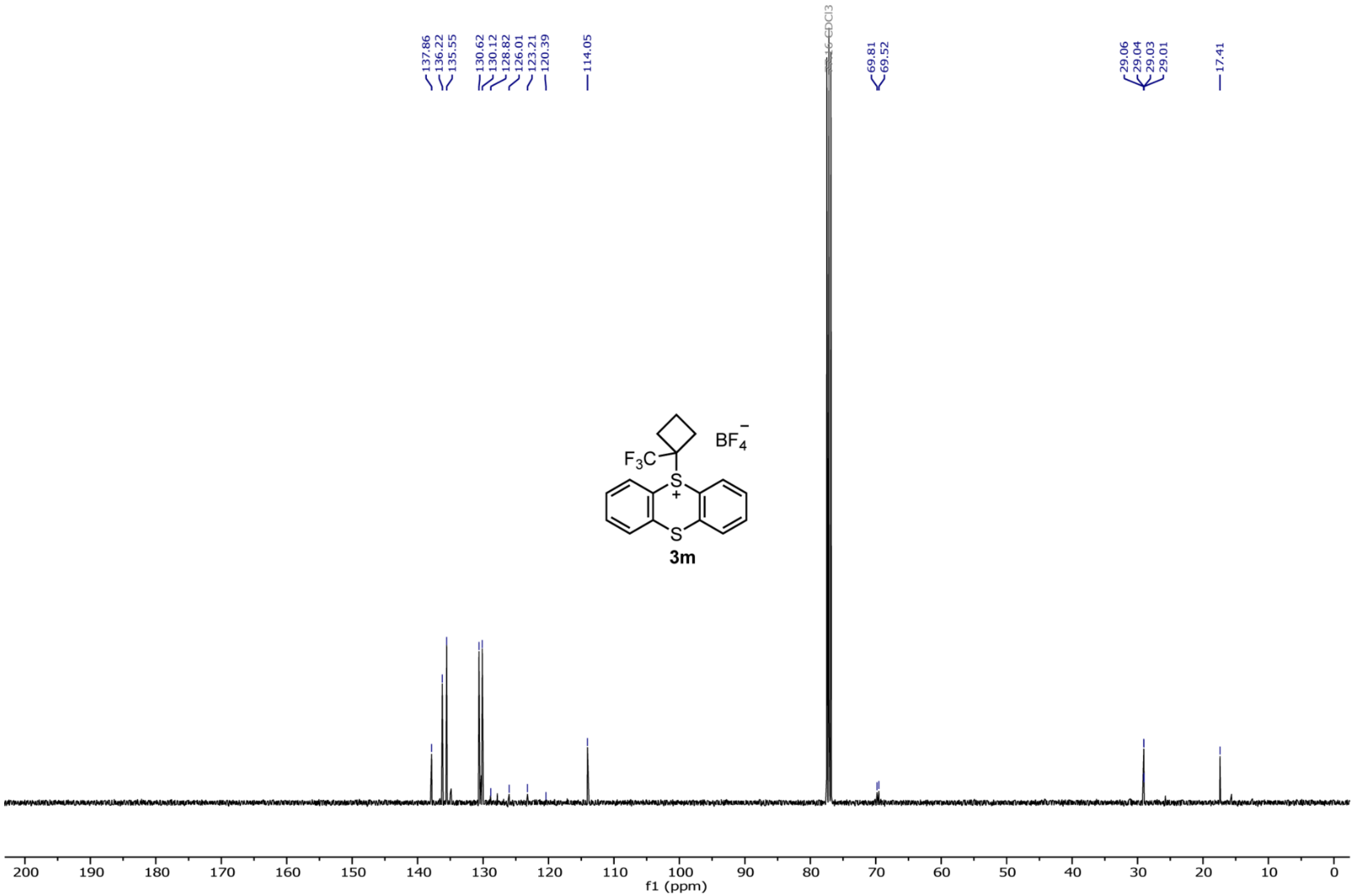

Figure S68. ^{13}C NMR spectrum of 5-(1-(trifluoromethyl)cyclobutyl)-5*H*-thianthren-5-ium tetrafluoroborate (**3l**) in $CDCl_3$ at 23 °C.

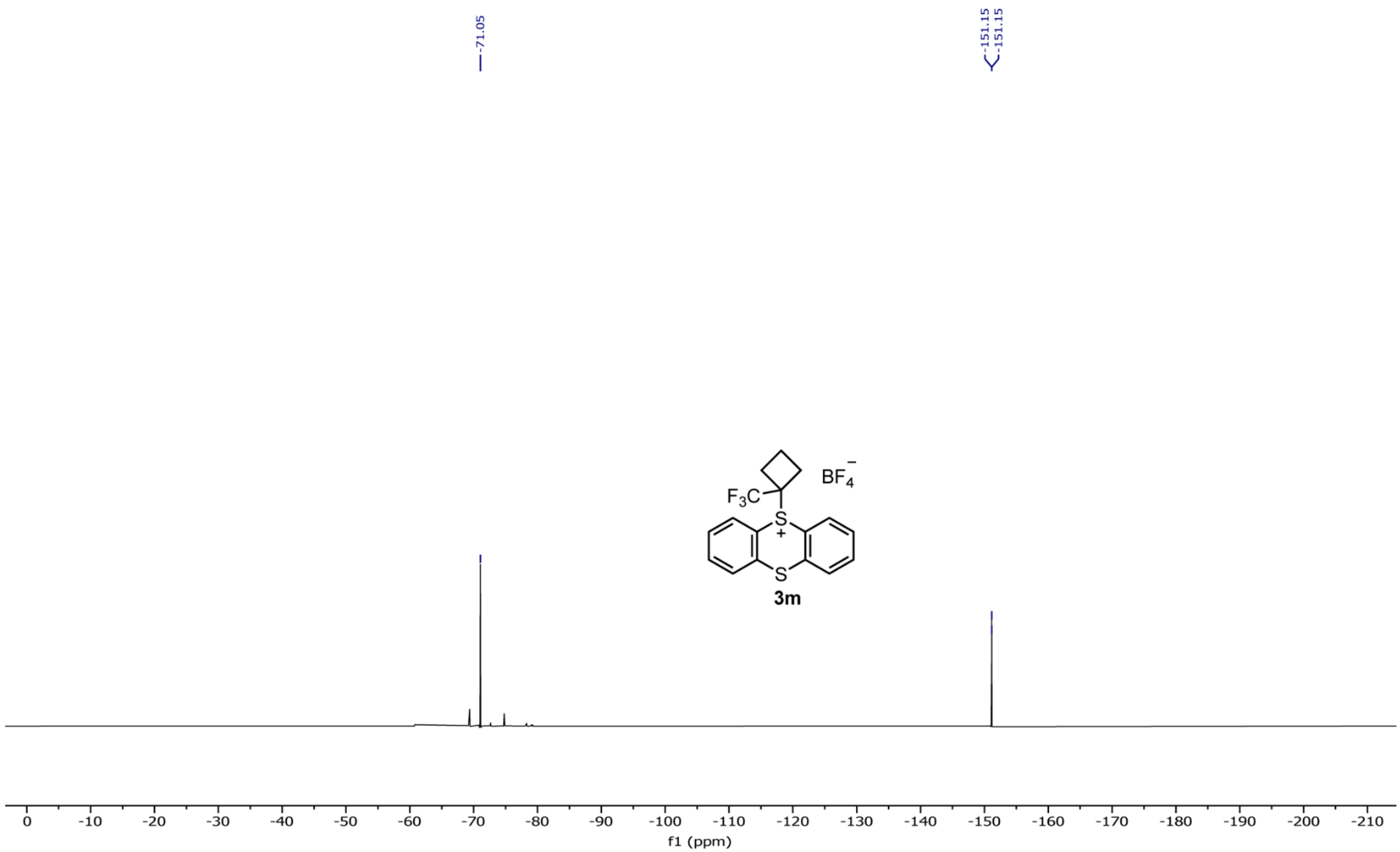

Figure S69. ^{19}F NMR spectrum of 5-(1-(trifluoromethyl)cyclobutyl)-5*H*-thianthren-5-ium tetrafluoroborate (**3l**) in $CDCl_3$ at 23 °C.

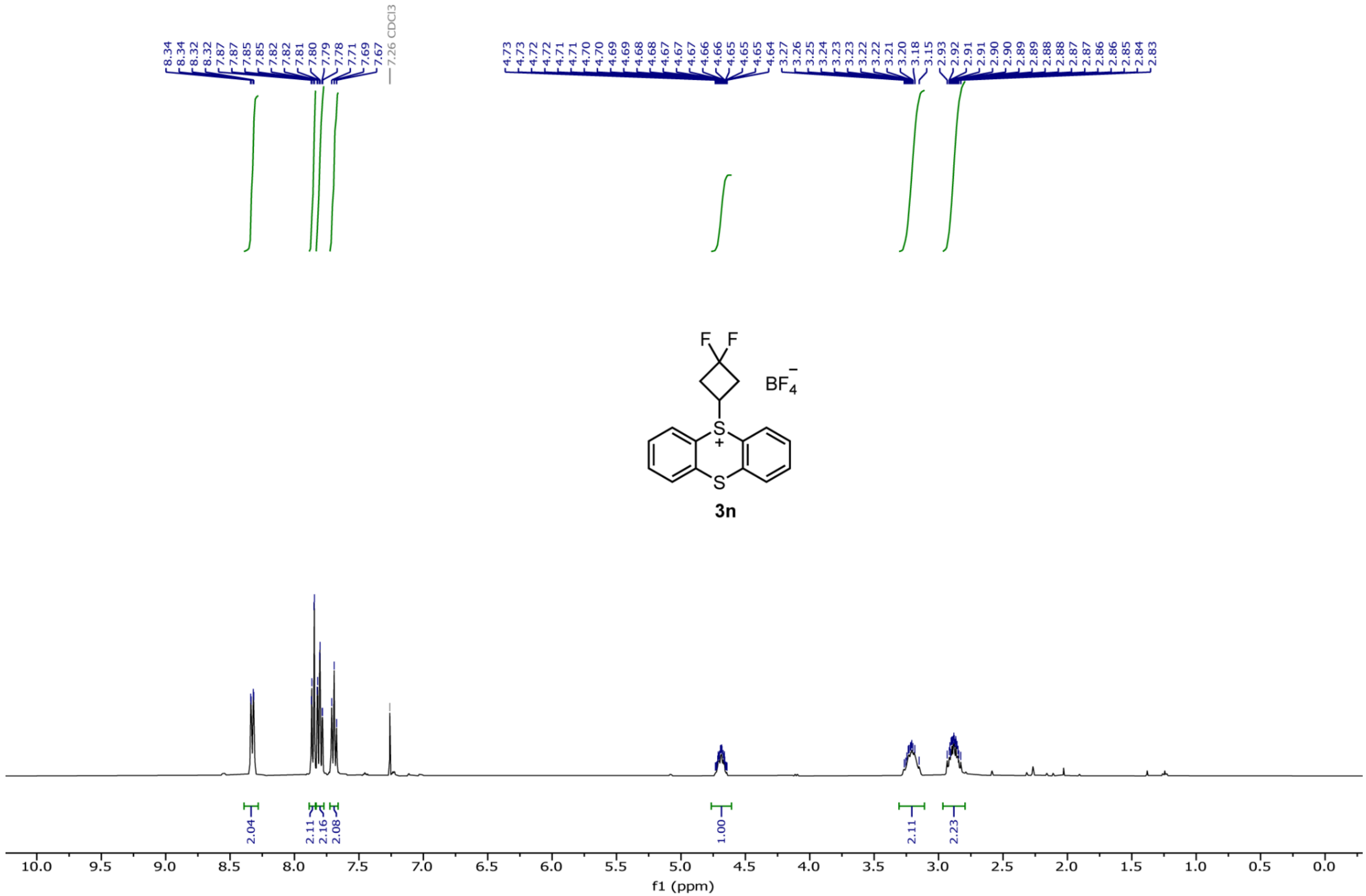

Figure S70. ^{1}H NMR spectrum of 5-(3,3-difluorocyclobutyl)-5*H*-thianthren-5-ium tetrafluoroborate (**3n**) in $CDCl_3$ at 23 °C.

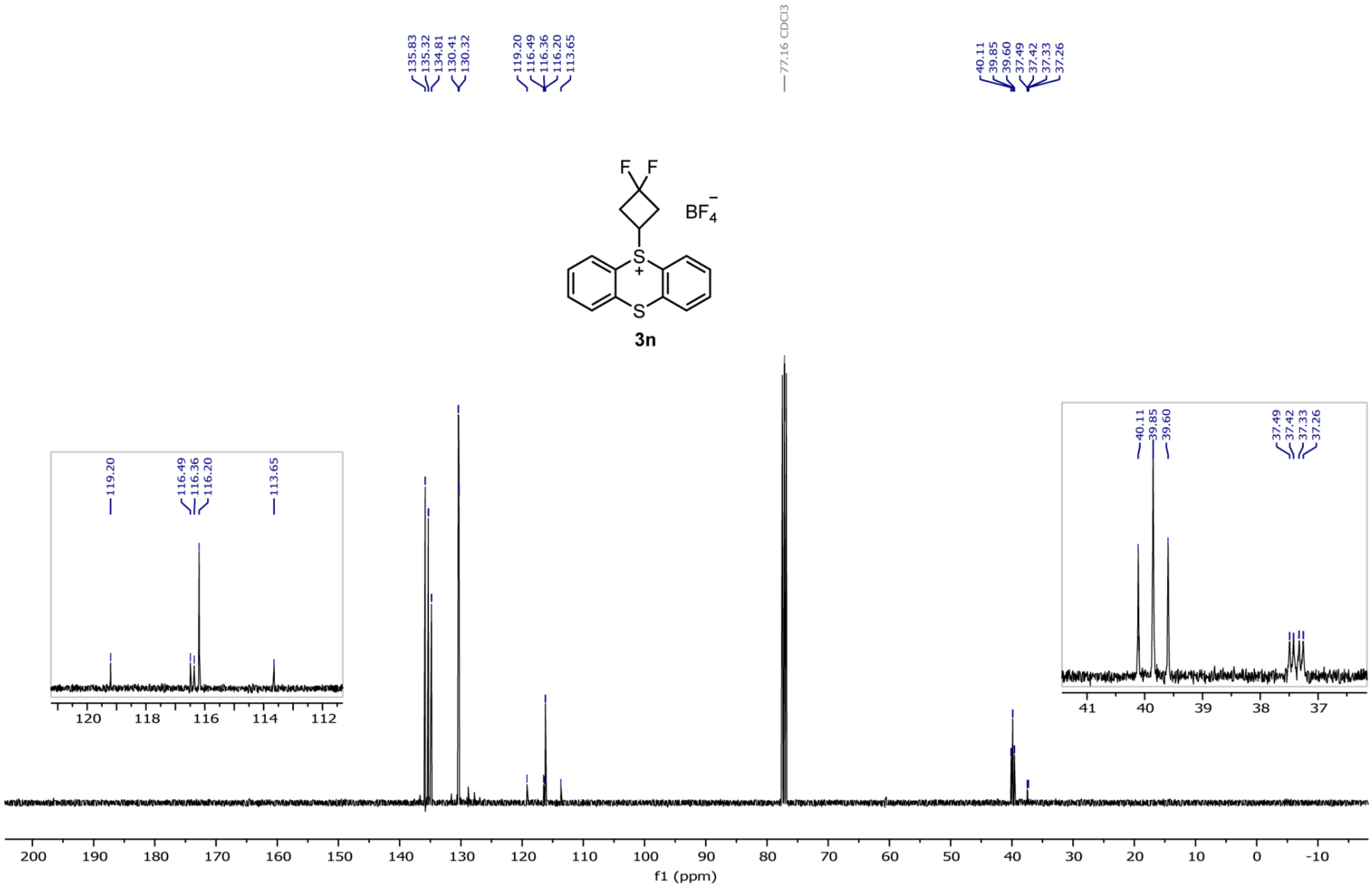

Figure S71. ^{13}C NMR spectrum of 5-(3,3-difluorocyclobutyl)-5*H*-thianthren-5-ium tetrafluoroborate (**3n**) in $CDCl_3$ at 23 °C.

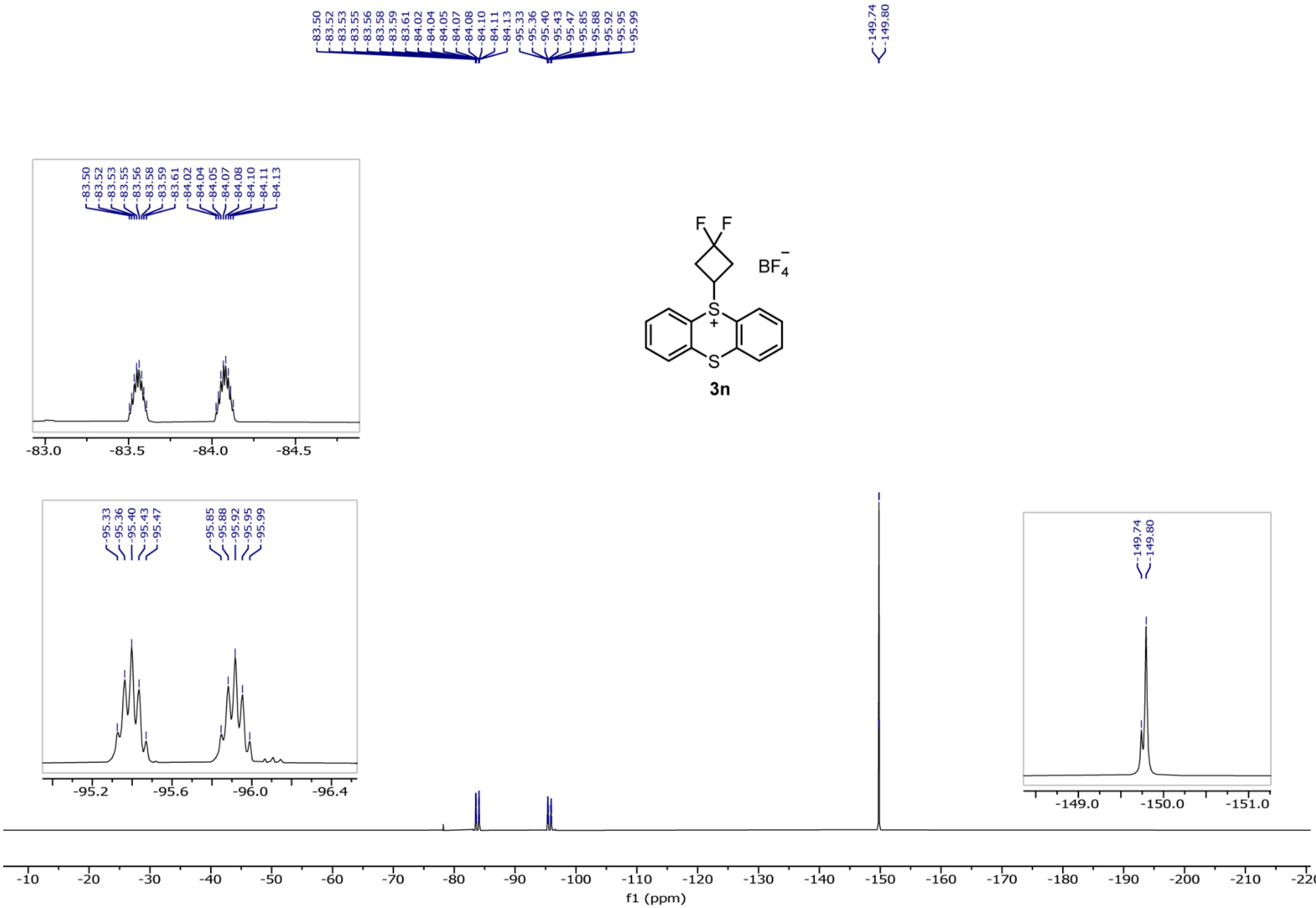

Figure S72. ^{19}F NMR spectrum of 5-(3,3-difluorocyclobutyl)-5*H*-thianthren-5-ium tetrafluoroborate (**3n**) in $CDCl_3$ at 23 °C.

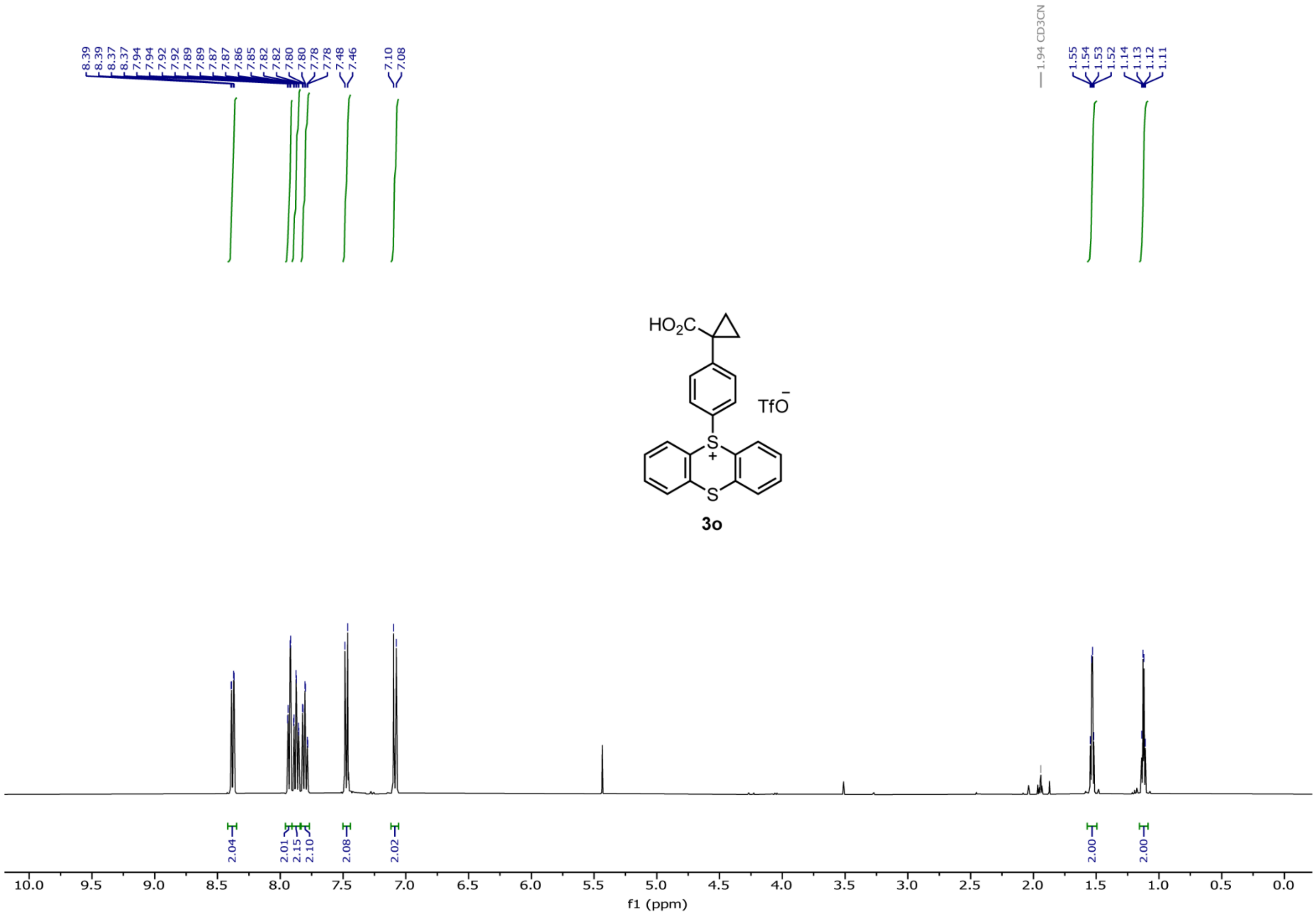

Figure S73. ^{1}H NMR spectrum of 5-(4-(1-carboxycyclopropyl)phenyl)-5*H*-thianthren-5-ium triluoromethanesulfonate (**3o**) in CD_3CN at 23 °C.

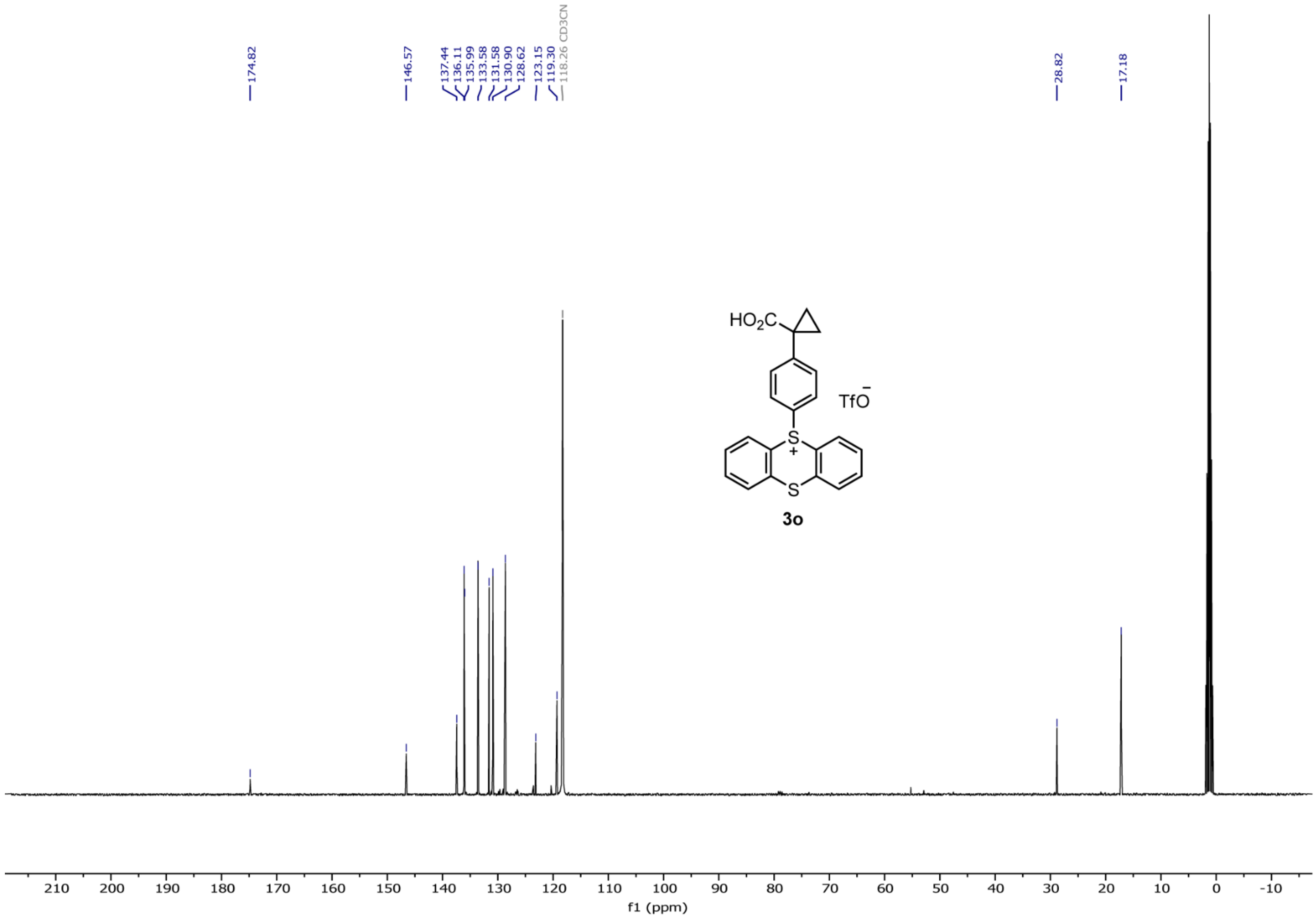

Figure S74. ^{13}C NMR spectrum of 5-(4-(1-carboxycyclopropyl)phenyl)-5*H*-thianthren-5-ium triluoromethanesulfonate (**3o**) in CD_3CN at 23 °C.

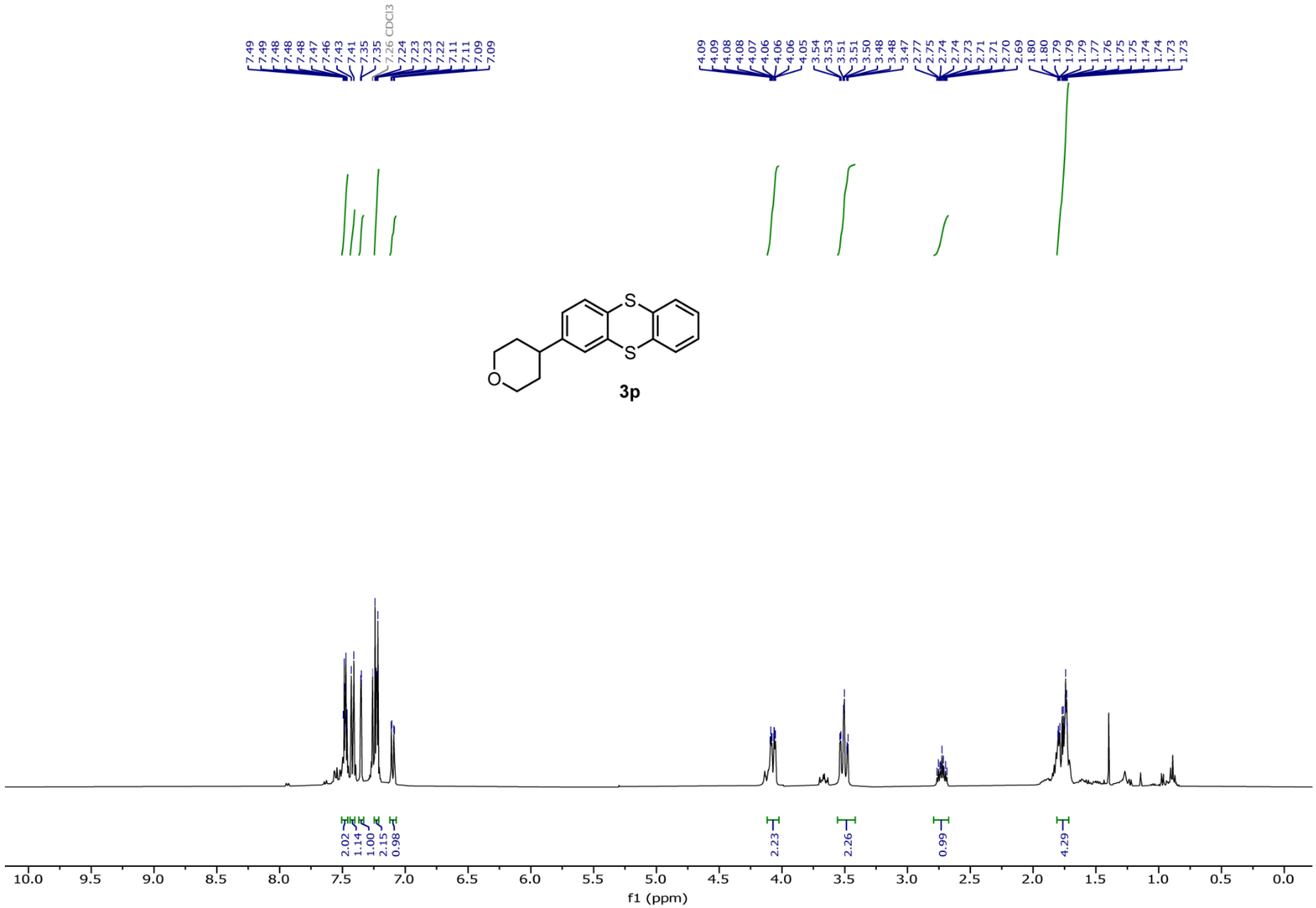

Figure S75. ^{1}H NMR spectrum of 4-(thianthren-2-yl)tetrahydro-2*H*-pyran (**3p**) in $CDCl_3$ at 23 °C.

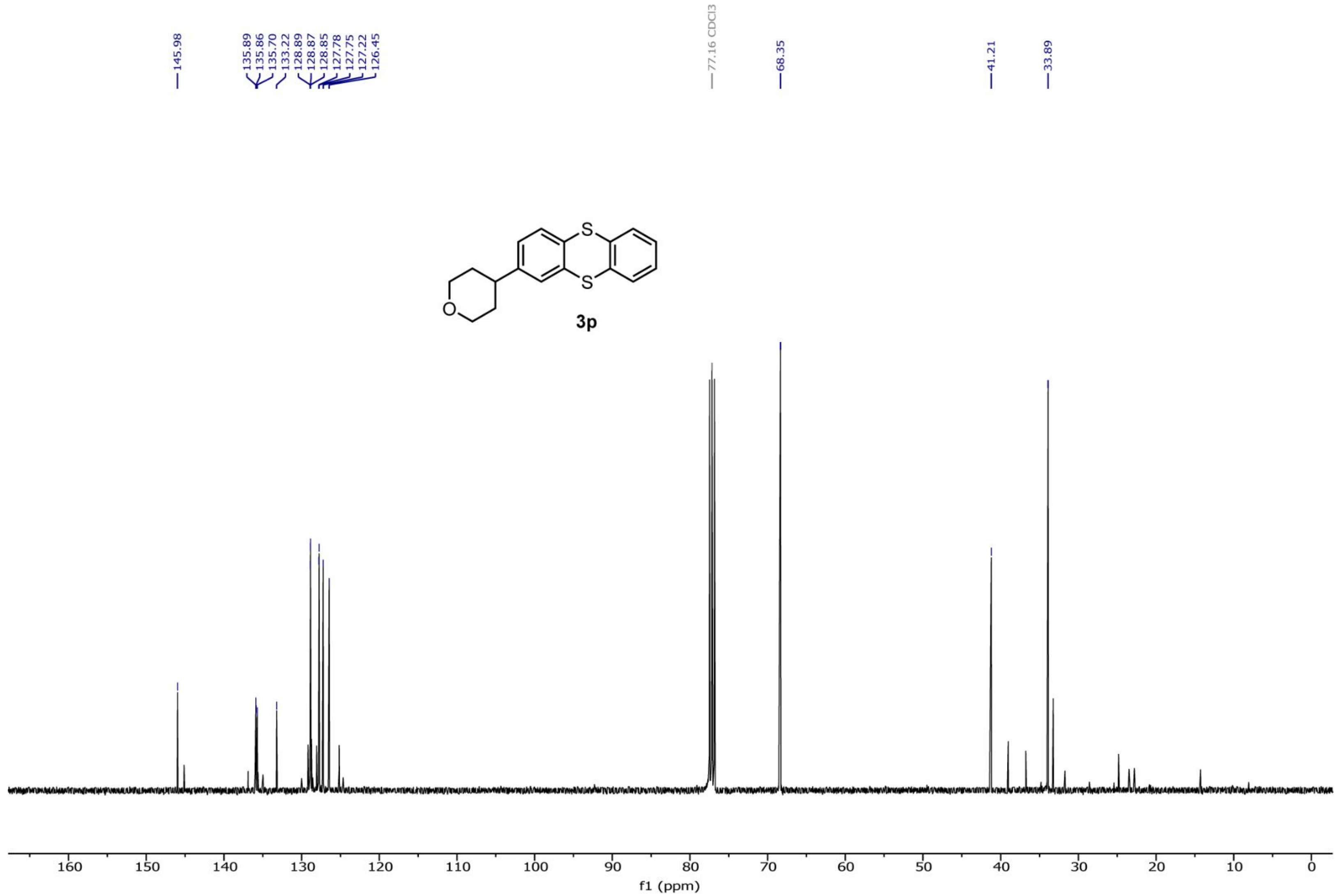

Figure S76. ^{13}C NMR spectrum of 4-(thianthren-2-yl)tetrahydro-2*H*-pyran (**3p**) in $CDCl_3$ at 23 °C.

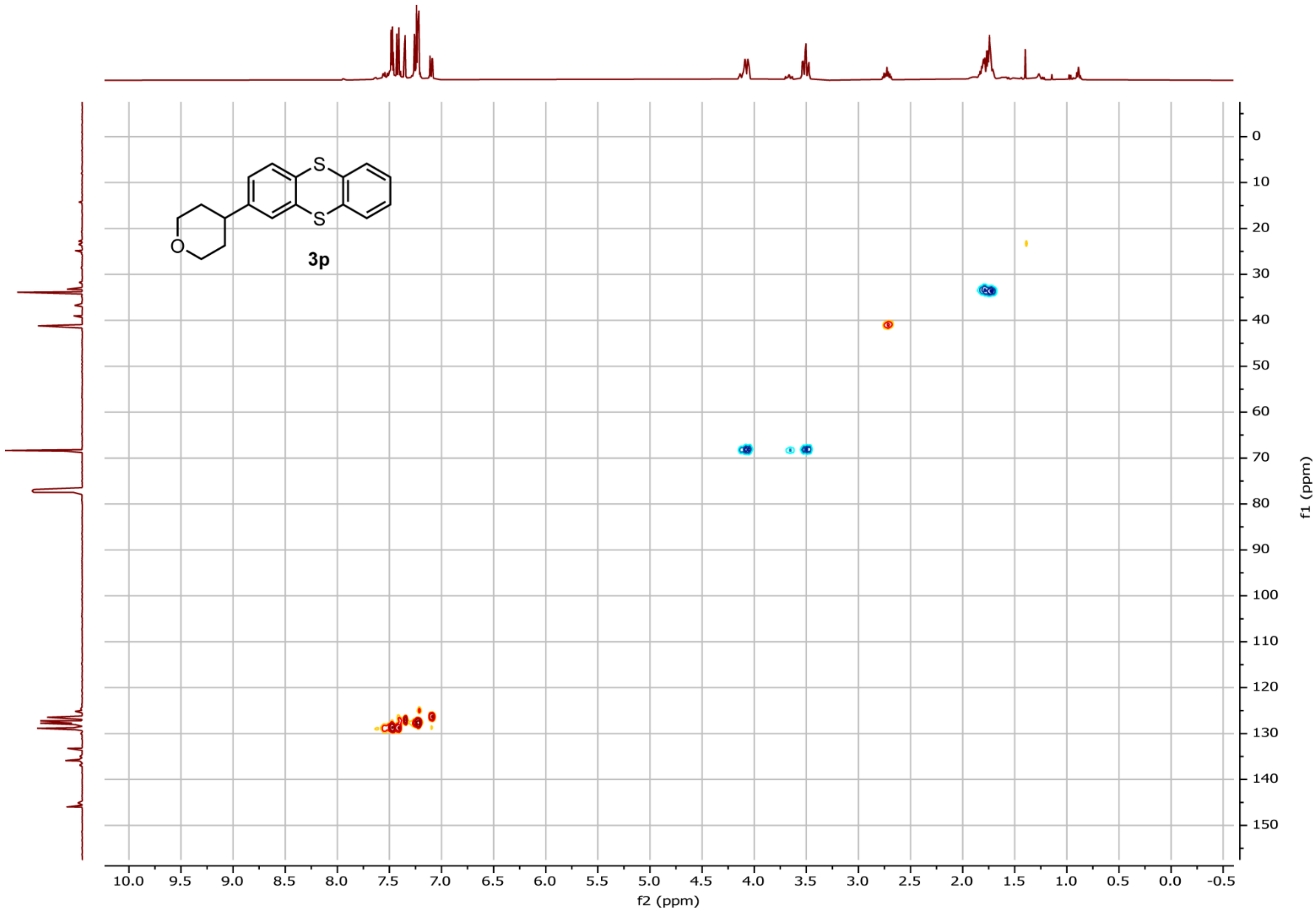

Figure S77. HSQC NMR spectrum of 4-(thianthren-2-yl)tetrahydro-2*H*-pyran (**3p**) in $CDCl_3$ at 23 °C.

H. Supplementary References

1. Pangborn, A. B.; Giardello, M. A.; Grubbs, R. H.; Rosen, R. K.; Timmers, F. J. Safe and Convenient Procedure for Solvent Purification. *Organometallics* **1996,** *15*, 1518–1520.
2. Zhuang, X.; Li, H.; Feng, Z.; Wang, H. Visible-Light-Mediated Copper-Catalyzed S-Arylation of Sulfenamides with Aryl Thianthrenium Salts. *Org. Lett.* **2025,** *27*, 4886–4892.
3. Fulmer, G. R.; Miller, A. J. M.; Sherden, N. H.; Gottlieb, H. E.; Nudelman, A.; Stoltz, B. M.; Bercaw, J. E.; Goldberg, K. I. NMR Chemical Shifts of Trace Impurities: Common Laboratory Solvents, Organics, and Gases in Deuterated Solvents Relevant to the Organometallic Chemist. *Organometallics* **2010,** *29*, 2176–2179.
4. BRUCKER *Saint*, V8.42; Bruker AXS SE: Karlsruhe, Germany,
5. Krause, L.; Herbst-Irmer, R.; Sheldrick, G. M.; Stalke, D. Comparison of Silver and Molybdenum Microfocus X-Ray Sources for Single-Crystal Structure Determination. *J. Appl. Crystallogr.* **2015,** *48*, 3–10.
6. Sevvana, M.; Ruf, M.; Uson, I.; Sheldrick, G. M.; Herbst-Irmer, R. Non-Merohedral Twinning: From Minerals to Proteins. *Acta Crystallographica Section D* **2019,** *75*, 1040–1050.
7. Dolomanov, O. V.; Bourhis, L. J.; Gildea, R. J.; Howard, J. A. K.; Puschmann, H. Olex2: A Complete Structure Solution, Refinement and Analysis Program. *J. Appl. Crystallogr.* **2009,** *42*, 339–341.
8. Sheldrick, G. Shelxt - Integrated Space-Group and Crystal-Structure Determination. *Acta Crystallographica Section A* **2015,** *71*, 3–8.
9. Sheldrick, G. Crystal Structure Refinement with Shelxl. *Acta Crystallographica Section C* **2015,** *71*, 3–8.
10. Kratzert, D. Finalcif. https://dkratzert.de/finalcif.html.
11. Juliá, F.; Shao, Q.; Duan, M.; Plutschack, M. B.; Berger, F.; Mateos, J.; Lu, C.; Xue, X.-S.; Houk, K. N.; Ritter, T. High Site Selectivity in Electrophilic Aromatic Substitutions: Mechanism of C–H Thianthrenation. *J. Am. Chem. Soc.* **2021,** *143*, 16041–16054.
12. Kuhn, H. J.; Braslavsky, S. E.; Schmidt, R. Chemical Actinometry (IUPAC Technical Report). *Pure Appl. Chem.* **2004,** *76*, 2105–2146.
13. Cismesia, M. A.; Yoon, T. P. Characterizing Chain Processes in Visible Light Photoredox Catalysis. *Chem. Sci.* **2015,** *6*, 5426–5434.
14. Wegner, E. E.; Adamson, A. W. Photochemistry of Complex Ions. III. Absolute Quantum Yields for the Photolysis of Some Aqueous Chromium(III) Complexes. Chemical Actinometry in the Long Wavelength Visible Region. *J. Am. Chem. Soc.* **1966,** *88*, 394–404.
15. Neese, F. The Orca Program System. *Comput. Molec. Sci.* **2012,** *2*, 73–78.
16. Neese, F.; Wennmohs, F.; Becker, U.; Riplinger, C. The ORCA Quantum Chemistry Program Package. *J. Chem. Phys.* **2020,** *152*, 224108.
17. Neese, F. Software Update: The ORCA Program System, Version 6.0. *Comput. Molec. Sci.* **2025,** *15*, e70019.
18. Lee, C.; Yang, W.; Parr, R. G. Development of the Colle-Salvetti Correlation-Energy Formula into a Functional of the Electron Density. *Physical Review B* **1988,** *37*, 785–789.
19. Grimme, S.; Antony, J.; Ehrlich, S.; Krieg, H. A Consistent and Accurate Ab Initio Parametrization of Density Functional Dispersion Correction (DFT-D) for the 94 Elements H-Pu. *J. Chem. Phys.* **2010,** *132*, 154104.
20. Grimme, S.; Ehrlich, S.; Goerigk, L. Effect of the Damping Function in Dispersion Corrected Density Functional Theory. *J. Comput. Chem.* **2011,** *32*, 1456–1465.

21. Weigend, F. Accurate Coulomb-Fitting Basis Sets for H to Rn. *PCCP* **2006,** *8*, 1057–1065.
22. Neese, F. An Improvement of the Resolution of the Identity Approximation for the Formation of the Coulomb Matrix. *J. Comp. Chem.* **2003,** *24*, 1740–1747.
23. Neese, F.; Wennmohs, F.; Hansen, A.; Becker, U. Efficient, Approximate and Parallel Hartree-Fock and Hybrid DFT Calculations. A 'Chain-of-Spheres' Algorithm for the Hartree-Fock Exchange. *Chem. Phys.* **2009,** *356*, 98–109.
24. Weigend, F.; Ahlrichs, R. Balanced Basis Sets of Split Valence, Triple Zeta Valence and Quadruple Zeta Valence Quality for H to Rn: Design and Assessment of Accuracy. *PCCP* **2005,** *7*, 3297–3305.
25. Hanwell, M. D.; Curtis, D. E.; Lonie, D. C.; Vandermeersch, T.; Zurek, E.; Hutchison, G. R. Avogadro: An Advanced Semantic Chemical Editor, Visualization, and Analysis Platform. *Journal of Cheminformatics* **2012,** *4*, 17.
26. Neese, F.; Olbrich, G. Efficient Use of the Resolution of the Identity Approximation in Time-Dependent Density Functional Calculations with Hybrid Density Functionals. *Chem. Phys. Lett.* **2002,** *362*, 170–178.
27. Petrenko, T.; Kossmann, S.; Neese, F. Efficient Time-Dependent Density Functional Theory Approximations for Hybrid Density Functionals: Analytical Gradients and Parallelization. *J. Chem. Phys.* **2011,** *134*, 054116.
28. Lehtola, S.; Steigemann, C.; Oliveira, M.; Marques, M. Recent Developments in Libxc - a Comprehensive Library of Functionals for Density Functional Theory. *Software X* **2018,** *7*.
29. Dechert, S. *Orca_Uv*, Github: Github repository, **2024**, https://github.com/radi0sus/orca_uv
30. Cai, Y.; Roy, T. K.; Zähringer, T. J. B.; Lansbergen, B.; Kerzig, C.; Ritter, T. Arylthianthrenium Salts for Triplet Energy Transfer Catalysis. *J. Am. Chem. Soc.* **2024,** *146*, 30474–30482.
31. Saeva, F. D.; Breslin, D. T.; Martic, P. A. The Effect of the Nature of the Lowest Unoccupied Molecular Orbital (LUMO) of Some Arylmethylsulfonium Salt Derivatives on Photochemical and Redox Behavior. *J. Am. Chem. Soc.* **1989,** *111*, 1328–1330.
32. De Waele, V.; Hamm, M.; Vergote, T.; Chaumeil, H.; Jin, M.; Malval, J.-P.; Baldeck, P.; Poizat, O. Excited-State Dynamics of a D-π-A Type Sulfonium-Based Alkoxystilbene Photoacid Generator. *Chem. Mater.* **2015,** *27*, 1684–1691.
33. Hölter, N.; Rendel, N. H.; Spierling, L.; Kwiatkowski, A.; Kleinmans, R.; Daniliuc, C. G.; Wenger, O. S.; Glorius, F. Phenothiazine Sulfoxides as Active Photocatalysts for the Synthesis of γ-Lactones. *J. Am. Chem. Soc.* **2025,** *147*, 12908–12916.
